# High-resolution antibody array analysis of proteins from primary human keratinocytes and leukocytes

**DOI:** 10.1371/journal.pone.0209271

**Published:** 2018-12-27

**Authors:** Daniel de la Rosa Carrillo, Krzysztof Sikorski, Denis Khnykin, Weiwei Wu, Fridtjof Lund-Johansen

**Affiliations:** 1 Institute of Clinical Medicine, University of Oslo, Oslo, Norway; 2 Department of Dermatology, Oslo University Hospital, Oslo, Norway; 3 Department of Immunology, Oslo University Hospital, Oslo, Norway; 4 K.G. Jebsen Center for Cancer Immunotherapy, University of Oslo, Oslo, Norway; 5 Department of Pathology, Oslo University Hospital, Oslo, Norway; Duke University School of Medicine, UNITED STATES

## Abstract

Antibody array analysis of labeled proteomes has high throughput and is simple to perform, but validation remains challenging. Here, we used differential detergent fractionation and size exclusion chromatography in sequence for high-resolution separation of biotinylated proteins from human primary keratinocytes and leukocytes. Ninety-six sample fractions from each cell type were analyzed with microsphere-based antibody arrays and flow cytometry (microsphere affinity proteomics; MAP). Monomeric proteins and multi-molecular complexes in the cytosol, cytoplasmic organelles, membranes and nuclei were resolved as discrete peaks of antibody reactivity across the fractions. The fractionation also provided a two-dimensional matrix for assessment of specificity. Thus, antibody reactivity peaks were considered to represent specific binding if the position in the matrix was in agreement with published information about i) subcellular location, ii) size of the intended target, and iii) cell type-dependent variation in protein expression. Similarities in the reactivity patterns of either different antibodies to the same protein or antibodies to similar proteins were used as additional supporting evidence. This approach provided validation of several hundred proteins and identification of monomeric proteins and protein complexes. High-resolution MAP solves many of the problems associated with obtaining specificity with immobilized antibodies and a protein label. Thus, laboratories with access to chromatography and flow cytometry can perform large-scale protein analysis on a daily basis. This opens new possibilities for cell biology research in dermatology and validation of antibodies.

## Introduction

Proteomics technology opens for a systems-based approach to cell biology research [1], but implementation of mass spectrometry (MS) is challenging due to the low throughput and complexity of the technique and restricted access to high-resolution instruments [2]. An attractive option is to multiplex detection with antibodies [3]. Antibody array analysis of labeled proteomes has far higher throughput than MS and is simple to perform [4]. However, the technology has yet to see widespread use due to concerns about the specificity. Assays based on the use of immobilized binders and a protein label are reliable when the capture reagent is mono-specific. However, antibodies to cellular proteins frequently cross-react, and the issue is further complicated by the fact that many cellular proteins occur in multi-molecular complexes. It has been suggested that widespread use of antibody array technology may have to await the development of new and better capture reagents that are specially designed for the application [5].

By implementing sample fractionation, it is feasible to use an assay format for antibody array analysis that resembles a multiplexed western blot [6]. This allows the use of "off the shelf" commercially available antibodies [7]. Bead-based arrays that are measured by flow cytometry are particularly useful for this application since they can be handled in microwell plates to allow parallel processing of large numbers of sample fractions. The term microsphere affinity proteomics (MAP) seems appropriate to discriminate the format from measurements with planar antibody arrays. In earlier studies, MAP has been combined with size exclusion chromatography (SEC), which is used to separate native proteins in context of their interaction partners [7, 8]. Results obtained by MAP analysis of a series of SEC fractions are used to generate size distribution profiles for the targets of all capture antibodies. With this approach, different proteins and protein complexes capable of binding to the same capture antibody are detected independently as peaks of antibody reactivity across the fractions.

The use of SEC is rational for cell biology research, since many of the functional units in cells consist of multi-molecular complexes. However, the elution profile of a given protein may be complex if it occurs both as a monomer and in one or more multi-molecular complexes [6, 7, 9, 10]. Moreover, there is currently no publically available database for SEC elution profiles. It may therefore be difficult to determine whether a given antibody reactivity peak corresponds to specific binding or cross-reactivity. Currently, it is necessary to use different antibodies to the same protein as an internal reference [6, 7, 8]. Since only 25–30% of antibodies perform well in the assay, the need for multiple antibodies per target is an important limiting factor [7].

The aim of the present study was to obtain better control of specificity in MAP. The model was the validation process used in the Human Protein Atlas (HPA, proteinatlas.org). The HPA is the largest and most systematic effort to date to produce and validate antibodies to human proteins. Antibodies are regarded as specific if their reactivity profiles are in agreement with published information about i) the size of the intended target (western blotting), ii) the subcellular localization (confocal microscopy), and iii) tissue distribution (immunohistochemistry). Finally, similarity in the reactivity profiles of different antibodies to the same protein is used as supportive evidence.

**Fig 1:**
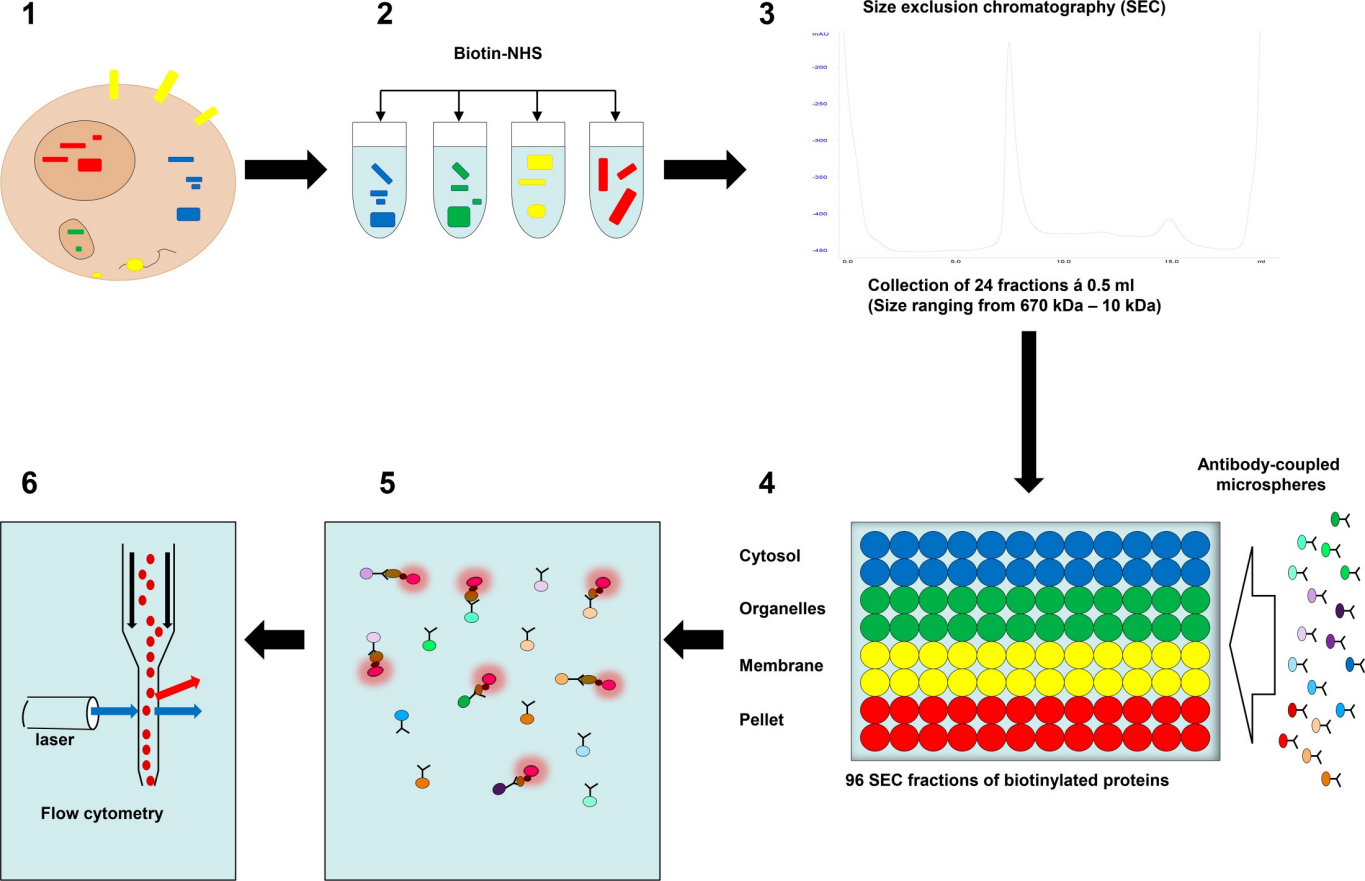
Microsphere affinity proteomics (MAP). **1–2:** Proteins from different subcellular compartments were lysed and labeled with amine-reactive biotin (Biotin-NHS). **3:** The biotinylated proteins were separated using a size exclusion chromatography (SEC) column (Superdex 200). **4:** A mixture of color-coded microspheres with antibodies to cellular proteins was added to all SEC fractions and the microspheres rotated overnight at 4oC. **5:** Microspheres were washed and then labeled with R-Phycoerythrin-conjugated streptavidin (SA-PE, red flashing circles). **6:** Finally, the microspheres were analyzed by flow cytometry.

In an earlier study by our group, SEC was combined with a crude method to separate soluble proteins from those tightly bound to membranes and chromatin [6]. Here, we aimed to obtain more precise information about subcellular location. On the basis of conclusions made in a recent review on methods for subcellular fractionation [11], we chose to rely on differential detergent fractionation. Published protocols involve successive treatment of cells with digitonin to permeabilize the plasma membrane, Triton X-100, to extract proteins from membranes and soluble proteins in organelles, and finally, anionic detergents to disrupt nuclei [12, 13, 14]. To further enhance the resolution, we sought to obtain a fourth fraction enriched for soluble organelle proteins. Thus, the mild detergent Brij 98 was used to permeabilize intracellular membranes in digitonin-treated cells prior to extraction of membrane proteins with NP-40 (Nonidet P40; octylphenoxypolyethoxyethanol) [15]. We also replaced anionic detergents with salt extraction to prevent denaturation of nuclear proteins [16]. Proteins in the four subcellular fractions were biotinylated and subjected to SEC for resolution of protein size (Fig 1). As reference for results obtained by MAP, we used annotations on the subcellular localization from the Compartments database (S1 Table). We also included results from a recent meta-analysis of subcellular proteomics data [11] (S1 Table).

To assess cell type-dependent variation in protein expression we measured proteins from human leukocytes (peripheral blood mononuclear cells; PBMC) and keratinocytes (human primary keratinocytes; HPK). We also compared results obtained with proteins from quiescent and proliferating cells. This should provide a reference of specificity based on the functional state of the cells, since the proteins in the cell-cycle machinery are differently expressed in quiescent and dividing cells. Finally, different antibodies to the same protein were used as an additional internal reference when these were available.

The combination of extensive protein separation and comparative analysis of different cell types provided high resolution of antibody targets and a complex matrix of reference to assess specificity. Thus, high-resolution MAP represents a high-throughput alternative to MS for laboratories that have access to chromatography and flow cytometry.

## Materials and methods

### Antibodies and antibody arrays

The antibodies used in the present study are listed in S1 Table.

### Microsphere-based antibody arrays

It is worth noting that microspheres with up to 500 fluorescent bar codes are commercially available from Luminex corporation [17]. The procedure for production of the in-house arrays used here has been described in detail previously [6, 7] Briefly, amine functionalized polymethyl-metha- acrylate (PMMA) microspheres (Bangs Laboratories, IN, USA) were first reacted with the hetero-bifunctional crosslinker succinimidyl 3-(2-pyridyldithio)propionate (SPDP, 50ug/ml, Sigma) and reduced with 5mM TCEP (Sigma) to obtain thiol-functionalized beads. The thiol groups were first used as binding sites for maleimide-derivatized Protein A/G (ProSpec-Tany TechnoGene Ltd, IL). Remaining thiols were used to bind serially diluted solutions of maleimide-derivatives of fluorescent dyes: Alexa-488 (five levels), Alexa-647 (five levels), Pacific Orange (three levels) and Pacific Blue (four levels). This yields a multiplexing capacity of 300. Antibodies from rabbit and goat were coupled directly to protein-A/G beads. For binding of mouse antibodies, the beads were first coupled with goat antibodies to mouse IgG subclasses (Jackson Immunoresearch). Bar-coded microspheres were kept separate in 384 well plates until completion of the antibody-coupling step. The beads were next mixed suspended in PBS Casein Block buffer (Thermo Fisher) and stored at -70°C until use. Bead mixtures were stable for more than a year [18].

### Skin samples

Human skin samples were obtained from healthy adult patients who underwent plastic surgery for breast or abdominal reduction. Age of volunteers ranged between 36 and 48 years. Written consent was obtained. The study was approved by the local ethical committee (Regional etisk komité, study number S-08374a).

### Isolation and culture of primary human leukocytes and keratinocytes

HPK were harvested from skin biopsies (12–18 5 mm punch biopsies per patient sample). These were washed with phosphate buffered saline (PBS) and incubated in sterilized 0.25% Dispase II solution in Roswell Park Memorial Institute (RPMI) medium with Hepes buffer at 4 ^0^C for 20 hours. Epidermis was then removed, washed with PBS and transferred to 5ml 0.25% trypsin with ethylenediaminetetraacetic acid (EDTA). Samples were incubated at 37 ^0^C for 15 minutes, shaking every five minutes. Cold 10% fetal calf serum (FCS) solution was then added to stop trypsinization. Samples were left on ice for one minute, filtered through a nylon filter (40–100 μm), washed in RPMI 1640 medium, then re-suspended in fully supplemented CnT-57, a progenitor cell targeted epidermal keratinocyte medium with low bovine pituitary extract with added gentamycin, and finally seeded into 2.5 cm^2^ collagen-coated flasks (1–2 million cells per flask). For each patient sample, two flasks were allowed to grow in CnT-57 medium until the cells reached 70–80% confluence (i.e. non-confluent sample) prior to cell lysis. Two other flasks were allowed to reach 100% confluence and were kept confluent in CnT-57 medium for ten days prior to cell lysis (i.e. confluent sample).

Prior to harvesting of cells, culture flasks were washed twice with cold PBS and incubated with PBS containing 2 mM EDTA at 37°C for 10 minutes. Cells were then scraped off from the flask bottom, transferred to 15 ml tubes and sedimented by centrifugation at 500x g for 5 minutes.

PBMC from healthy laboratory personnel were isolated using Ficoll. Subcellular fractions were obtained immediately after isolation of the cells (see below).

### Subcellular fractionation

Cells were resuspended in 350 μl of a solution containing 140 mM NaCl, 30 mM HEPES pH8, 1 mM Tris(2-carboxyethyl)phosphine hydrochloride (TCEP), protease inhibitors (Sigma cat. no P8340), 1 mM phenylmethylsulfonyl fluoride (PMSF) and phosphatase inhibitors (Sigma cat no P5726) (Buffer A). To permeabilize the plasma membrane, cells were treated with the cholesterol-binding glycoside digitonin (0.015%) at 4°C under constant shaking for 15 min [12]. The permeabilized cells were pelleted by centrifugation at 500x g for 5 minutes, and the supernatant was used as the cytoplasmic fraction (C). The concentration in the cytoplasmic fraction was 1–2 mg/ml. Cells were next re-suspended in buffer A containing 0.2% Brij 98 to permeabilize intracellular membranes. After 10 minutes of rotation at 4°C, the permeabilized cells were pelleted again, and the supernatant was kept as the organelle fraction (O). Membrane proteins were solubilized by treating the pellet with buffer A containing 0.2% NP-40. The pellet obtained after centrifugation at 1000x g was re-suspended in a solution containing 400 mM NaCl, 30 mM HEPES pH8, 1 mM TCEP, protease inhibitors (Sigma cat. no P8340), 1 mM PMSF and phosphatase inhibitors (Sigma cat no P5726) (Buffer B) and 1% dodecyl maltoside (DDM), and sonicated to extract membrane proteins that were insoluble in NP-40 and nuclear proteins. All four subcellular fractions were centrifuged at 14.000x g for 10 minutes to remove insoluble material and stored at -70°C until use.

### Protein labeling, size exclusion chromatography (SEC) and antibody array analysis

Proteins in subcellular fractions were labeled with biotin, fractionated by SEC and analyzed with bead-based antibody arrays and flow cytometry as described previously [6]. The steps are illustrated in Fig 1. Following subcellular fractionation (1) proteins reacted with 1 mg/ml biotin-PEO_4_-N-hydroxy-succinimidyl ester (Thermo Scientific) (2), incubated for 30 minutes on ice to label proteins and fractionated using an Äkta FPLC system with a Superdex 200 column (GE-Lifesciences, separation range 10-670kDa) (3). The running buffer was phosphate-buffered saline with 0.05% Tween 20. For all keratinocyte experiments a duplicate was prepared where aliquots from each fraction were denatured with 0.07% sodium dodecyl sulfate (SDS) as previously described [7]. Fractionated proteins were incubated overnight at 4°C with bead-based antibody arrays in 96 well plates (4), and captured proteins were labeled with streptavidin-phycoerythrin (SA-PE) (5). The microspheres were analyzed with an LSR II flow cytometer equipped with a unit that harvests samples directly from 96 well plates (BD biosciences) (6).

### Data analysis

The flow cytometry data files (fcs3.0) were exported and analyzed using dedicated software for analysis of MAP data [19]. The software identifies microsphere subsets with different color codes semi-automatically and exports the median SA-PE fluorescence value for each subset into a text file. The text files were imported into Microsoft Excel to generate line plots where the SA-PE fluorescence value for each of the 24 SEC fractions from each subcellular fraction was displayed as colored lines. Prior to generation of line plots, background signal from microspheres coupled with non-immune IgG was subtracted. The line charts were formatted to mimic western blotting. In that application, the signals are linear, and the blot is exposed to show optimal resolution of the intended antibody target. Here we normalized the line charts to the maximal signal value. Thus, the scale is the same on all charts showing results obtained with the same antibody in different samples.

### Bio-informatics

In S1 Table, results obtained by MAP are shown next to annotations on the subcellular localization from the Compartments database [20]. This database contains annotations from a wide range of sources and is currently used as reference in the Uniprot database. The table also contains a summary of results from six studies where mass spectrometry was used to analyze subcellular fractions. The datasets and the studies are described in detail elsewhere [11]. Raw data is also provided (S2 Table).

## Results

### The combination of subcellular fractionation, SEC and MAP enables high-resolution detection of proteins and protein complexes in subcellular compartments

**Fig 2:**
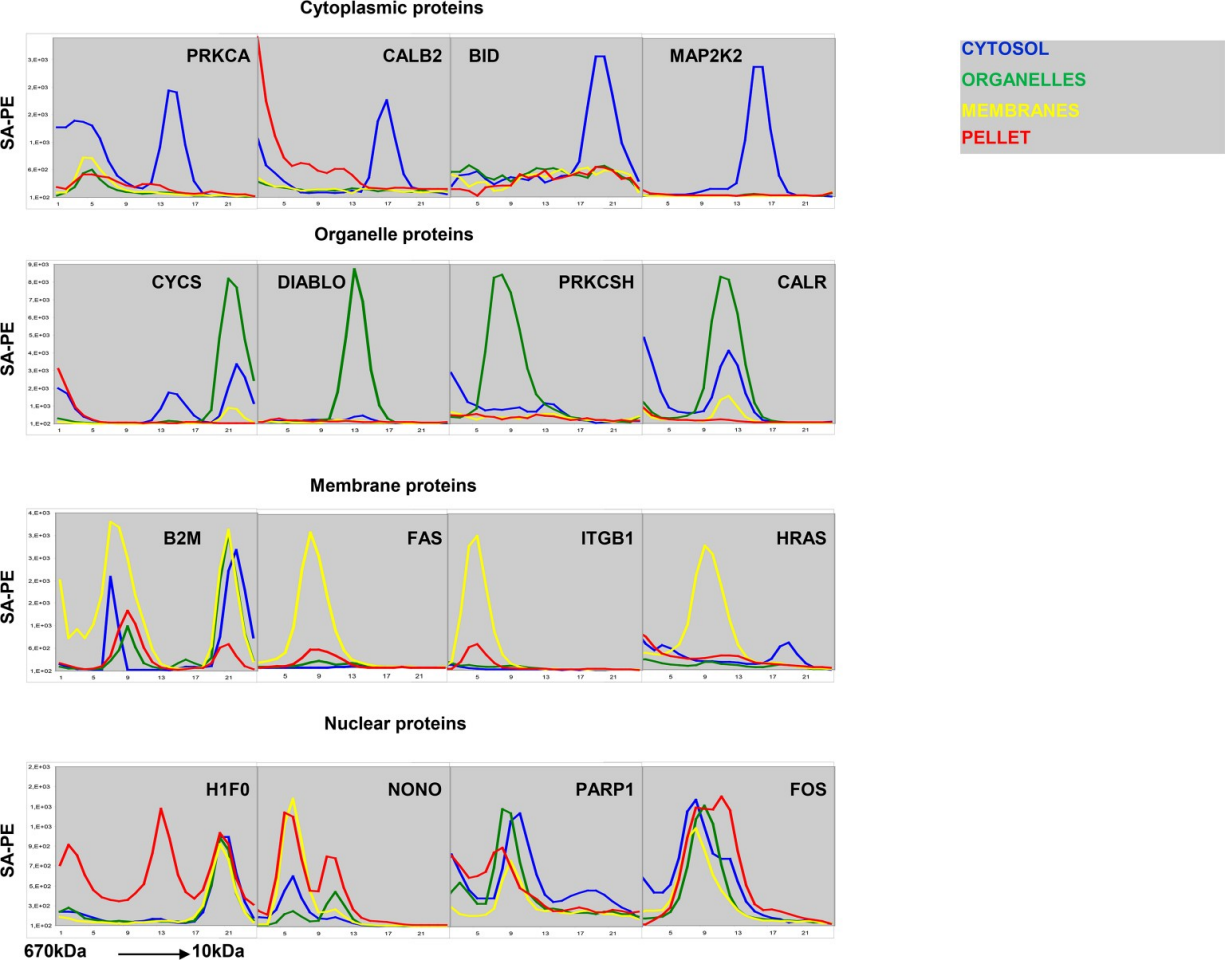
Content of PRKCA, CALB2, BID, MAP2K2, CYCS, DIABLO, PRKCSH, CALR, B2M, FAS, ITGB1, HRAS, H1F0, NONO, PARP1 AND FOS in human primary keratinocytes. The line plots show SA-PE signal intensity of microspheres coupled with indicated specificity (y-axis), plotted against SEC fraction number (fractions 1–24 i.e. protein size 670–10 kDa, x-axis). The first row shows proteins mainly found in the cytosolic fraction, the second row shows proteins mainly found in organelles, the third row shows proteins mainly found in the membrane fraction, and the fourth row shows proteins mainly found in the nuclear fraction. The differently colored lines in each plot correspond to results obtained from each subcellular fraction (cytosol = blue, organelles = green, membranes = yellow, pellet = red). The results show average of three similar experiments.

Antibodies often capture multiple targets from complex samples such as cell lysates. Here, we separated cellular proteins according to their subcellular localization and size prior to measurement with bead-based antibody arrays. The line plots in Fig 2 show fluorescence from captured protein (y-axis) plotted against SEC fraction number (x-axis). The colored lines in each plot correspond to results obtained with the cytosol fraction (blue), organelle fraction (green), membrane fraction (yellow) and nuclear fraction (red), respectively. Antibodies to PRKCA and CALB2 bound at least two targets (Fig 2). Yet, the specific targets were readily detected by analyzing results in context of reactivity peaks in the cytoplasm corresponding to the expected elution point for the monomeric forms, and the subcellular distribution is in good accordance with published data (S1 Table). Similar results were obtained with antibodies to BID and MAP2K2, which also are known to occur in the cytoplasm (Fig 2, S1 Table).

Cytochrome c (CYCS), Diablo, PRKCSH and Calreticulin (CALR) occur in the lumen of cytoplasmic organelles (S1 Table). Antibodies to these proteins captured targets that were released when digitonin-permeabilized cells were treated with Brij 98 to permeabilize organelle membranes (Fig 2, green lines). Antibodies to CYCS and CALR also captured proteins that eluted earlier in SEC (Fig 2). However, the specific targets are readily recognized on the basis of the subcellular localization, and the expected elution point for the monomeric forms (Fig 2, S1 Table). The size distribution profile of DIABLO is compatible with its occurrence as a dimer, and PRKCSH eluted as expected for a protein occurring in complex with the larger protein GANAB (S1 Table).

Membrane proteins elute early in SEC due to their association with detergent micelles [6]. As demonstrated by the results described above, many antibodies show non-specific binding in early eluting fractions. The subcellular distribution profiles of antibody targets were therefore highly useful to interpret the results. The major targets of antibodies to FAS, ITGB1 and H-RAS proteins were hardly detectable in the digitonin and Brij 98 extracts (Fig 2, green lines). After treatment with NP-40, the proteins were solubilized (Fig 2, yellow lines). The antibody to B2M captured an additional target found in all fractions except the nuclear fraction. The elution profile corresponds well with the monomeric form of the protein, and the target may therefore correspond to free intracellular B2M. A second target was also found for anti-HRAS (Fig 2). The elution profile was compatible with the monomeric form of the protein. However, while HRAS associates with membranes, a late-eluting peak was found in the cytoplasmic fraction (Fig 2). This target may therefore reflect cross-reactivtity.

Antibodies to the nuclear proteins Histone 1 (H1F0), PARP1, Fos and the nucleolar protein NONO captured targets from all fractions (Fig 2). Thus, the profiles were distinct from those obtained with antibodies to proteins localized in the cytoplasm, cytoplasmic organelles, and membranes. However, it seems clear that the protocol used here causes extensive leakage of nuclear proteins, and the results obtained with these proteins should therefore be interpreted with care.

### Tissue-specific protein profiling

**Fig 3:**
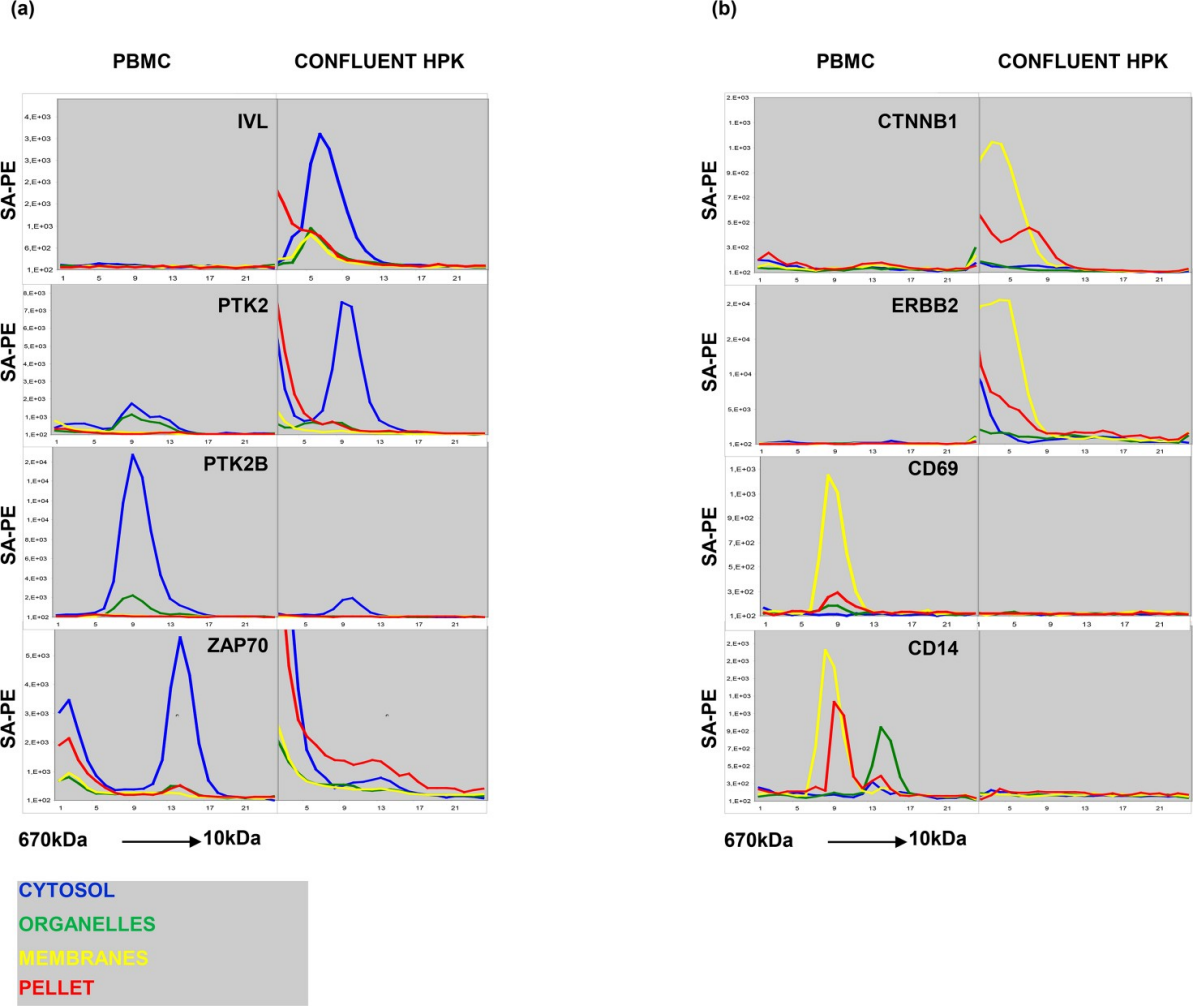
**(A) Content of IVL, PTK2, PTK2B, ZAP70 in peripheral blood mononuclear cells (PBMC) and confluent human peripheral keratinocytes (HPK).** The line plots have the same disposition as Fig 2. **(B) Content of CTNNB1, ERBB2, CD69 AND CD14 in PBMC and confluent HPK.** The line plots have the same disposition as Fig 3A. The results show average of three similar experiments.

Since no method yields fractions with absolute purity, additional criteria to assess specificity are clearly beneficial. One useful parameter is cell type-restricted expression. For example, an antibody to involucrin (IVL) captured a protein from the cytosol of keratinocytes, but not from leukocytes (Fig 3). The subcellular distribution is in line with annotations in Uniprot (S1 Table). The elution profile is also compatible with the occurrence of the protein in a multi-molecular complex (S1 Table). However, elution profiles of multi-molecular complexes are less reliable than those of monomeric proteins since many antibodies capture targets non-specifically from the early fractions of SEC elution [6, 7, 8]. The results showing that anti-IVL antibody captured a protein selectively from keratinocytes simplify the interpretation of the results. The cell type-restricted expression is in good agreement with annotations on the tissue distribution of IVL in Uniprot (S1 Table) and meta-data from mRNA profiling experiments published in the In Silico Transcriptomics (IST) database, which cumulates data from over 20,000 DNA microarray experiments (ist.medsapiens.com).

The results obtained with antibodies to the two tyrosine kinases PTK2 and PTK2B also exemplify the utility of comparing results from different cell types. In the HPA, PTK2B is listed as mainly localized in the nucleus (S1 Table). By contrast, the antibody used here captured protein exclusively from the cytoplasm (Fig 3A). The results showing that the target was selectively expressed in leukocytes are in good agreement with the information in the IST database and with meta-data from MS analysis of subcellular fractions (S1 Table). The antibody to PTK2 captured a target with similar elution profile and subcellular distribution, and the results showing higher levels in keratinocytes are in good agreement with those in the IST database.

An antibody to the T cell-specific kinase Zap 70 captured a 70kDa target from the cytosol of leukocytes. Since ZAP70 is selectively expressed in leukocytes, the signal observed near the void volume in keratinocytes can safely be disregarded as cross-reactivity. Finally, the results obtained with antibodies to membrane proteins found in keratinocytes such as Her2 (ERBB2) and beta catenin (CTNNB1) and leukocytes (CD69 and CD14), are in good agreement with published tissue distribution profiles. The cell type-dependent reactivity profile was particularly useful for interpretation of the results obtained with anti-CD14. The target captured from the organelle fraction might be interpreted as cross-reactivity. However, since it was selectively found in leukocytes, the target is more likely to correspond to the secreted form of CD14 [21].

### Functional protein expression profiling

**Fig 4:**
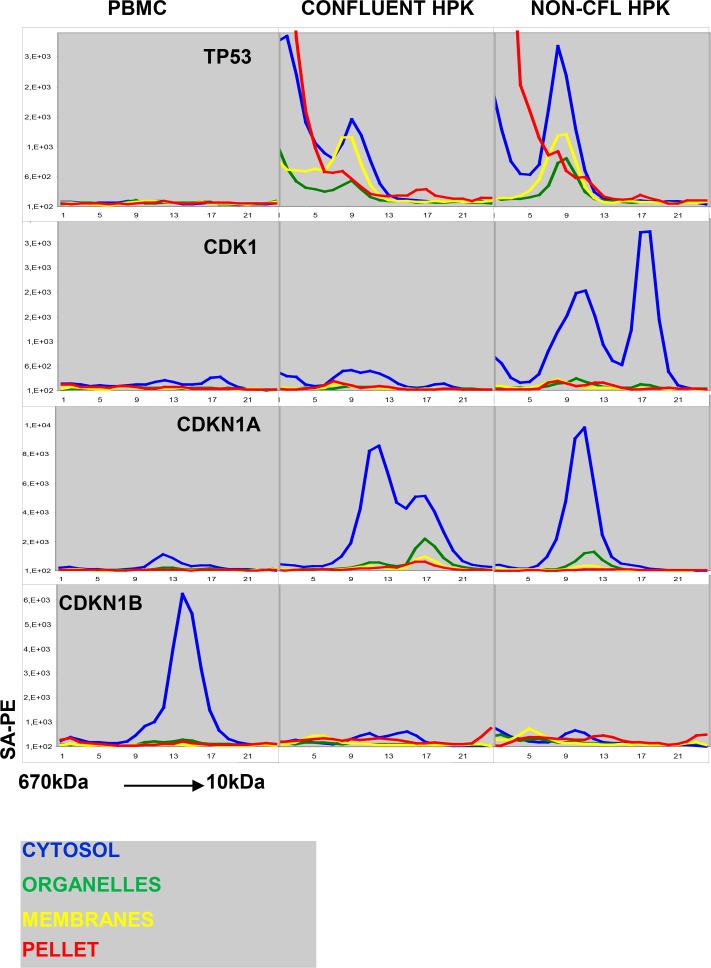
Content of TP53, CDK1, CDKN1A and CDKN1B in peripheral blood mononuclear cells (PBMC), confluent human peripheral keratinocytes (HPK) and non-confluent HPK. The line plots have the same disposition as previous figures. The results show average of three similar experiments.

The cell-cycle machinery is one of the best-characterized cellular protein networks. Thus, the expression and context of many of the proteins can be predicted from the cell-cycle status (Fig 4). The elution profile of the target for anti-TP53 in keratinocyte lysates is in accordance with the occurrence of the protein as a tetramer (Fig 4). However, cross-reactivity in early SEC fractions is common, and the high levels measured in the cytosolic fraction did not fit with the annotations in the HPA showing that TP53 is mainly nuclear (S1 Table). Yet, the results showing that TP53 was absent in quiescent PMBC, and upregulated in actively dividing keratinocytes still support the view that the binding was specific.

As explained above, the protocol used for subcellular fractionation seems to cause loss of nuclear proteins to the cytoplasmic fraction (Fig 2). An antibody to the mitotic kinase CDK1 bound two distinct targets (Fig 4). Both were selectively found in dividing cells, and an earlier study has provided evidence that these correspond to the monomeric kinase and the complex with Cyclin B, respectively [8]. The reactivity pattern of the antibody to the regulatory protein CDKN1A (Fig 4) is also in accordance with published results. The protein was not detected in quiescent leukocytes and co-eluted with CDK-cyclin complexes in lysates obtained from proliferating cells [6]. By contrast, CDKN1B is highly expressed in resting leukocytes and down-regulated in proliferating cells.

## Discussion

Obtaining specificity with immobilized antibodies and a protein label is the most important challenge in the development of antibody array analysis. The assay format provides information about the amount of protein bound by the capture antibodies, but there is no intrinsic control of specificity. By implementing sample fractionation, different targets binding to the same antibody can be measured independently [6, 7, 8]. Under these conditions, assay precision is limited only by the resolution of the separation method and the ability to discriminate targets that correspond to specific binding and cross-reactivity, respectively.

In earlier studies where SEC was used to fractionate complex samples, nearly all antibodies showed extensive background binding in the early-eluting fractions [6, 7, 8]. In this study, we combined SEC with differential detergent fractionation and obtained size distribution profiles with far better resolution. The information about the subcellular localization of proteins also simplified the task of discriminating targets corresponding to specific binding and cross-reactivity, respectively.

Among several alternative methods for subcellular fractionation, we chose to rely on differential detergent fractionation (DDF). Compared to the traditional method of homogenizing cells in detergent-free media and separating intact organelles by differential centrifugation, DDF is simple to perform, and the results appear to be more reproducible [11, 12, 13]. To a certain extent, we confirmed earlier results showing that DDF method yields fractions with high purity. With the improvements that were implemented here, there was very little overlap of proteins in the cytoplasm, cytoplasmic organelles and membranes. The similarity in the results obtained with keratinocytes and leukocytes also support the view that DDF has high reproducibility.

The most important limitation of the study was the extensive leakage of nuclear proteins to the cytoplasmic fraction. The nuclear membrane is permeable for proteins smaller than 40kDa, but we found that large nuclear proteins and protein complexes, such as the TP53 tetramer, were lost when cells were permeabilized with digitonin (Fig 2 and Fig 4). This result was surprising, and there appears to be a direct conflict with results obtained earlier by mass spectrometry [22, 23]. There is also strong evidence that the nuclear envelope remains intact after digitonin-treatment [24]. In fact, digitonin-permeabilized cells are used extensively to study active protein transport across the nuclear membrane [24]. In the present study, proteins in mitochondria (CYCS and DIABLO) and the ER (PRKCSH and CALR) were retained during digitonin treatment. Thus, it seems unlikely that the loss of nuclear proteins reflect the use of excessive amounts of the compound. Our result may therefore point to an unexpected difference in the permeability of the membranes surrounding the nucleus and cytoplasmic organelles, respectively. It is also possible that the loss is not related to disruption of membrane structures. In one of the studies that were reviewed in a recent meta-analysis, nuclear proteins were retained even when cells were lysed in 0.5% Triton X-100 [11, 25]. Thus, the present study underscores the need for further studies to establish optimal conditions for separation of proteins from subcellular compartments. The assay format used here combines large-scale detection with high-throughput analysis and is therefore well suited for comparing fractionation protocols.

In spite of the limitations described above, the results obtained here represent a significant step forward in the direction of developing antibody array analysis into a high precision tool in cell biology research and antibody validation. In the supplemental data, we provide reactivity profiles for more than 300 antibodies to commonly studied proteins (S1 Fig). While the presentation is in a graphical format, the results obtained by MAP are numerical. Thus, the datasets can be published in spreadsheet format. With new tools for analysis of results obtained by SEC fractionation, these data can be subjected to meta-analysis. Compared to the current practice of publishing antibody-based results as images, a numerical format provides entirely new possibilities for cross-validation and standardization.

In conclusion, the present study shows that the combination of differential detergent fractionation and SEC enhances the resolution in antibody array analysis and provides a two-dimensional reference for assessment of specificity.

## Supporting information

S1 Fig Validated antibodies. The figure includes the content of the remaining validated antibodies in peripheral blood mononuclear cells (PBMC), confluent human primary keratinocytes (HPK) and non-confluent HPK. The line plots have the same disposition as Figs 1–4. In addition, there is a comment on the reactivity pattern and the validation of each antibody. The results show average of three similar experiments.
(PPT)

S1 Table List of antibody targets. The first table (S1 Fig antibody targets) lists the antibody targets displayed in S1 Fig. The Uniprot Identification number is provided for further reference (Uniprot Id, column A). The antibody targets are displayed in alphabetical order according to their gene name (Gene, column B). Column C indicates antibody targets that are transmembrane or lipid anchored, while column D lists information about the main subcellular location of the targets from the Compartments database. The antibody number on our database (Ab number, column E), immunoglobulin type (Ig type, column F) and the figure number in S1 Fig where the antibody target appears (Fig 1–138, column G) are also listed. Note that several antibodies are listed several times. This is partly because some antibodies detected more than one target and also because several antibodies were immobilized to more than one microsphere subset. The use of the same antibody on different microspheres serves as an internal control of assay precision. The expected result is that these should always cluster as nearest neighbors. The table displays the main subcellular location obtained with MAP (MAP Main, column H) plus additional locations (MAP other, column I). Finally, the table indicates the subcellular location of the antibody targets in the MS datasets used as reference [11] (Dataset 1, 4, 5, 8, 10 and 11, columns J-O). The second table (All Figs antibody targets) lists the antibody targets displayed in all figures. Only antibodies that were validated in the present study are indicated, together with results and references used to assess specificity. The antibody targets are displayed in order of appearance in Figs 1–4, then in alphabetical order as displayed in S1 Fig (Gene, column B). Column C lists information about the main subcellular location of the targets from the Compartments database. The Uniprot Identification number is provided for further reference (Uniprot Id, column A) in addition to the protein size according to Uniprot (mass kDa, column D). The table displays the main subcellular location obtained with MAP (MAP Main, column F) plus additional locations (MAP other, column G), the figure number where the antibody target can be found (Figures, column H) and the line plot within the figure (line plot, column I). Next, the table indicates the subcellular location of the antibody targets in the MS datasets used as reference [11] (Dataset 1, 4, 5, 8, 10 and 11, columns J-O). Finally, the table includes the information on subcellular localization obtained from Uniprot (Uniprot, column P) and GO (G.O., column Q), and information on tissue specificity and protein subunit structure from Uniprot (Tissue specificity (Uniprot), column R and Subunit structure [CC], column S). The third table (Compartments database formatted) lists all the proteins in the Compartments database.
(XLS)

S2 Table Supporting data. The file contains all raw data, displayed in 5 tables according to cell type (keratinocytes, PBMC), cell culture state (confluent, non-confluent) and lysate condition (native, denatured). The tables provide information on validation status of the antibodies (validated, column B). The antibody targets are displayed according to their gene name (Gene, column C), in addition to the protein size (mass kDa, column D). The antibody number in our database is also indicated (antibody nbr, column E). The tables provide further information on the antibodies, such as name, type of immunoglobulin and position in the array (antibody, column F). Finally, raw data is displayed according to subcellular fraction and number of SEC fraction within each subcellular fraction (Dig_01-Dig_24, brij98_01-brij98_24, np40_01-np40_24, DDM_01-DDM_24, columns G-CX).
(XLSX)
