# Supplementary material for: High-resolution antibody array analysis of proteins from primary human keratinocytes and leukocytes
Source: PLoS One. 2018 Dec 27;13(12):e0209271. doi: 10.1371/journal.pone.0209271 (PMC6307719; doi:10.1371/journal.pone.0209271)
Supplement: S1 Fig — The figure includes the content of the remaining validated antibodies in peripheral blood mononuclear cells (PBMC), confluent human primary keratinocytes (HPK) and non-confluent HPK. The line plots have the same disposition as Figs 1–4. In addition, there is a comment on the reactivity pattern and the validation of each antibody. The results show average of three similar experiments. (PPT) [file pone.0209271.s001.ppt]

## Slide 1
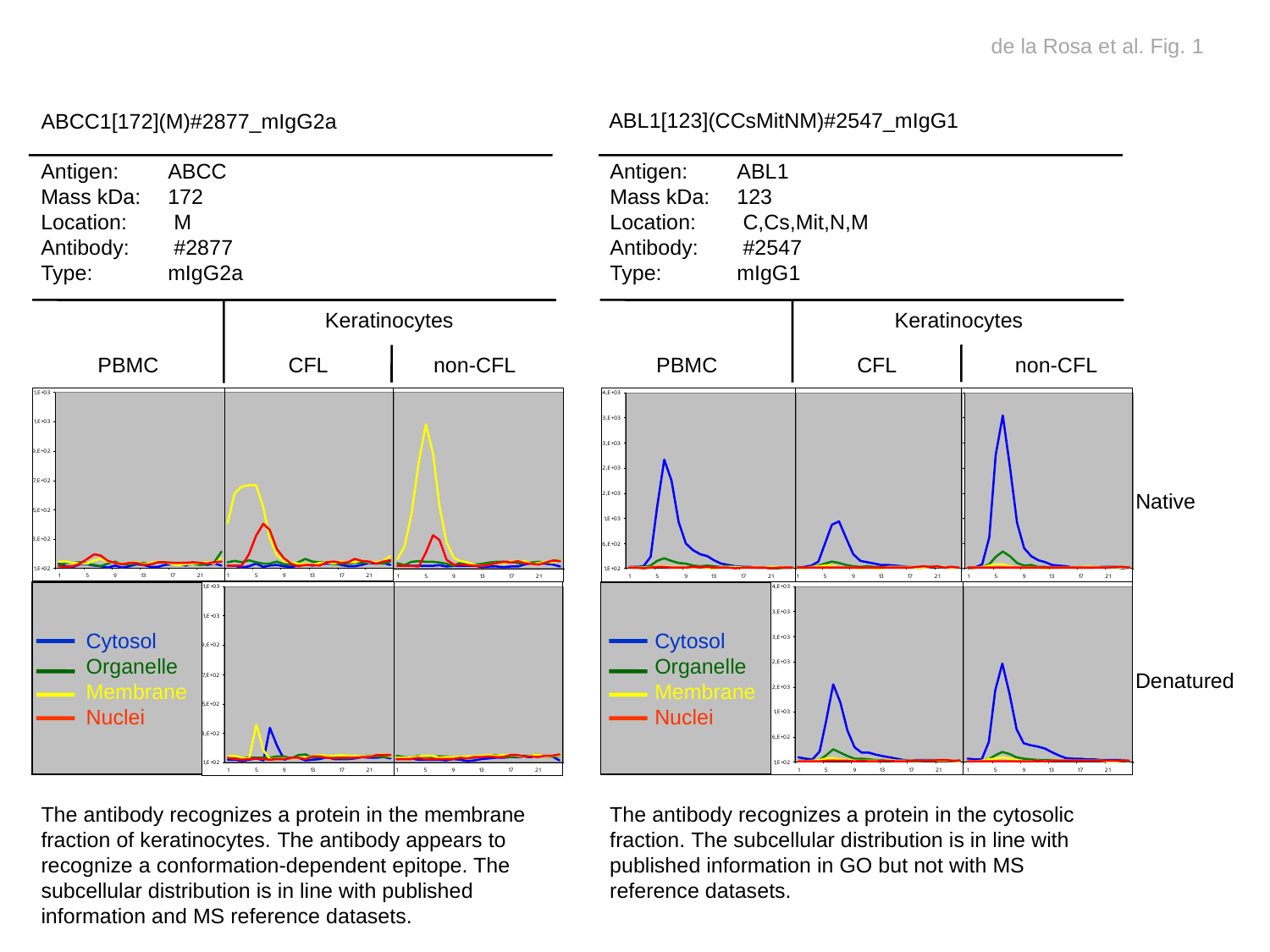

de la Rosa et al. Fig. <number>
ABL1[123](CCsMitNM)#2547_mIgG1
# ABCC1[172](M)#2877_mIgG2a
Antigen: 	ABCC
Mass kDa:	172
Location: 	 M
Antibody: 	 #2877
Type:	mIgG2a
Antigen: 	ABL1
Mass kDa:	123
Location: 	 C,Cs,Mit,N,M
Antibody: 	 #2547
Type:	mIgG1
The antibody recognizes a protein in the membrane fraction of keratinocytes. The antibody appears to recognize a conformation-dependent epitope. The subcellular distribution is in line with published information and MS reference datasets.
The antibody recognizes a protein in the cytosolic fraction. The subcellular distribution is in line with published information in GO but not with MS reference datasets.

## Slide 2
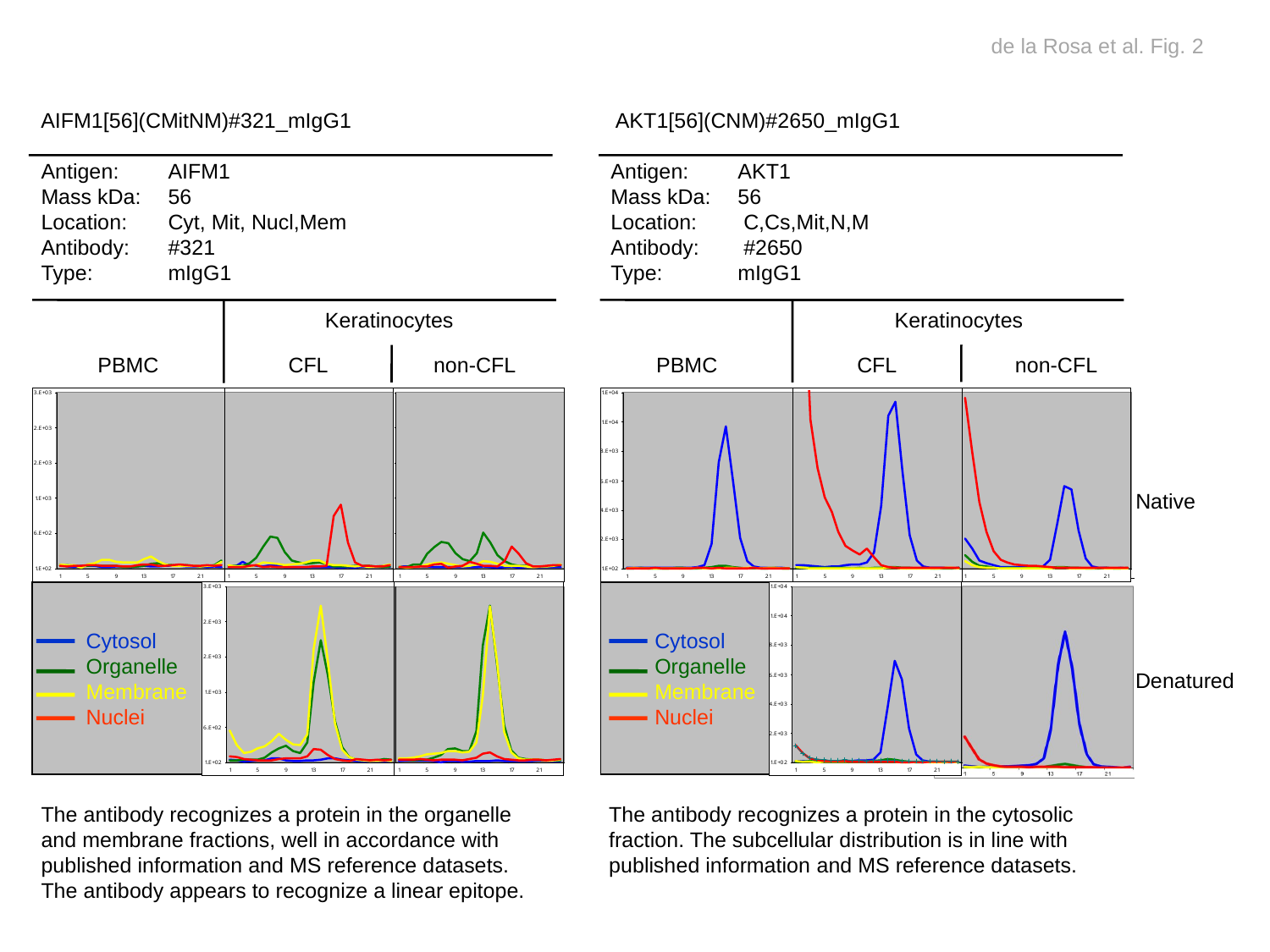

de la Rosa et al. Fig. <number>
AIFM1[56](CMitNM)#321_mIgG1
AKT1[56](CNM)#2650_mIgG1
Antigen: 	AIFM1
Mass kDa:	56
Location: 	Cyt, Mit, Nucl,Mem
Antibody: 	#321
Type:	mIgG1
Antigen: 	AKT1
Mass kDa:	56
Location: 	 C,Cs,Mit,N,M
Antibody: 	 #2650
Type:	mIgG1
The antibody recognizes a protein in the organelle and membrane fractions, well in accordance with published information and MS reference datasets. The antibody appears to recognize a linear epitope.
The antibody recognizes a protein in the cytosolic fraction. The subcellular distribution is in line with published information and MS reference datasets.

## Slide 3
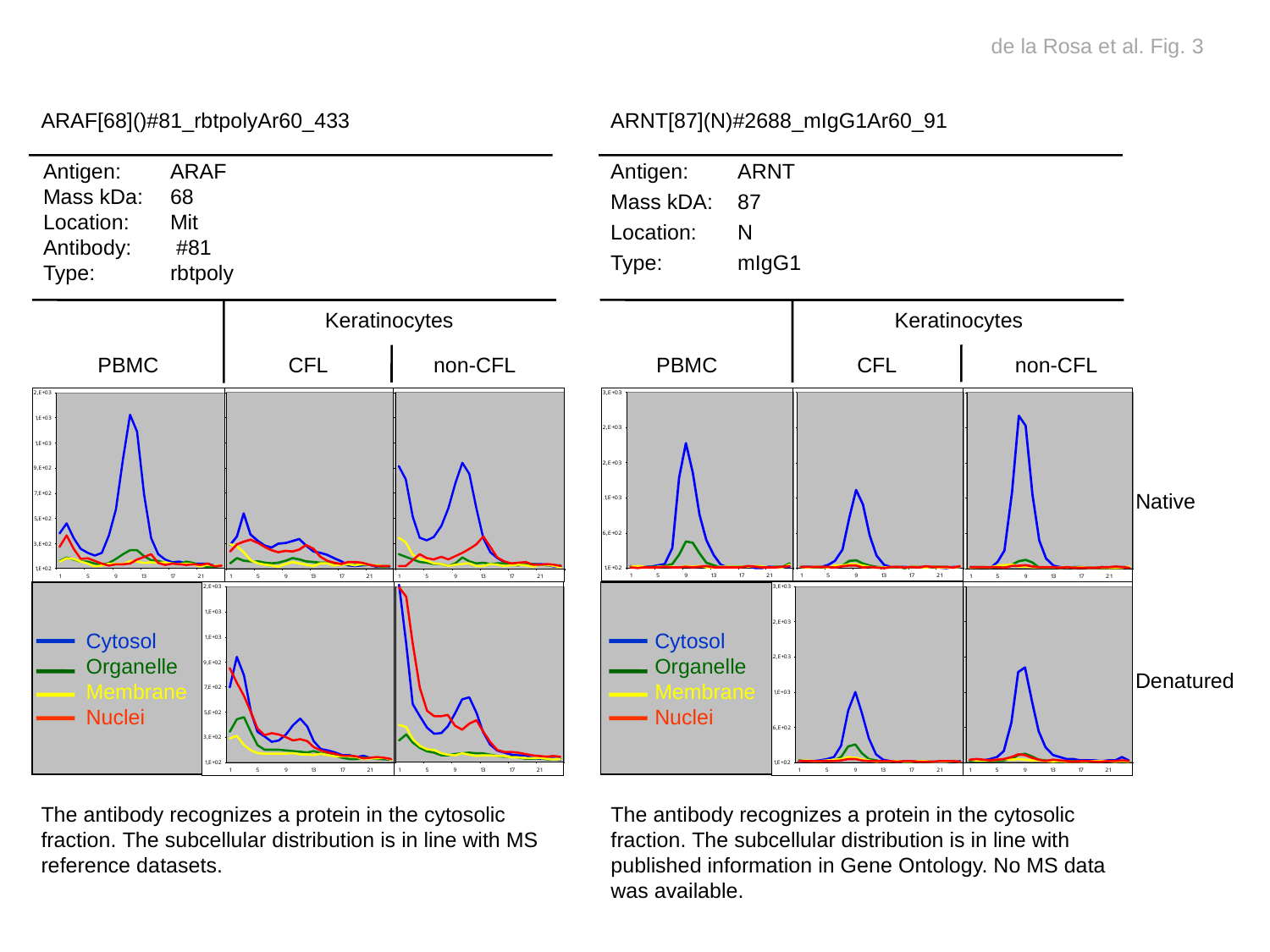

de la Rosa et al. Fig. <number>
# ARAF[68]()#81_rbtpolyAr60_433
ARNT[87](N)#2688_mIgG1Ar60_91
Antigen:	ARNT
Mass kDA:	87
Location:	N
Type:	mIgG1
Antigen: 	ARAF
Mass kDa:	68
Location: 	Mit
Antibody: 	 #81
Type:	rbtpoly
The antibody recognizes a protein in the cytosolic fraction. The subcellular distribution is in line with MS reference datasets.
The antibody recognizes a protein in the cytosolic fraction. The subcellular distribution is in line with published information in Gene Ontology. No MS data was available.

## Slide 4
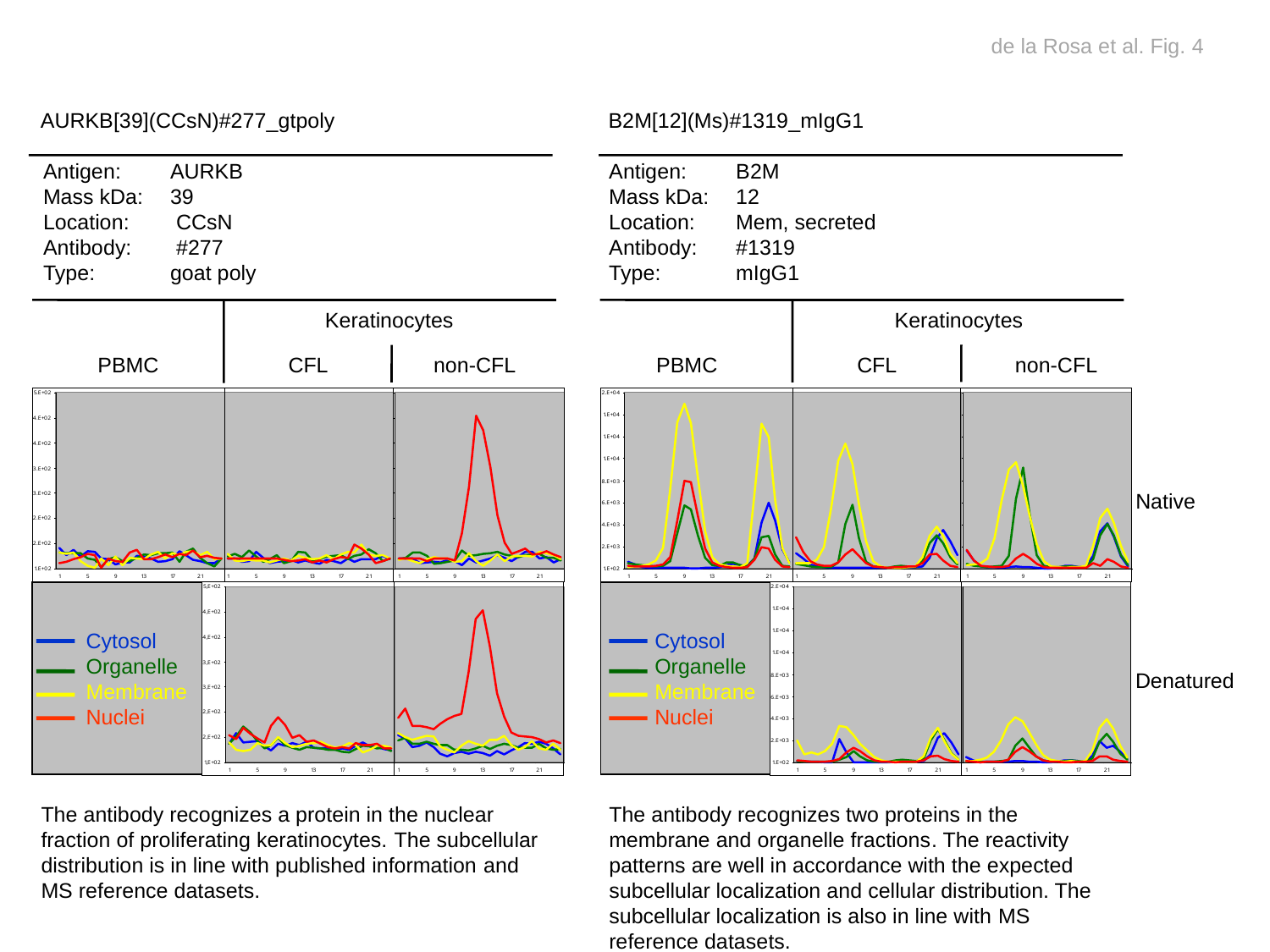

de la Rosa et al. Fig. <number>
AURKB[39](CCsN)#277_gtpoly
B2M[12](Ms)#1319_mIgG1
Antigen: 	AURKB
Mass kDa:	39
Location: 	 CCsN
Antibody: 	 #277
Type:	goat poly
Antigen: 	B2M
Mass kDa:	12
Location: 	Mem, secreted
Antibody: 	#1319
Type:	mIgG1
The antibody recognizes a protein in the nuclear fraction of proliferating keratinocytes. The subcellular distribution is in line with published information and MS reference datasets.
The antibody recognizes two proteins in the membrane and organelle fractions. The reactivity patterns are well in accordance with the expected subcellular localization and cellular distribution. The subcellular localization is also in line with MS reference datasets.

## Slide 5
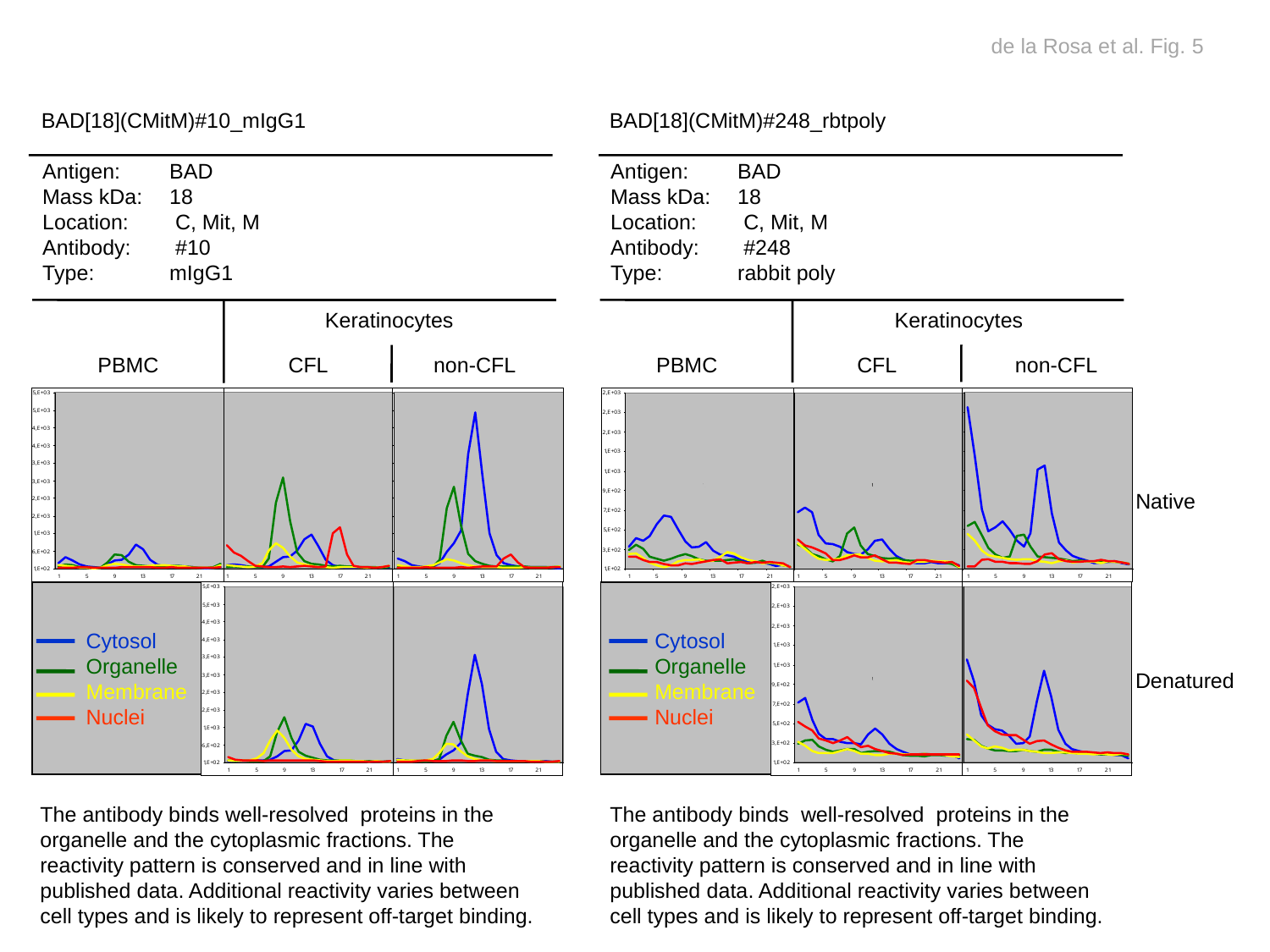

de la Rosa et al. Fig. <number>
BAD[18](CMitM)#10_mIgG1
BAD[18](CMitM)#248_rbtpoly
Antigen: 	BAD
Mass kDa:	18
Location: 	 C, Mit, M
Antibody: 	 #10
Type:	mIgG1
Antigen: 	BAD
Mass kDa:	18
Location: 	 C, Mit, M
Antibody: 	 #248
Type:	rabbit poly
The antibody binds well-resolved proteins in the organelle and the cytoplasmic fractions. The reactivity pattern is conserved and in line with published data. Additional reactivity varies between cell types and is likely to represent off-target binding.
The antibody binds well-resolved proteins in the organelle and the cytoplasmic fractions. The reactivity pattern is conserved and in line with published data. Additional reactivity varies between cell types and is likely to represent off-target binding.

## Slide 6
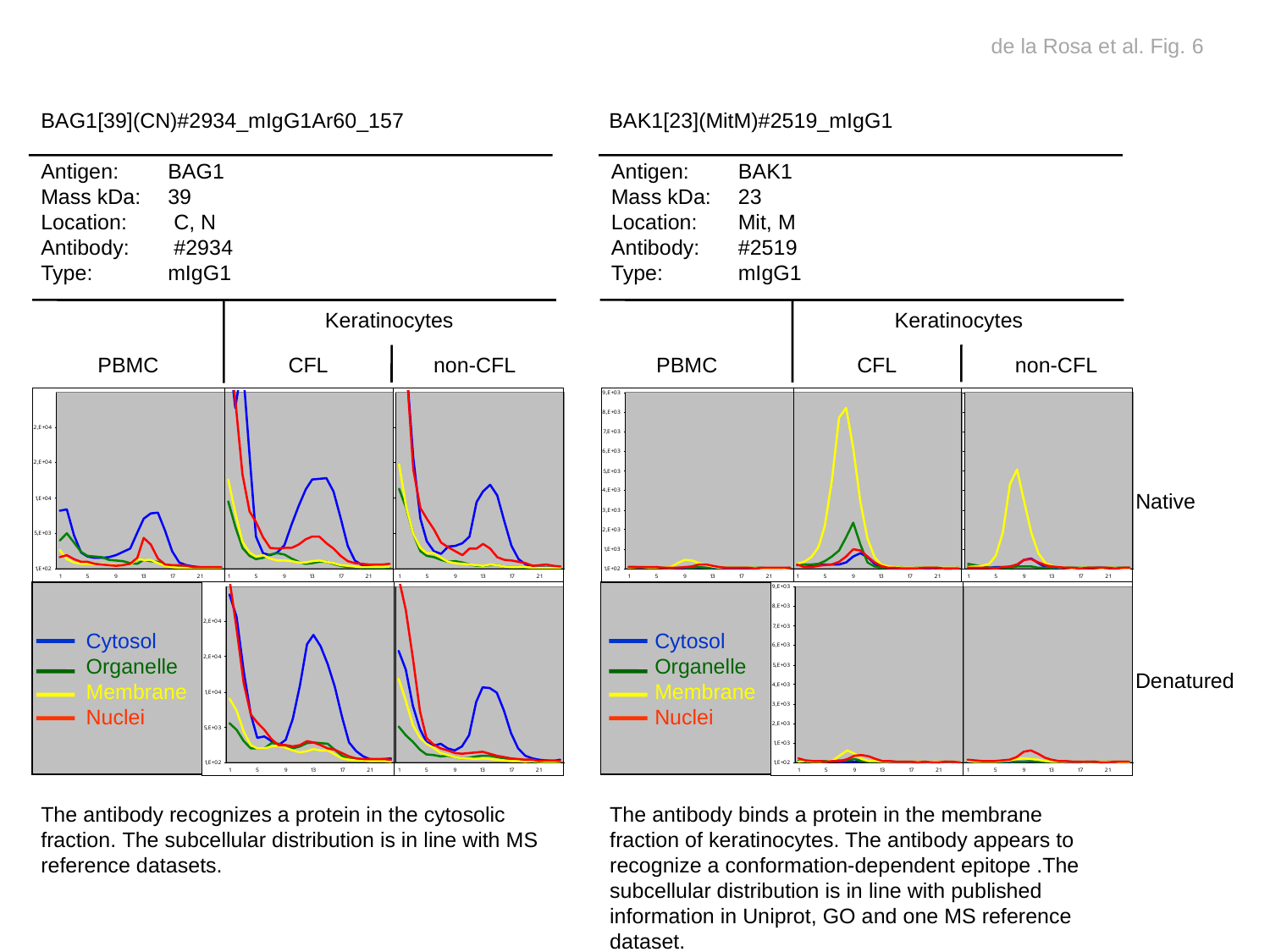

de la Rosa et al. Fig. <number>
BAG1[39](CN)#2934_mIgG1Ar60_157
BAK1[23](MitM)#2519_mIgG1
# Antigen: 	BAG1
Mass kDa:	39
Location: 	 C, N
Antibody: 	 #2934
Type:	mIgG1
Antigen: 	BAK1
Mass kDa:	23
Location: 	Mit, M
Antibody: 	#2519
Type:	mIgG1
The antibody recognizes a protein in the cytosolic fraction. The subcellular distribution is in line with MS reference datasets.
The antibody binds a protein in the membrane
fraction of keratinocytes. The antibody appears to recognize a conformation-dependent epitope .The subcellular distribution is in line with published information in Uniprot, GO and one MS reference dataset.

## Slide 7
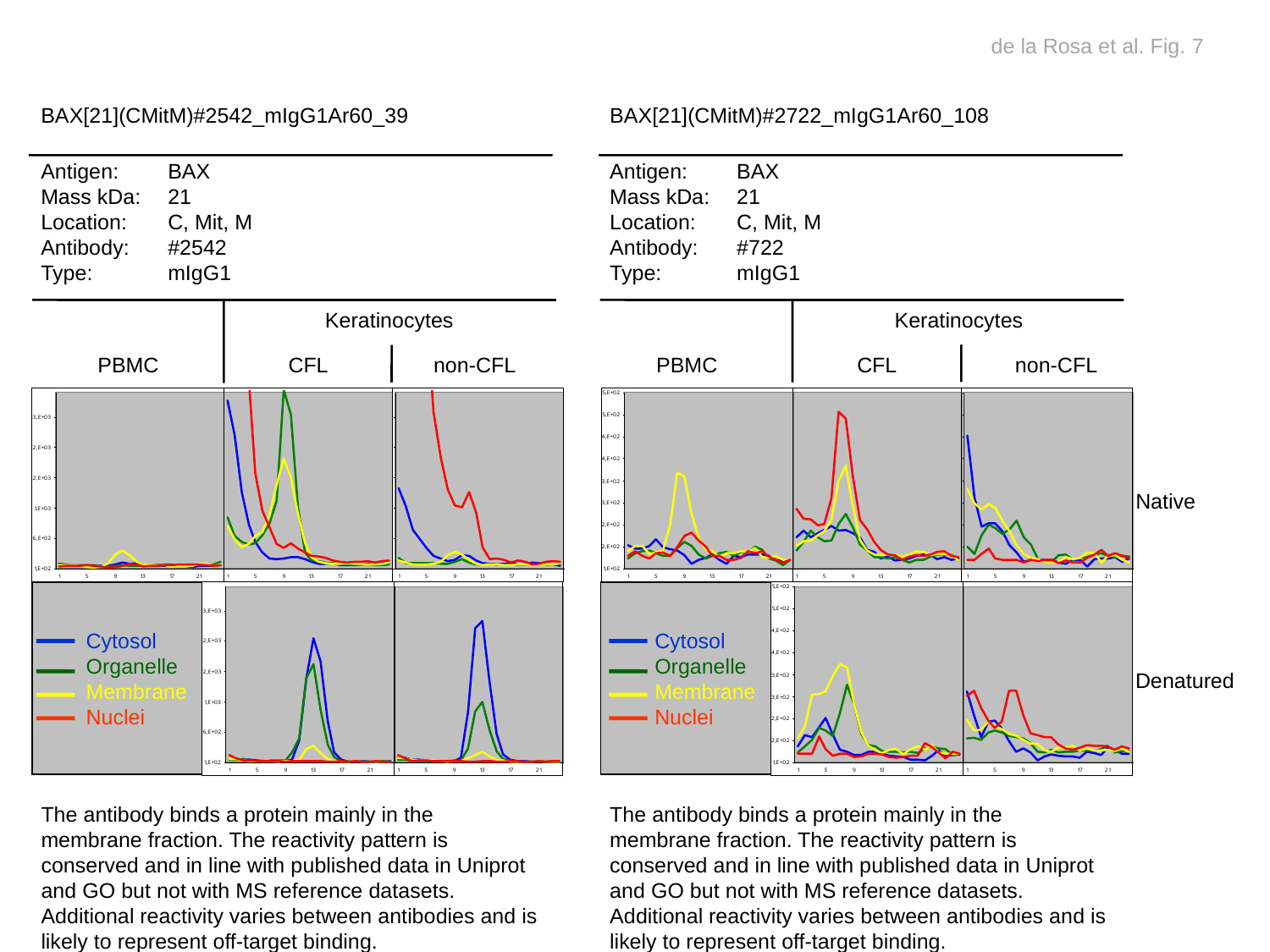

de la Rosa et al. Fig. <number>
# BAX[21](CMitM)#2542_mIgG1Ar60_39
BAX[21](CMitM)#2722_mIgG1Ar60_108
Antigen:	BAX
Mass kDa:	21
Location: 	C, Mit, M
Antibody: 	#2542
Type:	mIgG1
Antigen:	BAX
Mass kDa:	21
Location: 	C, Mit, M
Antibody: 	#722
Type:	mIgG1
The antibody binds a protein mainly in the membrane fraction. The reactivity pattern is conserved and in line with published data in Uniprot and GO but not with MS reference datasets. Additional reactivity varies between antibodies and is likely to represent off-target binding.
The antibody binds a protein mainly in the membrane fraction. The reactivity pattern is conserved and in line with published data in Uniprot and GO but not with MS reference datasets. Additional reactivity varies between antibodies and is likely to represent off-target binding.

## Slide 8
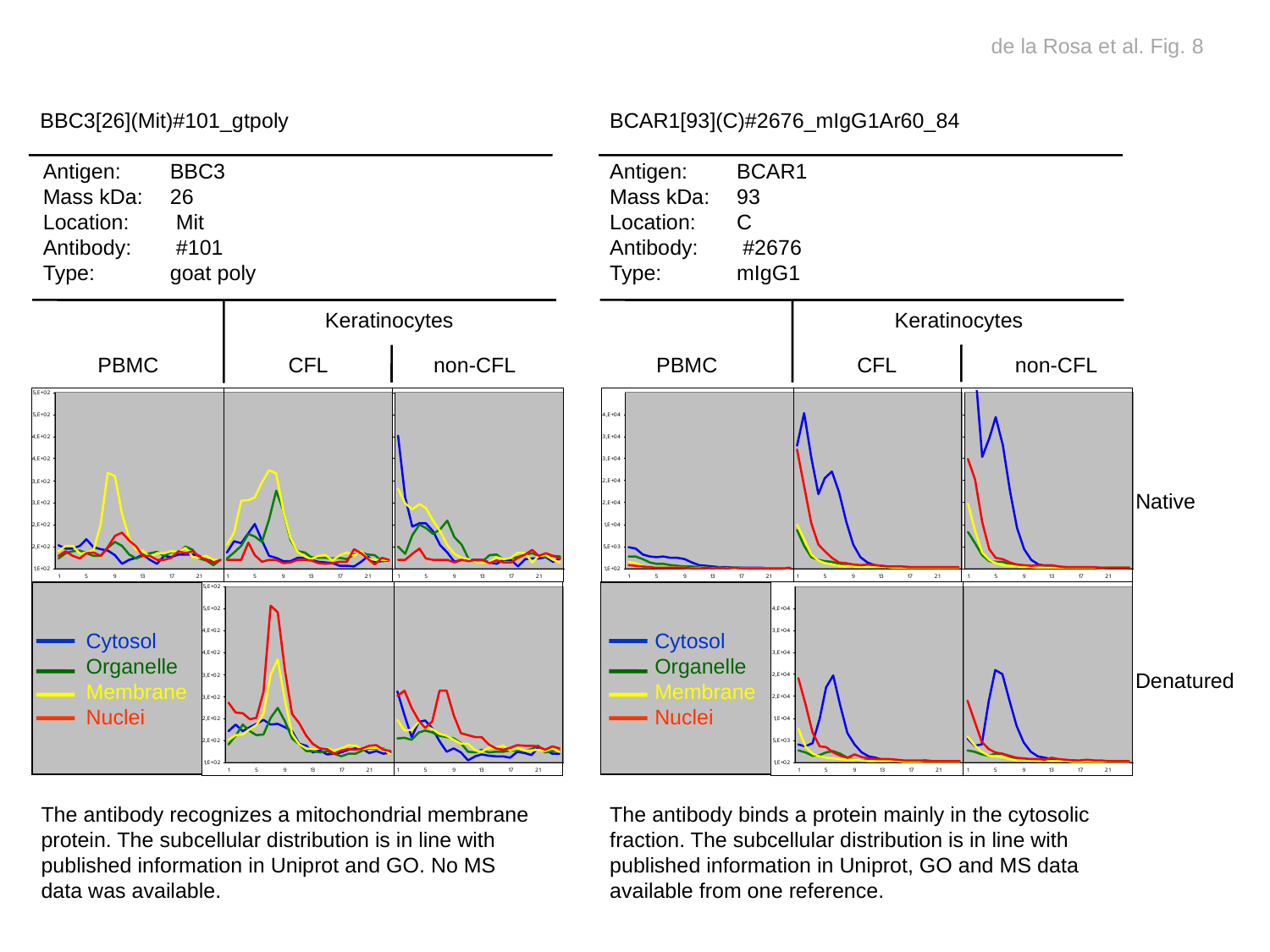

de la Rosa et al. Fig. <number>
BCAR1[93](C)#2676_mIgG1Ar60_84
BBC3[26](Mit)#101_gtpoly
# Antigen: 	BCAR1
Mass kDa:	93
Location: 	C
Antibody: 	 #2676
Type:	mIgG1
Antigen: 	BBC3
Mass kDa:	26
Location: 	 Mit
Antibody: 	 #101
Type:	goat poly
The antibody recognizes a mitochondrial membrane protein. The subcellular distribution is in line with published information in Uniprot and GO. No MS data was available.
The antibody binds a protein mainly in the cytosolic fraction. The subcellular distribution is in line with published information in Uniprot, GO and MS data available from one reference.

## Slide 9
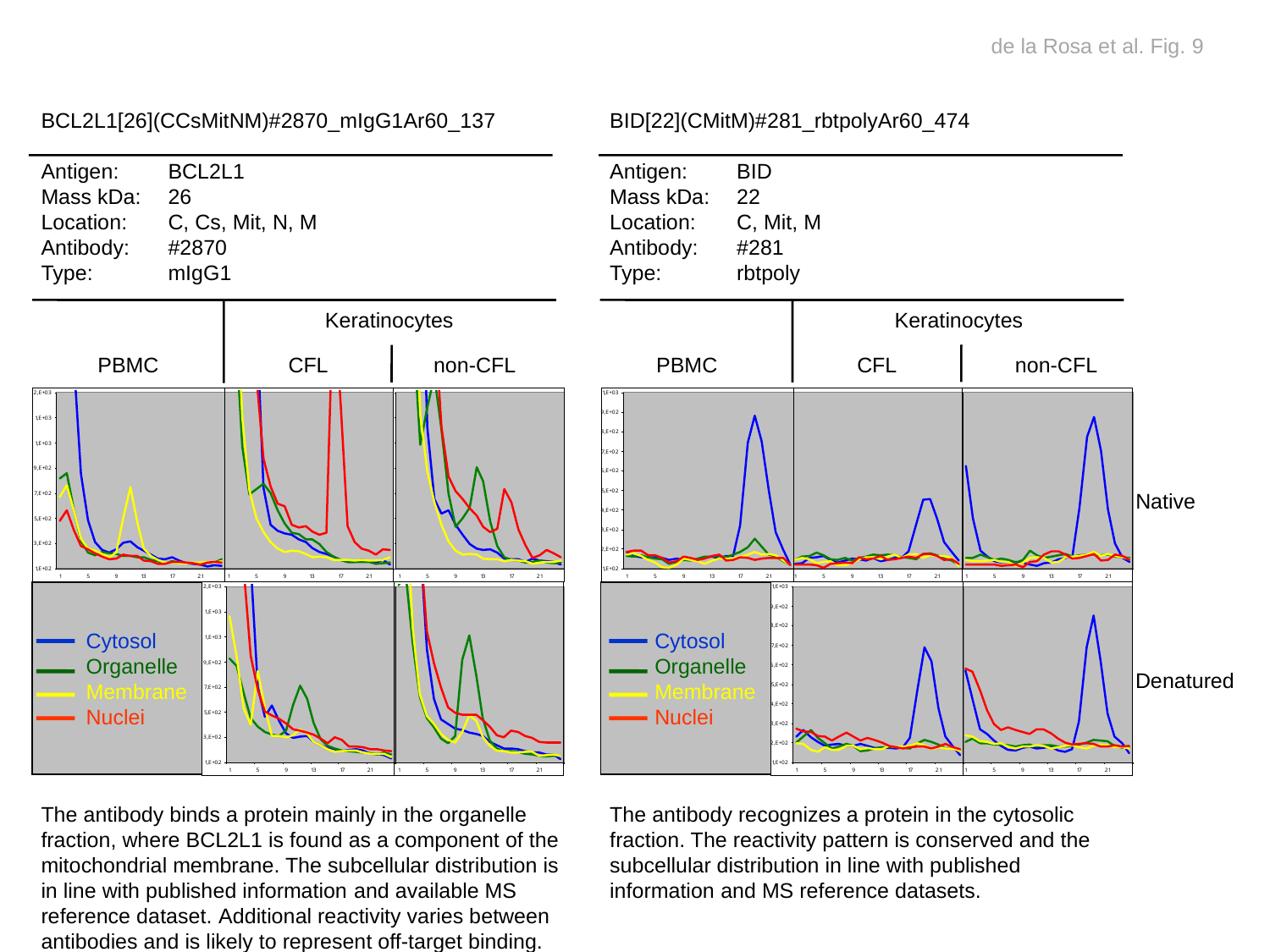

de la Rosa et al. Fig. <number>
# BCL2L1[26](CCsMitNM)#2870_mIgG1Ar60_137
BID[22](CMitM)#281_rbtpolyAr60_474
Antigen: 	BCL2L1
Mass kDa:	26
Location: 	C, Cs, Mit, N, M
Antibody: 	#2870
Type:	mIgG1
Antigen: 	BID
Mass kDa:	22
Location: 	C, Mit, M
Antibody: 	#281
Type:	rbtpoly
The antibody binds a protein mainly in the organelle fraction, where BCL2L1 is found as a component of the mitochondrial membrane. The subcellular distribution is in line with published information and available MS reference dataset. Additional reactivity varies between antibodies and is likely to represent off-target binding.
The antibody recognizes a protein in the cytosolic fraction. The reactivity pattern is conserved and the subcellular distribution in line with published information and MS reference datasets.

## Slide 10
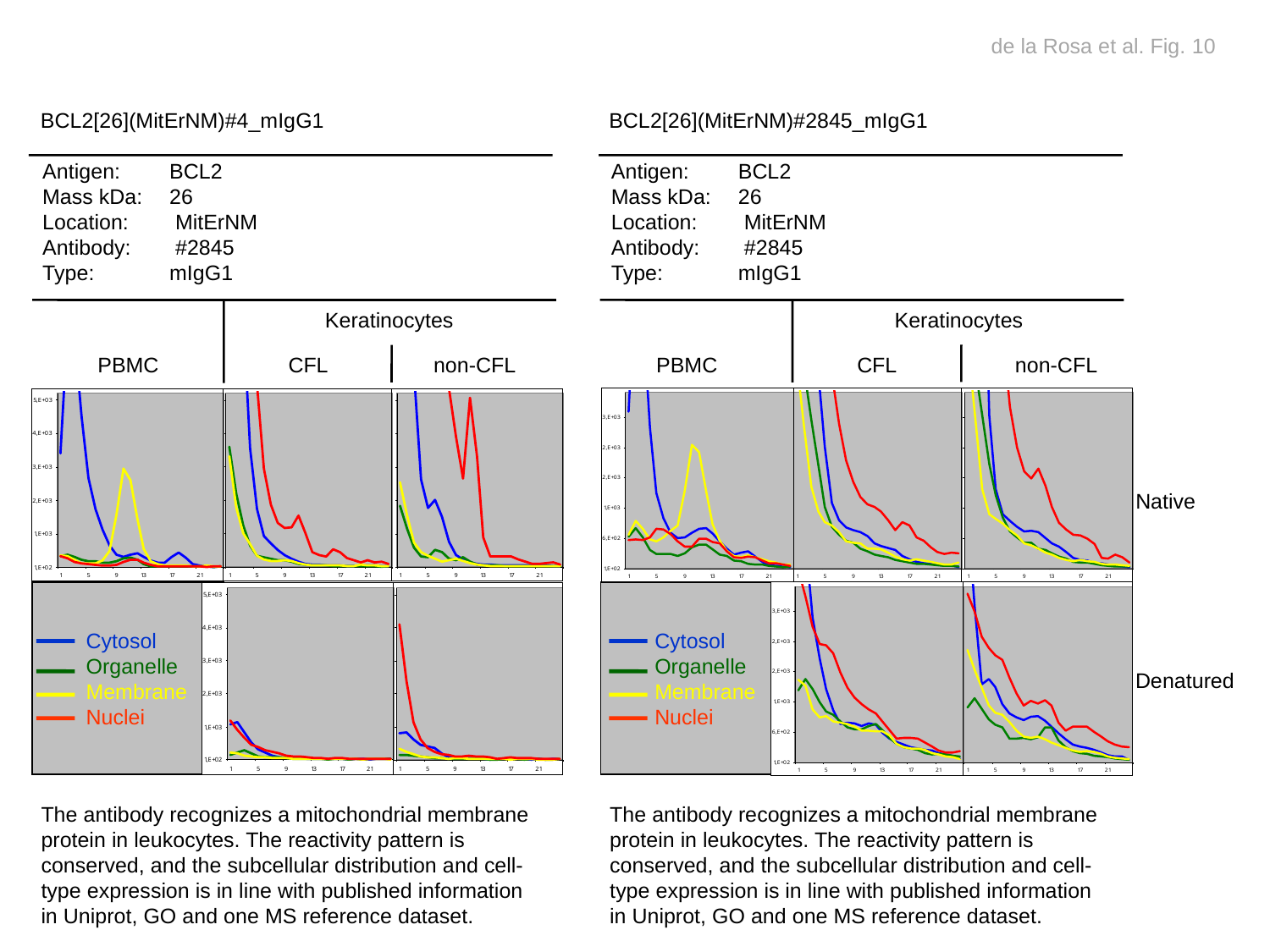

de la Rosa et al. Fig. <number>
BCL2[26](MitErNM)#4_mIgG1
BCL2[26](MitErNM)#2845_mIgG1
Antigen: 	BCL2
Mass kDa:	26
Location: 	 MitErNM
Antibody: 	 #2845
Type:	mIgG1
Antigen: 	BCL2
Mass kDa:	26
Location: 	 MitErNM
Antibody: 	 #2845
Type:	mIgG1
The antibody recognizes a mitochondrial membrane protein in leukocytes. The reactivity pattern is conserved, and the subcellular distribution and cell-type expression is in line with published information in Uniprot, GO and one MS reference dataset.
The antibody recognizes a mitochondrial membrane protein in leukocytes. The reactivity pattern is conserved, and the subcellular distribution and cell-type expression is in line with published information in Uniprot, GO and one MS reference dataset.

## Slide 11
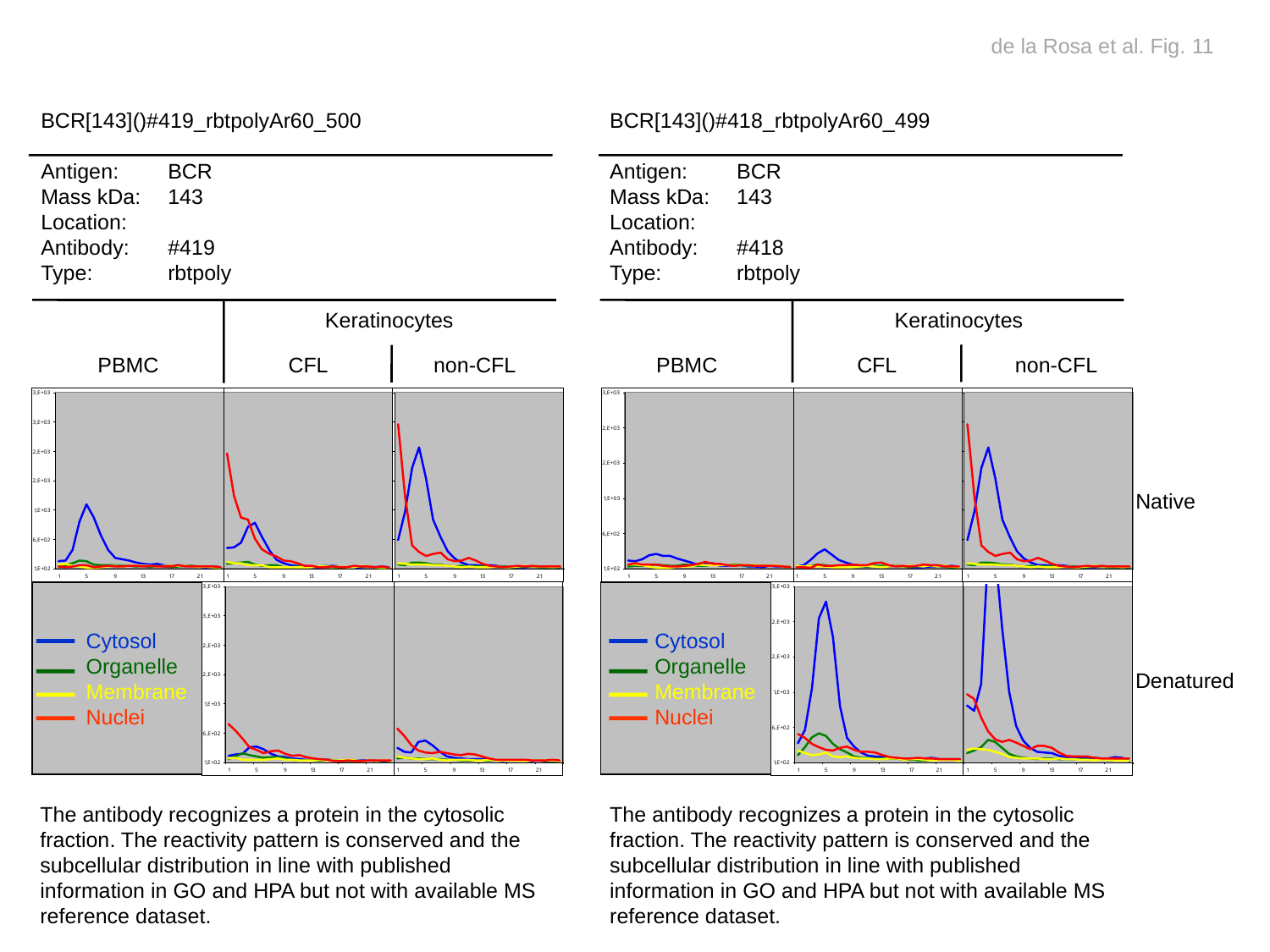

de la Rosa et al. Fig. <number>
BCR[143]()#419_rbtpolyAr60_500
# BCR[143]()#418_rbtpolyAr60_499
Antigen: 	BCR
Mass kDa:	143
Location:
Antibody: 	#419
Type:	rbtpoly
Antigen: 	BCR
Mass kDa:	143
Location:
Antibody: 	#418
Type:	rbtpoly
The antibody recognizes a protein in the cytosolic fraction. The reactivity pattern is conserved and the subcellular distribution in line with published information in GO and HPA but not with available MS reference dataset.
The antibody recognizes a protein in the cytosolic fraction. The reactivity pattern is conserved and the subcellular distribution in line with published information in GO and HPA but not with available MS reference dataset.

## Slide 12
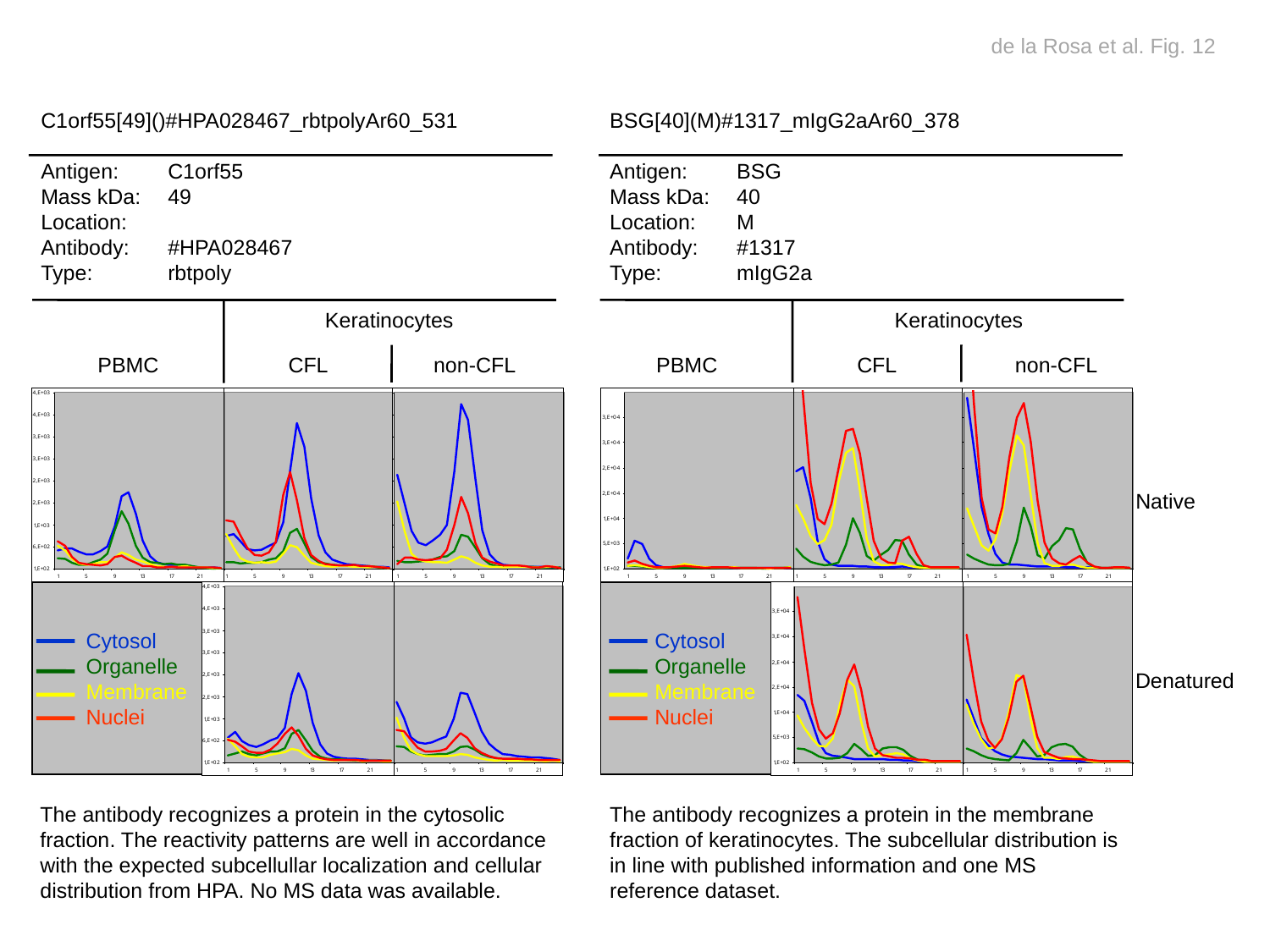

de la Rosa et al. Fig. <number>
C1orf55[49]()#HPA028467_rbtpolyAr60_531
# BSG[40](M)#1317_mIgG2aAr60_378
Antigen: 	C1orf55
Mass kDa:	49
Location:
Antibody: 	#HPA028467
Type:	rbtpoly
Antigen: 	BSG
Mass kDa:	40
Location: 	M
Antibody: 	#1317
Type:	mIgG2a
The antibody recognizes a protein in the cytosolic fraction. The reactivity patterns are well in accordance with the expected subcellullar localization and cellular distribution from HPA. No MS data was available.
The antibody recognizes a protein in the membrane fraction of keratinocytes. The subcellular distribution is in line with published information and one MS reference dataset.

## Slide 13
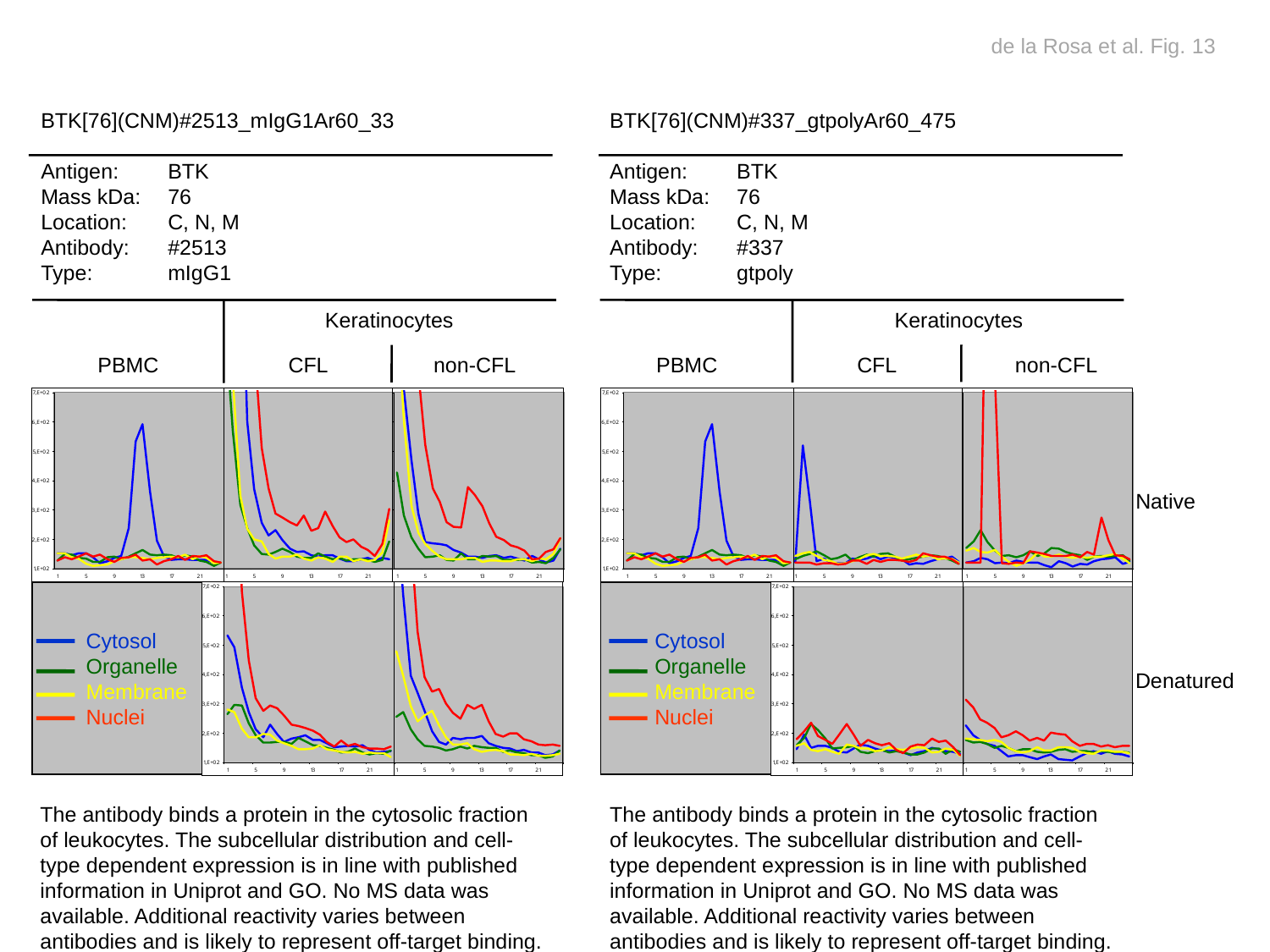

de la Rosa et al. Fig. <number>
# BTK[76](CNM)#2513_mIgG1Ar60_33
BTK[76](CNM)#337_gtpolyAr60_475
Antigen: 	BTK
Mass kDa:	76
Location: 	C, N, M
Antibody: 	#2513
Type:	mIgG1
Antigen: 	BTK
Mass kDa:	76
Location: 	C, N, M
Antibody: 	#337
Type:	gtpoly
The antibody binds a protein in the cytosolic fraction of leukocytes. The subcellular distribution and cell-type dependent expression is in line with published information in Uniprot and GO. No MS data was available. Additional reactivity varies between antibodies and is likely to represent off-target binding.
The antibody binds a protein in the cytosolic fraction of leukocytes. The subcellular distribution and cell-type dependent expression is in line with published information in Uniprot and GO. No MS data was available. Additional reactivity varies between antibodies and is likely to represent off-target binding.

## Slide 14
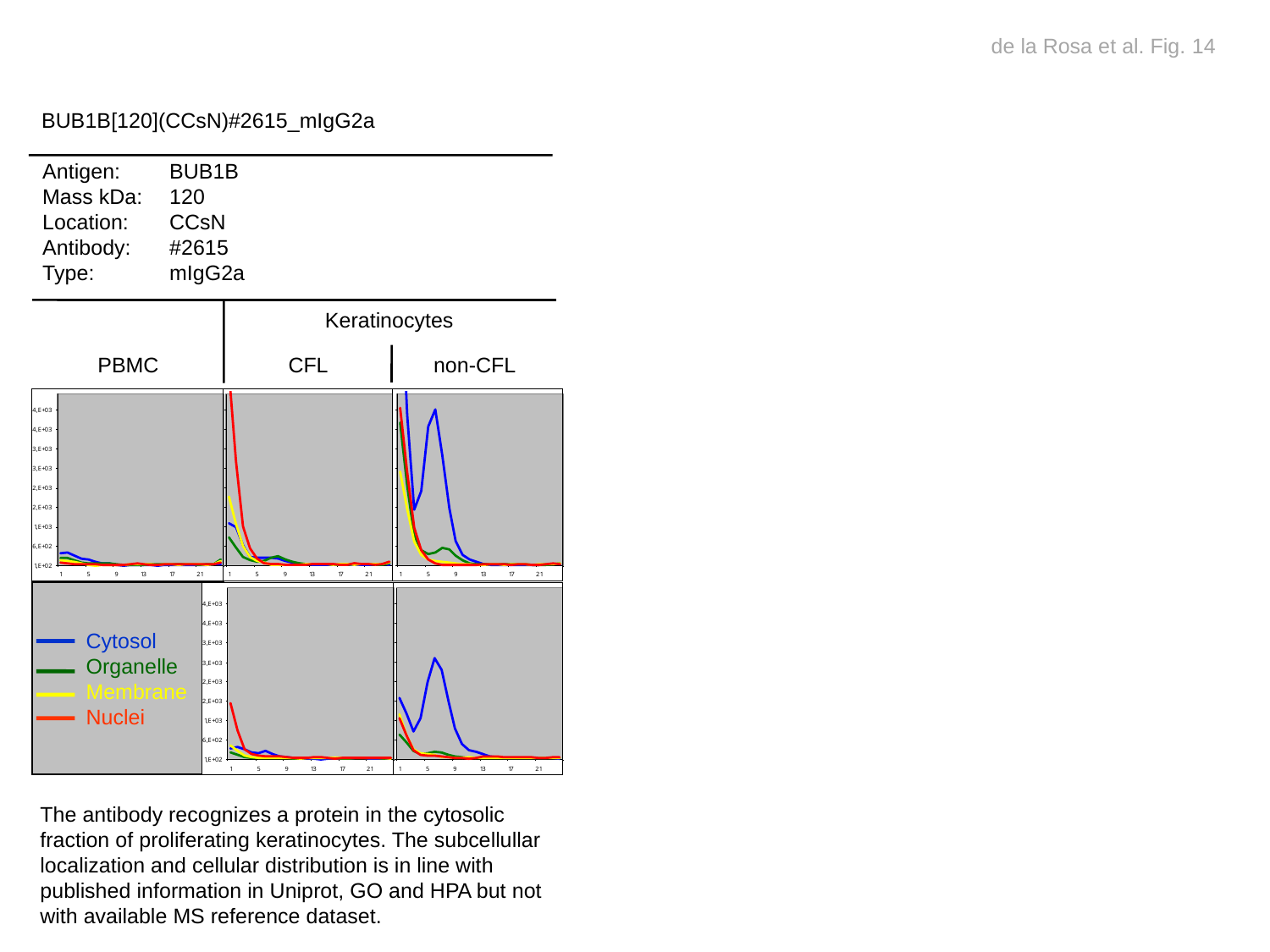

de la Rosa et al. Fig. <number>
BUB1B[120](CCsN)#2615_mIgG2a
Antigen: 	BUB1B
Mass kDa:	120
Location: 	CCsN
Antibody: 	#2615
Type:	mIgG2a
The antibody recognizes a protein in the cytosolic fraction of proliferating keratinocytes. The subcellullar localization and cellular distribution is in line with published information in Uniprot, GO and HPA but not with available MS reference dataset.

## Slide 15
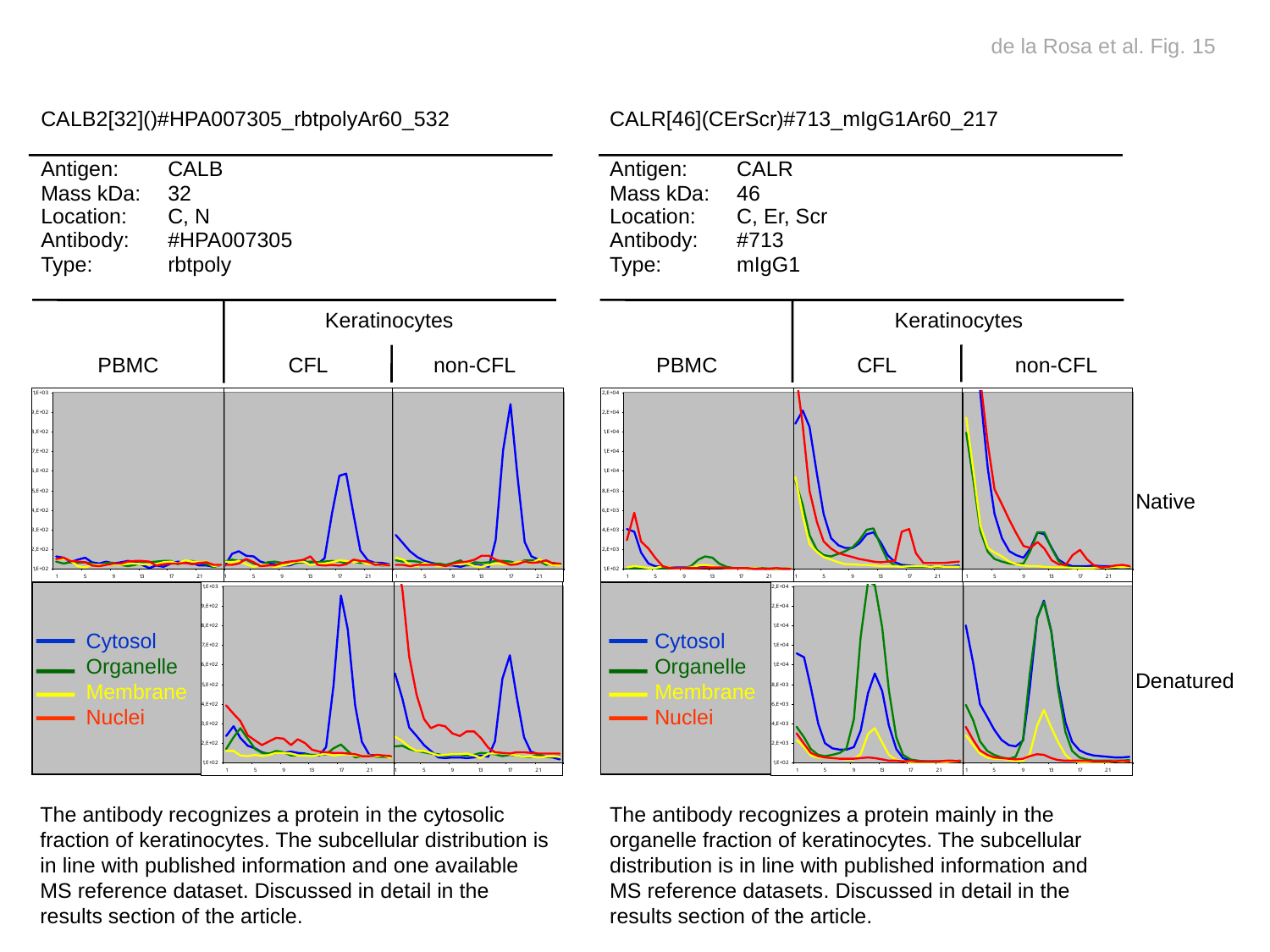

de la Rosa et al. Fig. <number>
# CALB2[32]()#HPA007305_rbtpolyAr60_532
CALR[46](CErScr)#713_mIgG1Ar60_217
Antigen: 	CALB
Mass kDa:	32
Location: 	C, N
Antibody: 	#HPA007305
Type:	rbtpoly
Antigen: 	CALR
Mass kDa:	46
Location: 	C, Er, Scr
Antibody: 	#713
Type:	mIgG1
The antibody recognizes a protein in the cytosolic fraction of keratinocytes. The subcellular distribution is in line with published information and one available MS reference dataset. Discussed in detail in the results section of the article.
The antibody recognizes a protein mainly in the organelle fraction of keratinocytes. The subcellular distribution is in line with published information and MS reference datasets. Discussed in detail in the results section of the article.

## Slide 16
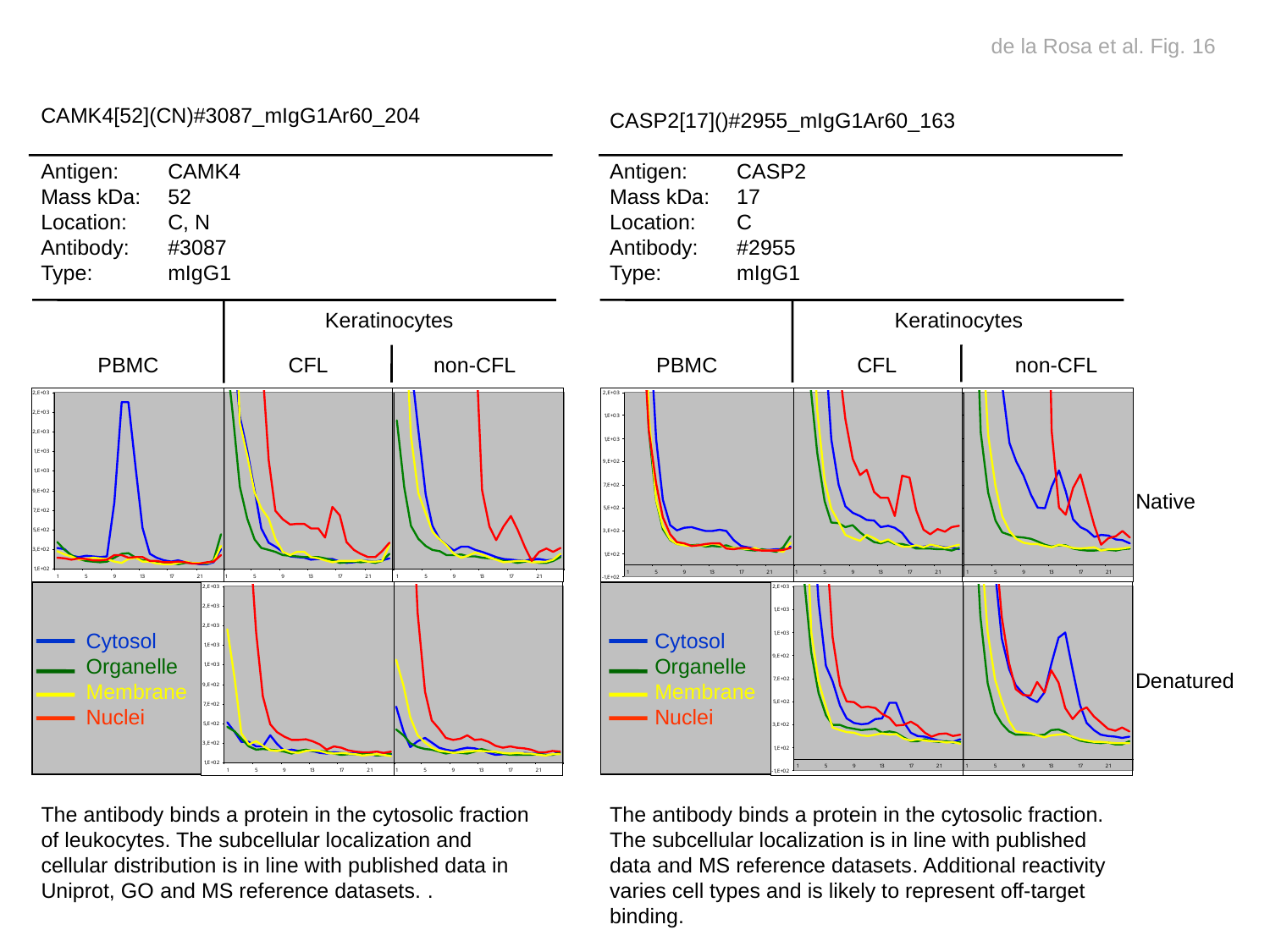

de la Rosa et al. Fig. <number>
# CAMK4[52](CN)#3087_mIgG1Ar60_204
CASP2[17]()#2955_mIgG1Ar60_163
Antigen: 	CAMK4
Mass kDa:	52
Location: 	C, N
Antibody: 	#3087
Type:	mIgG1
Antigen: 	CASP2
Mass kDa:	17
Location: 	C
Antibody: 	#2955
Type:	mIgG1
The antibody binds a protein in the cytosolic fraction of leukocytes. The subcellular localization and cellular distribution is in line with published data in Uniprot, GO and MS reference datasets. .
The antibody binds a protein in the cytosolic fraction. The subcellular localization is in line with published data and MS reference datasets. Additional reactivity varies cell types and is likely to represent off-target binding.

## Slide 17
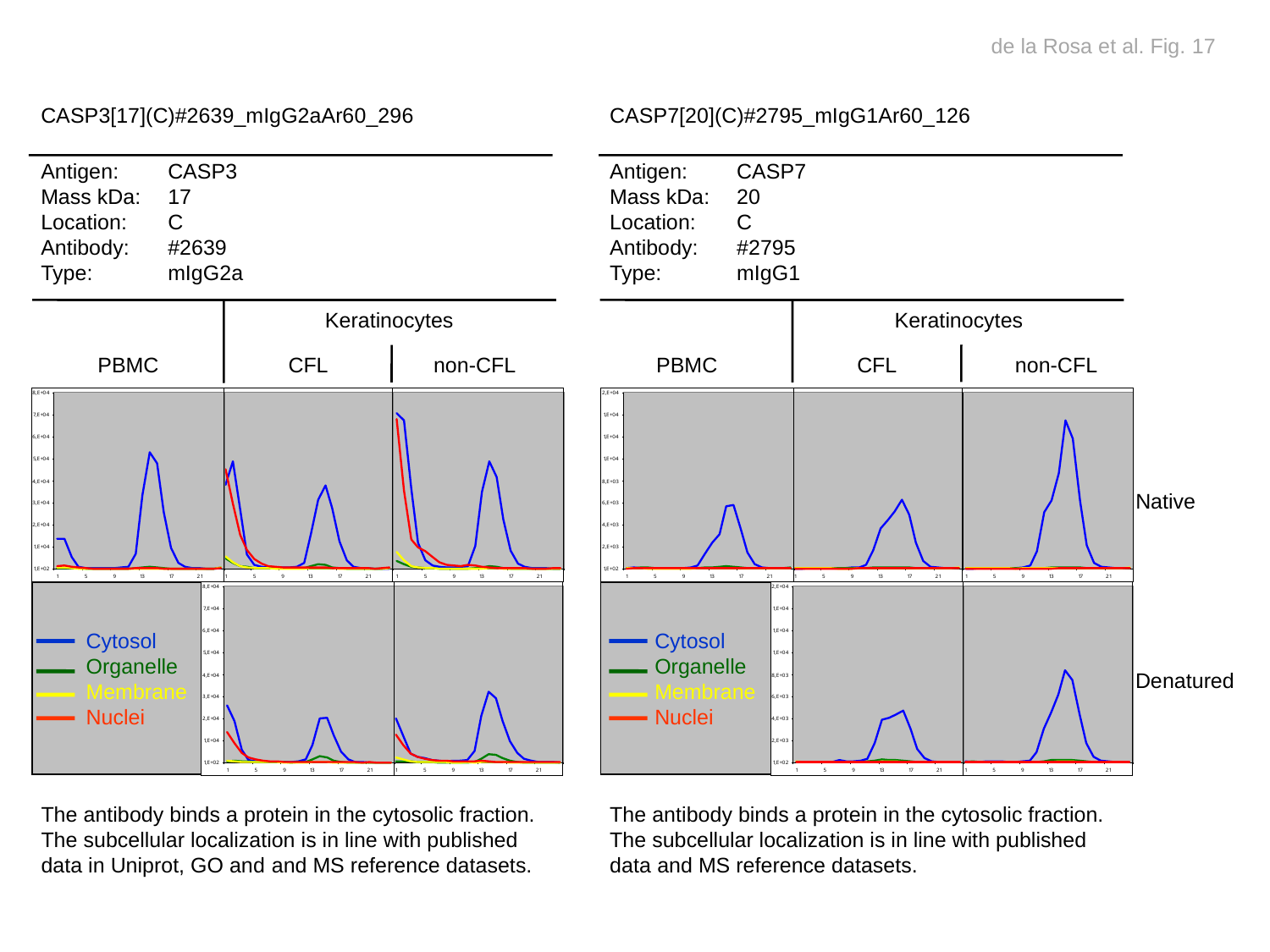

de la Rosa et al. Fig. <number>
# CASP3[17](C)#2639_mIgG2aAr60_296
CASP7[20](C)#2795_mIgG1Ar60_126
Antigen: 	CASP3
Mass kDa:	17
Location: 	C
Antibody: 	#2639
Type:	mIgG2a
Antigen: 	CASP7
Mass kDa:	20
Location: 	C
Antibody: 	#2795
Type:	mIgG1
The antibody binds a protein in the cytosolic fraction. The subcellular localization is in line with published data in Uniprot, GO and and MS reference datasets.
The antibody binds a protein in the cytosolic fraction. The subcellular localization is in line with published data and MS reference datasets.

## Slide 18
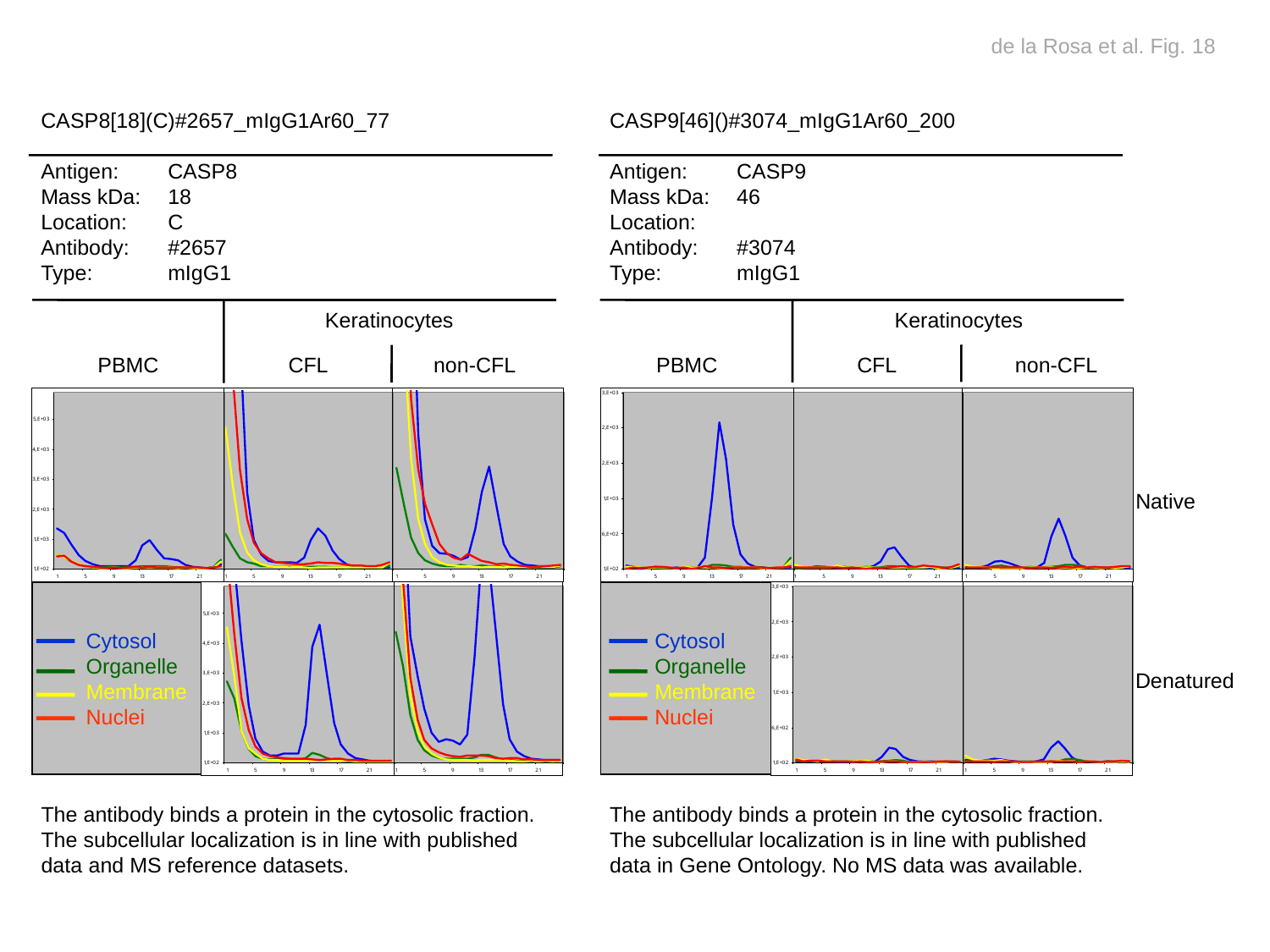

de la Rosa et al. Fig. <number>
# CASP8[18](C)#2657_mIgG1Ar60_77
CASP9[46]()#3074_mIgG1Ar60_200
Antigen: 	CASP8
Mass kDa:	18
Location: 	C
Antibody: 	#2657
Type:	mIgG1
Antigen: 	CASP9
Mass kDa:	46
Location:
Antibody: 	#3074
Type:	mIgG1
The antibody binds a protein in the cytosolic fraction. The subcellular localization is in line with published data and MS reference datasets.
The antibody binds a protein in the cytosolic fraction. The subcellular localization is in line with published data in Gene Ontology. No MS data was available.

## Slide 19
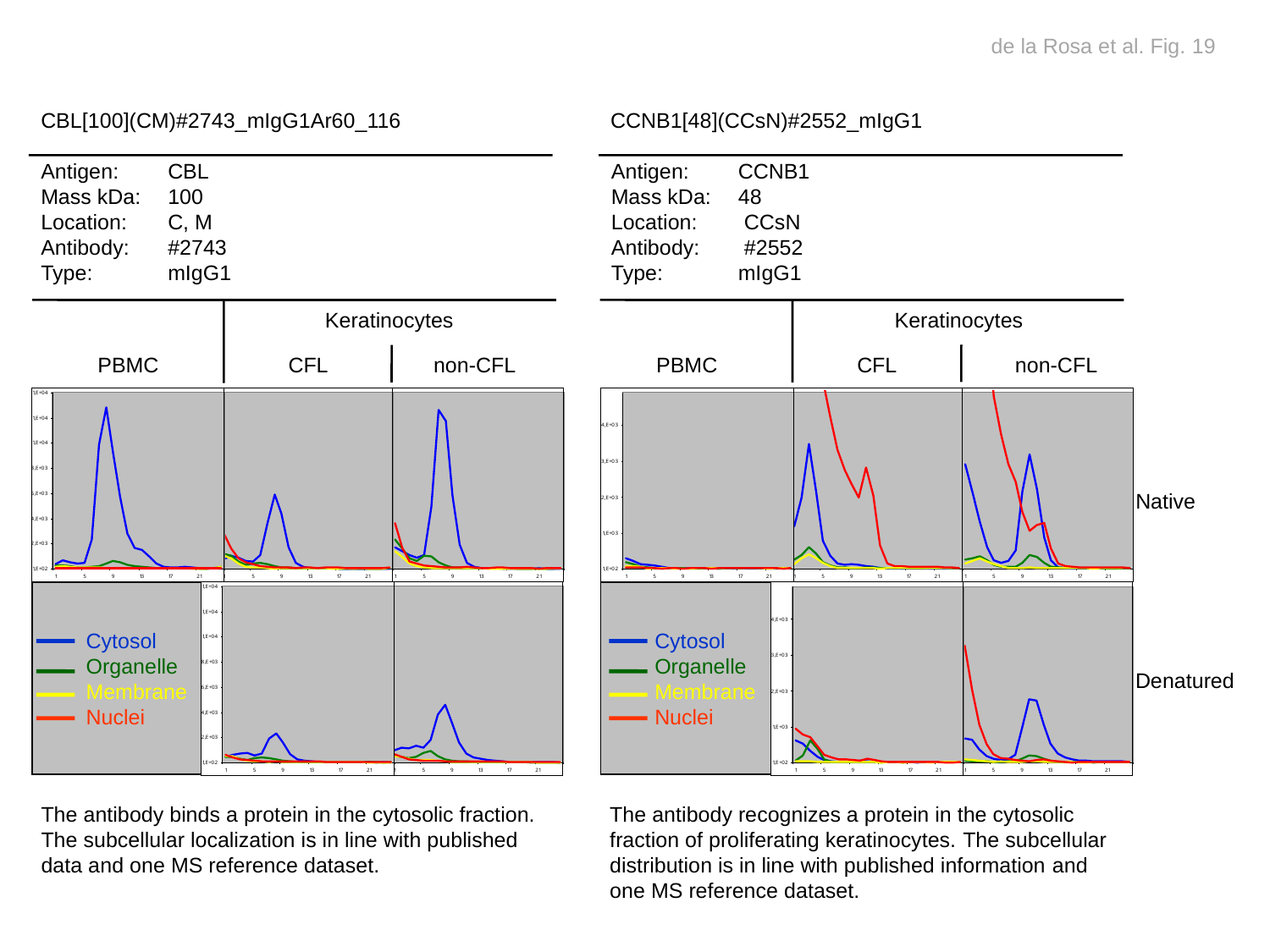

de la Rosa et al. Fig. <number>
# CBL[100](CM)#2743_mIgG1Ar60_116
CCNB1[48](CCsN)#2552_mIgG1
Antigen: 	CBL
Mass kDa:	100
Location: 	C, M
Antibody: 	#2743
Type:	mIgG1
Antigen: 	CCNB1
Mass kDa:	48
Location: 	 CCsN
Antibody: 	 #2552
Type:	mIgG1
The antibody binds a protein in the cytosolic fraction. The subcellular localization is in line with published data and one MS reference dataset.
The antibody recognizes a protein in the cytosolic fraction of proliferating keratinocytes. The subcellular distribution is in line with published information and one MS reference dataset.

## Slide 20
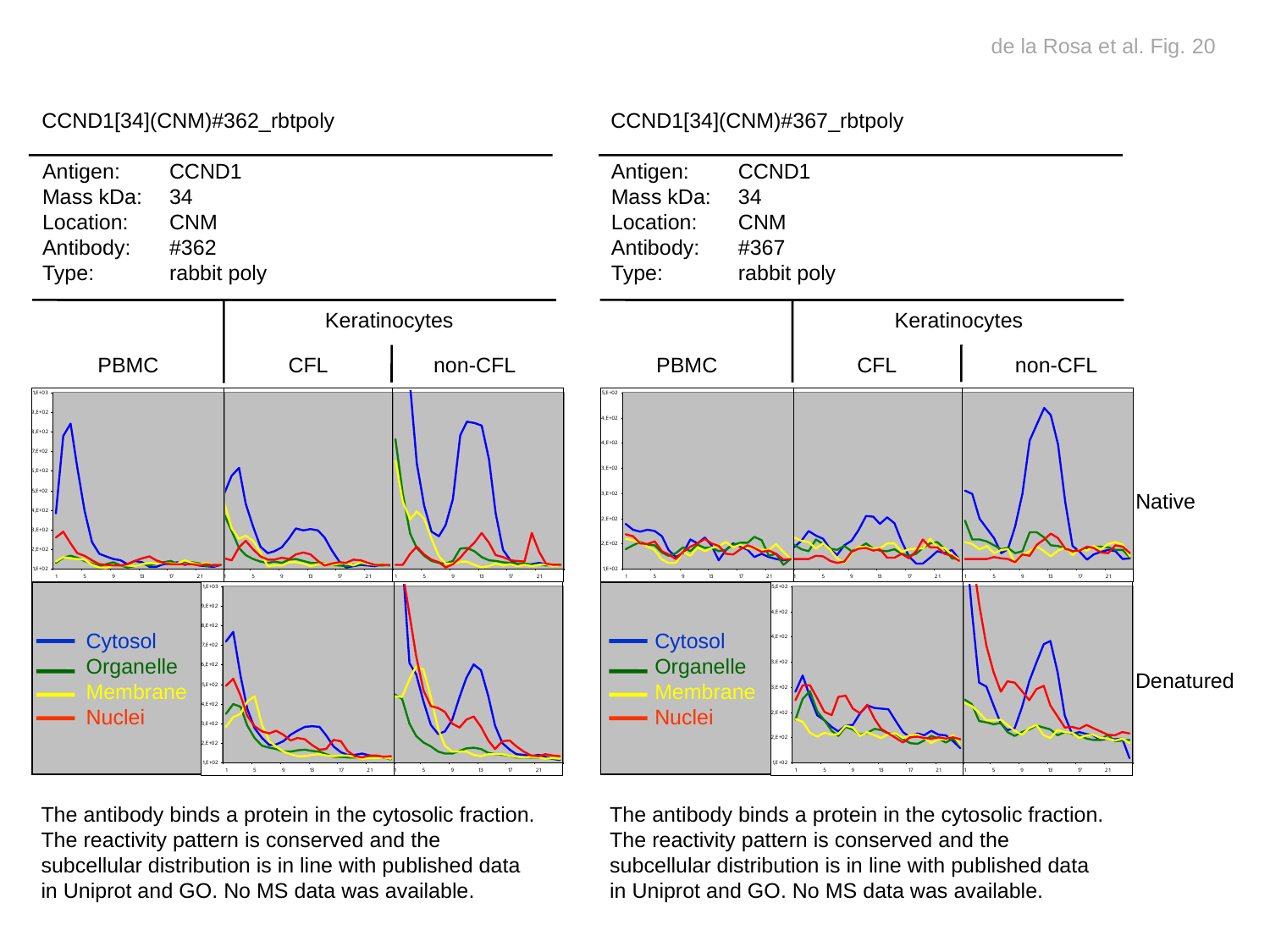

de la Rosa et al. Fig. <number>
CCND1[34](CNM)#362_rbtpoly
CCND1[34](CNM)#367_rbtpoly
Antigen: 	CCND1
Mass kDa:	34
Location: 	CNM
Antibody: 	#362
Type:	rabbit poly
Antigen: 	CCND1
Mass kDa:	34
Location: 	CNM
Antibody: 	#367
Type:	rabbit poly
The antibody binds a protein in the cytosolic fraction. The reactivity pattern is conserved and the subcellular distribution is in line with published data in Uniprot and GO. No MS data was available.
The antibody binds a protein in the cytosolic fraction. The reactivity pattern is conserved and the subcellular distribution is in line with published data in Uniprot and GO. No MS data was available.

## Slide 21
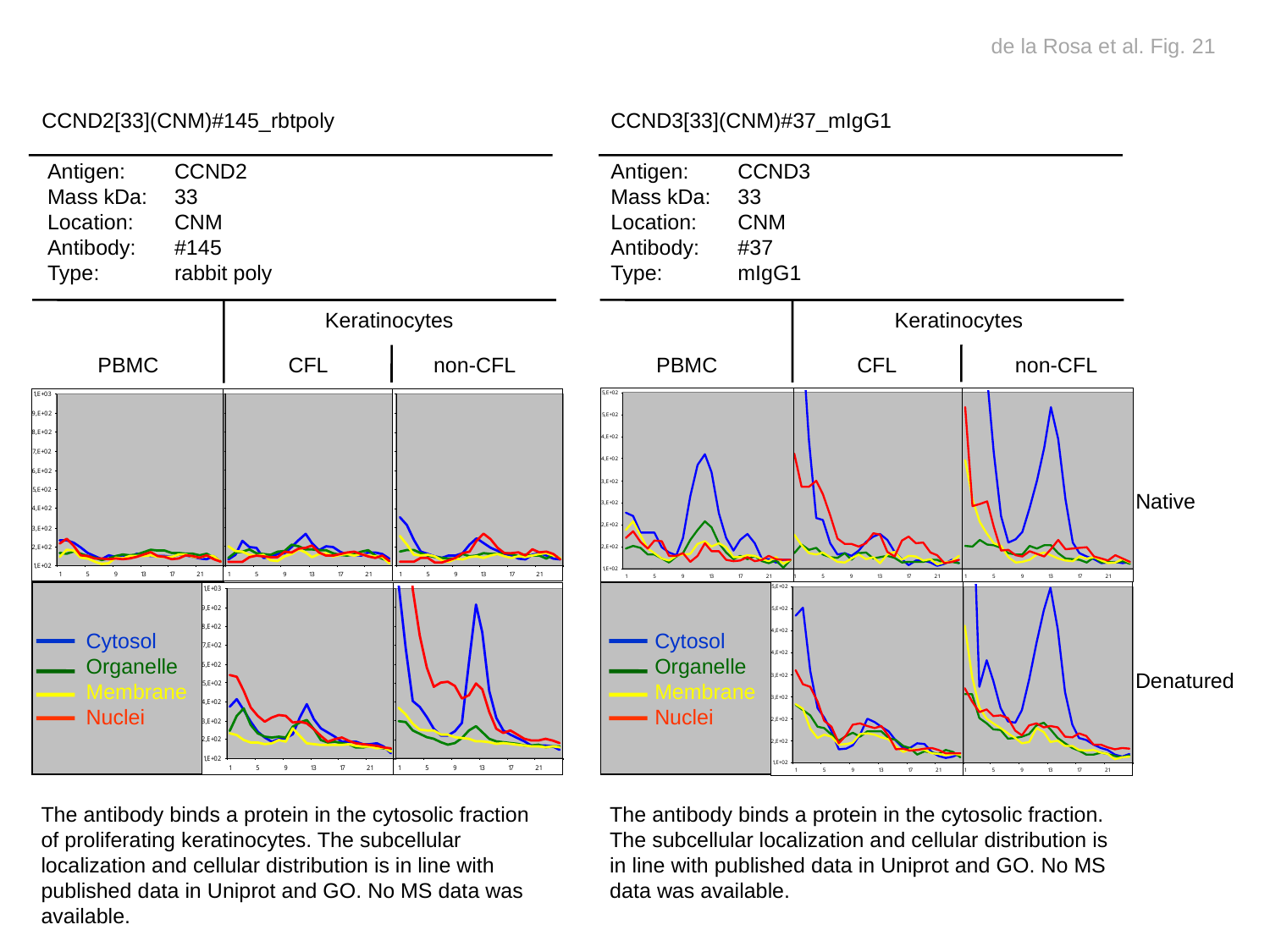

de la Rosa et al. Fig. <number>
CCND2[33](CNM)#145_rbtpoly
CCND3[33](CNM)#37_mIgG1
Antigen: 	CCND2
Mass kDa:	33
Location: 	CNM
Antibody: 	#145
Type:	rabbit poly
Antigen:	CCND3
Mass kDa:	33
Location: 	CNM
Antibody: 	#37
Type:	mIgG1
The antibody binds a protein in the cytosolic fraction of proliferating keratinocytes. The subcellular localization and cellular distribution is in line with published data in Uniprot and GO. No MS data was available.
The antibody binds a protein in the cytosolic fraction. The subcellular localization and cellular distribution is in line with published data in Uniprot and GO. No MS data was available.

## Slide 22
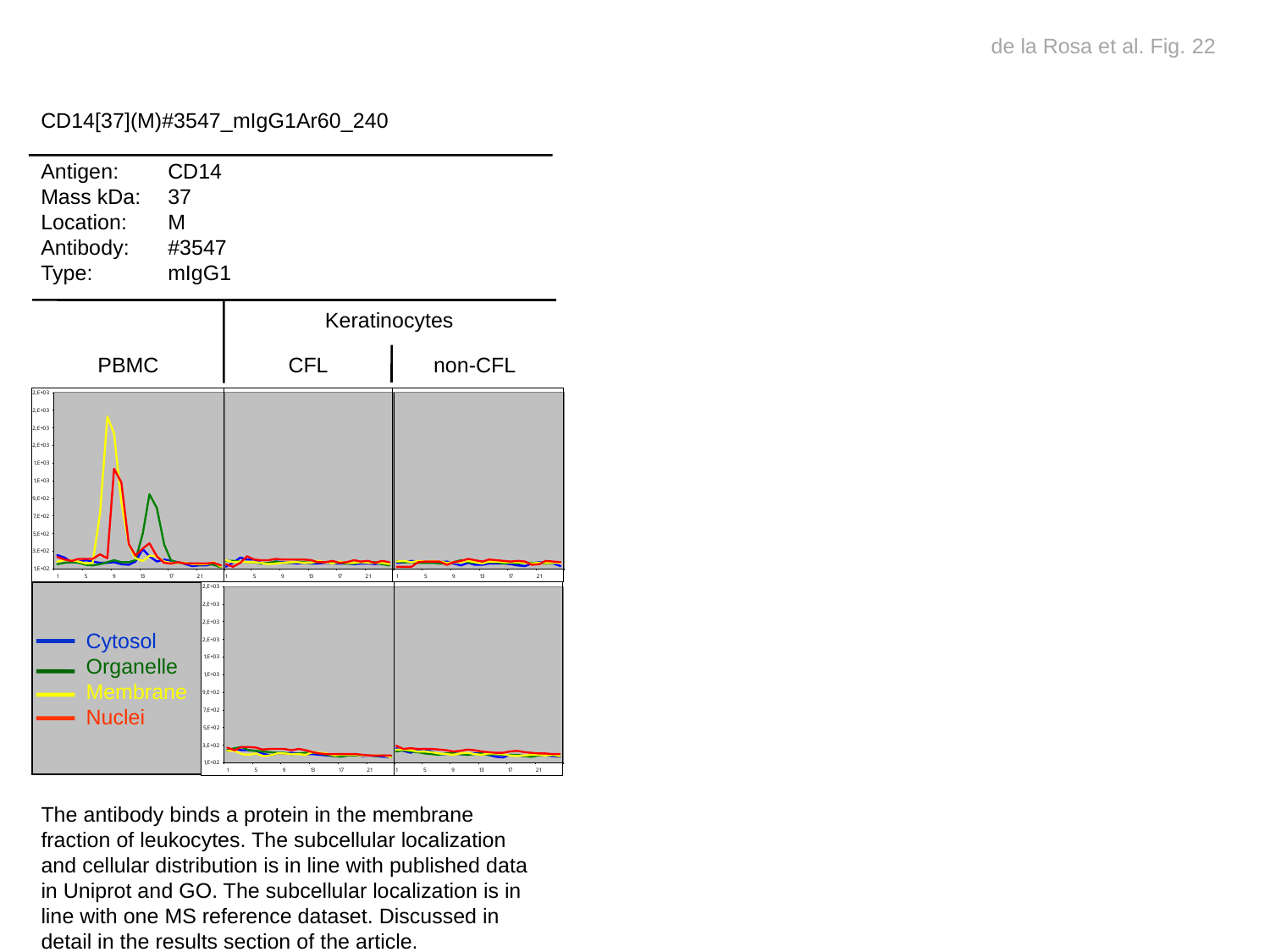

de la Rosa et al. Fig. <number>
CD14[37](M)#3547_mIgG1Ar60_240
# Antigen: 	CD14
Mass kDa:	37
Location: 	M
Antibody: 	#3547
Type:	mIgG1
The antibody binds a protein in the membrane fraction of leukocytes. The subcellular localization and cellular distribution is in line with published data in Uniprot and GO. The subcellular localization is in line with one MS reference dataset. Discussed in detail in the results section of the article.

## Slide 23
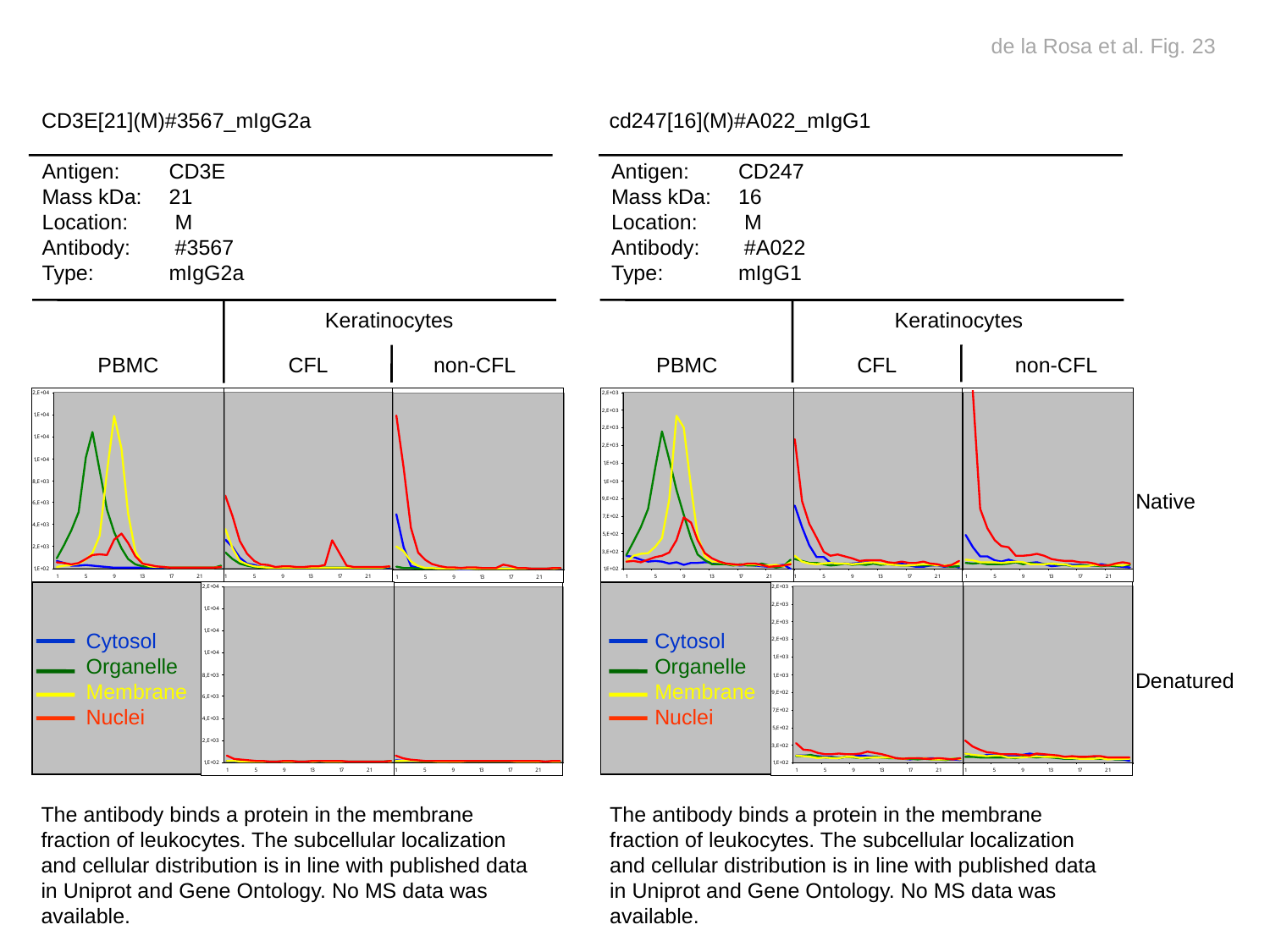

de la Rosa et al. Fig. <number>
CD3E[21](M)#3567_mIgG2a
cd247[16](M)#A022_mIgG1
Antigen:	CD3E
Mass kDa:	21
Location: 	 M
Antibody: 	 #3567
Type:	mIgG2a
Antigen: 	CD247
Mass kDa:	16
Location: 	 M
Antibody: 	 #A022
Type:	mIgG1
The antibody binds a protein in the membrane fraction of leukocytes. The subcellular localization and cellular distribution is in line with published data in Uniprot and Gene Ontology. No MS data was available.
The antibody binds a protein in the membrane fraction of leukocytes. The subcellular localization and cellular distribution is in line with published data in Uniprot and Gene Ontology. No MS data was available.

## Slide 24
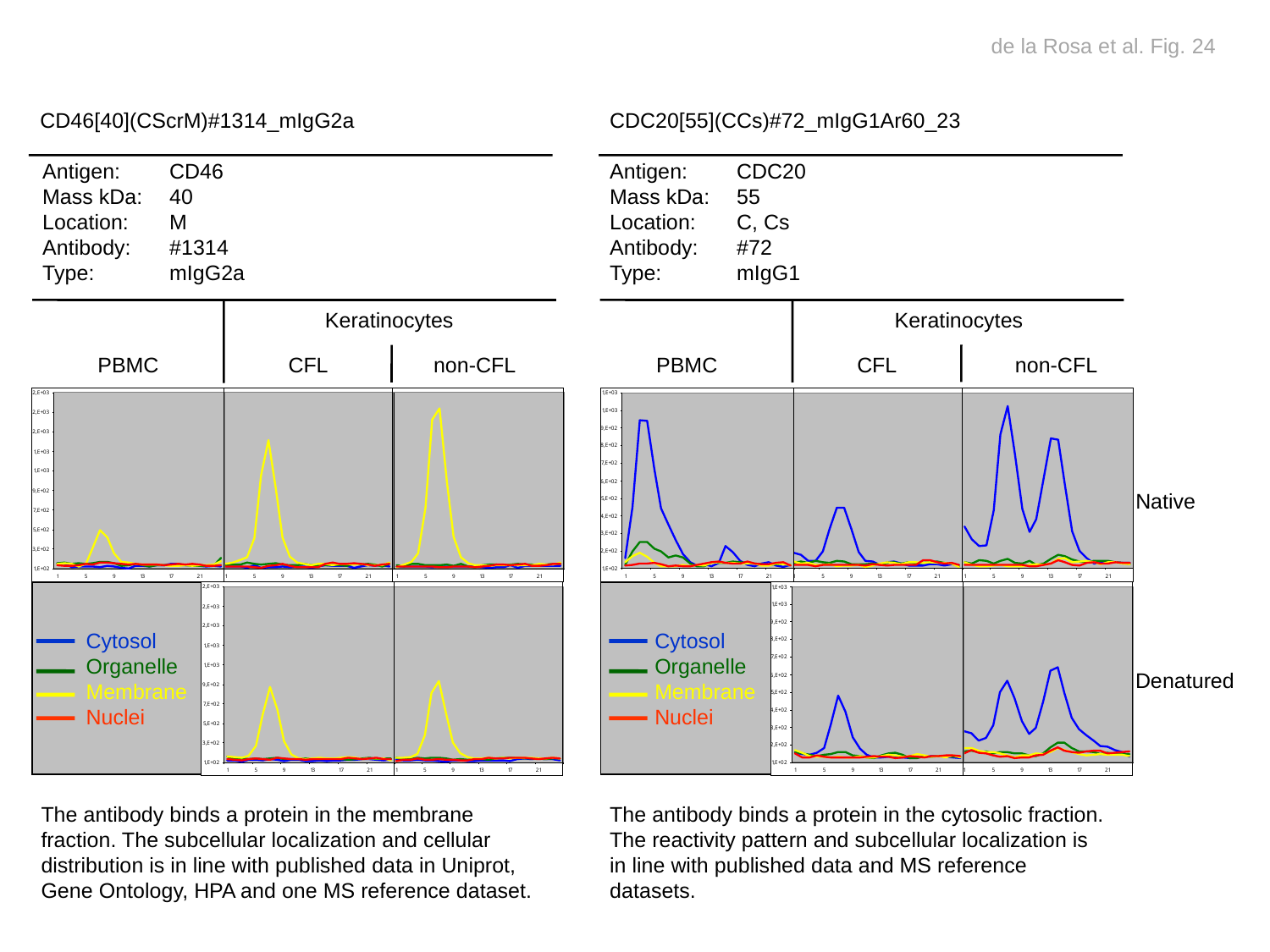

de la Rosa et al. Fig. <number>
# CDC20[55](CCs)#72_mIgG1Ar60_23
CD46[40](CScrM)#1314_mIgG2a
Antigen: 	CDC20
Mass kDa:	55
Location: 	C, Cs
Antibody: 	#72
Type:	mIgG1
Antigen: 	CD46
Mass kDa:	40
Location: 	M
Antibody: 	#1314
Type:	mIgG2a
The antibody binds a protein in the membrane fraction. The subcellular localization and cellular distribution is in line with published data in Uniprot, Gene Ontology, HPA and one MS reference dataset.
The antibody binds a protein in the cytosolic fraction. The reactivity pattern and subcellular localization is in line with published data and MS reference datasets.

## Slide 25
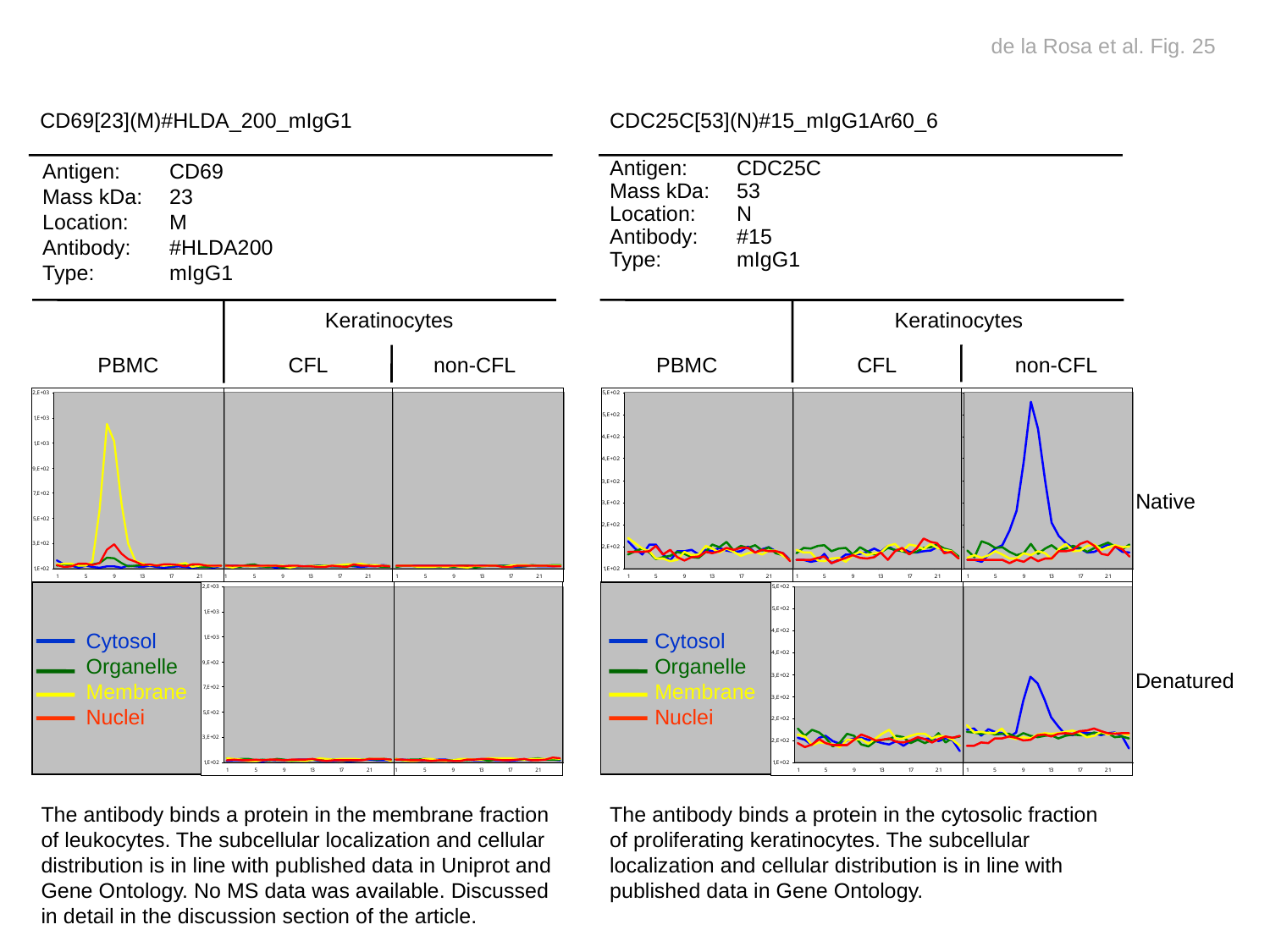

de la Rosa et al. Fig. <number>
# CDC25C[53](N)#15_mIgG1Ar60_6
CD69[23](M)#HLDA_200_mIgG1
Antigen: 	CD69
Mass kDa:	23
Location: 	M
Antibody: 	#HLDA200
Type:	mIgG1
Antigen: 	CDC25C
Mass kDa:	53
Location: 	N
Antibody: 	#15
Type:	mIgG1
The antibody binds a protein in the membrane fraction of leukocytes. The subcellular localization and cellular distribution is in line with published data in Uniprot and Gene Ontology. No MS data was available. Discussed in detail in the discussion section of the article.
The antibody binds a protein in the cytosolic fraction of proliferating keratinocytes. The subcellular localization and cellular distribution is in line with published data in Gene Ontology.

## Slide 26
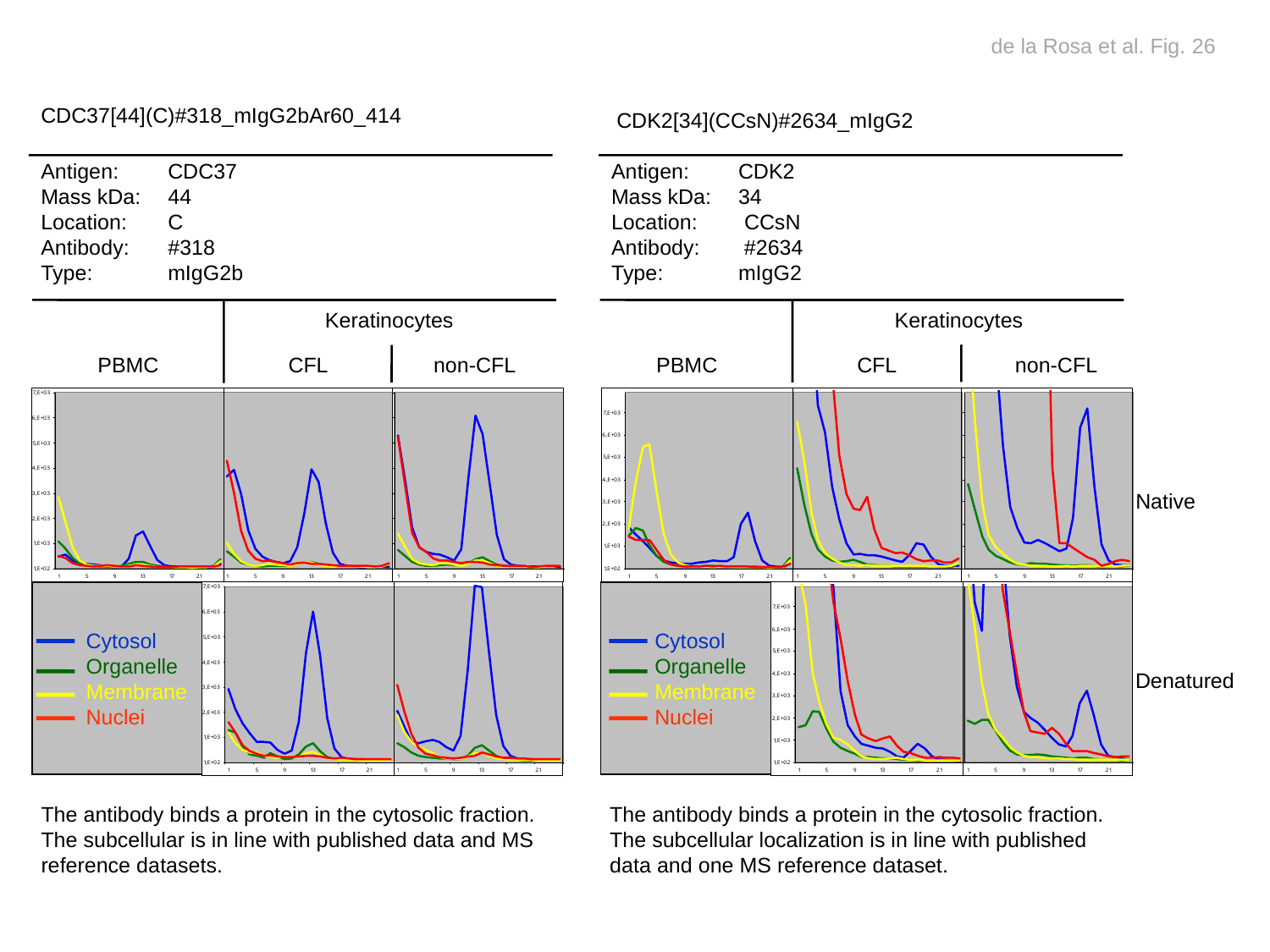

de la Rosa et al. Fig. <number>
# CDC37[44](C)#318_mIgG2bAr60_414
CDK2[34](CCsN)#2634_mIgG2
Antigen: 	CDC37
Mass kDa:	44
Location: 	C
Antibody: 	#318
Type:	mIgG2b
Antigen: 	CDK2
Mass kDa:	34
Location: 	 CCsN
Antibody: 	 #2634
Type:	mIgG2
The antibody binds a protein in the cytosolic fraction. The subcellular is in line with published data and MS reference datasets.
The antibody binds a protein in the cytosolic fraction. The subcellular localization is in line with published data and one MS reference dataset.

## Slide 27
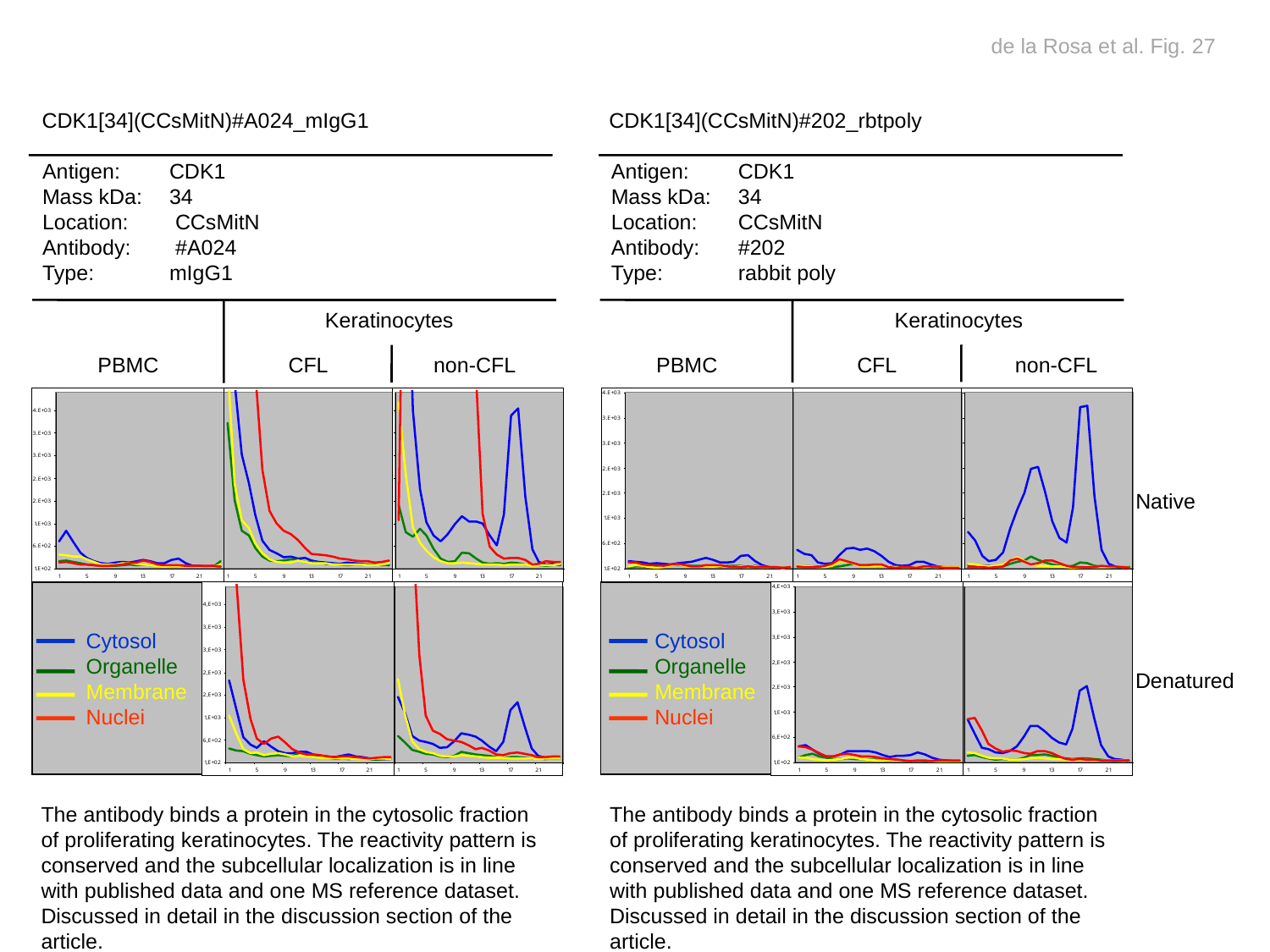

de la Rosa et al. Fig. <number>
CDK1[34](CCsMitN)#A024_mIgG1
CDK1[34](CCsMitN)#202_rbtpoly
Antigen: 	CDK1
Mass kDa:	34
Location: 	 CCsMitN
Antibody: 	 #A024
Type:	mIgG1
Antigen: 	CDK1
Mass kDa:	34
Location: 	CCsMitN
Antibody: 	#202
Type:	rabbit poly
The antibody binds a protein in the cytosolic fraction of proliferating keratinocytes. The reactivity pattern is conserved and the subcellular localization is in line with published data and one MS reference dataset. Discussed in detail in the discussion section of the article.
The antibody binds a protein in the cytosolic fraction of proliferating keratinocytes. The reactivity pattern is conserved and the subcellular localization is in line with published data and one MS reference dataset. Discussed in detail in the discussion section of the article.

## Slide 28
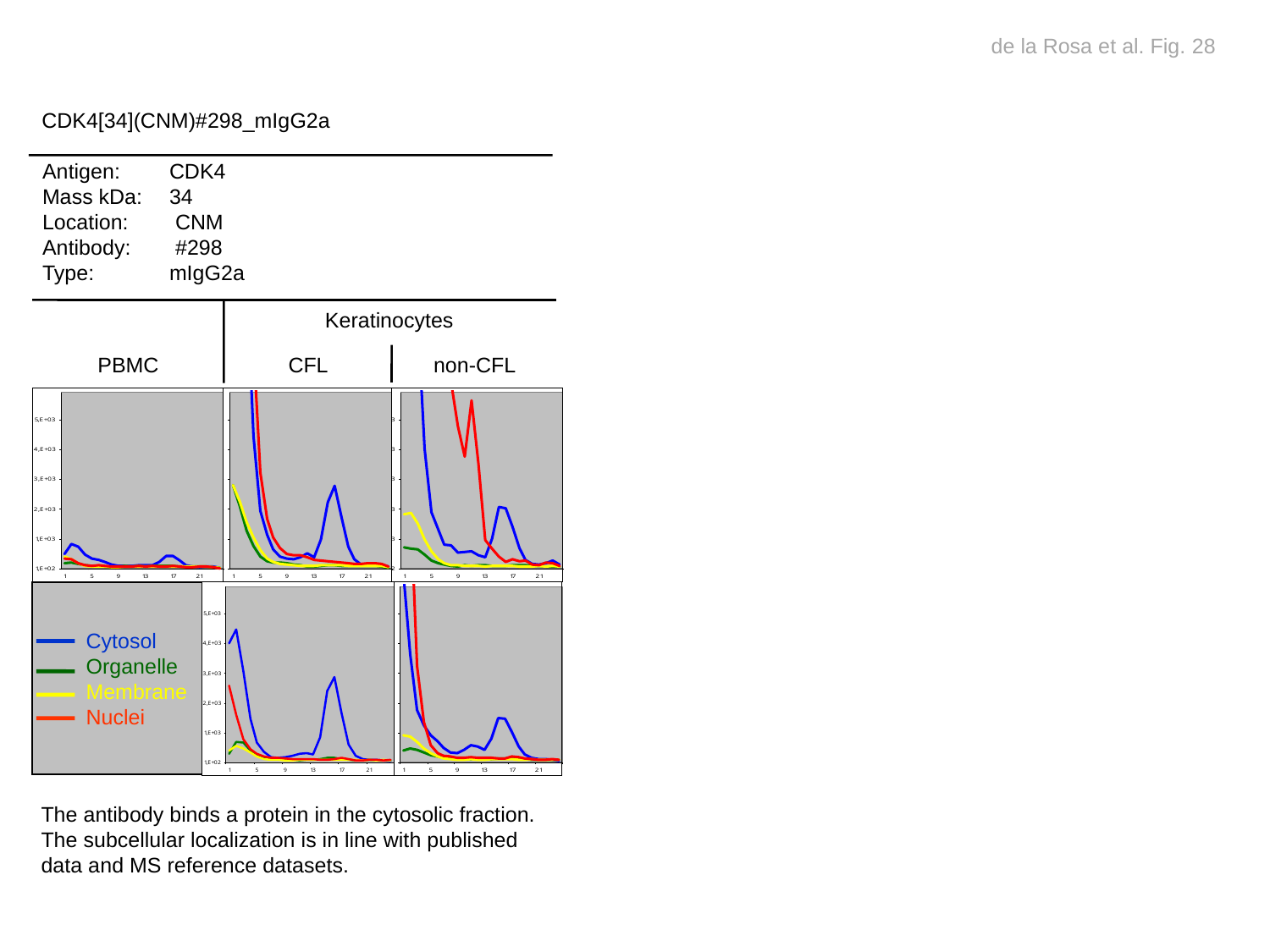

de la Rosa et al. Fig. <number>
CDK4[34](CNM)#298_mIgG2a
Antigen: 	CDK4
Mass kDa:	34
Location: 	 CNM
Antibody: 	 #298
Type:	mIgG2a
The antibody binds a protein in the cytosolic fraction. The subcellular localization is in line with published data and MS reference datasets.

## Slide 29
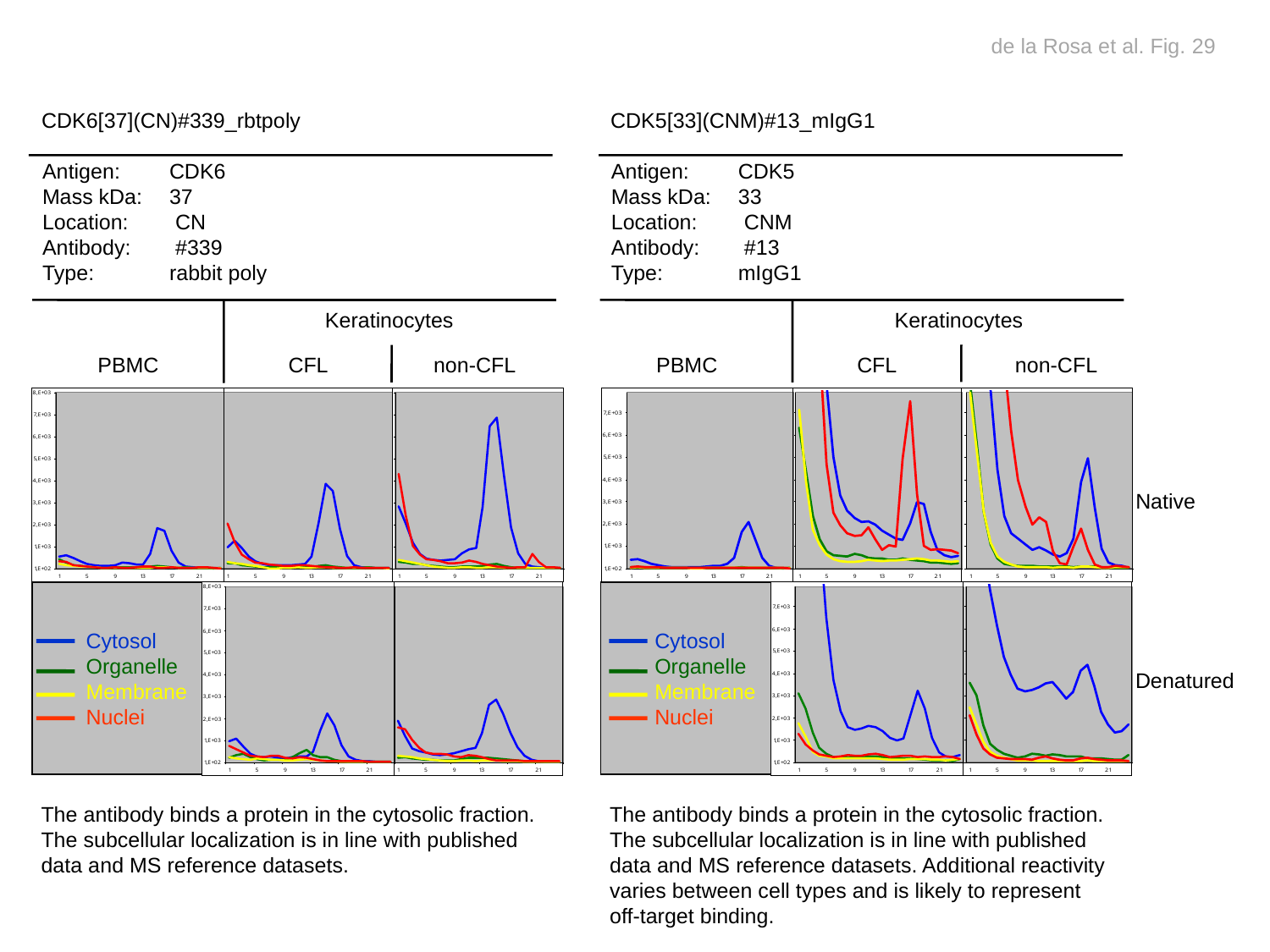

de la Rosa et al. Fig. <number>
CDK6[37](CN)#339_rbtpoly
CDK5[33](CNM)#13_mIgG1
Antigen: 	CDK6
Mass kDa:	37
Location: 	 CN
Antibody: 	 #339
Type:	rabbit poly
Antigen: 	CDK5
Mass kDa:	33
Location: 	 CNM
Antibody: 	 #13
Type:	mIgG1
The antibody binds a protein in the cytosolic fraction. The subcellular localization is in line with published data and MS reference datasets.
The antibody binds a protein in the cytosolic fraction. The subcellular localization is in line with published data and MS reference datasets. Additional reactivity varies between cell types and is likely to represent off-target binding.

## Slide 30
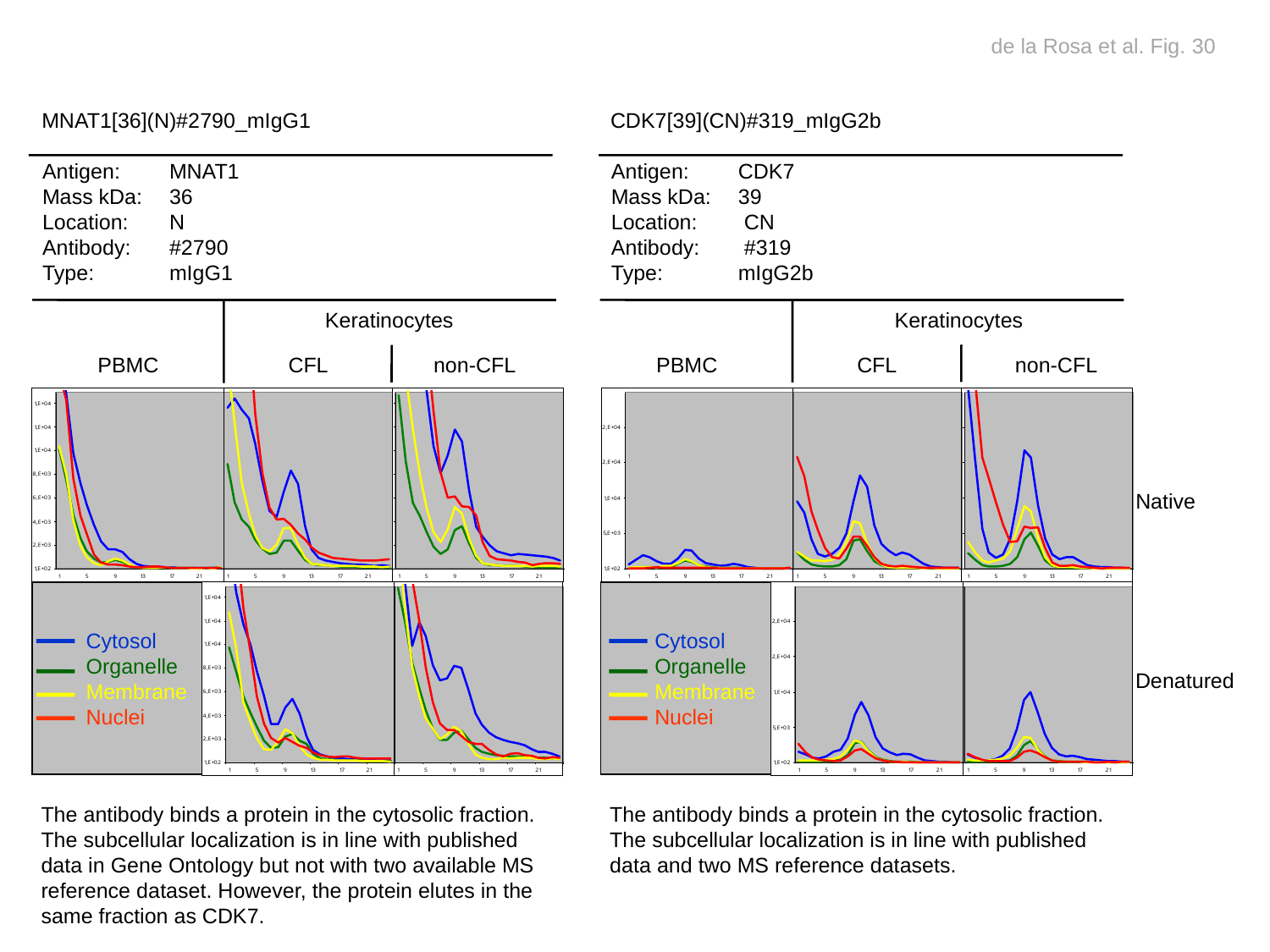

de la Rosa et al. Fig. <number>
MNAT1[36](N)#2790_mIgG1
CDK7[39](CN)#319_mIgG2b
Antigen: 	MNAT1
Mass kDa:	36
Location: 	N
Antibody: 	#2790
Type:	mIgG1
Antigen: 	CDK7
Mass kDa:	39
Location: 	 CN
Antibody: 	 #319
Type:	mIgG2b
The antibody binds a protein in the cytosolic fraction. The subcellular localization is in line with published data in Gene Ontology but not with two available MS reference dataset. However, the protein elutes in the same fraction as CDK7.
The antibody binds a protein in the cytosolic fraction. The subcellular localization is in line with published data and two MS reference datasets.

## Slide 31
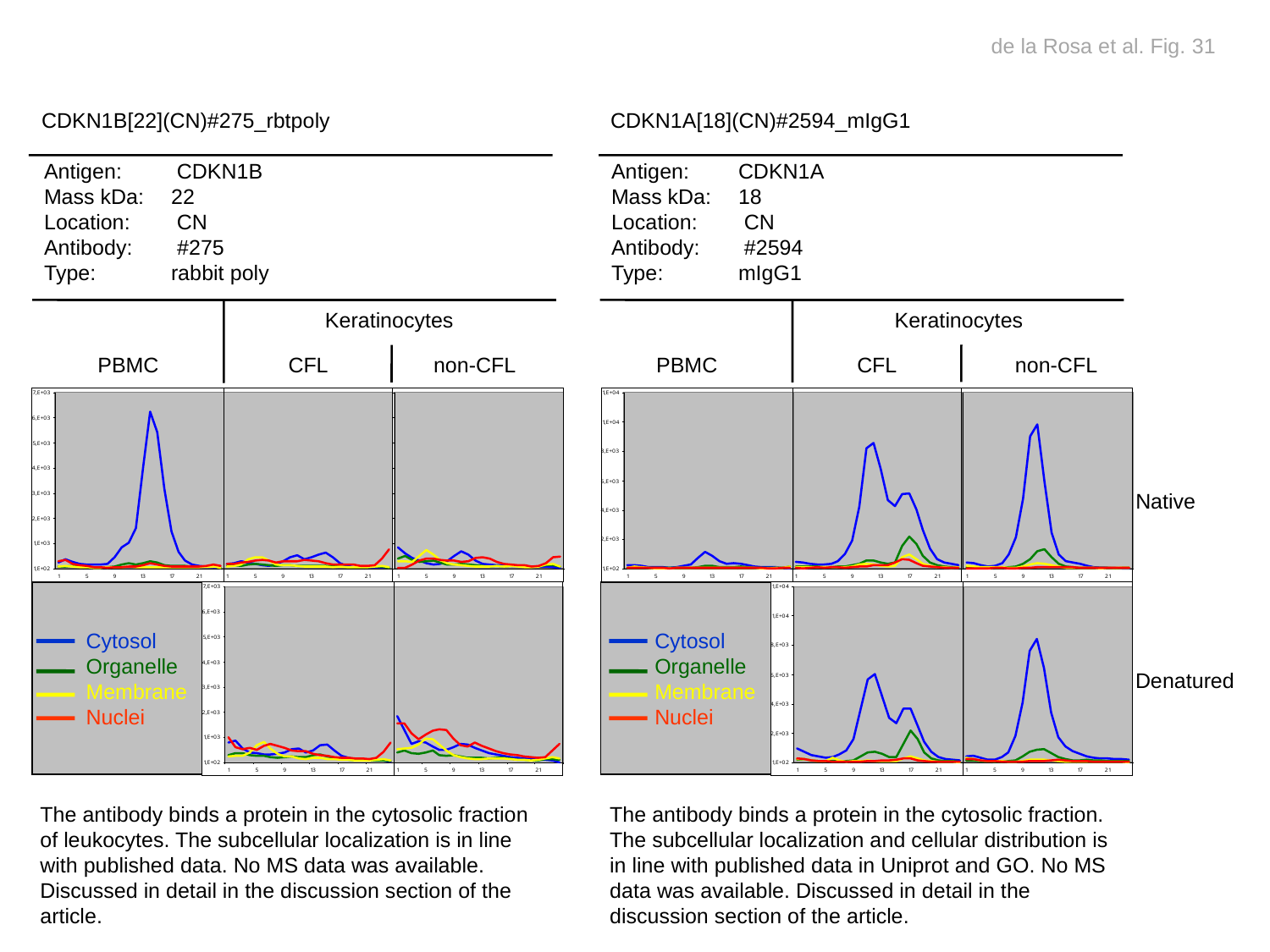

de la Rosa et al. Fig. <number>
CDKN1B[22](CN)#275_rbtpoly
CDKN1A[18](CN)#2594_mIgG1
Antigen:	 CDKN1B
Mass kDa:	22
Location: 	 CN
Antibody: 	 #275
Type:	rabbit poly
Antigen: 	CDKN1A
Mass kDa:	18
Location: 	 CN
Antibody: 	 #2594
Type:	mIgG1
The antibody binds a protein in the cytosolic fraction of leukocytes. The subcellular localization is in line with published data. No MS data was available. Discussed in detail in the discussion section of the article.
The antibody binds a protein in the cytosolic fraction. The subcellular localization and cellular distribution is in line with published data in Uniprot and GO. No MS data was available. Discussed in detail in the discussion section of the article.

## Slide 32
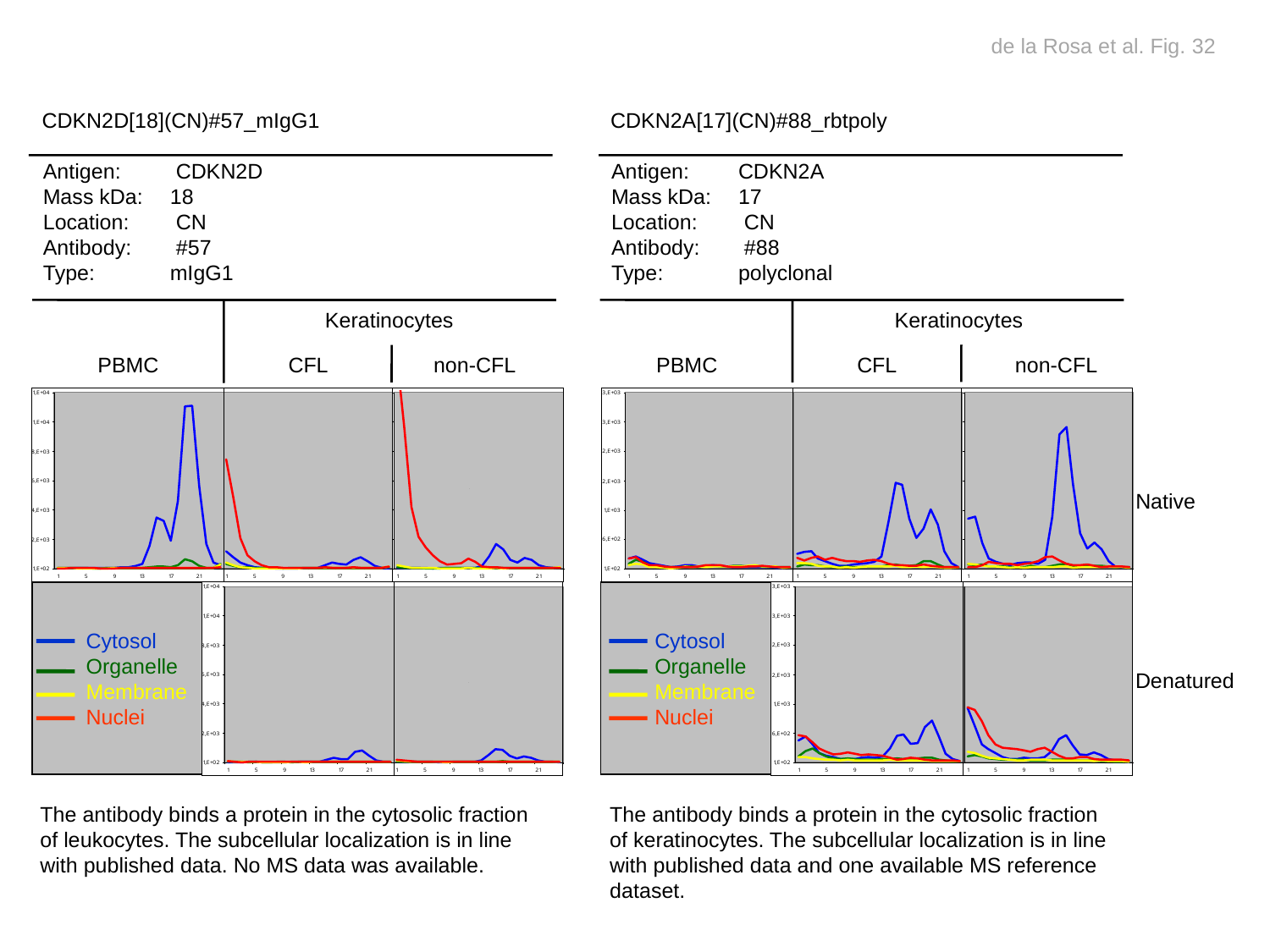

de la Rosa et al. Fig. <number>
CDKN2D[18](CN)#57_mIgG1
CDKN2A[17](CN)#88_rbtpoly
Antigen:	 CDKN2D
Mass kDa:	18
Location: 	 CN
Antibody: 	 #57
Type:	mIgG1
Antigen: 	CDKN2A
Mass kDa:	17
Location: 	 CN
Antibody: 	 #88
Type:	polyclonal
The antibody binds a protein in the cytosolic fraction of leukocytes. The subcellular localization is in line with published data. No MS data was available.
The antibody binds a protein in the cytosolic fraction of keratinocytes. The subcellular localization is in line with published data and one available MS reference dataset.

## Slide 33
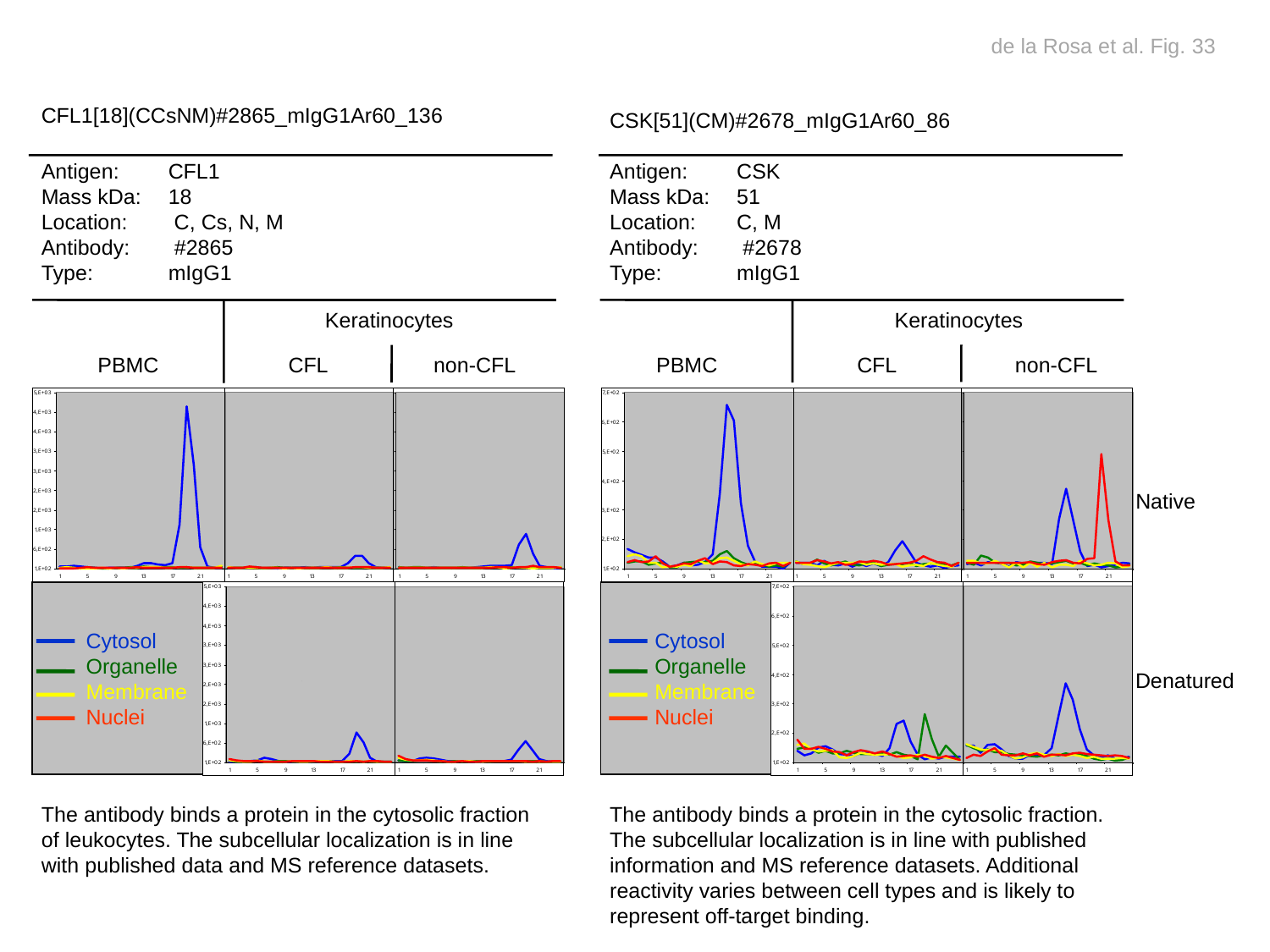

de la Rosa et al. Fig. <number>
# CFL1[18](CCsNM)#2865_mIgG1Ar60_136
CSK[51](CM)#2678_mIgG1Ar60_86
Antigen: 	CFL1
Mass kDa:	18
Location: 	 C, Cs, N, M
Antibody: 	 #2865
Type:	mIgG1
Antigen: 	CSK
Mass kDa:	51
Location: 	C, M
Antibody: 	 #2678
Type:	mIgG1
The antibody binds a protein in the cytosolic fraction of leukocytes. The subcellular localization is in line with published data and MS reference datasets.
The antibody binds a protein in the cytosolic fraction. The subcellular localization is in line with published information and MS reference datasets. Additional reactivity varies between cell types and is likely to represent off-target binding.

## Slide 34
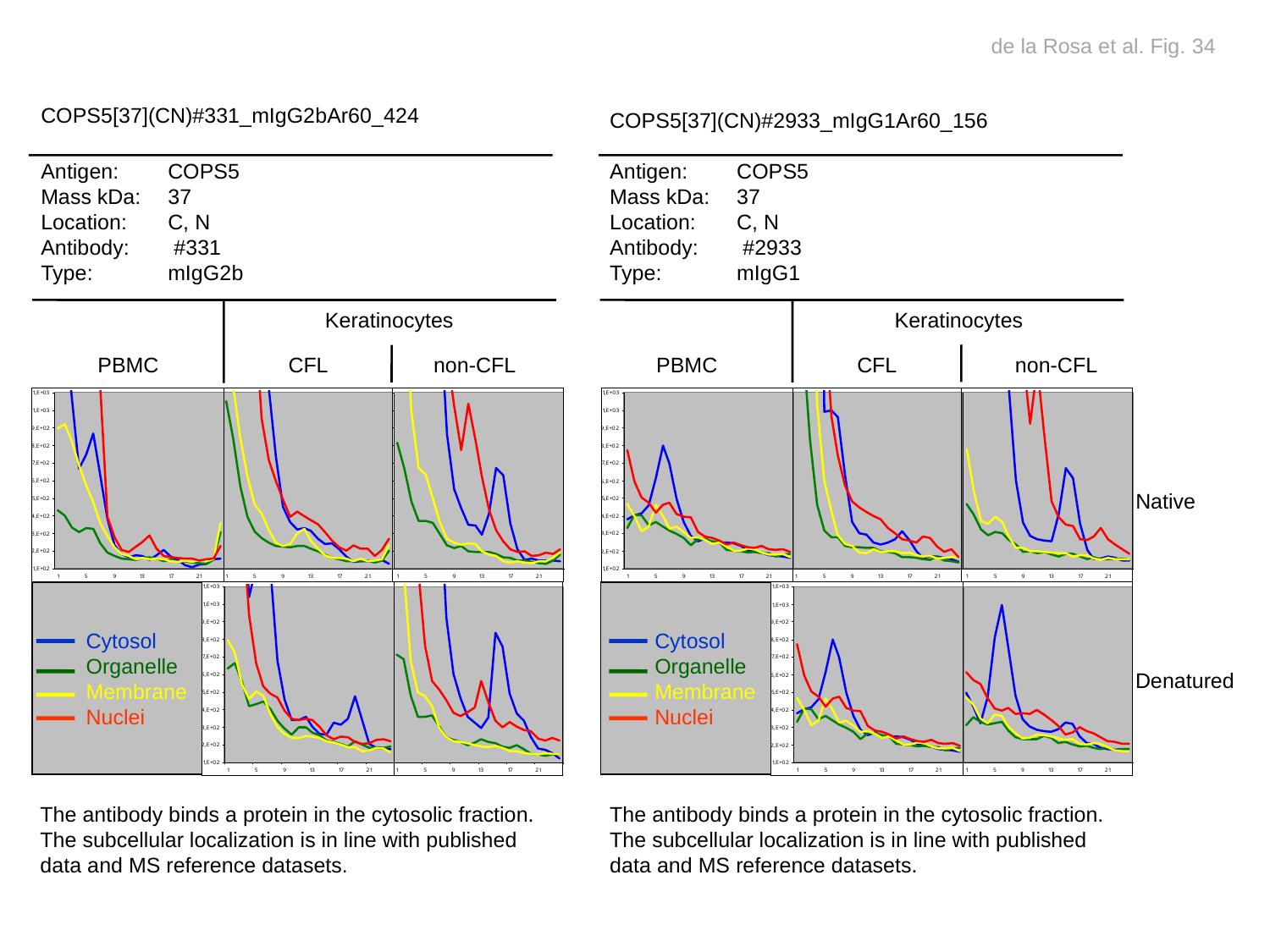

de la Rosa et al. Fig. <number>
# COPS5[37](CN)#331_mIgG2bAr60_424
COPS5[37](CN)#2933_mIgG1Ar60_156
Antigen: 	COPS5
Mass kDa:	37
Location: 	C, N
Antibody: 	 #331
Type:	mIgG2b
Antigen: 	COPS5
Mass kDa:	37
Location: 	C, N
Antibody: 	 #2933
Type:	mIgG1
The antibody binds a protein in the cytosolic fraction. The subcellular localization is in line with published data and MS reference datasets.
The antibody binds a protein in the cytosolic fraction. The subcellular localization is in line with published data and MS reference datasets.

## Slide 35
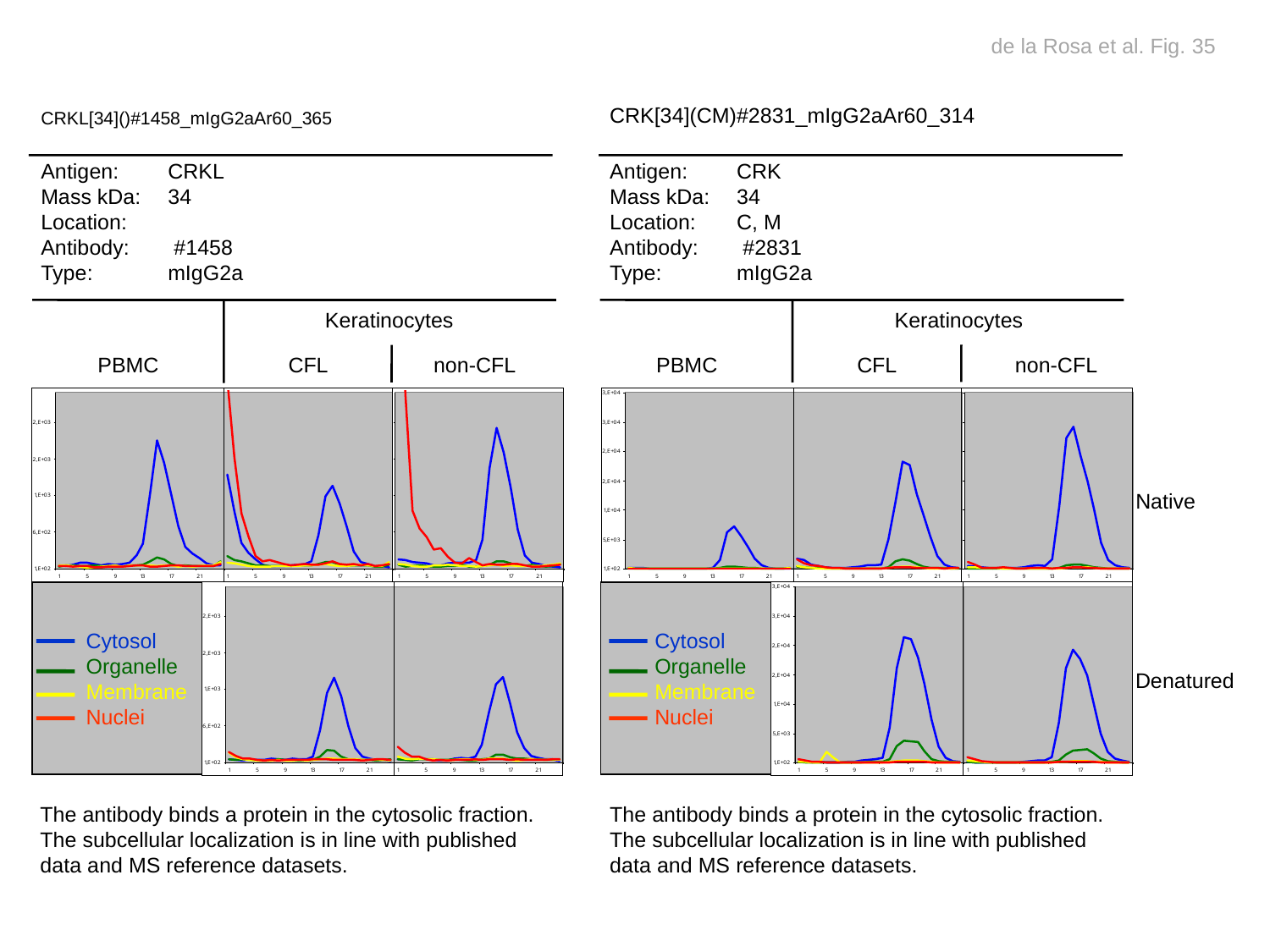

de la Rosa et al. Fig. <number>
# CRKL[34]()#1458_mIgG2aAr60_365
CRK[34](CM)#2831_mIgG2aAr60_314
Antigen: 	CRKL
Mass kDa:	34
Location:
Antibody: 	 #1458
Type:	mIgG2a
Antigen: 	CRK
Mass kDa:	34
Location: 	C, M
Antibody: 	 #2831
Type:	mIgG2a
The antibody binds a protein in the cytosolic fraction. The subcellular localization is in line with published data and MS reference datasets.
The antibody binds a protein in the cytosolic fraction. The subcellular localization is in line with published data and MS reference datasets.

## Slide 36
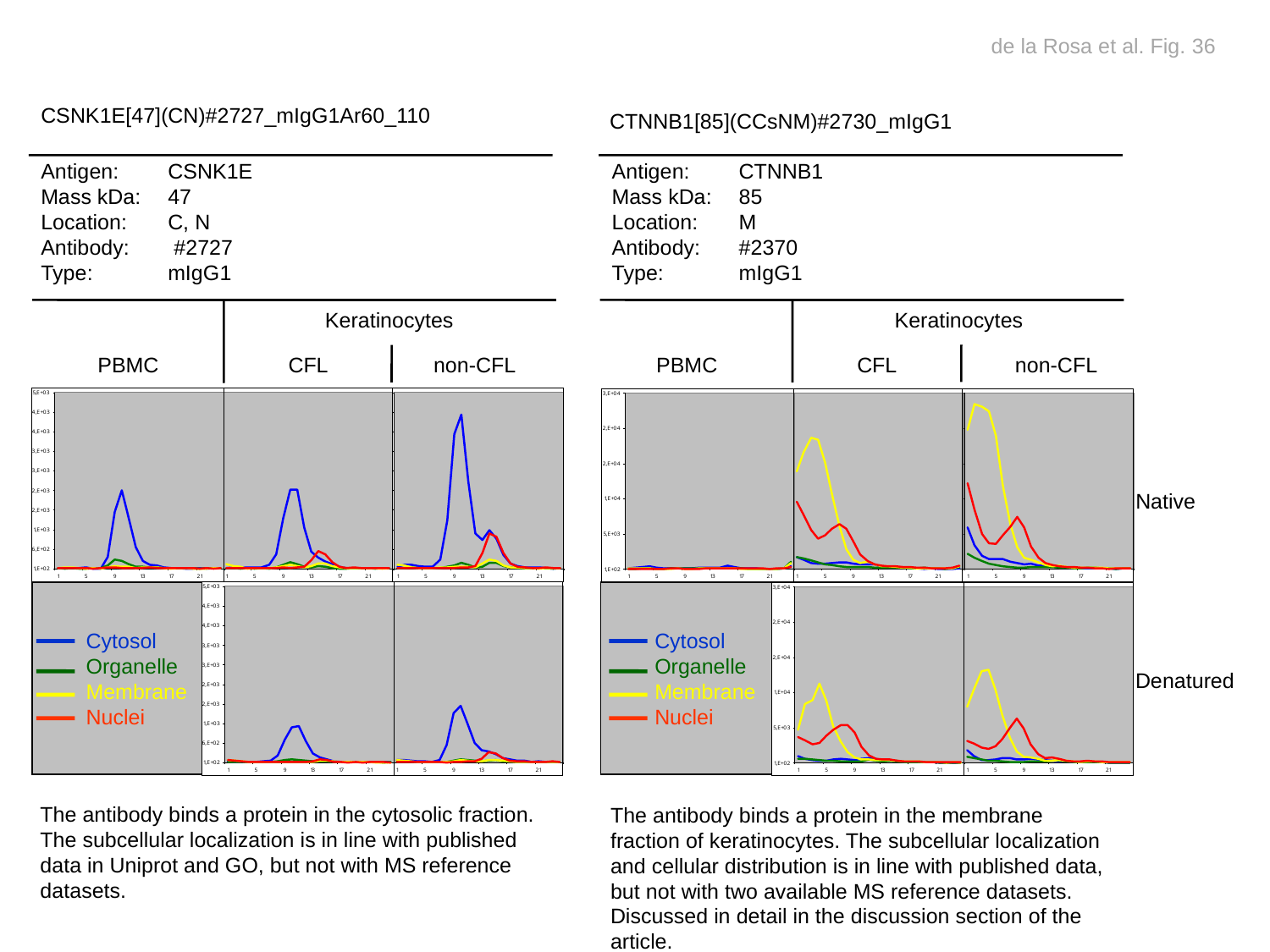

de la Rosa et al. Fig. <number>
# CSNK1E[47](CN)#2727_mIgG1Ar60_110
CTNNB1[85](CCsNM)#2730_mIgG1
Antigen: 	CSNK1E
Mass kDa:	47
Location: 	C, N
Antibody: 	 #2727
Type:	mIgG1
Antigen: 	CTNNB1
Mass kDa:	85
Location: 	M
Antibody: 	#2370
Type:	mIgG1
The antibody binds a protein in the cytosolic fraction. The subcellular localization is in line with published data in Uniprot and GO, but not with MS reference datasets.
The antibody binds a protein in the membrane fraction of keratinocytes. The subcellular localization and cellular distribution is in line with published data, but not with two available MS reference datasets. Discussed in detail in the discussion section of the article.

## Slide 37
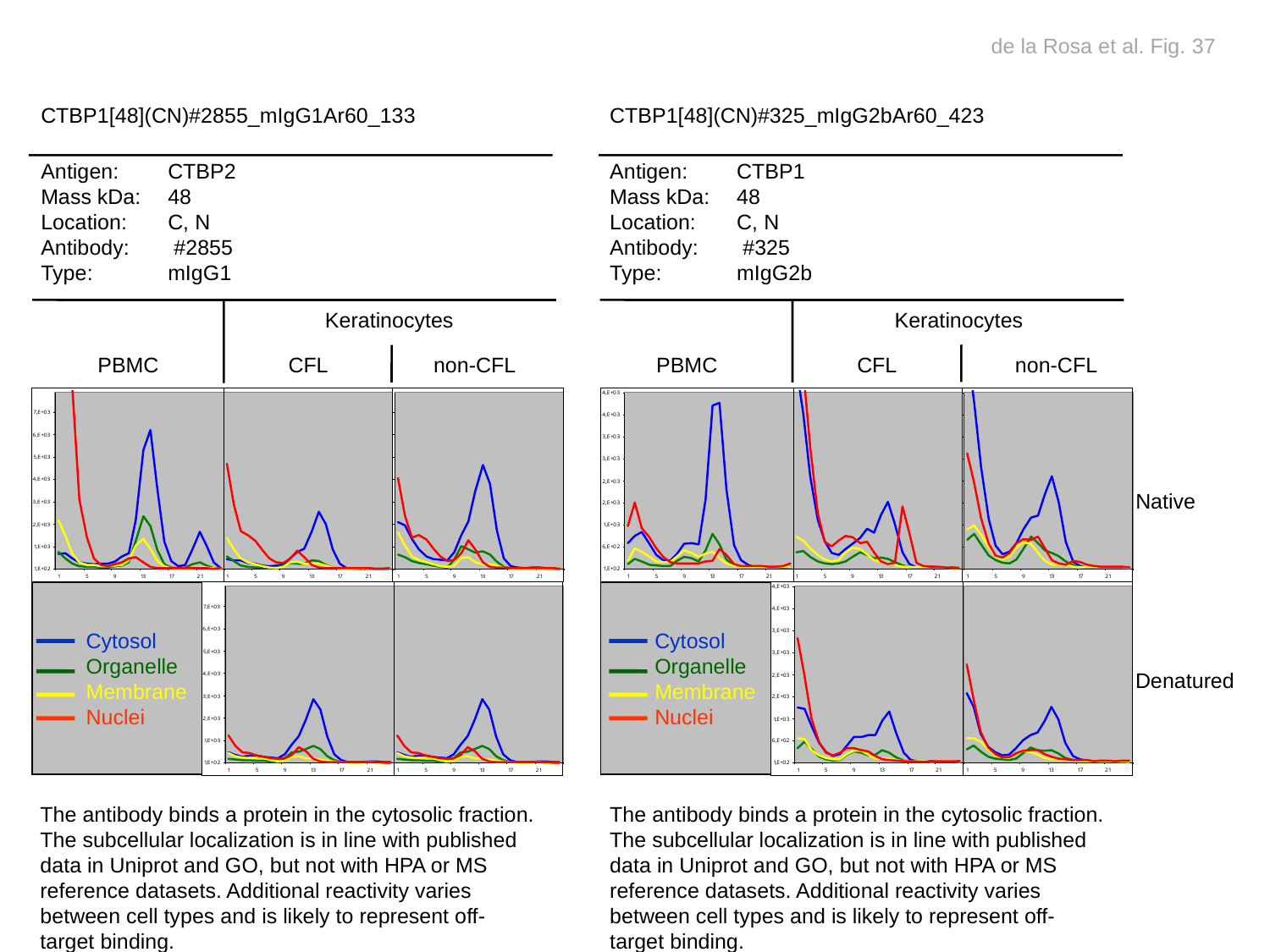

de la Rosa et al. Fig. <number>
# CTBP1[48](CN)#2855_mIgG1Ar60_133
CTBP1[48](CN)#325_mIgG2bAr60_423
Antigen: 	CTBP2
Mass kDa:	48
Location: 	C, N
Antibody: 	 #2855
Type:	mIgG1
Antigen: 	CTBP1
Mass kDa:	48
Location: 	C, N
Antibody: 	 #325
Type:	mIgG2b
The antibody binds a protein in the cytosolic fraction. The subcellular localization is in line with published data in Uniprot and GO, but not with HPA or MS reference datasets. Additional reactivity varies between cell types and is likely to represent off-target binding.
The antibody binds a protein in the cytosolic fraction. The subcellular localization is in line with published data in Uniprot and GO, but not with HPA or MS reference datasets. Additional reactivity varies between cell types and is likely to represent off-target binding.

## Slide 38
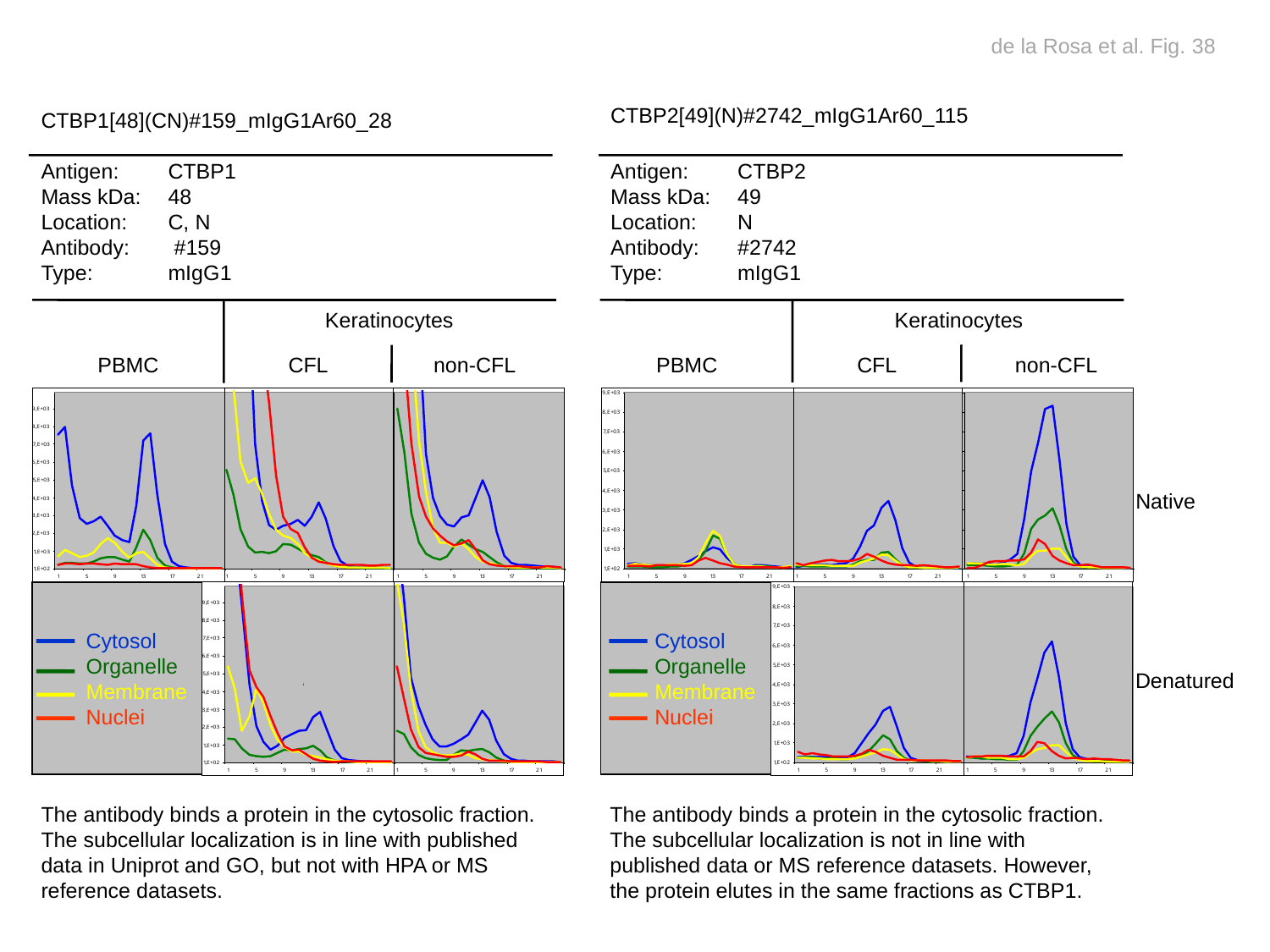

de la Rosa et al. Fig. <number>
CTBP1[48](CN)#159_mIgG1Ar60_28
# CTBP2[49](N)#2742_mIgG1Ar60_115
Antigen: 	CTBP1
Mass kDa:	48
Location: 	C, N
Antibody: 	 #159
Type:	mIgG1
Antigen: 	CTBP2
Mass kDa:	49
Location: 	N
Antibody: 	#2742
Type:	mIgG1
The antibody binds a protein in the cytosolic fraction. The subcellular localization is in line with published data in Uniprot and GO, but not with HPA or MS reference datasets.
The antibody binds a protein in the cytosolic fraction. The subcellular localization is not in line with published data or MS reference datasets. However, the protein elutes in the same fractions as CTBP1.

## Slide 39
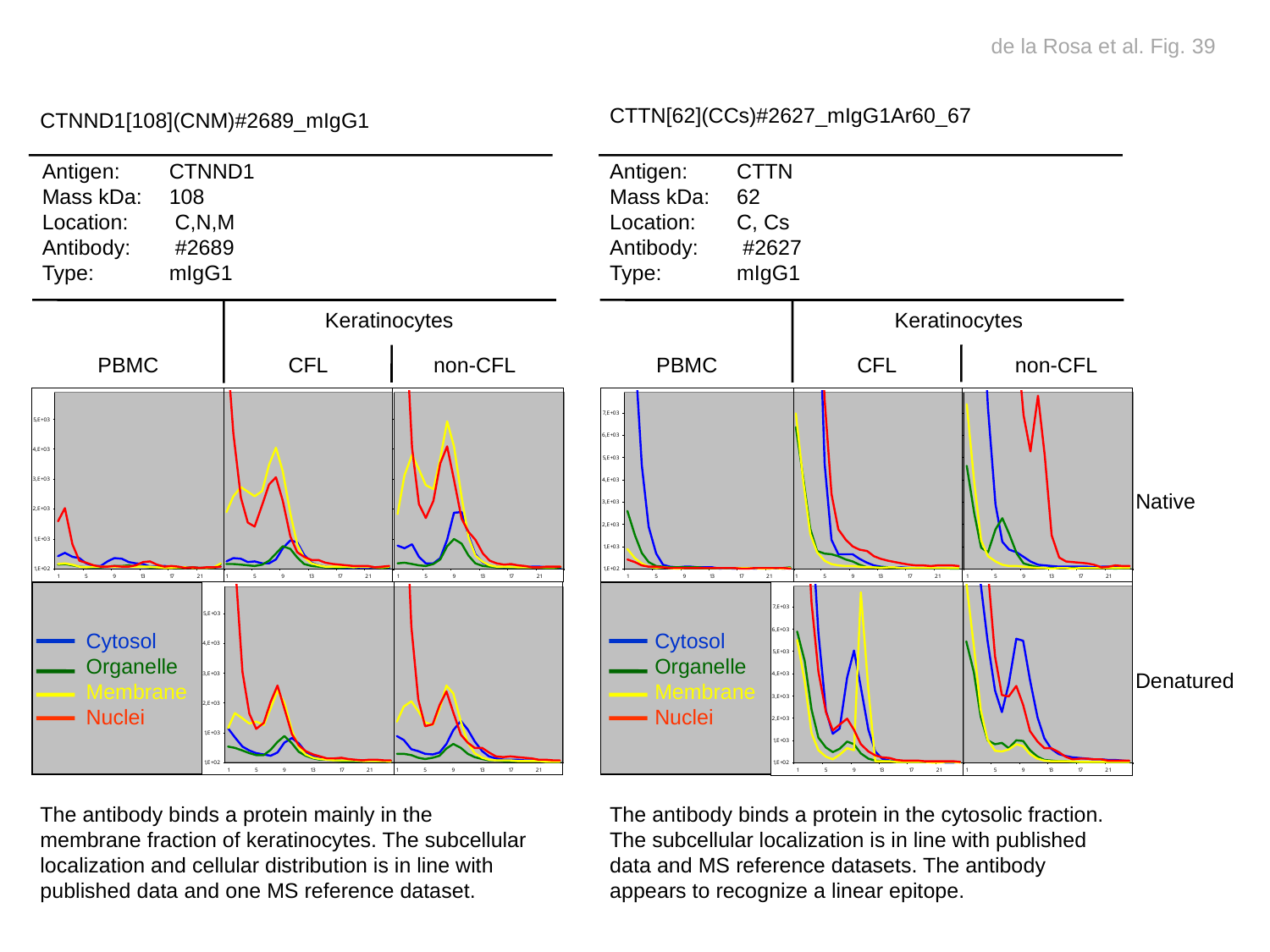

de la Rosa et al. Fig. <number>
# CTTN[62](CCs)#2627_mIgG1Ar60_67
CTNND1[108](CNM)#2689_mIgG1
Antigen:	CTNND1
Mass kDa:	108
Location: 	 C,N,M
Antibody: 	 #2689
Type:	mIgG1
Antigen: 	CTTN
Mass kDa:	62
Location: 	C, Cs
Antibody: 	 #2627
Type:	mIgG1
The antibody binds a protein mainly in the membrane fraction of keratinocytes. The subcellular localization and cellular distribution is in line with published data and one MS reference dataset.
The antibody binds a protein in the cytosolic fraction. The subcellular localization is in line with published data and MS reference datasets. The antibody appears to recognize a linear epitope.

## Slide 40
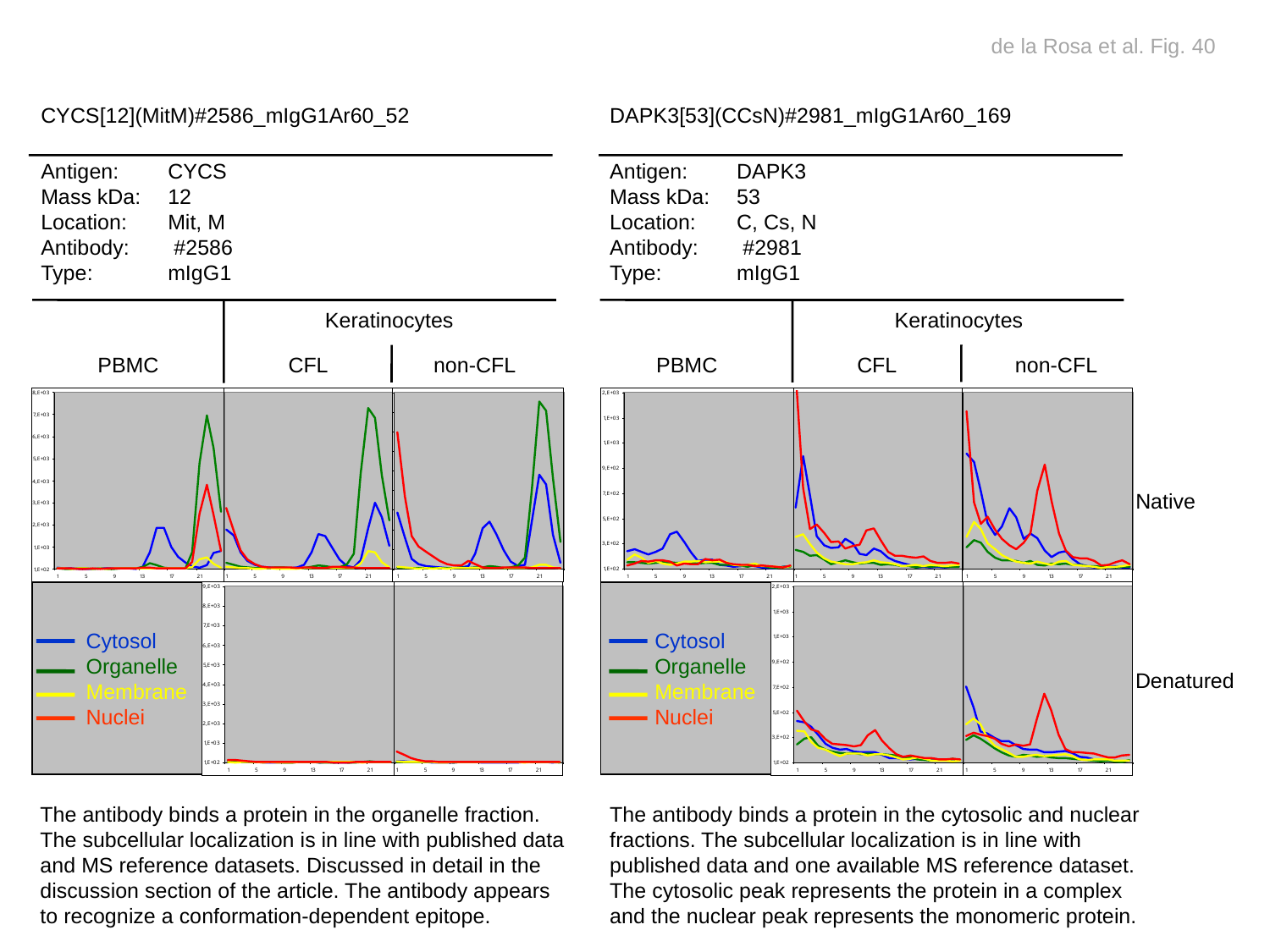

de la Rosa et al. Fig. <number>
# CYCS[12](MitM)#2586_mIgG1Ar60_52
DAPK3[53](CCsN)#2981_mIgG1Ar60_169
Antigen: 	CYCS
Mass kDa:	12
Location: 	Mit, M
Antibody: 	 #2586
Type:	mIgG1
Antigen: 	DAPK3
Mass kDa:	53
Location: 	C, Cs, N
Antibody: 	 #2981
Type:	mIgG1
The antibody binds a protein in the organelle fraction. The subcellular localization is in line with published data and MS reference datasets. Discussed in detail in the discussion section of the article. The antibody appears to recognize a conformation-dependent epitope.
The antibody binds a protein in the cytosolic and nuclear fractions. The subcellular localization is in line with published data and one available MS reference dataset. The cytosolic peak represents the protein in a complex and the nuclear peak represents the monomeric protein.

## Slide 41
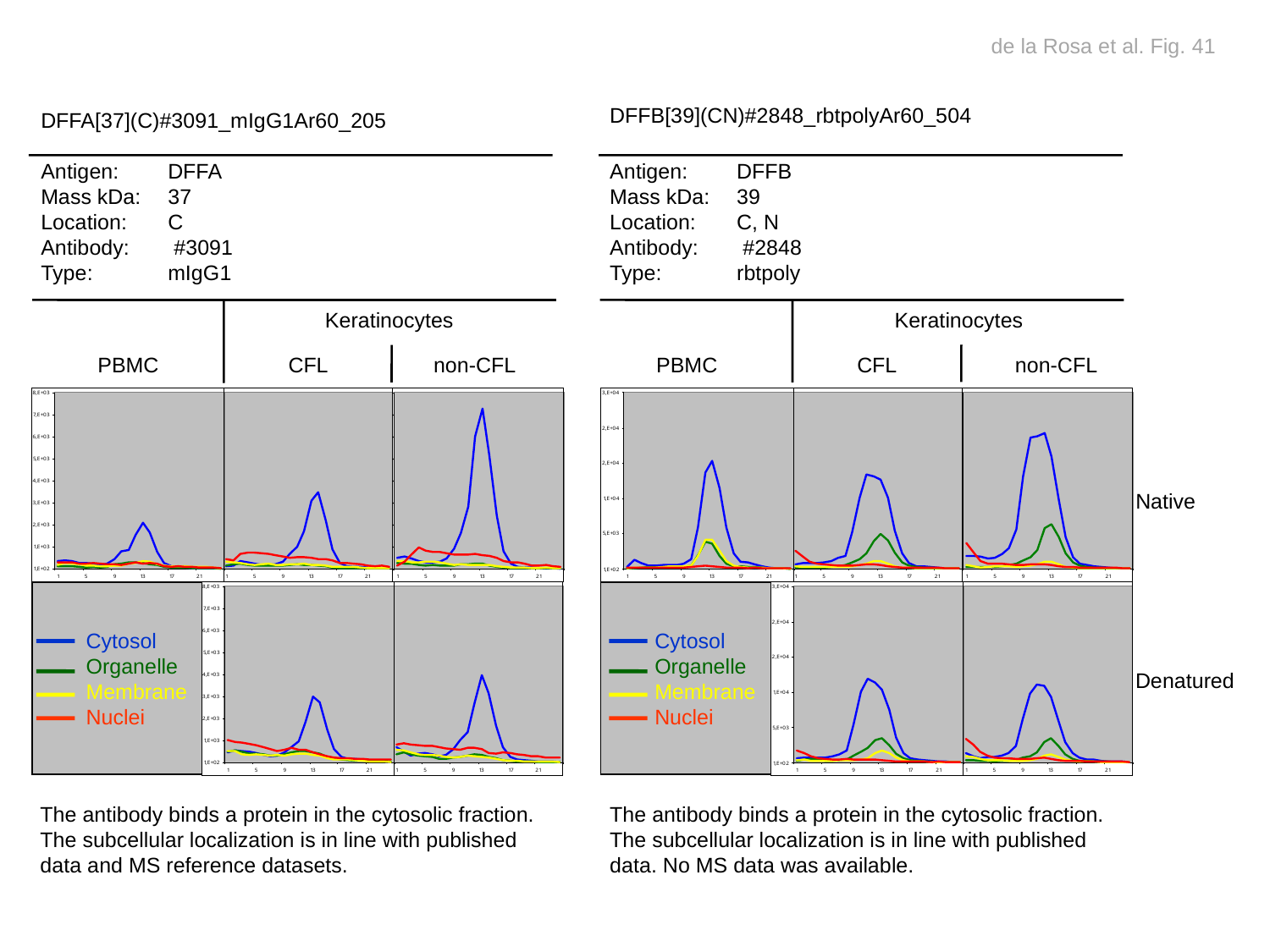

de la Rosa et al. Fig. <number>
# DFFA[37](C)#3091_mIgG1Ar60_205
DFFB[39](CN)#2848_rbtpolyAr60_504
Antigen: 	DFFA
Mass kDa:	37
Location: 	C
Antibody: 	 #3091
Type:	mIgG1
Antigen: 	DFFB
Mass kDa:	39
Location: 	C, N
Antibody: 	 #2848
Type:	rbtpoly
The antibody binds a protein in the cytosolic fraction. The subcellular localization is in line with published data and MS reference datasets.
The antibody binds a protein in the cytosolic fraction. The subcellular localization is in line with published data. No MS data was available.

## Slide 42
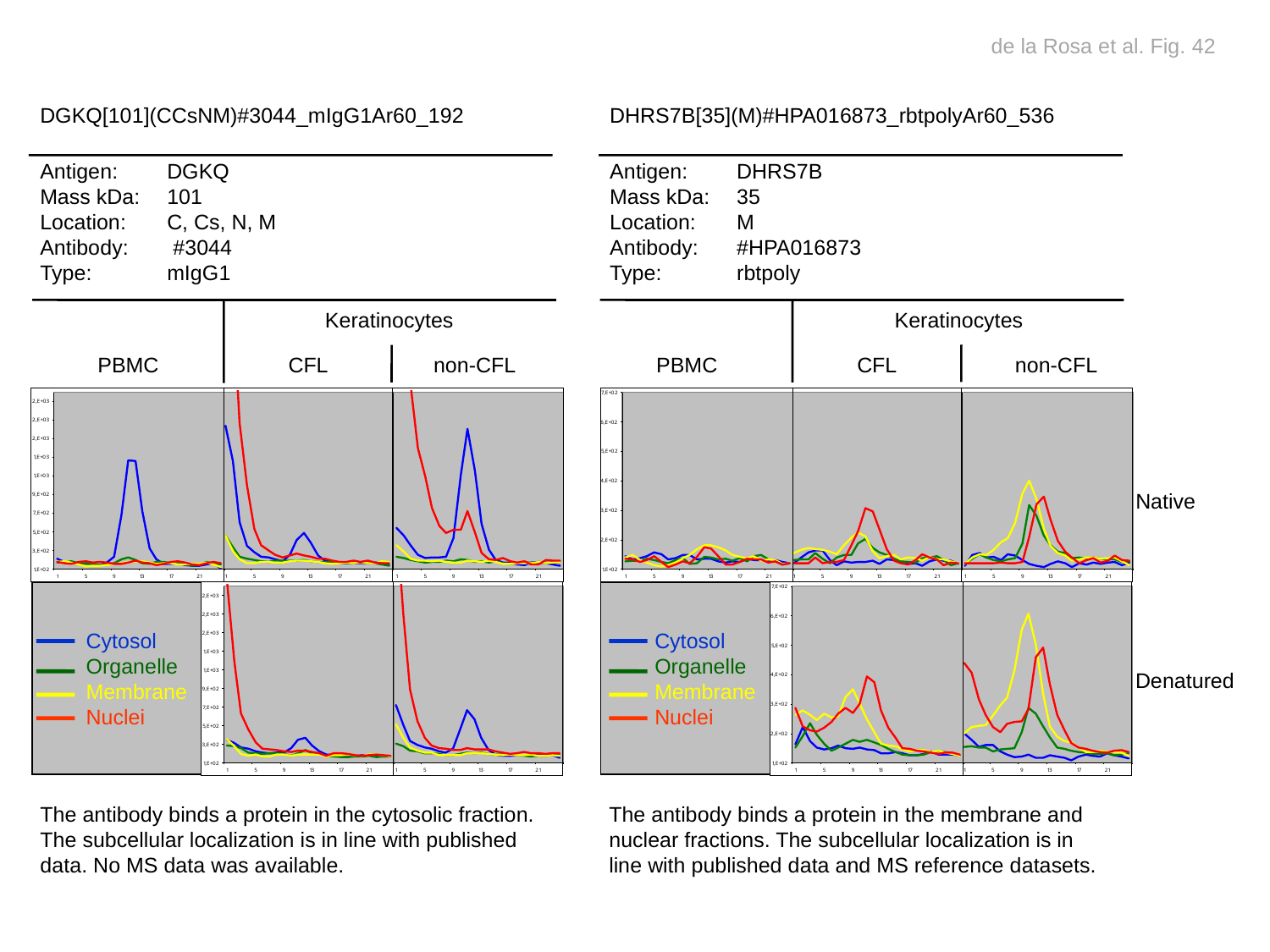

de la Rosa et al. Fig. <number>
# DGKQ[101](CCsNM)#3044_mIgG1Ar60_192
DHRS7B[35](M)#HPA016873_rbtpolyAr60_536
Antigen: 	DGKQ
Mass kDa:	101
Location: 	C, Cs, N, M
Antibody: 	 #3044
Type:	mIgG1
Antigen: 	DHRS7B
Mass kDa:	35
Location: 	M
Antibody: 	#HPA016873
Type:	rbtpoly
The antibody binds a protein in the cytosolic fraction. The subcellular localization is in line with published data. No MS data was available.
The antibody binds a protein in the membrane and nuclear fractions. The subcellular localization is in line with published data and MS reference datasets.

## Slide 43
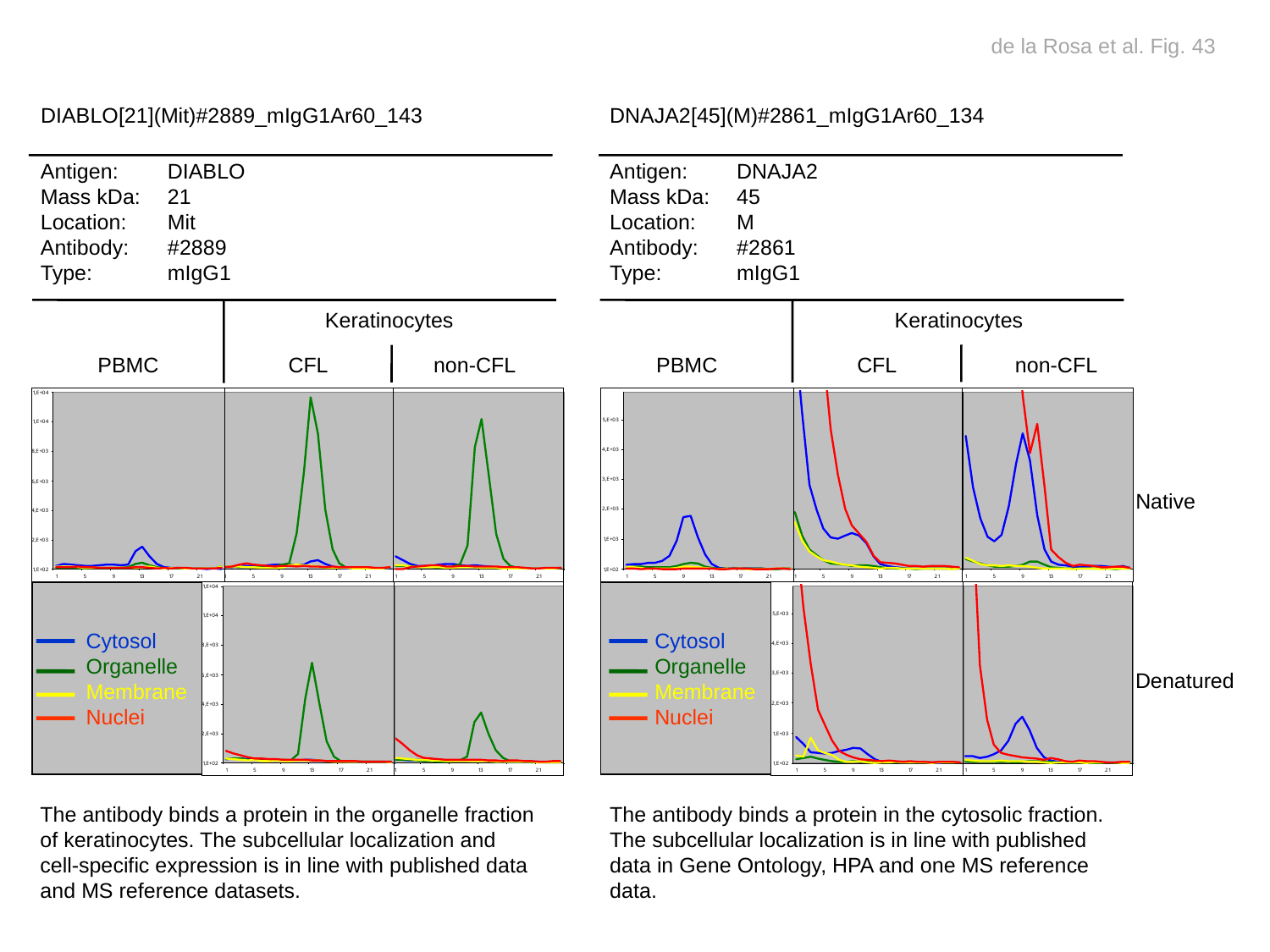

de la Rosa et al. Fig. <number>
# DIABLO[21](Mit)#2889_mIgG1Ar60_143
DNAJA2[45](M)#2861_mIgG1Ar60_134
Antigen: 	DIABLO
Mass kDa:	21
Location: 	Mit
Antibody: 	#2889
Type:	mIgG1
Antigen: 	DNAJA2
Mass kDa:	45
Location: 	M
Antibody: 	#2861
Type:	mIgG1
The antibody binds a protein in the organelle fraction of keratinocytes. The subcellular localization and cell-specific expression is in line with published data and MS reference datasets.
The antibody binds a protein in the cytosolic fraction. The subcellular localization is in line with published data in Gene Ontology, HPA and one MS reference data.

## Slide 44
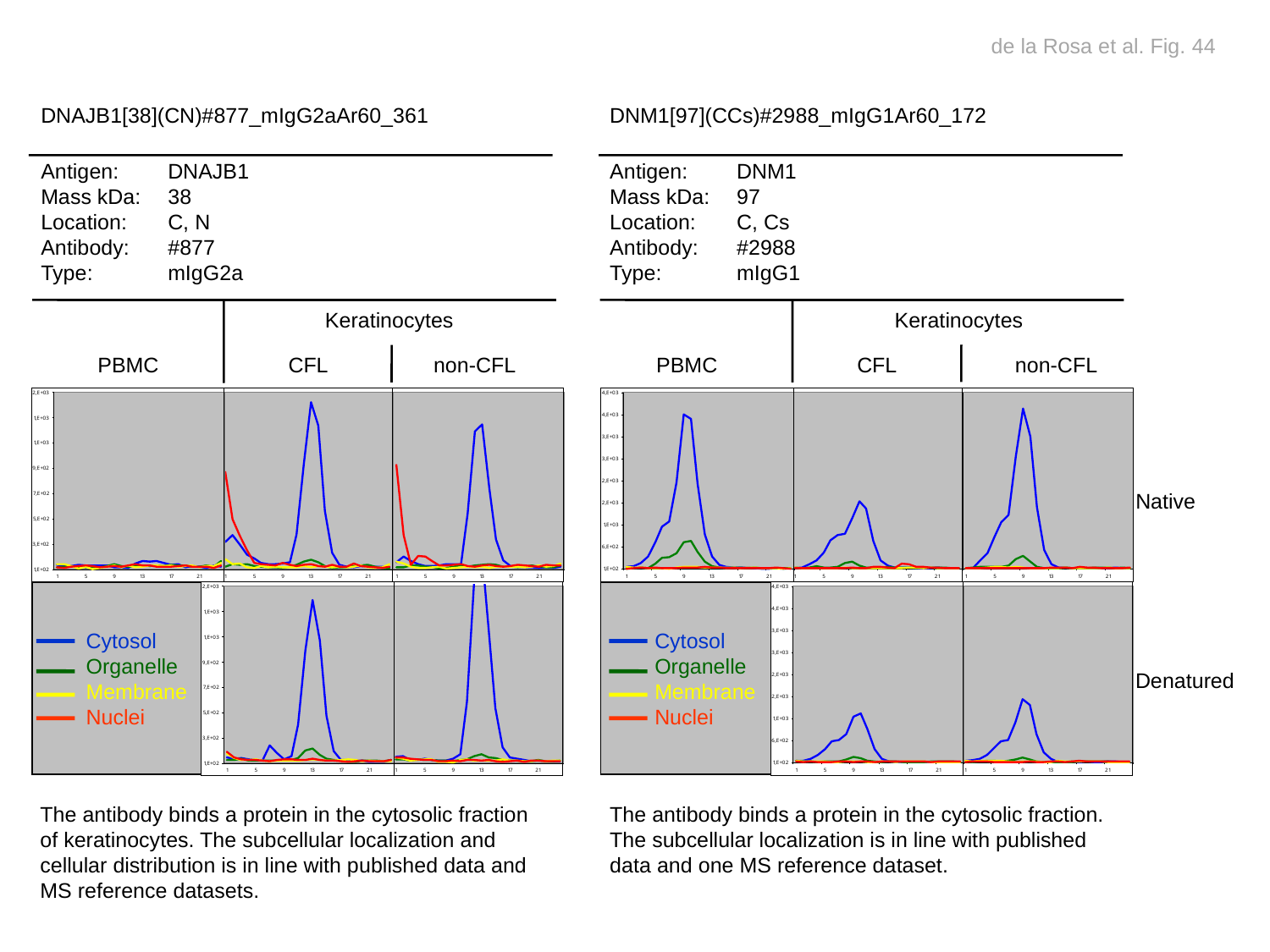

de la Rosa et al. Fig. <number>
# DNAJB1[38](CN)#877_mIgG2aAr60_361
DNM1[97](CCs)#2988_mIgG1Ar60_172
Antigen: 	DNAJB1
Mass kDa:	38
Location: 	C, N
Antibody: 	#877
Type:	mIgG2a
Antigen: 	DNM1
Mass kDa:	97
Location: 	C, Cs
Antibody: 	#2988
Type:	mIgG1
The antibody binds a protein in the cytosolic fraction of keratinocytes. The subcellular localization and cellular distribution is in line with published data and MS reference datasets.
The antibody binds a protein in the cytosolic fraction. The subcellular localization is in line with published data and one MS reference dataset.

## Slide 45
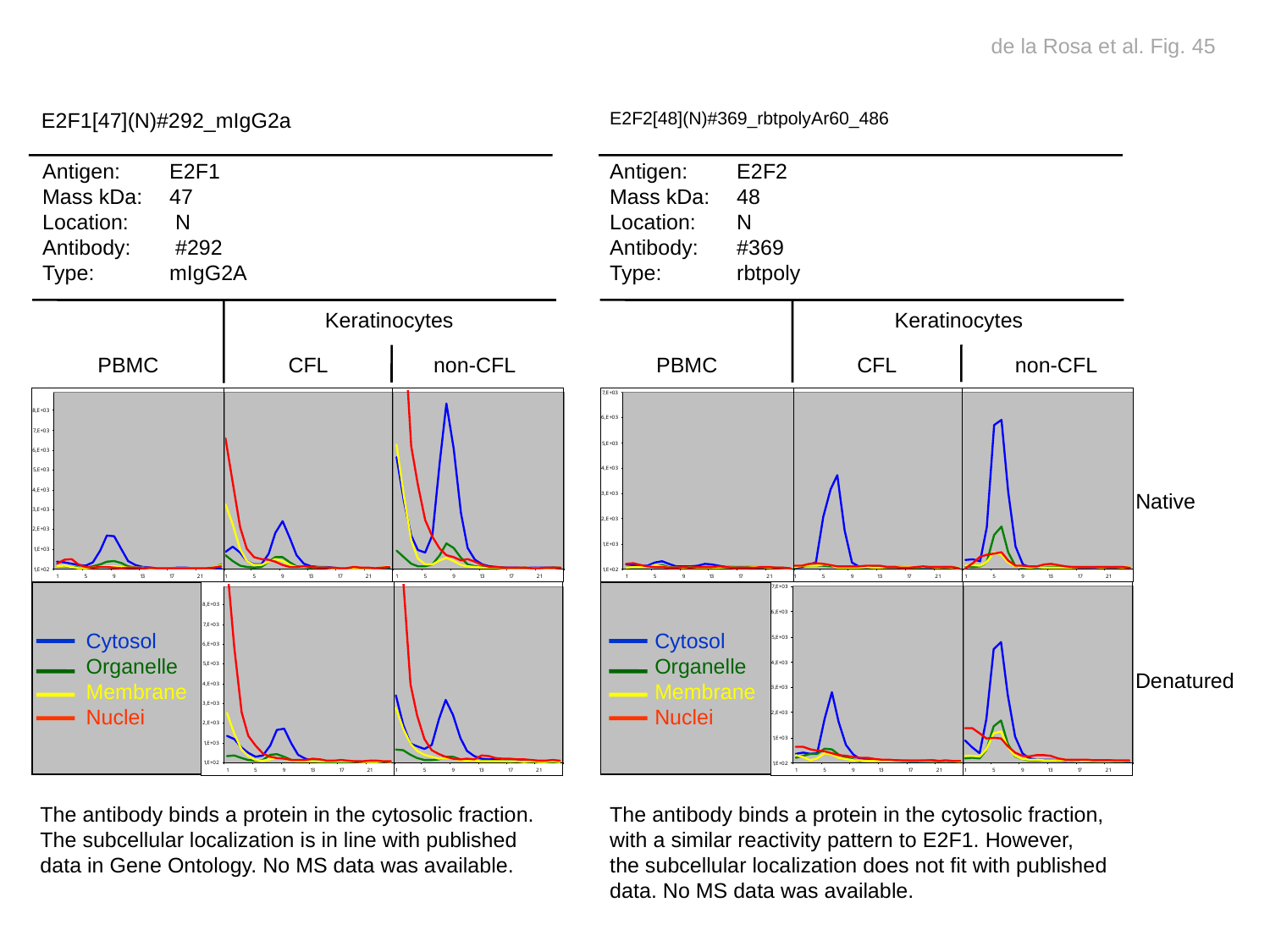

de la Rosa et al. Fig. <number>
# E2F2[48](N)#369_rbtpolyAr60_486
E2F1[47](N)#292_mIgG2a
Antigen: 	E2F1
Mass kDa:	47
Location: 	 N
Antibody: 	 #292
Type:	mIgG2A
Antigen: 	E2F2
Mass kDa:	48
Location: 	N
Antibody: 	#369
Type:	rbtpoly
The antibody binds a protein in the cytosolic fraction. The subcellular localization is in line with published data in Gene Ontology. No MS data was available.
The antibody binds a protein in the cytosolic fraction, with a similar reactivity pattern to E2F1. However, the subcellular localization does not fit with published data. No MS data was available.

## Slide 46
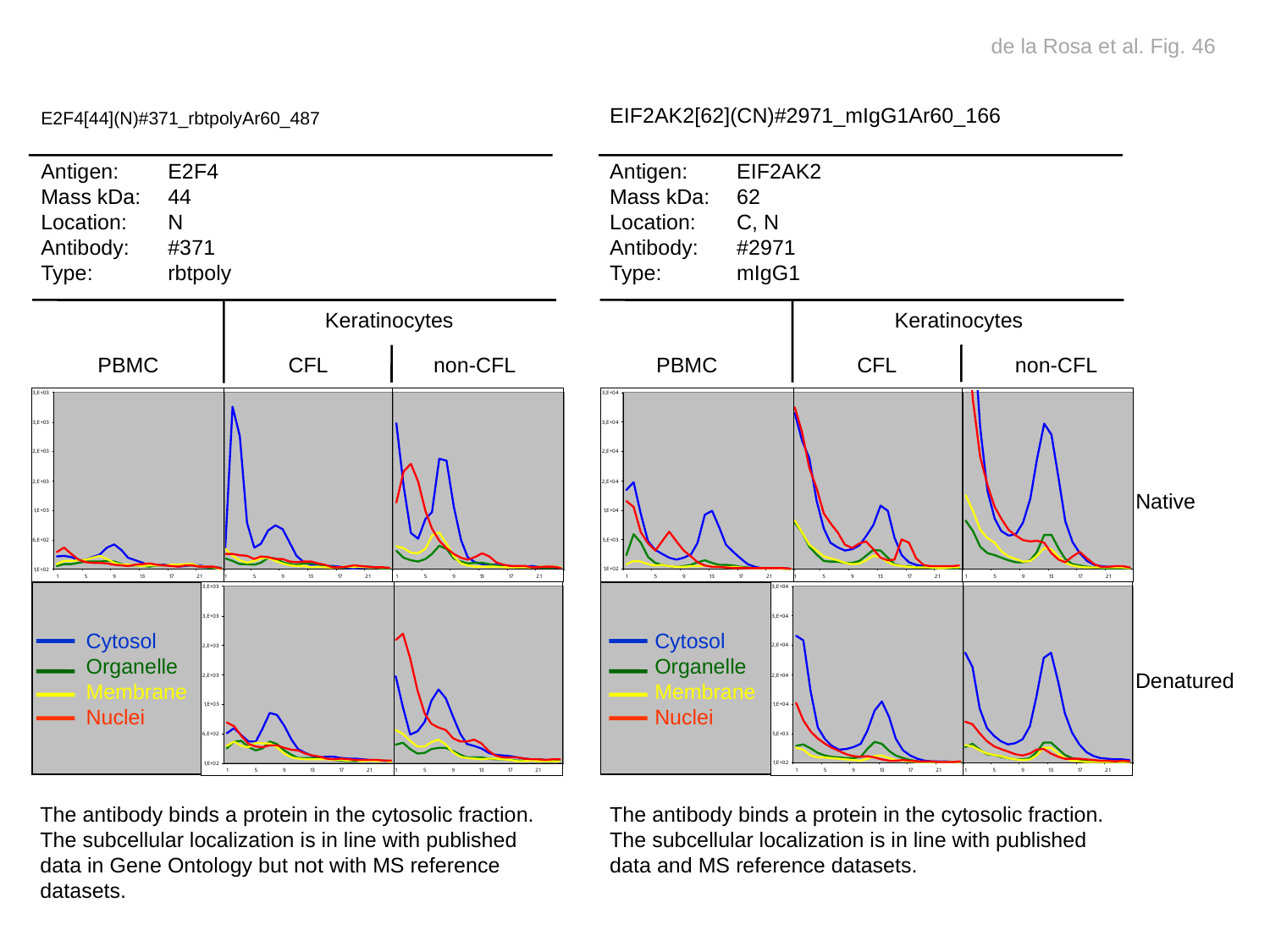

de la Rosa et al. Fig. <number>
# E2F4[44](N)#371_rbtpolyAr60_487
EIF2AK2[62](CN)#2971_mIgG1Ar60_166
Antigen: 	E2F4
Mass kDa:	44
Location: 	N
Antibody: 	#371
Type:	rbtpoly
Antigen: 	EIF2AK2
Mass kDa:	62
Location: 	C, N
Antibody: 	#2971
Type:	mIgG1
The antibody binds a protein in the cytosolic fraction. The subcellular localization is in line with published data in Gene Ontology but not with MS reference datasets.
The antibody binds a protein in the cytosolic fraction. The subcellular localization is in line with published data and MS reference datasets.

## Slide 47
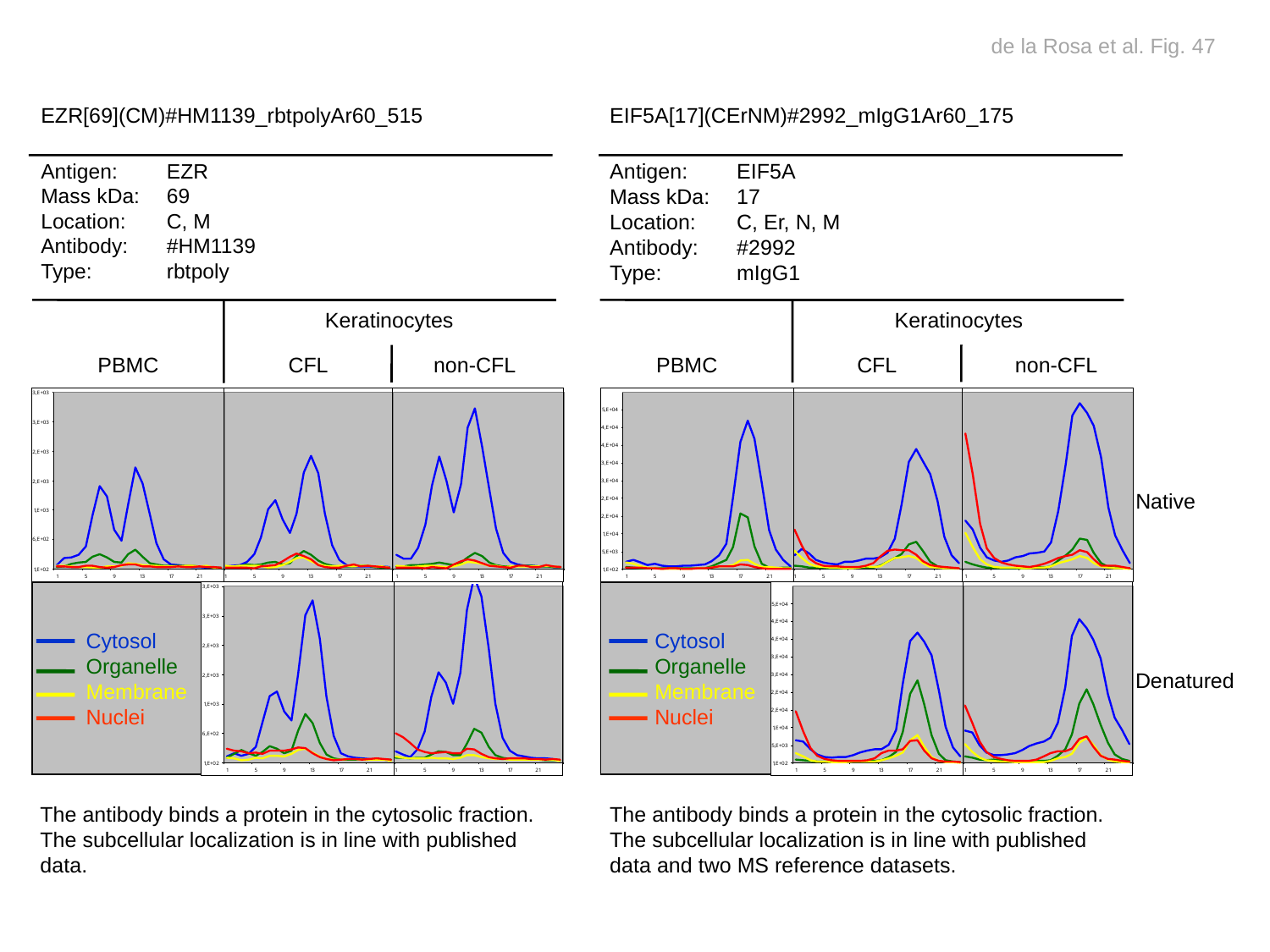

de la Rosa et al. Fig. <number>
EZR[69](CM)#HM1139_rbtpolyAr60_515
# EIF5A[17](CErNM)#2992_mIgG1Ar60_175
Antigen: 	EZR
Mass kDa:	69
Location: 	C, M
Antibody: 	#HM1139
Type:	rbtpoly
Antigen: 	EIF5A
Mass kDa:	17
Location: 	C, Er, N, M
Antibody: 	#2992
Type:	mIgG1
The antibody binds a protein in the cytosolic fraction. The subcellular localization is in line with published data.
The antibody binds a protein in the cytosolic fraction. The subcellular localization is in line with published data and two MS reference datasets.

## Slide 48
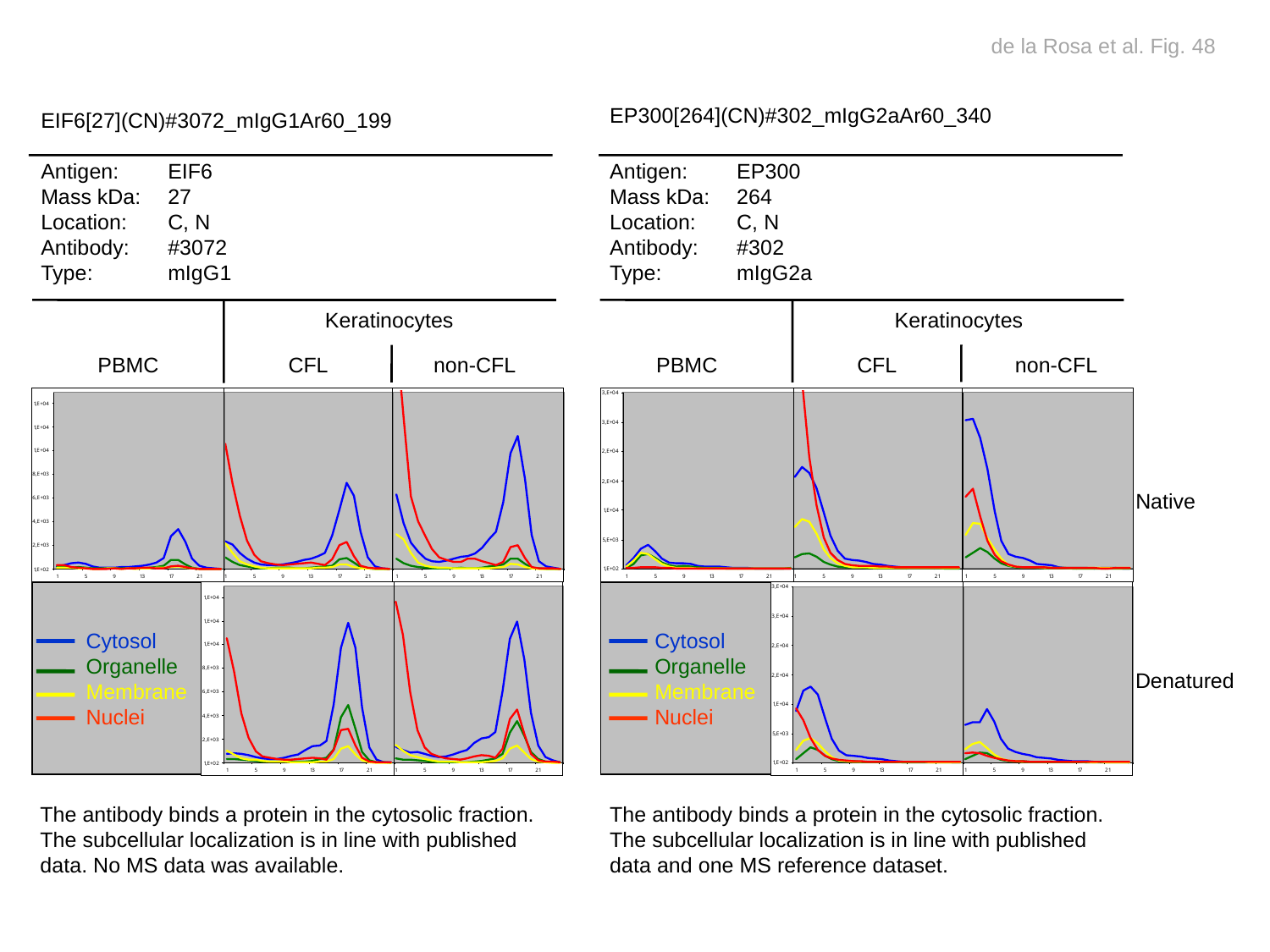

de la Rosa et al. Fig. <number>
# EIF6[27](CN)#3072_mIgG1Ar60_199
EP300[264](CN)#302_mIgG2aAr60_340
Antigen: 	EIF6
Mass kDa:	27
Location: 	C, N
Antibody: 	#3072
Type:	mIgG1
Antigen: 	EP300
Mass kDa:	264
Location: 	C, N
Antibody: 	#302
Type:	mIgG2a
The antibody binds a protein in the cytosolic fraction. The subcellular localization is in line with published data. No MS data was available.
The antibody binds a protein in the cytosolic fraction. The subcellular localization is in line with published data and one MS reference dataset.

## Slide 49
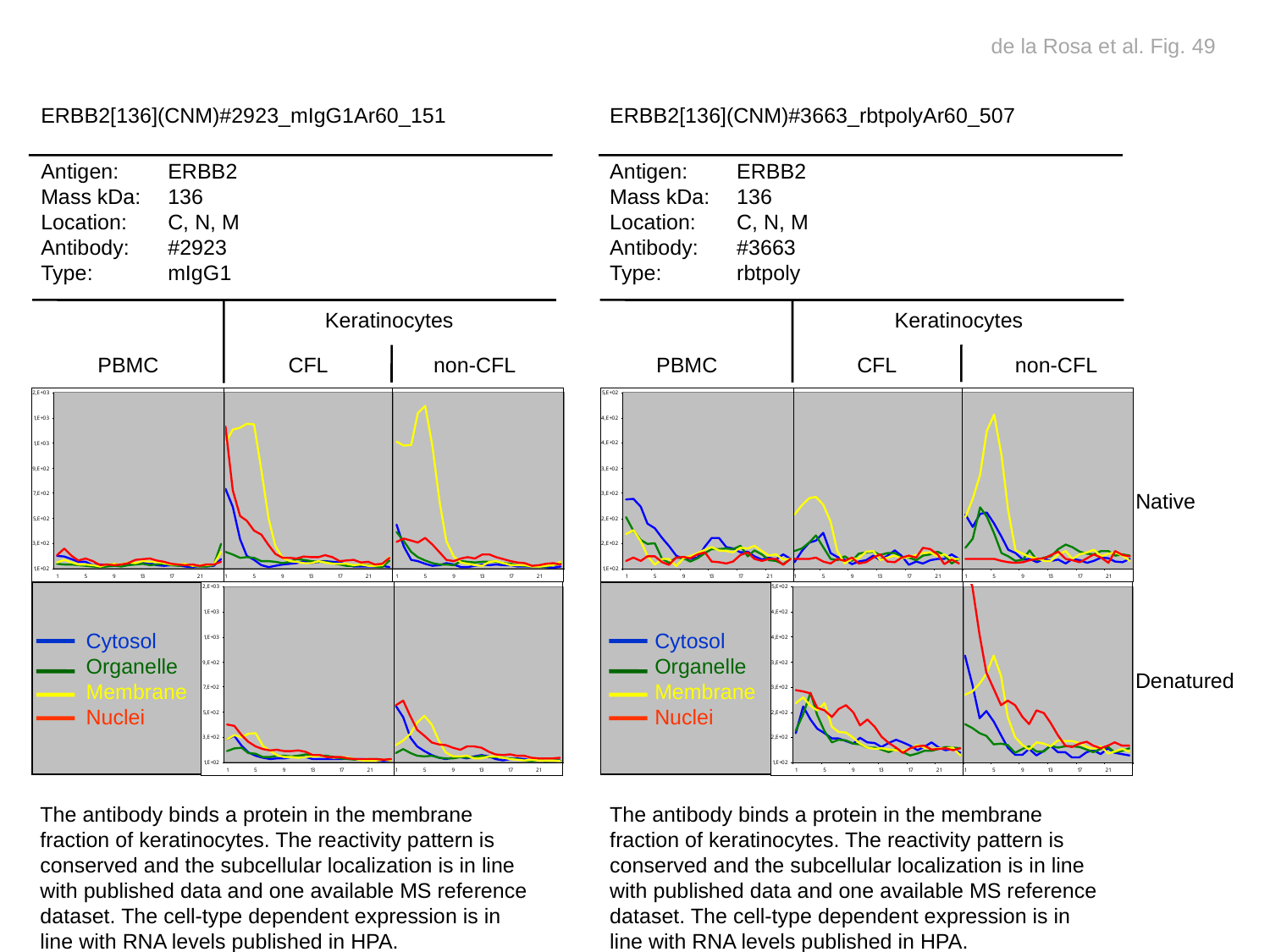

de la Rosa et al. Fig. <number>
# ERBB2[136](CNM)#2923_mIgG1Ar60_151
ERBB2[136](CNM)#3663_rbtpolyAr60_507
Antigen: 	ERBB2
Mass kDa:	136
Location: 	C, N, M
Antibody: 	#2923
Type:	mIgG1
Antigen: 	ERBB2
Mass kDa:	136
Location: 	C, N, M
Antibody: 	#3663
Type:	rbtpoly
The antibody binds a protein in the membrane fraction of keratinocytes. The reactivity pattern is conserved and the subcellular localization is in line with published data and one available MS reference dataset. The cell-type dependent expression is in line with RNA levels published in HPA.
The antibody binds a protein in the membrane fraction of keratinocytes. The reactivity pattern is conserved and the subcellular localization is in line with published data and one available MS reference dataset. The cell-type dependent expression is in line with RNA levels published in HPA.

## Slide 50
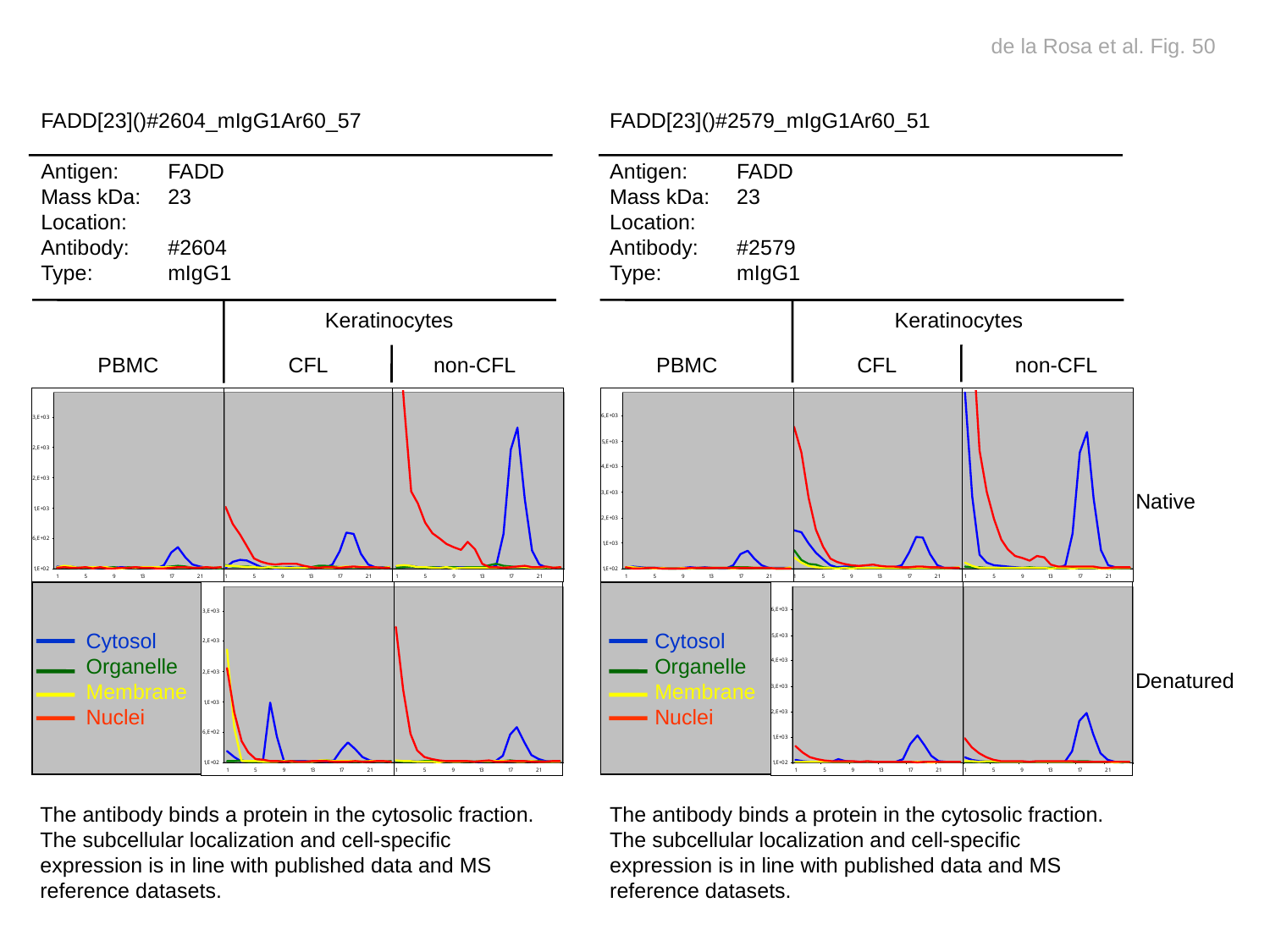

de la Rosa et al. Fig. <number>
FADD[23]()#2604_mIgG1Ar60_57
# FADD[23]()#2579_mIgG1Ar60_51
Antigen: 	FADD
Mass kDa:	23
Location:
Antibody: 	#2604
Type:	mIgG1
Antigen: 	FADD
Mass kDa:	23
Location:
Antibody: 	#2579
Type:	mIgG1
The antibody binds a protein in the cytosolic fraction. The subcellular localization and cell-specific expression is in line with published data and MS reference datasets.
The antibody binds a protein in the cytosolic fraction. The subcellular localization and cell-specific expression is in line with published data and MS reference datasets.

## Slide 51
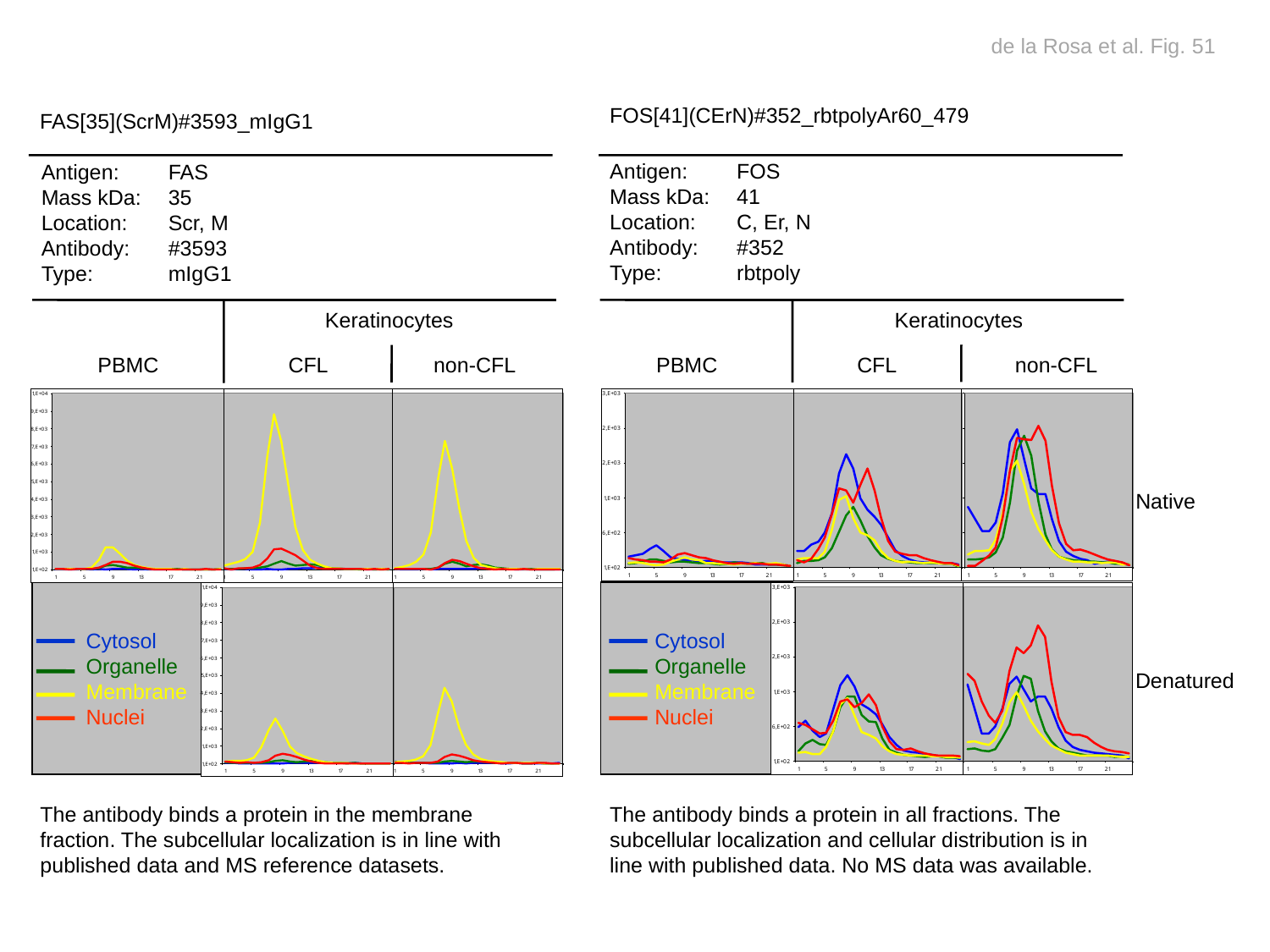

de la Rosa et al. Fig. <number>
# FOS[41](CErN)#352_rbtpolyAr60_479
FAS[35](ScrM)#3593_mIgG1
Antigen: 	FOS
Mass kDa:	41
Location: 	C, Er, N
Antibody: 	#352
Type:	rbtpoly
Antigen:	FAS
Mass kDa:	35
Location: 	Scr, M
Antibody: 	#3593
Type:	mIgG1
The antibody binds a protein in the membrane fraction. The subcellular localization is in line with published data and MS reference datasets.
The antibody binds a protein in all fractions. The subcellular localization and cellular distribution is in line with published data. No MS data was available.

## Slide 52
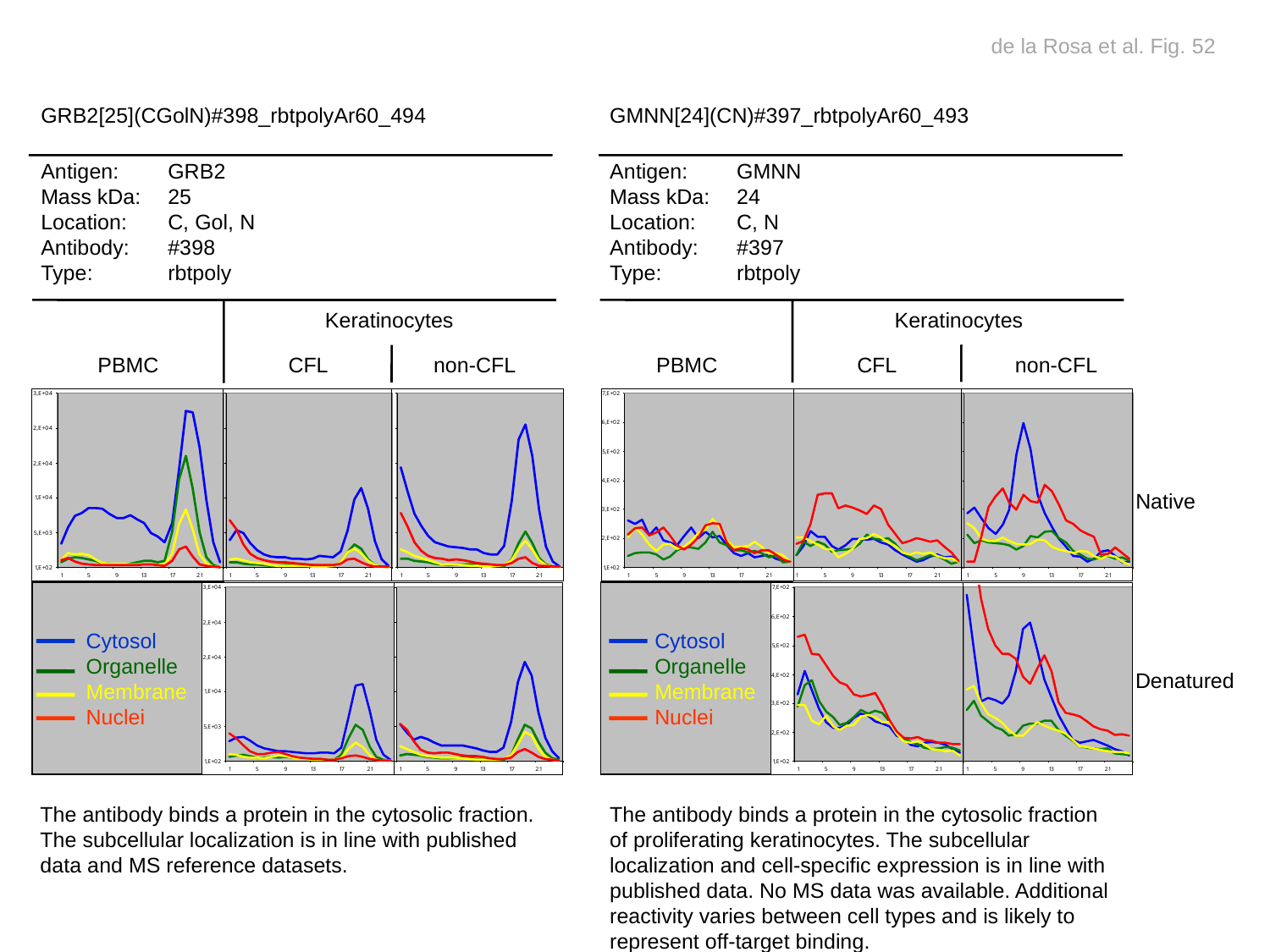

de la Rosa et al. Fig. <number>
GRB2[25](CGolN)#398_rbtpolyAr60_494
# GMNN[24](CN)#397_rbtpolyAr60_493
Antigen: 	GRB2
Mass kDa:	25
Location: 	C, Gol, N
Antibody: 	#398
Type:	rbtpoly
Antigen: 	GMNN
Mass kDa:	24
Location: 	C, N
Antibody: 	#397
Type:	rbtpoly
The antibody binds a protein in the cytosolic fraction. The subcellular localization is in line with published data and MS reference datasets.
The antibody binds a protein in the cytosolic fraction of proliferating keratinocytes. The subcellular localization and cell-specific expression is in line with published data. No MS data was available. Additional reactivity varies between cell types and is likely to represent off-target binding.

## Slide 53
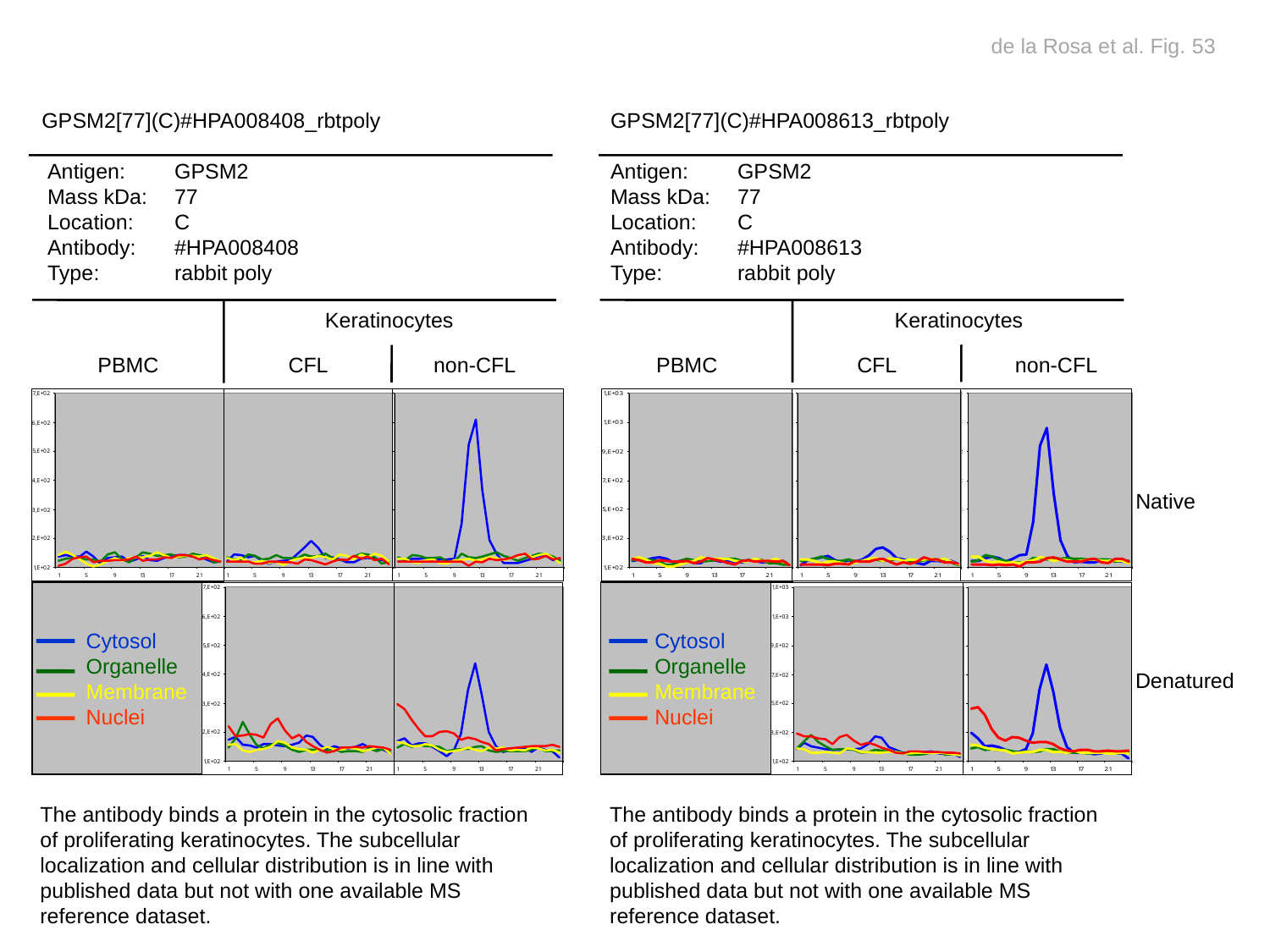

de la Rosa et al. Fig. <number>
GPSM2[77](C)#HPA008408_rbtpoly
GPSM2[77](C)#HPA008613_rbtpoly
Antigen: 	GPSM2
Mass kDa:	77
Location: 	C
Antibody: 	#HPA008408
Type:	rabbit poly
Antigen:	GPSM2
Mass kDa:	77
Location: 	C
Antibody: 	#HPA008613
Type:	rabbit poly
The antibody binds a protein in the cytosolic fraction of proliferating keratinocytes. The subcellular localization and cellular distribution is in line with published data but not with one available MS reference dataset.
The antibody binds a protein in the cytosolic fraction of proliferating keratinocytes. The subcellular localization and cellular distribution is in line with published data but not with one available MS reference dataset.

## Slide 54
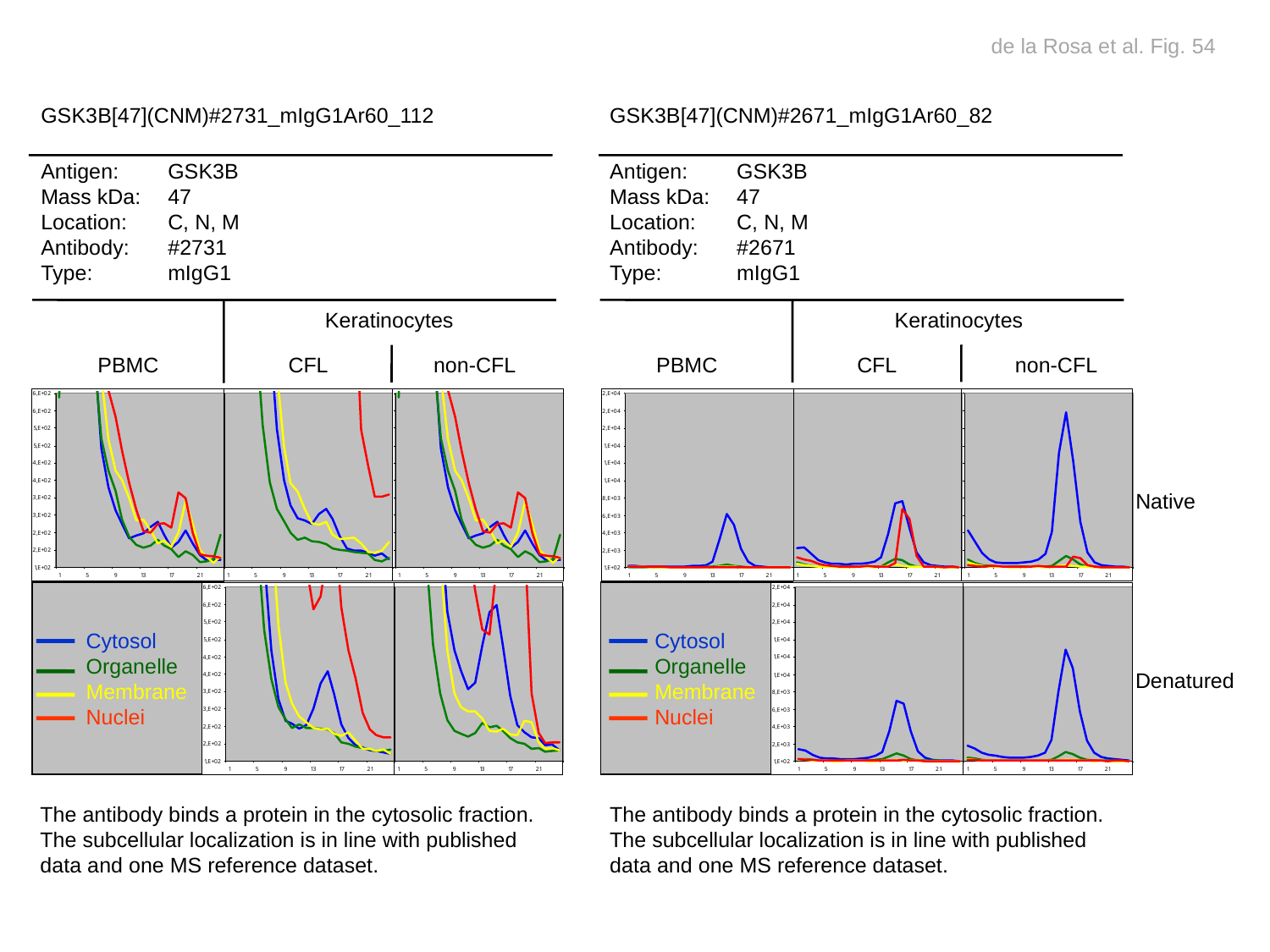

de la Rosa et al. Fig. <number>
# GSK3B[47](CNM)#2731_mIgG1Ar60_112
GSK3B[47](CNM)#2671_mIgG1Ar60_82
Antigen: 	GSK3B
Mass kDa:	47
Location: 	C, N, M
Antibody: 	#2731
Type:	mIgG1
Antigen: 	GSK3B
Mass kDa:	47
Location: 	C, N, M
Antibody: 	#2671
Type:	mIgG1
The antibody binds a protein in the cytosolic fraction. The subcellular localization is in line with published data and one MS reference dataset.
The antibody binds a protein in the cytosolic fraction. The subcellular localization is in line with published data and one MS reference dataset.

## Slide 55
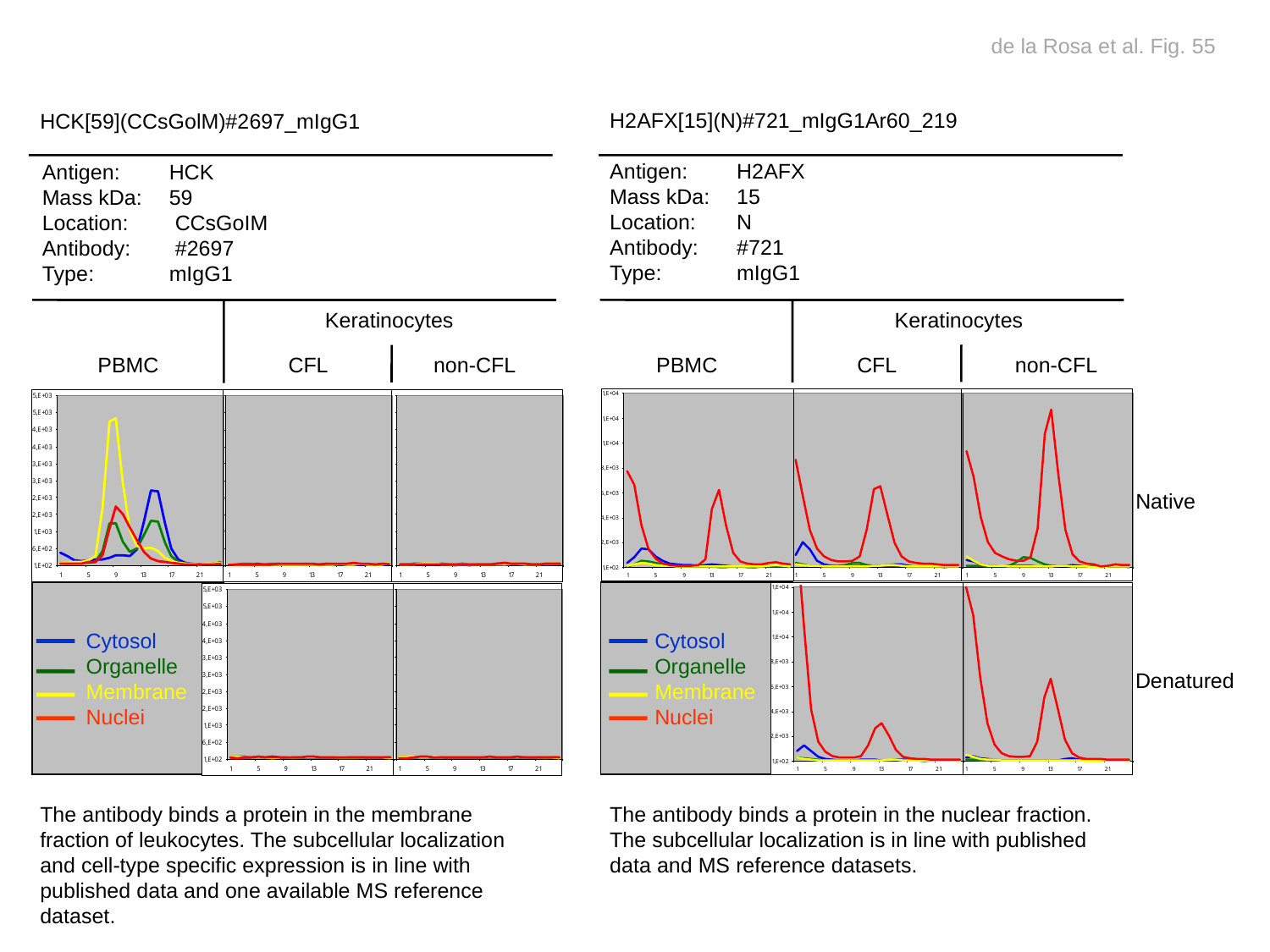

de la Rosa et al. Fig. <number>
# H2AFX[15](N)#721_mIgG1Ar60_219
HCK[59](CCsGolM)#2697_mIgG1
Antigen: 	H2AFX
Mass kDa:	15
Location: 	N
Antibody: 	#721
Type:	mIgG1
Antigen: 	HCK
Mass kDa:	59
Location: 	 CCsGoIM
Antibody: 	 #2697
Type:	mIgG1
The antibody binds a protein in the membrane fraction of leukocytes. The subcellular localization and cell-type specific expression is in line with published data and one available MS reference dataset.
The antibody binds a protein in the nuclear fraction. The subcellular localization is in line with published data and MS reference datasets.

## Slide 56
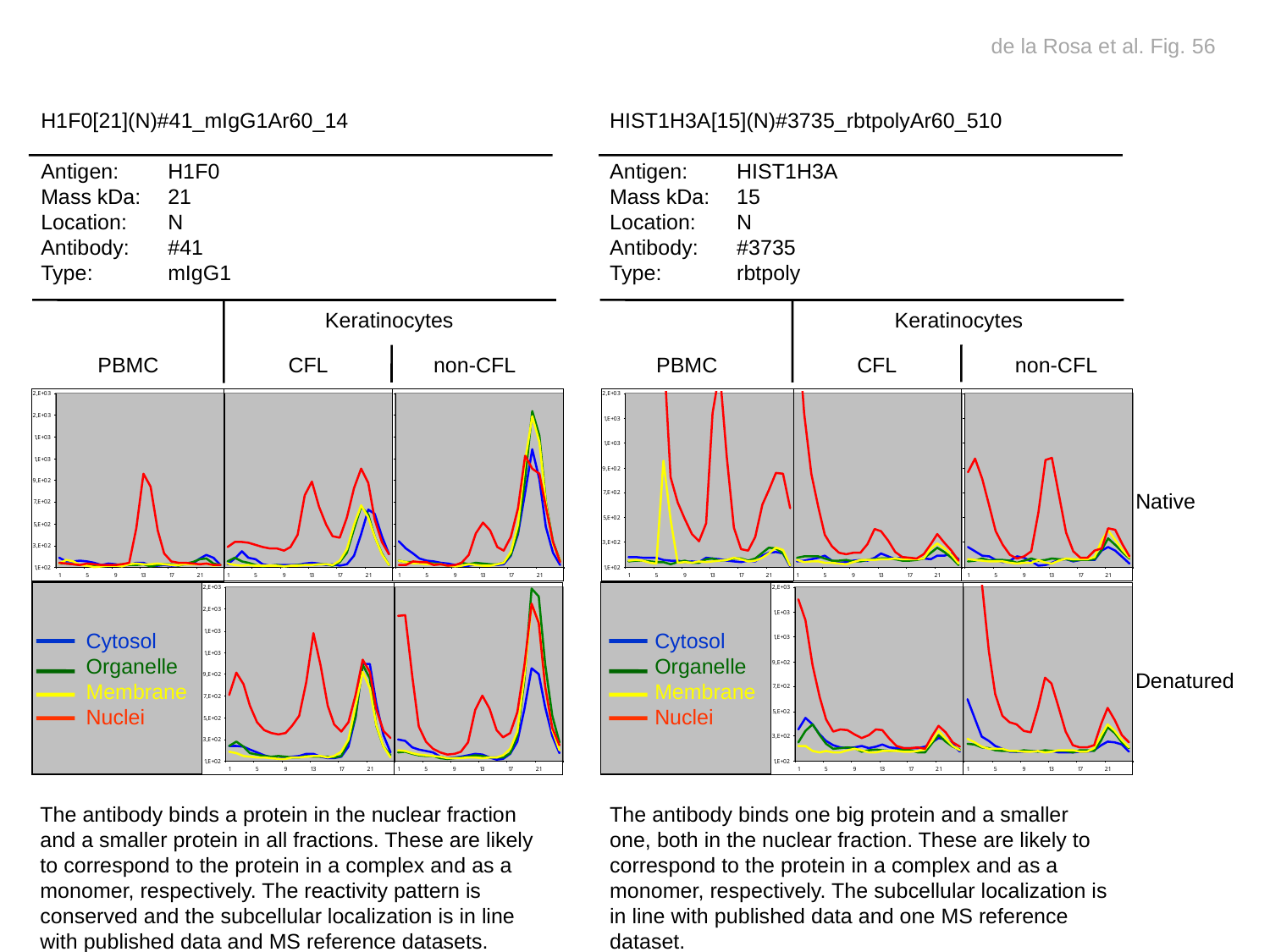

de la Rosa et al. Fig. <number>
H1F0[21](N)#41_mIgG1Ar60_14
# HIST1H3A[15](N)#3735_rbtpolyAr60_510
Antigen: 	H1F0
Mass kDa:	21
Location: 	N
Antibody: 	#41
Type:	mIgG1
Antigen: 	HIST1H3A
Mass kDa:	15
Location: 	N
Antibody: 	#3735
Type:	rbtpoly
The antibody binds a protein in the nuclear fraction and a smaller protein in all fractions. These are likely to correspond to the protein in a complex and as a monomer, respectively. The reactivity pattern is conserved and the subcellular localization is in line with published data and MS reference datasets.
The antibody binds one big protein and a smaller one, both in the nuclear fraction. These are likely to correspond to the protein in a complex and as a monomer, respectively. The subcellular localization is in line with published data and one MS reference dataset.

## Slide 57
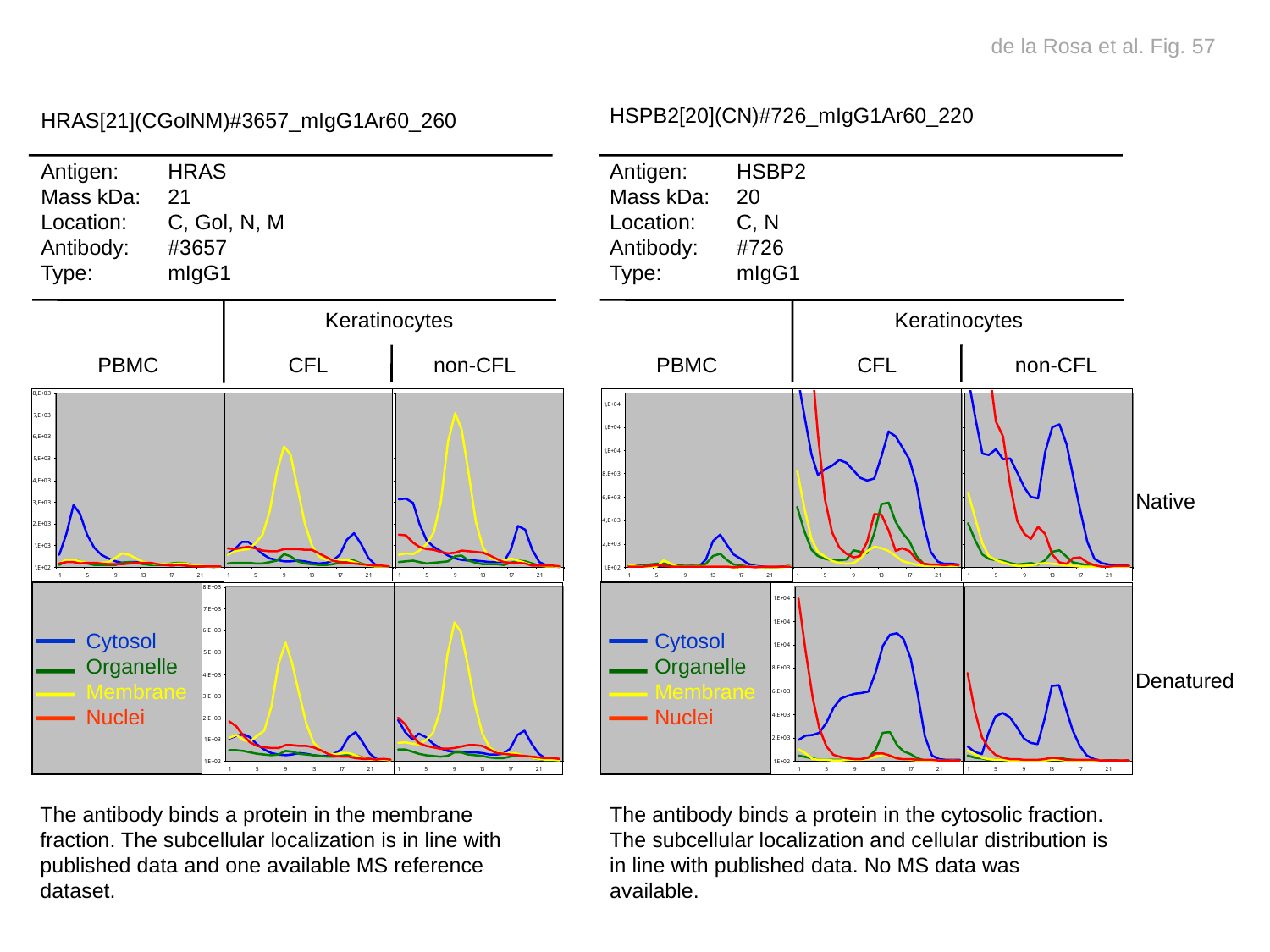

de la Rosa et al. Fig. <number>
# HRAS[21](CGolNM)#3657_mIgG1Ar60_260
HSPB2[20](CN)#726_mIgG1Ar60_220
Antigen: 	HRAS
Mass kDa:	21
Location: 	C, Gol, N, M
Antibody: 	#3657
Type:	mIgG1
Antigen: 	HSBP2
Mass kDa:	20
Location: 	C, N
Antibody: 	#726
Type:	mIgG1
The antibody binds a protein in the membrane fraction. The subcellular localization is in line with published data and one available MS reference dataset.
The antibody binds a protein in the cytosolic fraction. The subcellular localization and cellular distribution is in line with published data. No MS data was available.

## Slide 58
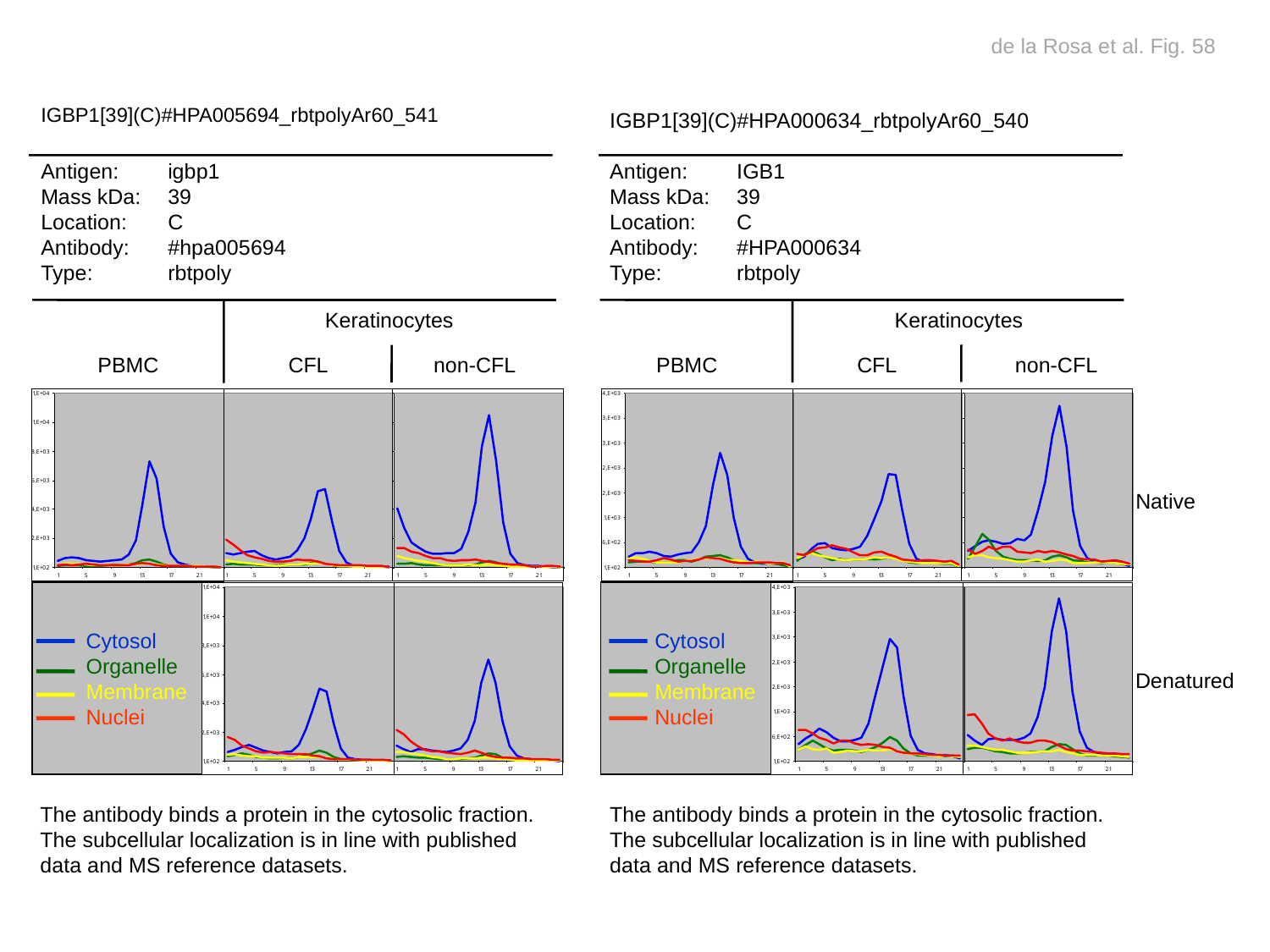

de la Rosa et al. Fig. <number>
IGBP1[39](C)#HPA005694_rbtpolyAr60_541
# IGBP1[39](C)#HPA000634_rbtpolyAr60_540
Antigen: 	igbp1
Mass kDa:	39
Location: 	C
Antibody: 	#hpa005694
Type:	rbtpoly
Antigen: 	IGB1
Mass kDa:	39
Location: 	C
Antibody: 	#HPA000634
Type:	rbtpoly
The antibody binds a protein in the cytosolic fraction. The subcellular localization is in line with published data and MS reference datasets.
The antibody binds a protein in the cytosolic fraction. The subcellular localization is in line with published data and MS reference datasets.

## Slide 59
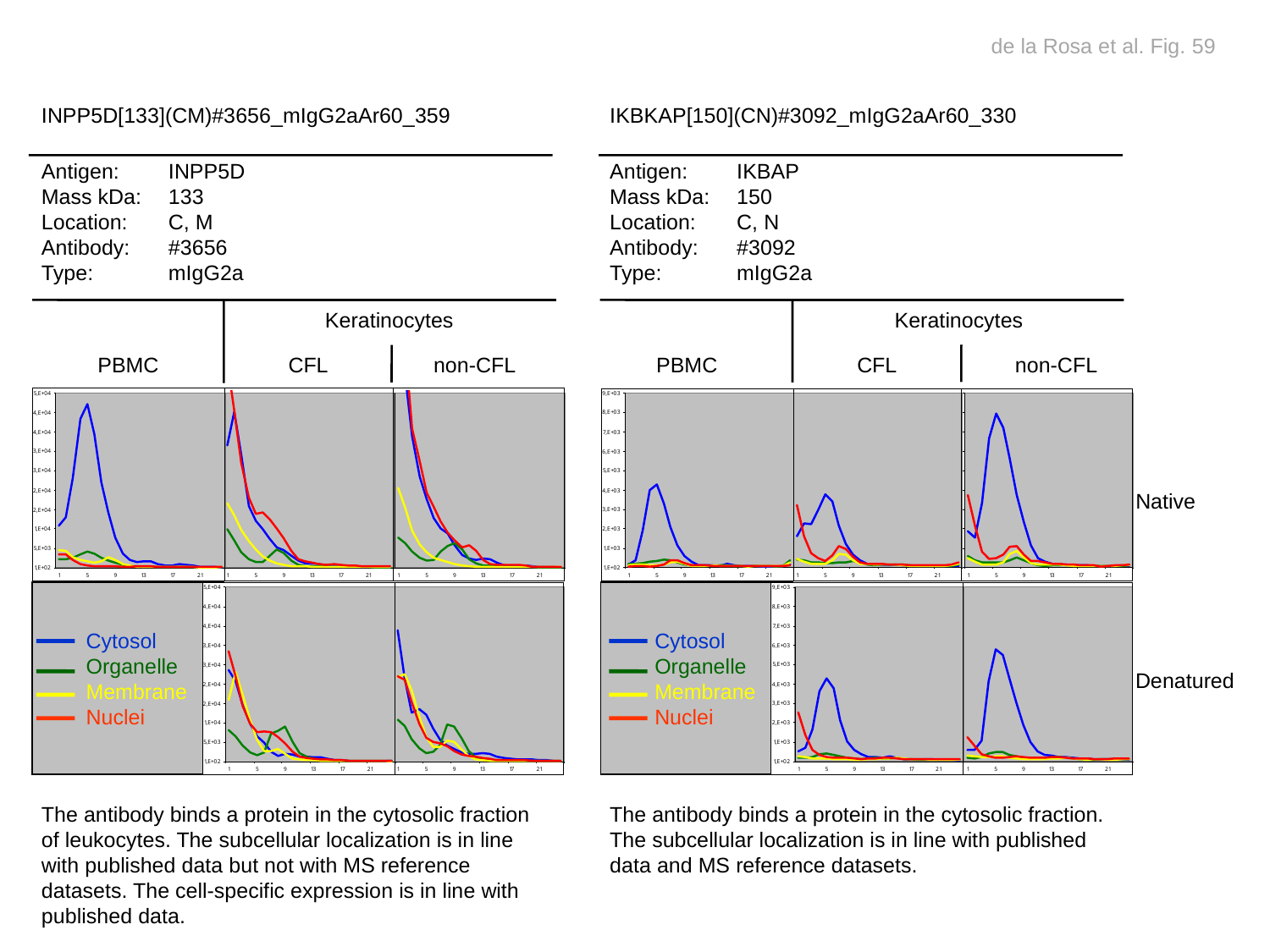

de la Rosa et al. Fig. <number>
INPP5D[133](CM)#3656_mIgG2aAr60_359
# IKBKAP[150](CN)#3092_mIgG2aAr60_330
Antigen: 	INPP5D
Mass kDa:	133
Location: 	C, M
Antibody: 	#3656
Type:	mIgG2a
Antigen: 	IKBAP
Mass kDa:	150
Location: 	C, N
Antibody: 	#3092
Type:	mIgG2a
The antibody binds a protein in the cytosolic fraction of leukocytes. The subcellular localization is in line with published data but not with MS reference datasets. The cell-specific expression is in line with published data.
The antibody binds a protein in the cytosolic fraction. The subcellular localization is in line with published data and MS reference datasets.

## Slide 60
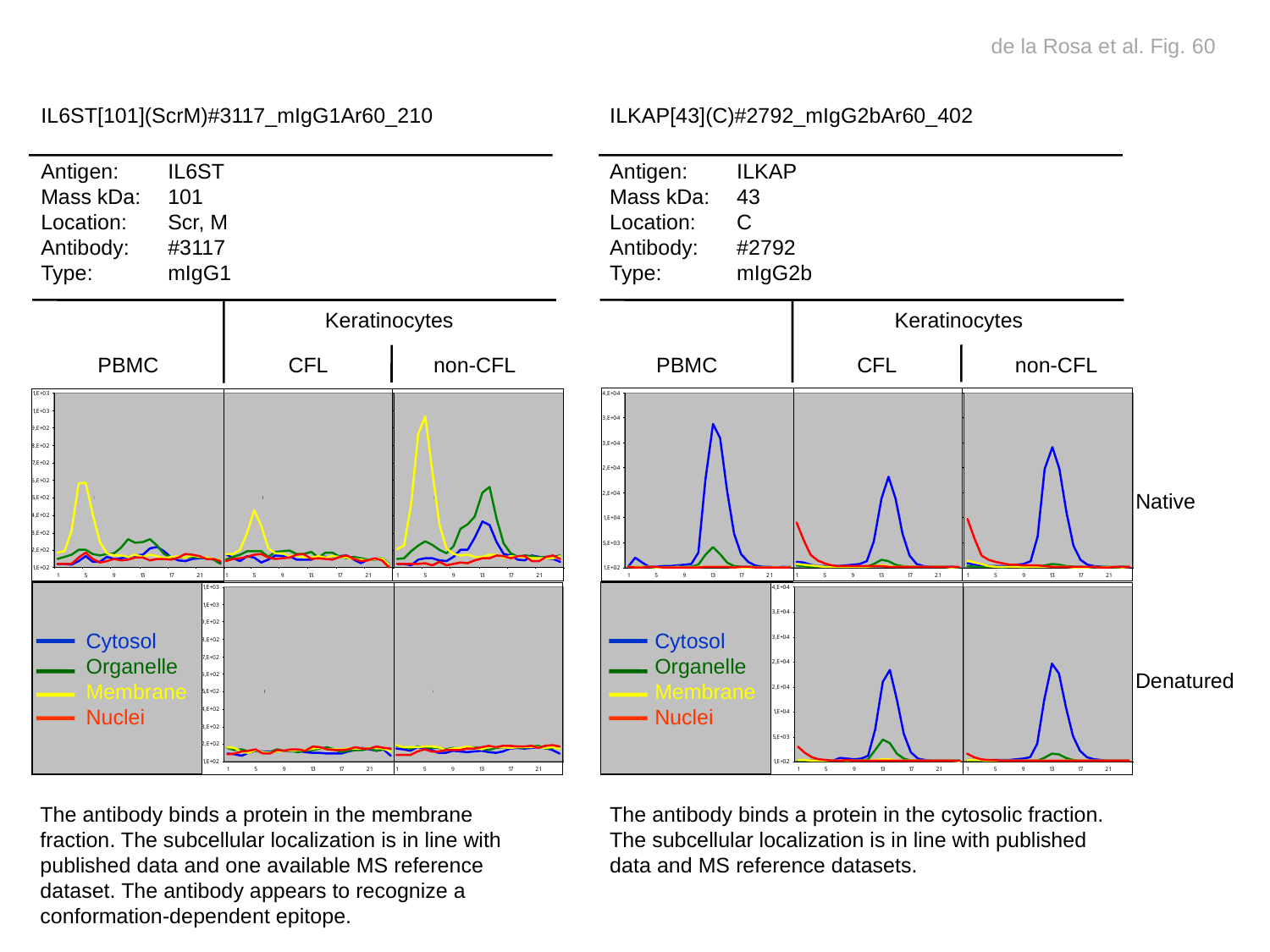

de la Rosa et al. Fig. <number>
# IL6ST[101](ScrM)#3117_mIgG1Ar60_210
ILKAP[43](C)#2792_mIgG2bAr60_402
Antigen: 	IL6ST
Mass kDa:	101
Location: 	Scr, M
Antibody: 	#3117
Type:	mIgG1
Antigen: 	ILKAP
Mass kDa:	43
Location: 	C
Antibody: 	#2792
Type:	mIgG2b
The antibody binds a protein in the membrane fraction. The subcellular localization is in line with published data and one available MS reference dataset. The antibody appears to recognize a conformation-dependent epitope.
The antibody binds a protein in the cytosolic fraction. The subcellular localization is in line with published data and MS reference datasets.

## Slide 61
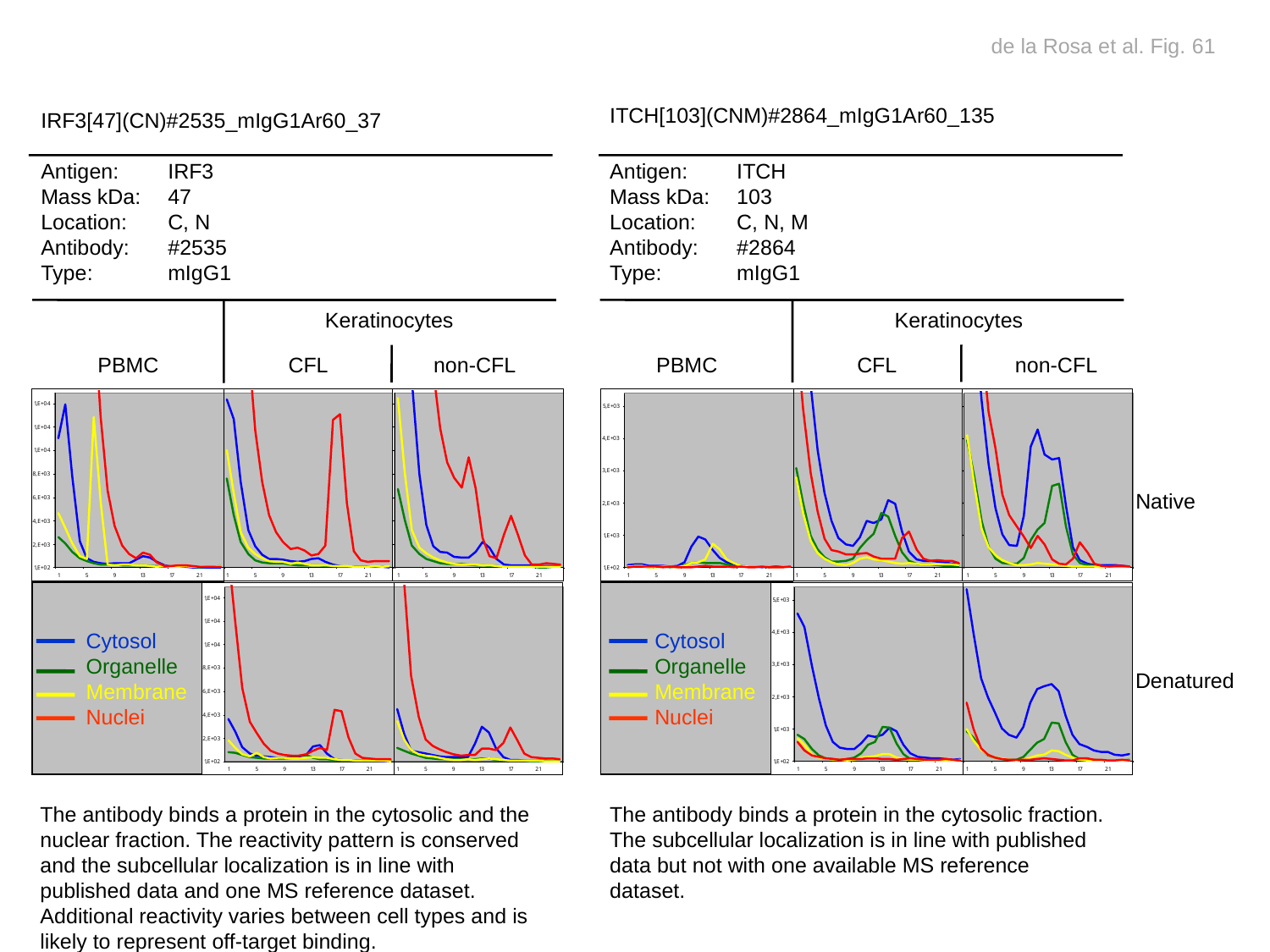

de la Rosa et al. Fig. <number>
# IRF3[47](CN)#2535_mIgG1Ar60_37
ITCH[103](CNM)#2864_mIgG1Ar60_135
Antigen: 	IRF3
Mass kDa:	47
Location: 	C, N
Antibody: 	#2535
Type:	mIgG1
Antigen: 	ITCH
Mass kDa:	103
Location: 	C, N, M
Antibody: 	#2864
Type:	mIgG1
The antibody binds a protein in the cytosolic and the nuclear fraction. The reactivity pattern is conserved and the subcellular localization is in line with published data and one MS reference dataset. Additional reactivity varies between cell types and is likely to represent off-target binding.
The antibody binds a protein in the cytosolic fraction. The subcellular localization is in line with published data but not with one available MS reference dataset.

## Slide 62
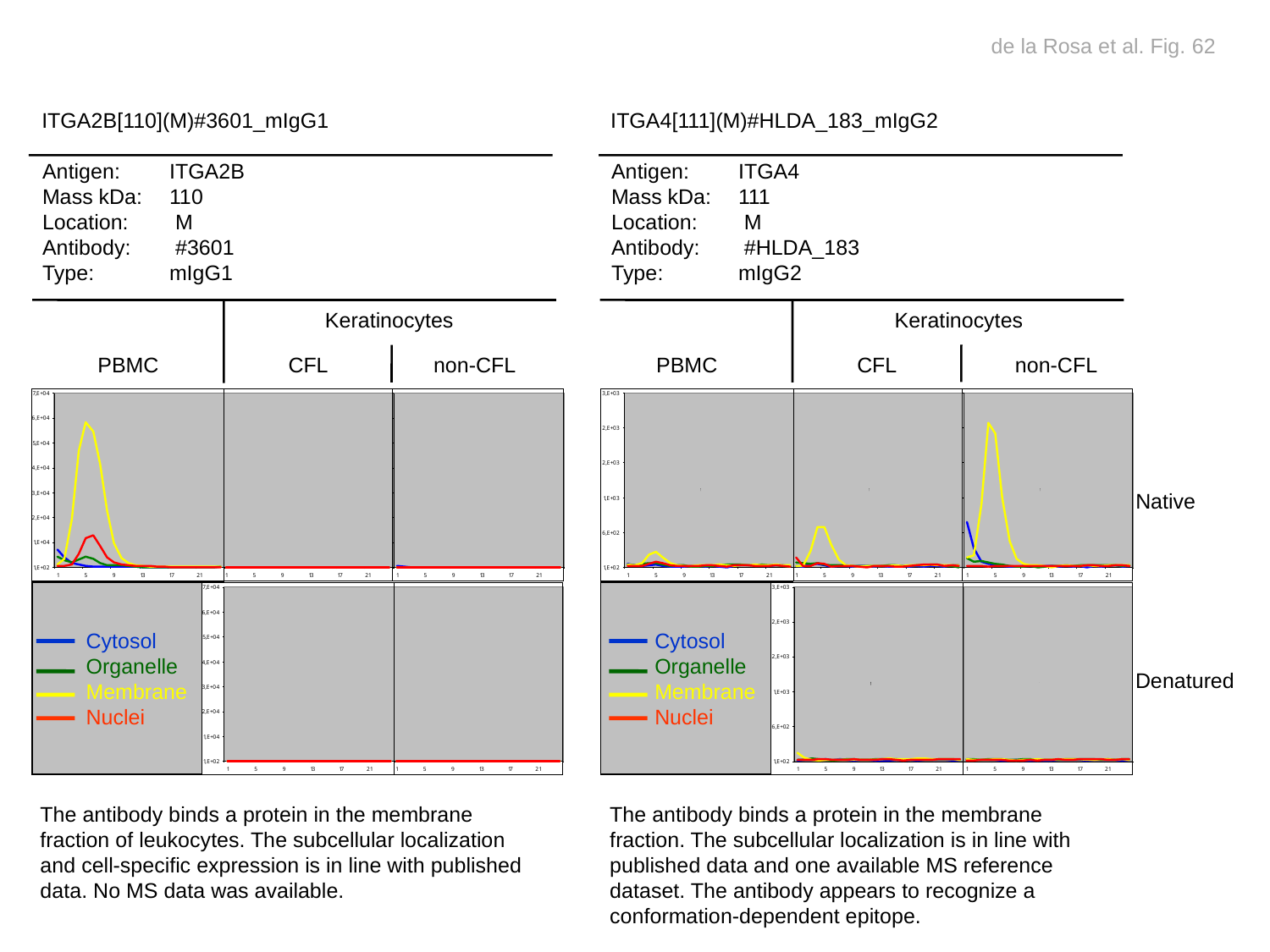

de la Rosa et al. Fig. <number>
ITGA2B[110](M)#3601_mIgG1
ITGA4[111](M)#HLDA_183_mIgG2
Antigen: 	ITGA2B
Mass kDa:	110
Location: 	 M
Antibody: 	 #3601
Type:	mIgG1
Antigen: 	ITGA4
Mass kDa:	111
Location: 	 M
Antibody: 	 #HLDA_183
Type:	mIgG2
The antibody binds a protein in the membrane fraction of leukocytes. The subcellular localization and cell-specific expression is in line with published data. No MS data was available.
The antibody binds a protein in the membrane fraction. The subcellular localization is in line with published data and one available MS reference dataset. The antibody appears to recognize a conformation-dependent epitope.

## Slide 63
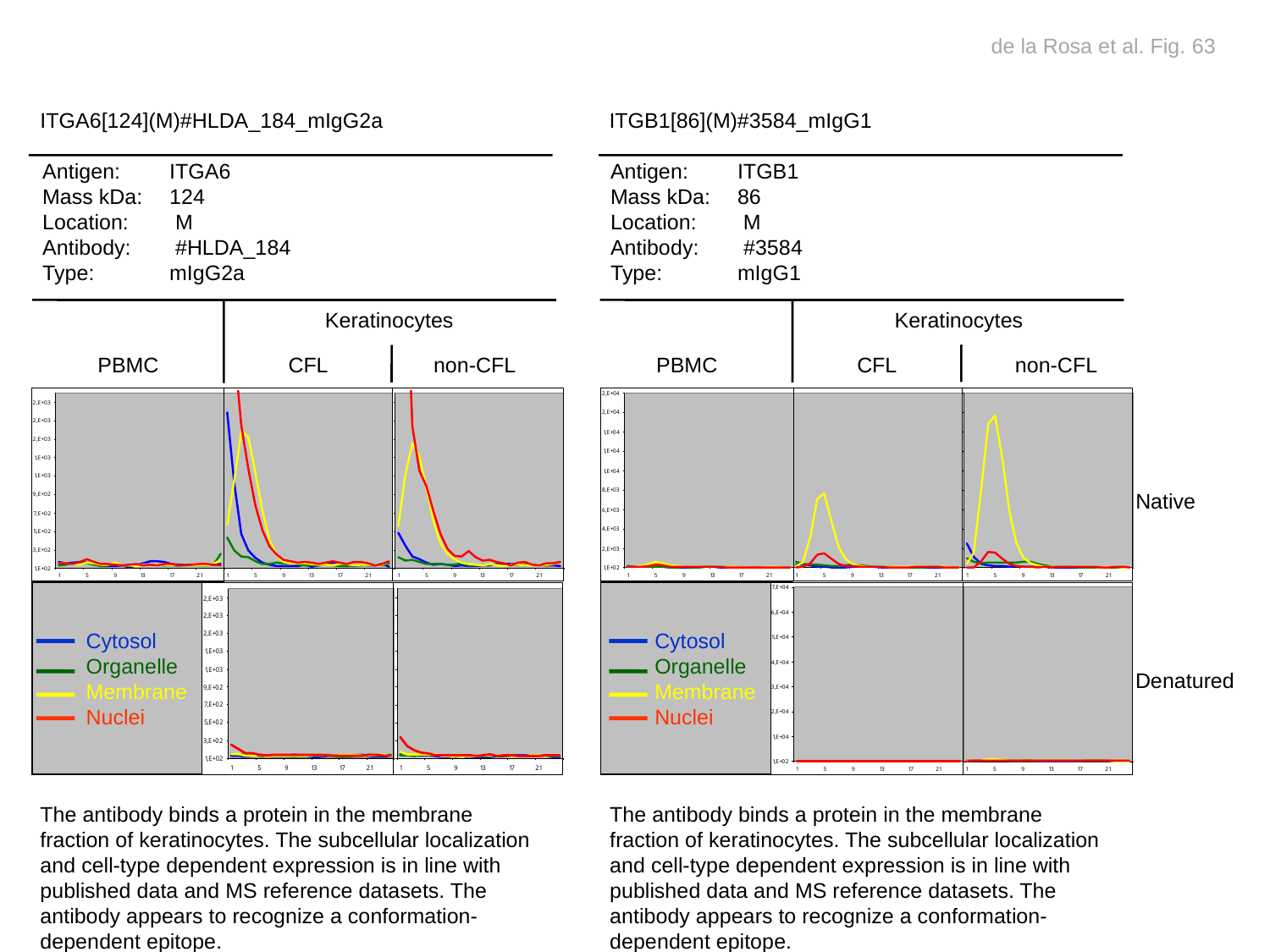

de la Rosa et al. Fig. <number>
ITGA6[124](M)#HLDA_184_mIgG2a
ITGB1[86](M)#3584_mIgG1
Antigen: 	ITGA6
Mass kDa:	124
Location: 	 M
Antibody: 	 #HLDA_184
Type:	mIgG2a
Antigen:	ITGB1
Mass kDa:	86
Location: 	 M
Antibody: 	 #3584
Type:	mIgG1
The antibody binds a protein in the membrane fraction of keratinocytes. The subcellular localization and cell-type dependent expression is in line with published data and MS reference datasets. The antibody appears to recognize a conformation-dependent epitope.
The antibody binds a protein in the membrane fraction of keratinocytes. The subcellular localization and cell-type dependent expression is in line with published data and MS reference datasets. The antibody appears to recognize a conformation-dependent epitope.

## Slide 64
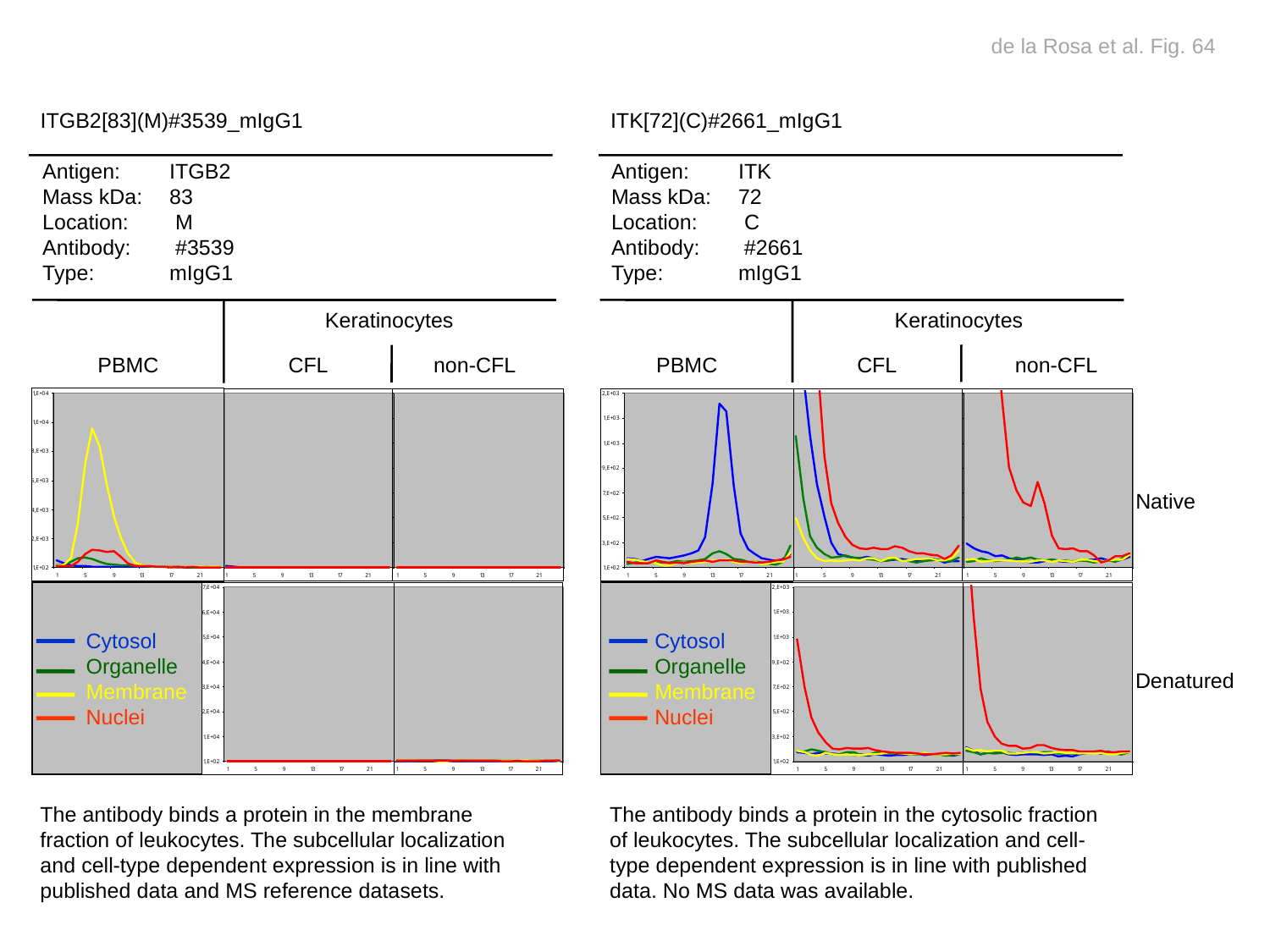

de la Rosa et al. Fig. <number>
ITGB2[83](M)#3539_mIgG1
ITK[72](C)#2661_mIgG1
Antigen: 	ITGB2
Mass kDa:	83
Location: 	 M
Antibody: 	 #3539
Type:	mIgG1
Antigen: 	ITK
Mass kDa:	72
Location: 	 C
Antibody: 	 #2661
Type:	mIgG1
The antibody binds a protein in the membrane fraction of leukocytes. The subcellular localization and cell-type dependent expression is in line with published data and MS reference datasets.
The antibody binds a protein in the cytosolic fraction of leukocytes. The subcellular localization and cell-type dependent expression is in line with published data. No MS data was available.

## Slide 65
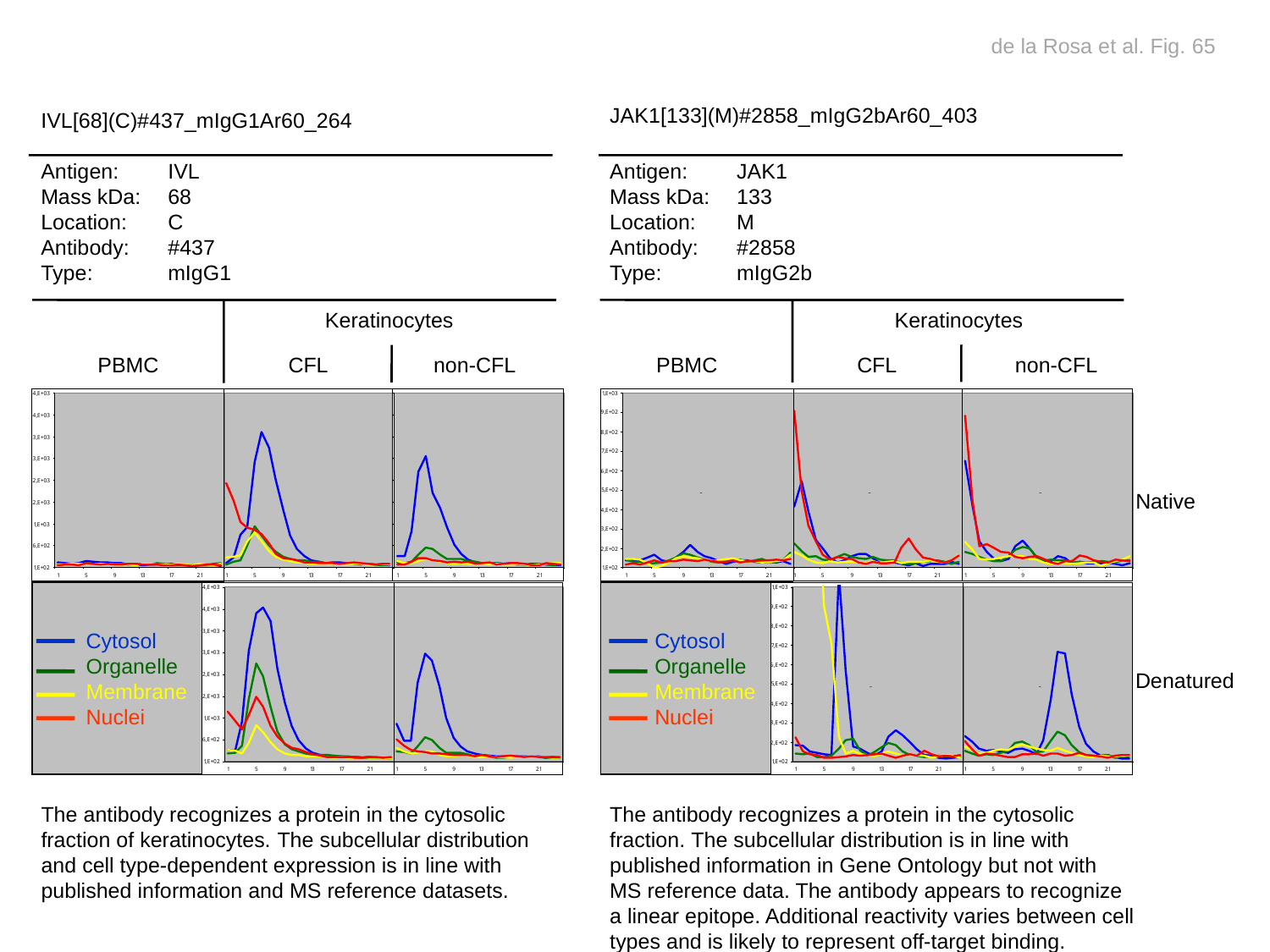

de la Rosa et al. Fig. <number>
# IVL[68](C)#437_mIgG1Ar60_264
JAK1[133](M)#2858_mIgG2bAr60_403
Antigen: 	IVL
Mass kDa:	68
Location: 	C
Antibody: 	#437
Type:	mIgG1
Antigen: 	JAK1
Mass kDa:	133
Location: 	M
Antibody: 	#2858
Type:	mIgG2b
The antibody recognizes a protein in the cytosolic fraction of keratinocytes. The subcellular distribution and cell type-dependent expression is in line with published information and MS reference datasets.
The antibody recognizes a protein in the cytosolic fraction. The subcellular distribution is in line with published information in Gene Ontology but not with MS reference data. The antibody appears to recognize a linear epitope. Additional reactivity varies between cell types and is likely to represent off-target binding.

## Slide 66
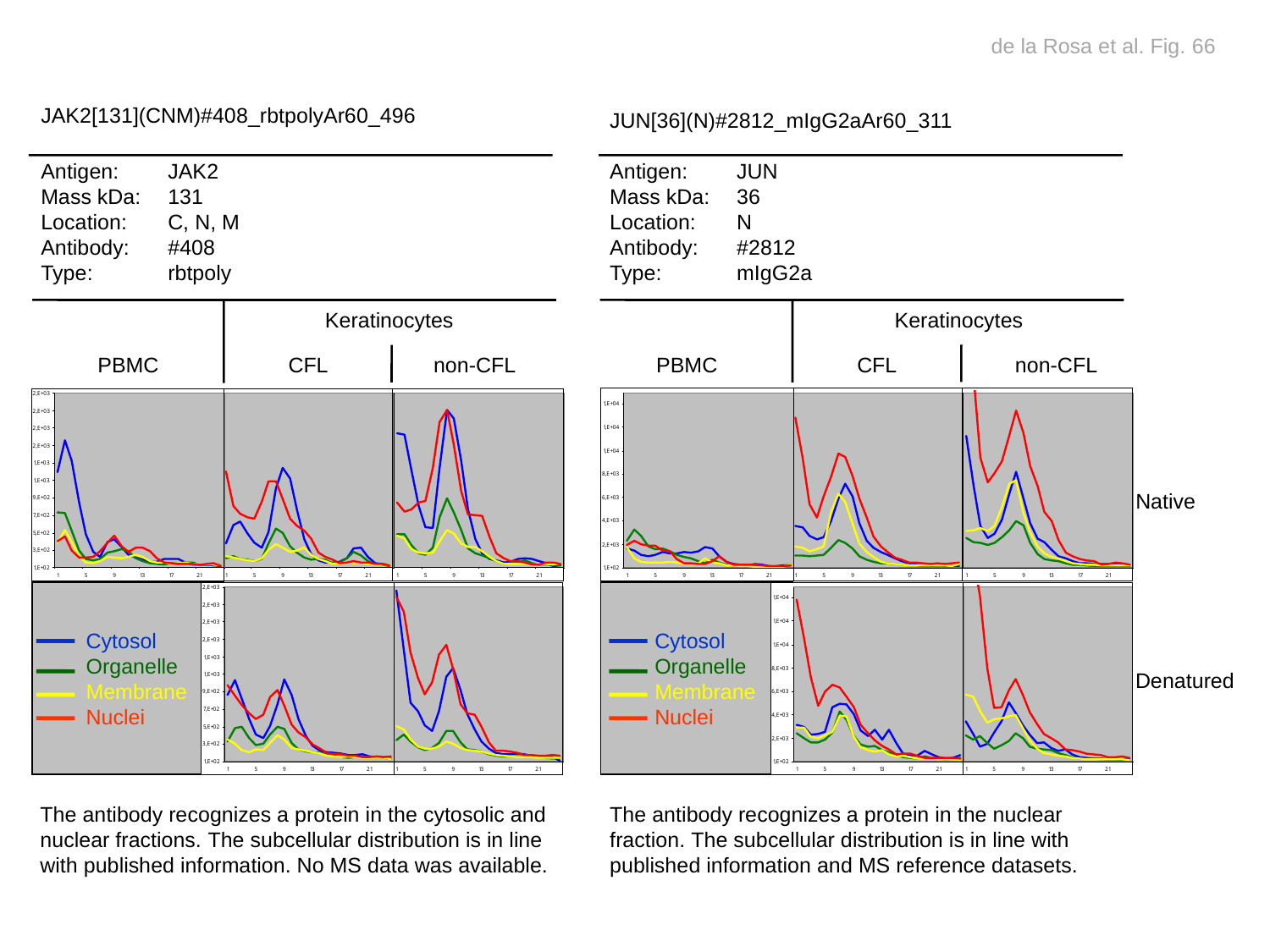

de la Rosa et al. Fig. <number>
# JAK2[131](CNM)#408_rbtpolyAr60_496
JUN[36](N)#2812_mIgG2aAr60_311
Antigen: 	JAK2
Mass kDa:	131
Location: 	C, N, M
Antibody: 	#408
Type:	rbtpoly
Antigen: 	JUN
Mass kDa:	36
Location: 	N
Antibody: 	#2812
Type:	mIgG2a
The antibody recognizes a protein in the cytosolic and nuclear fractions. The subcellular distribution is in line with published information. No MS data was available.
The antibody recognizes a protein in the nuclear fraction. The subcellular distribution is in line with published information and MS reference datasets.

## Slide 67
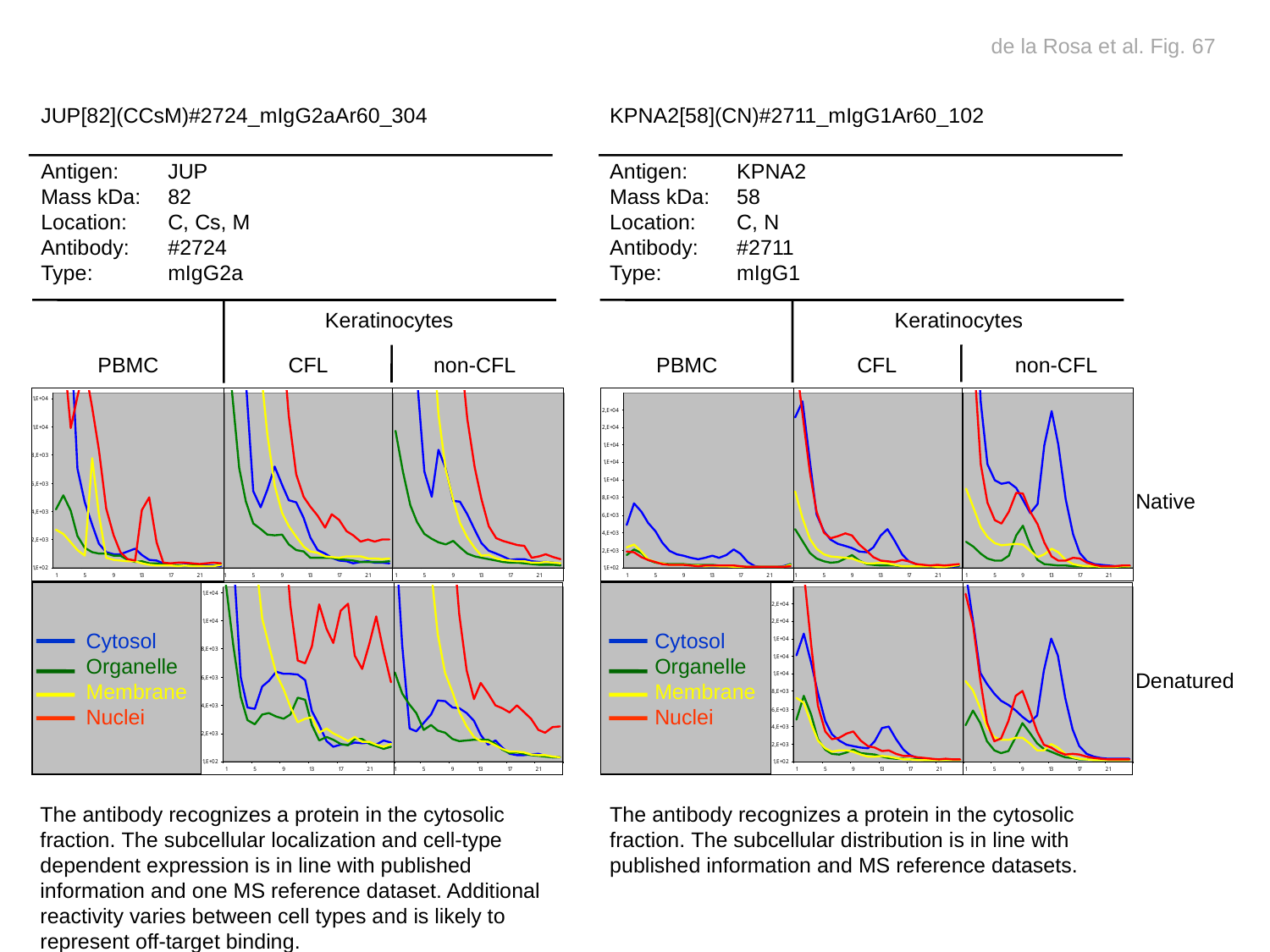

de la Rosa et al. Fig. <number>
# JUP[82](CCsM)#2724_mIgG2aAr60_304
KPNA2[58](CN)#2711_mIgG1Ar60_102
Antigen: 	JUP
Mass kDa:	82
Location: 	C, Cs, M
Antibody: 	#2724
Type:	mIgG2a
Antigen: 	KPNA2
Mass kDa:	58
Location: 	C, N
Antibody: 	#2711
Type:	mIgG1
The antibody recognizes a protein in the cytosolic fraction. The subcellular localization and cell-type dependent expression is in line with published information and one MS reference dataset. Additional reactivity varies between cell types and is likely to represent off-target binding.
The antibody recognizes a protein in the cytosolic fraction. The subcellular distribution is in line with published information and MS reference datasets.

## Slide 68
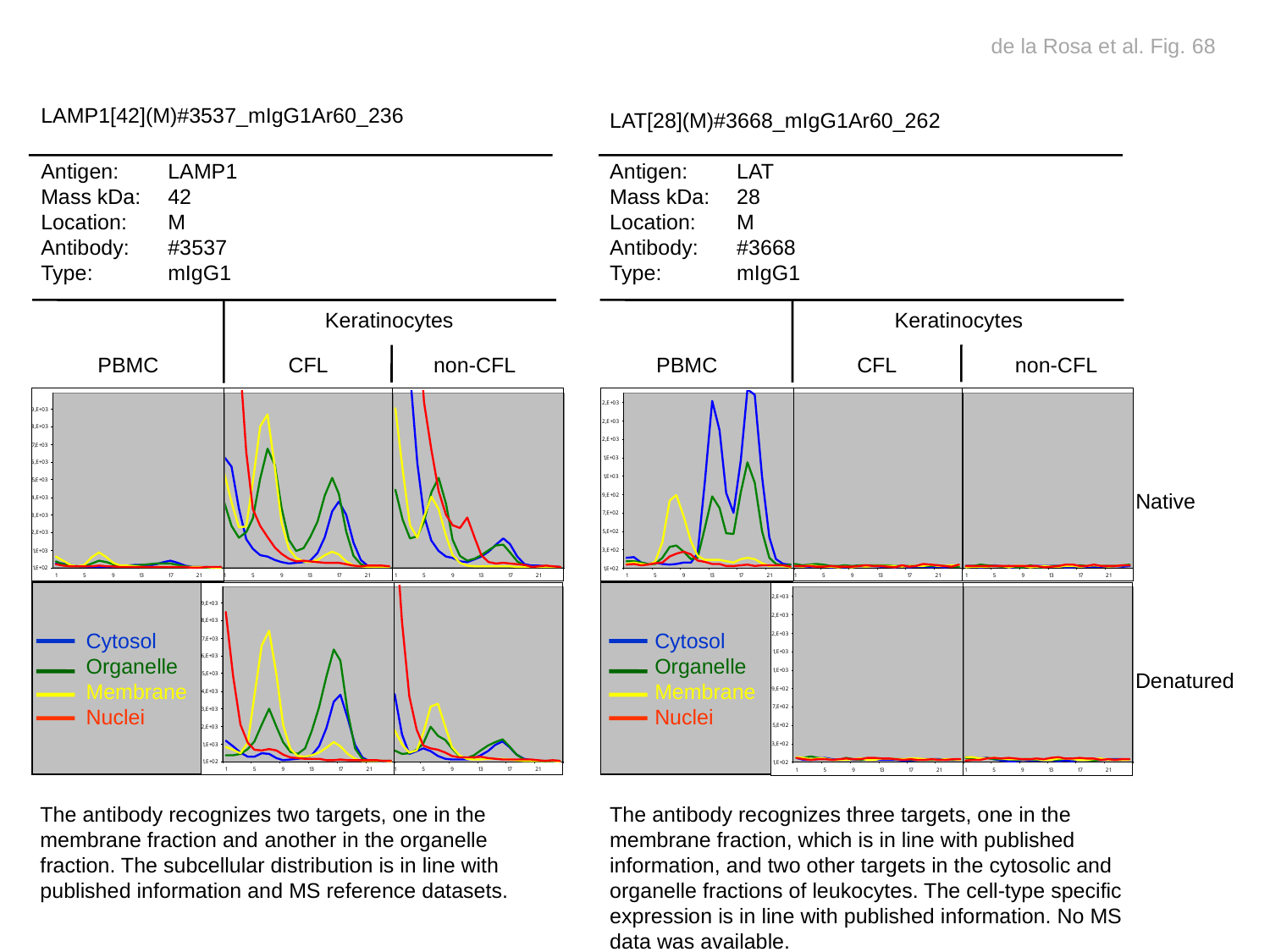

de la Rosa et al. Fig. <number>
# LAMP1[42](M)#3537_mIgG1Ar60_236
LAT[28](M)#3668_mIgG1Ar60_262
Antigen: 	LAMP1
Mass kDa:	42
Location: 	M
Antibody: 	#3537
Type:	mIgG1
Antigen: 	LAT
Mass kDa:	28
Location: 	M
Antibody: 	#3668
Type:	mIgG1
The antibody recognizes two targets, one in the membrane fraction and another in the organelle fraction. The subcellular distribution is in line with published information and MS reference datasets.
The antibody recognizes three targets, one in the membrane fraction, which is in line with published information, and two other targets in the cytosolic and organelle fractions of leukocytes. The cell-type specific expression is in line with published information. No MS data was available.

## Slide 69
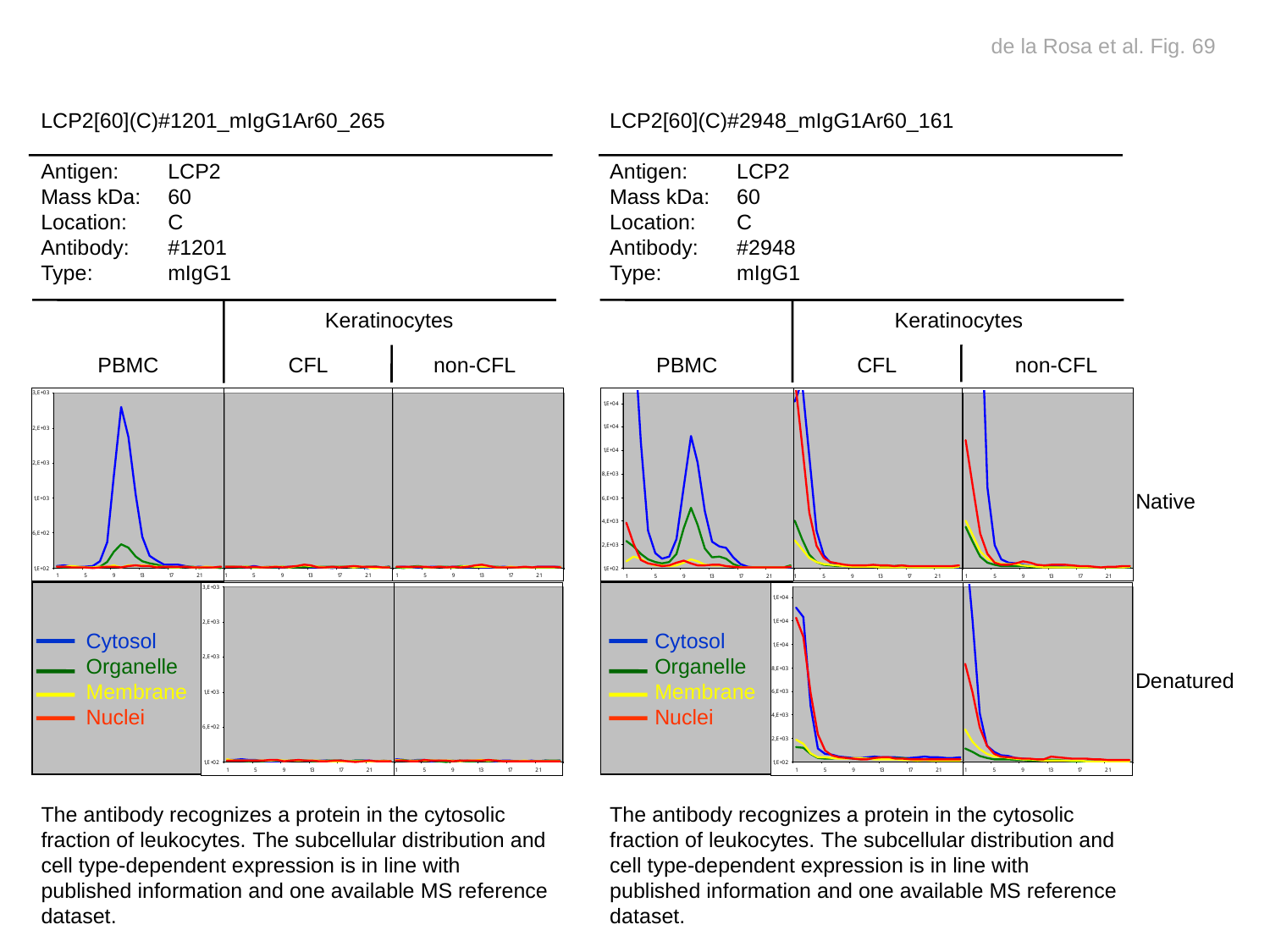

de la Rosa et al. Fig. <number>
# LCP2[60](C)#1201_mIgG1Ar60_265
LCP2[60](C)#2948_mIgG1Ar60_161
Antigen: 	LCP2
Mass kDa:	60
Location: 	C
Antibody: 	#1201
Type:	mIgG1
Antigen: 	LCP2
Mass kDa:	60
Location: 	C
Antibody: 	#2948
Type:	mIgG1
The antibody recognizes a protein in the cytosolic fraction of leukocytes. The subcellular distribution and cell type-dependent expression is in line with published information and one available MS reference dataset.
The antibody recognizes a protein in the cytosolic fraction of leukocytes. The subcellular distribution and cell type-dependent expression is in line with published information and one available MS reference dataset.

## Slide 70
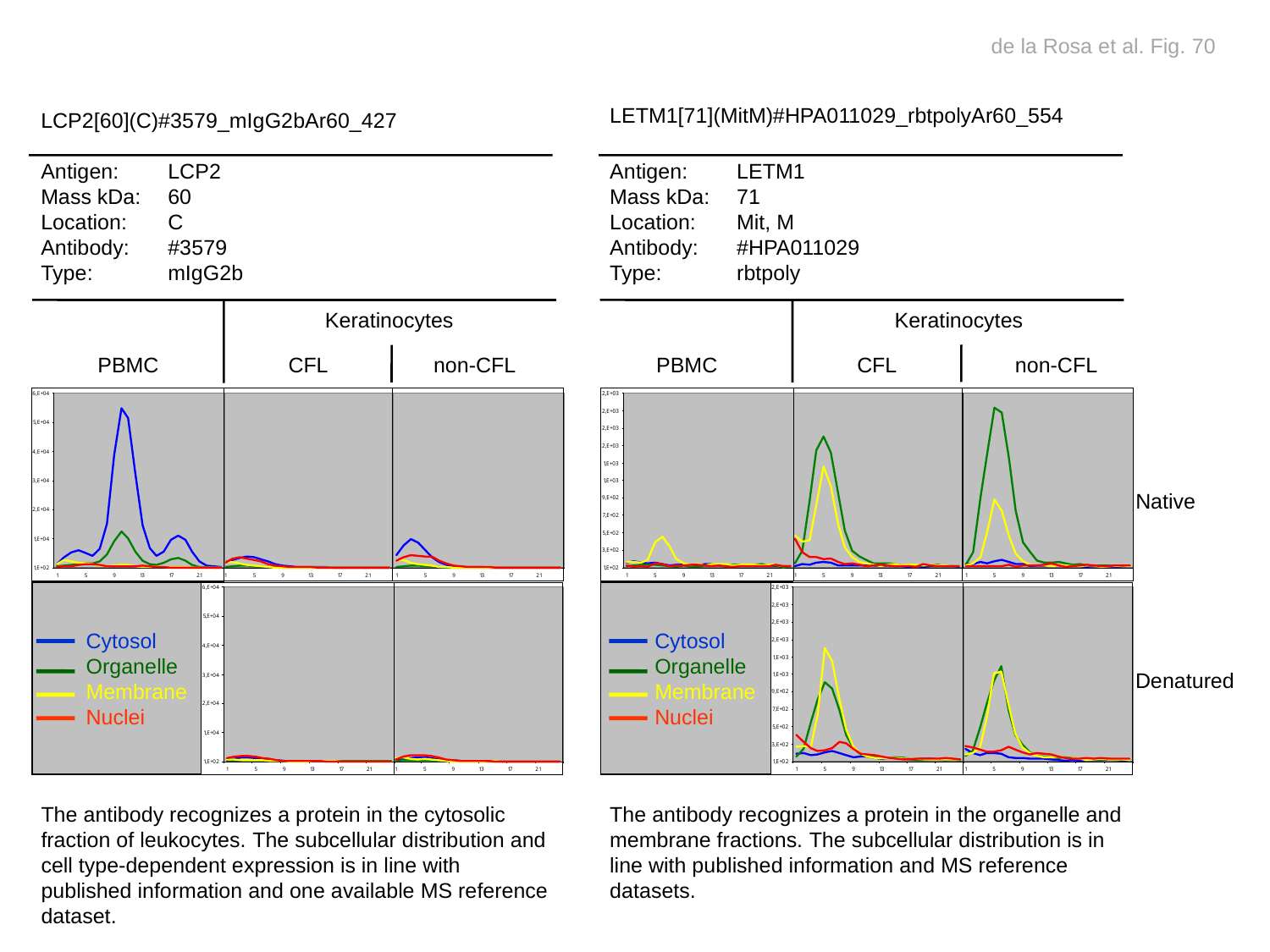

de la Rosa et al. Fig. <number>
# LCP2[60](C)#3579_mIgG2bAr60_427
LETM1[71](MitM)#HPA011029_rbtpolyAr60_554
Antigen: 	LCP2
Mass kDa:	60
Location: 	C
Antibody: 	#3579
Type:	mIgG2b
Antigen: 	LETM1
Mass kDa:	71
Location: 	Mit, M
Antibody: 	#HPA011029
Type:	rbtpoly
The antibody recognizes a protein in the cytosolic fraction of leukocytes. The subcellular distribution and cell type-dependent expression is in line with published information and one available MS reference dataset.
The antibody recognizes a protein in the organelle and membrane fractions. The subcellular distribution is in line with published information and MS reference datasets.

## Slide 71
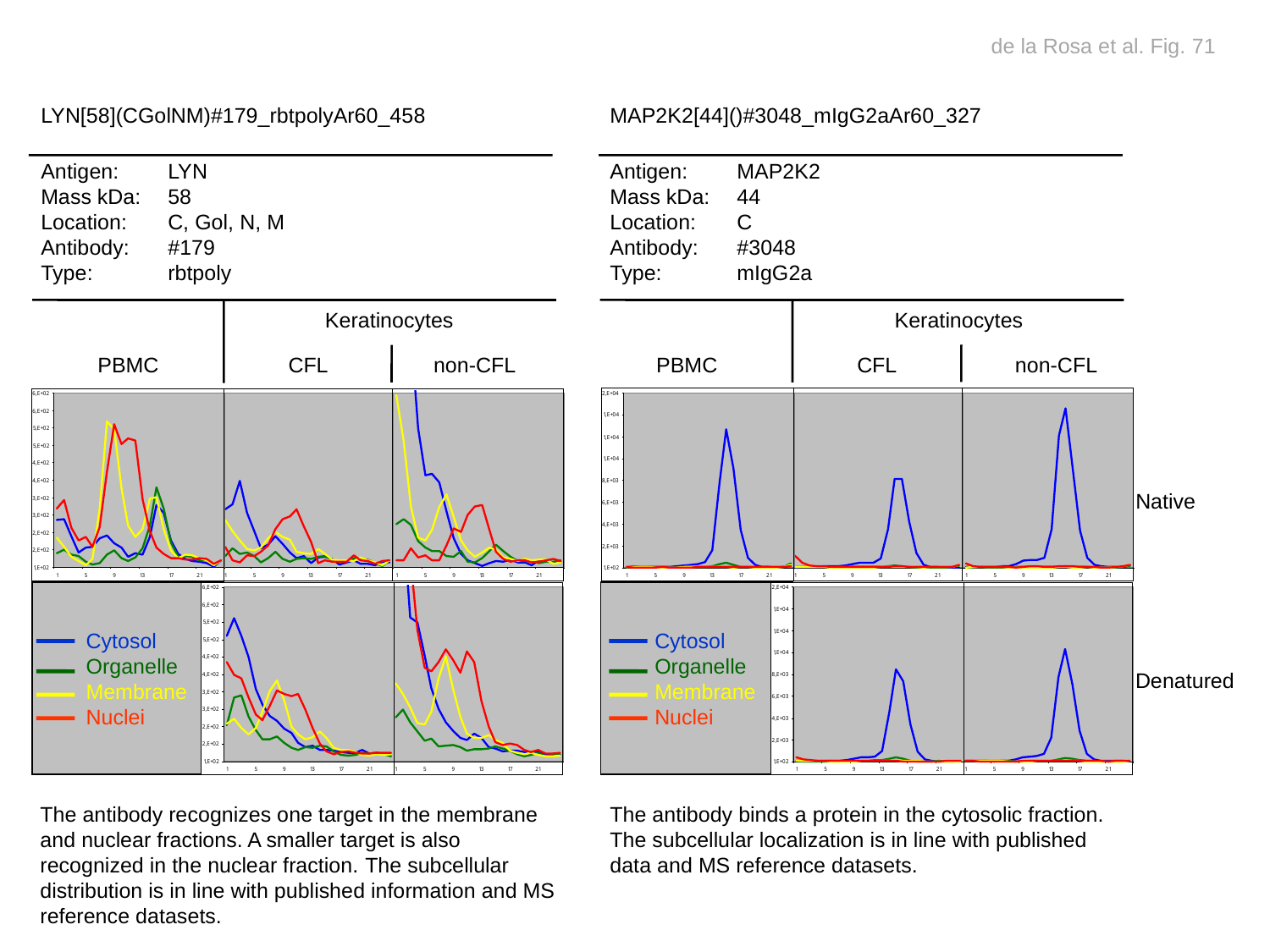

de la Rosa et al. Fig. <number>
# LYN[58](CGolNM)#179_rbtpolyAr60_458
MAP2K2[44]()#3048_mIgG2aAr60_327
Antigen: 	LYN
Mass kDa:	58
Location: 	C, Gol, N, M
Antibody: 	#179
Type:	rbtpoly
Antigen: 	MAP2K2
Mass kDa:	44
Location: 	C
Antibody: 	#3048
Type:	mIgG2a
The antibody recognizes one target in the membrane and nuclear fractions. A smaller target is also recognized in the nuclear fraction. The subcellular distribution is in line with published information and MS reference datasets.
The antibody binds a protein in the cytosolic fraction. The subcellular localization is in line with published data and MS reference datasets.

## Slide 72
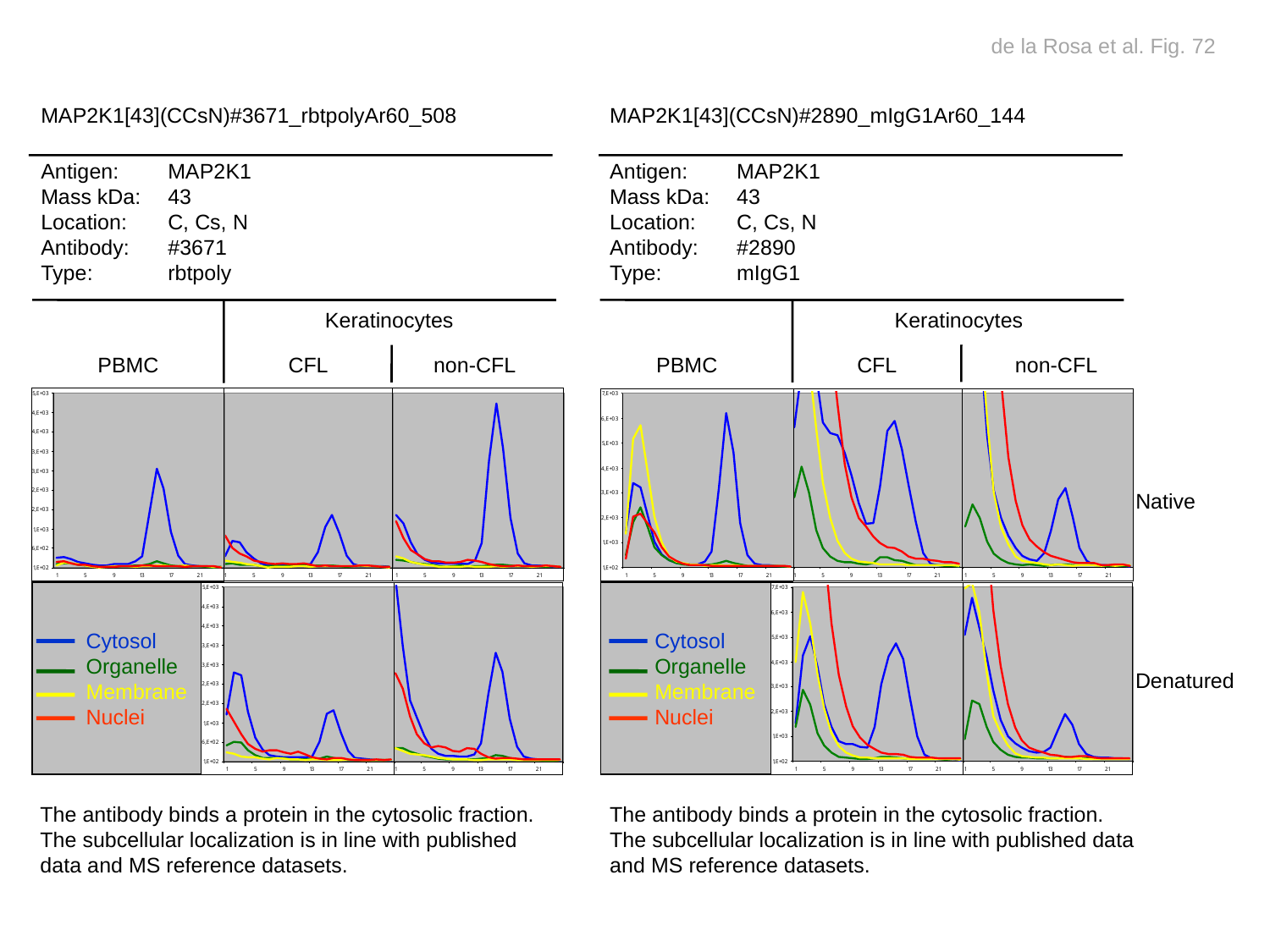

de la Rosa et al. Fig. <number>
# MAP2K1[43](CCsN)#3671_rbtpolyAr60_508
MAP2K1[43](CCsN)#2890_mIgG1Ar60_144
Antigen: 	MAP2K1
Mass kDa:	43
Location: 	C, Cs, N
Antibody: 	#3671
Type:	rbtpoly
Antigen: 	MAP2K1
Mass kDa:	43
Location: 	C, Cs, N
Antibody: 	#2890
Type:	mIgG1
The antibody binds a protein in the cytosolic fraction. The subcellular localization is in line with published data and MS reference datasets.
The antibody binds a protein in the cytosolic fraction. The subcellular localization is in line with published data and MS reference datasets.

## Slide 73
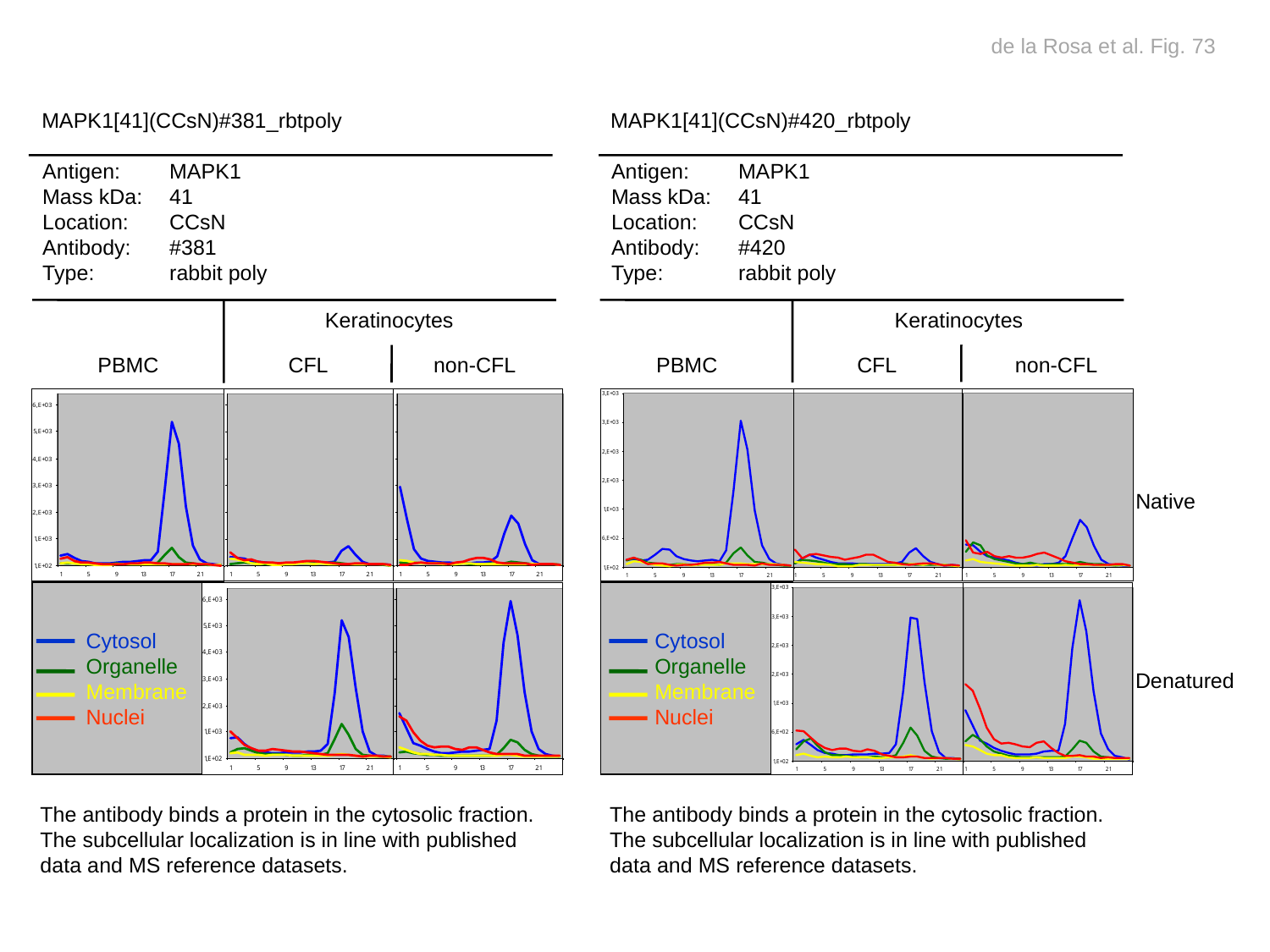

de la Rosa et al. Fig. <number>
MAPK1[41](CCsN)#381_rbtpoly
MAPK1[41](CCsN)#420_rbtpoly
Antigen: 	MAPK1
Mass kDa:	41
Location: 	CCsN
Antibody: 	#381
Type:	rabbit poly
Antigen: 	MAPK1
Mass kDa:	41
Location: 	CCsN
Antibody: 	#420
Type:	rabbit poly
The antibody binds a protein in the cytosolic fraction. The subcellular localization is in line with published data and MS reference datasets.
The antibody binds a protein in the cytosolic fraction. The subcellular localization is in line with published data and MS reference datasets.

## Slide 74
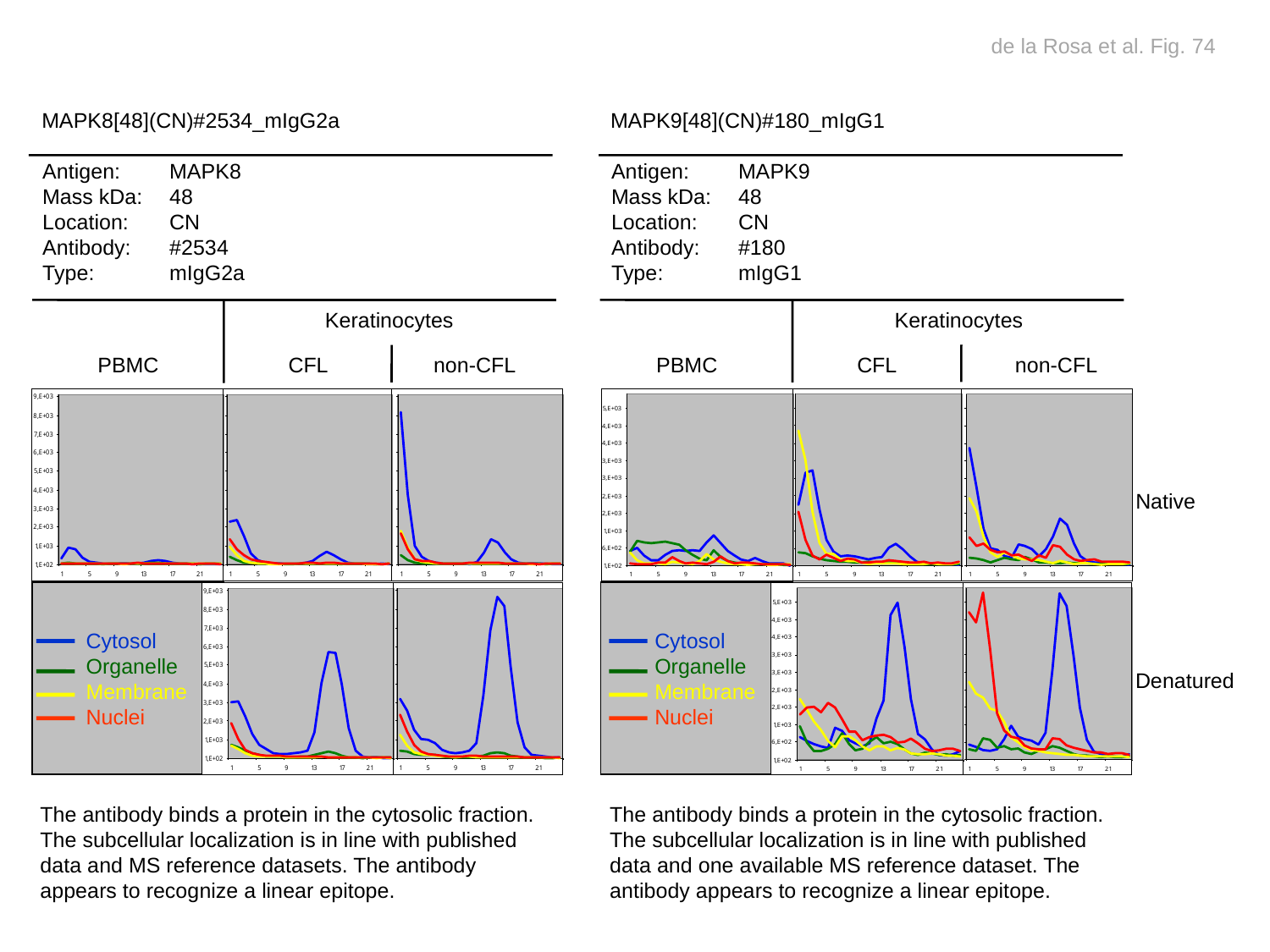

de la Rosa et al. Fig. <number>
MAPK8[48](CN)#2534_mIgG2a
MAPK9[48](CN)#180_mIgG1
Antigen: 	MAPK8
Mass kDa:	48
Location: 	CN
Antibody: 	#2534
Type:	mIgG2a
Antigen: 	MAPK9
Mass kDa:	48
Location: 	CN
Antibody: 	#180
Type:	mIgG1
The antibody binds a protein in the cytosolic fraction. The subcellular localization is in line with published data and MS reference datasets. The antibody appears to recognize a linear epitope.
The antibody binds a protein in the cytosolic fraction. The subcellular localization is in line with published data and one available MS reference dataset. The antibody appears to recognize a linear epitope.

## Slide 75
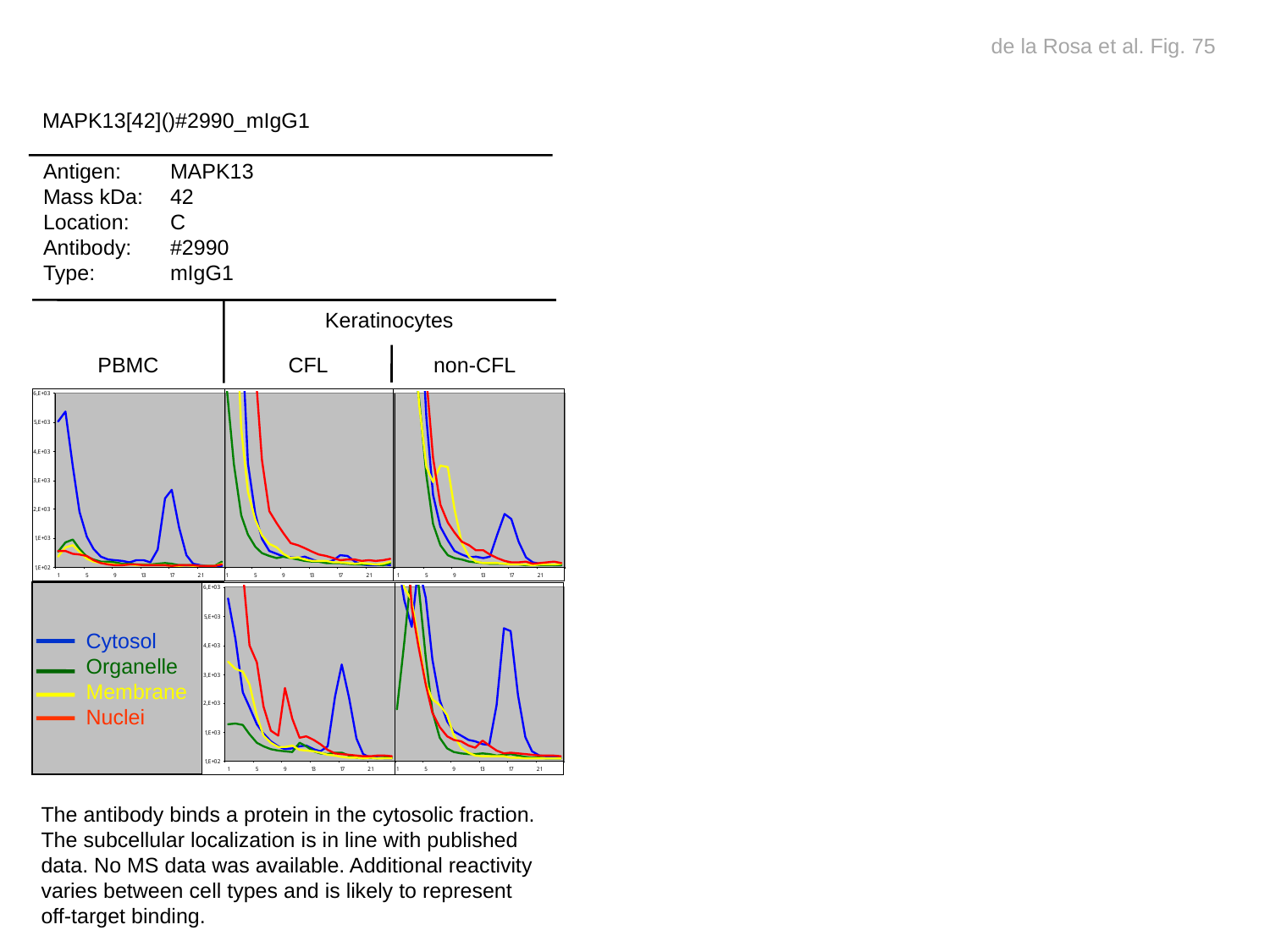

de la Rosa et al. Fig. <number>
MAPK13[42]()#2990_mIgG1
Antigen: 	MAPK13
Mass kDa:	42
Location: 	C
Antibody: 	#2990
Type:	mIgG1
The antibody binds a protein in the cytosolic fraction. The subcellular localization is in line with published data. No MS data was available. Additional reactivity varies between cell types and is likely to represent off-target binding.

## Slide 76
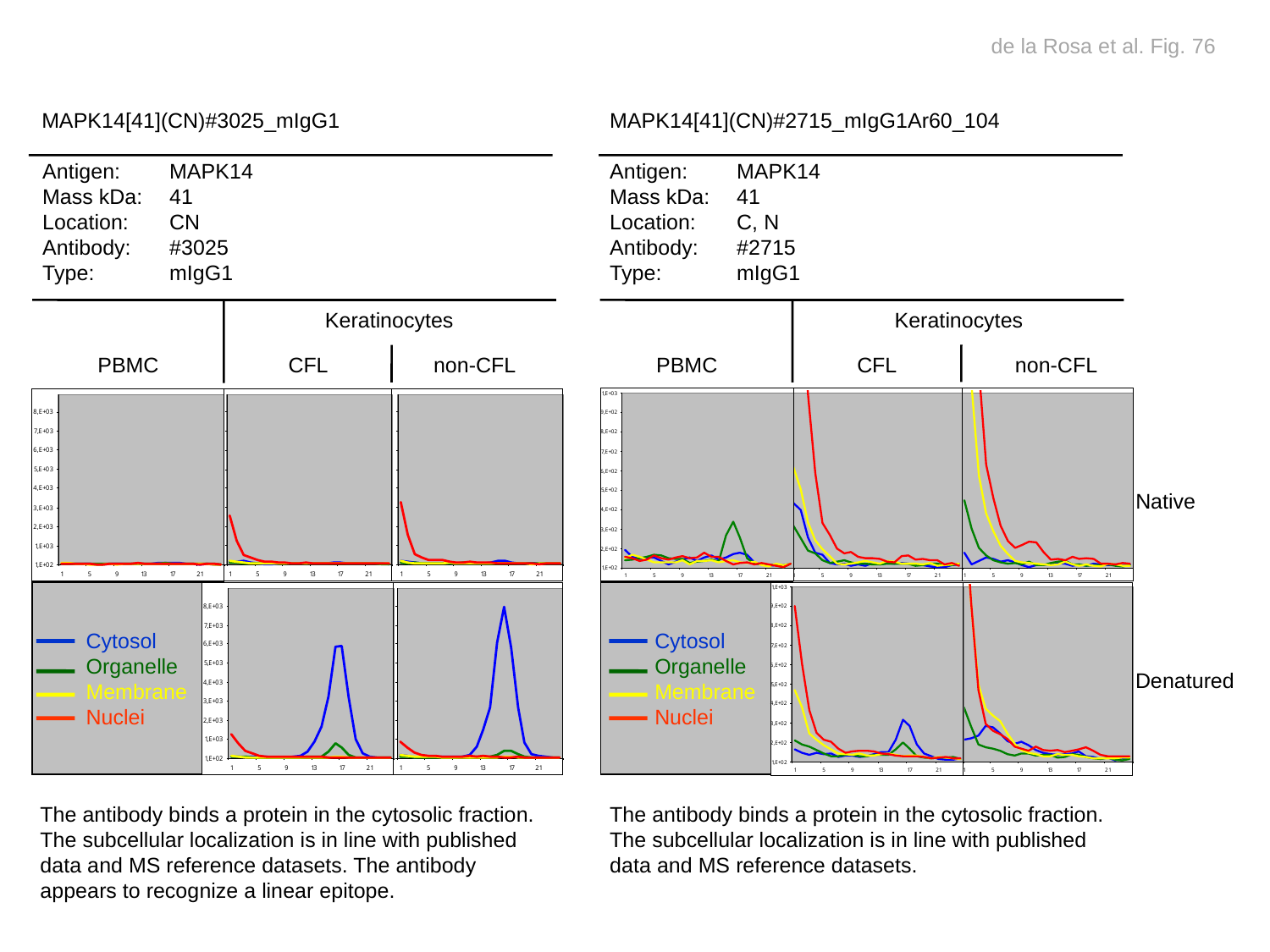

de la Rosa et al. Fig. <number>
# MAPK14[41](CN)#2715_mIgG1Ar60_104
MAPK14[41](CN)#3025_mIgG1
Antigen: 	MAPK14
Mass kDa:	41
Location: 	CN
Antibody: 	#3025
Type:	mIgG1
Antigen: 	MAPK14
Mass kDa:	41
Location: 	C, N
Antibody: 	#2715
Type:	mIgG1
The antibody binds a protein in the cytosolic fraction. The subcellular localization is in line with published data and MS reference datasets. The antibody appears to recognize a linear epitope.
The antibody binds a protein in the cytosolic fraction. The subcellular localization is in line with published data and MS reference datasets.

## Slide 77
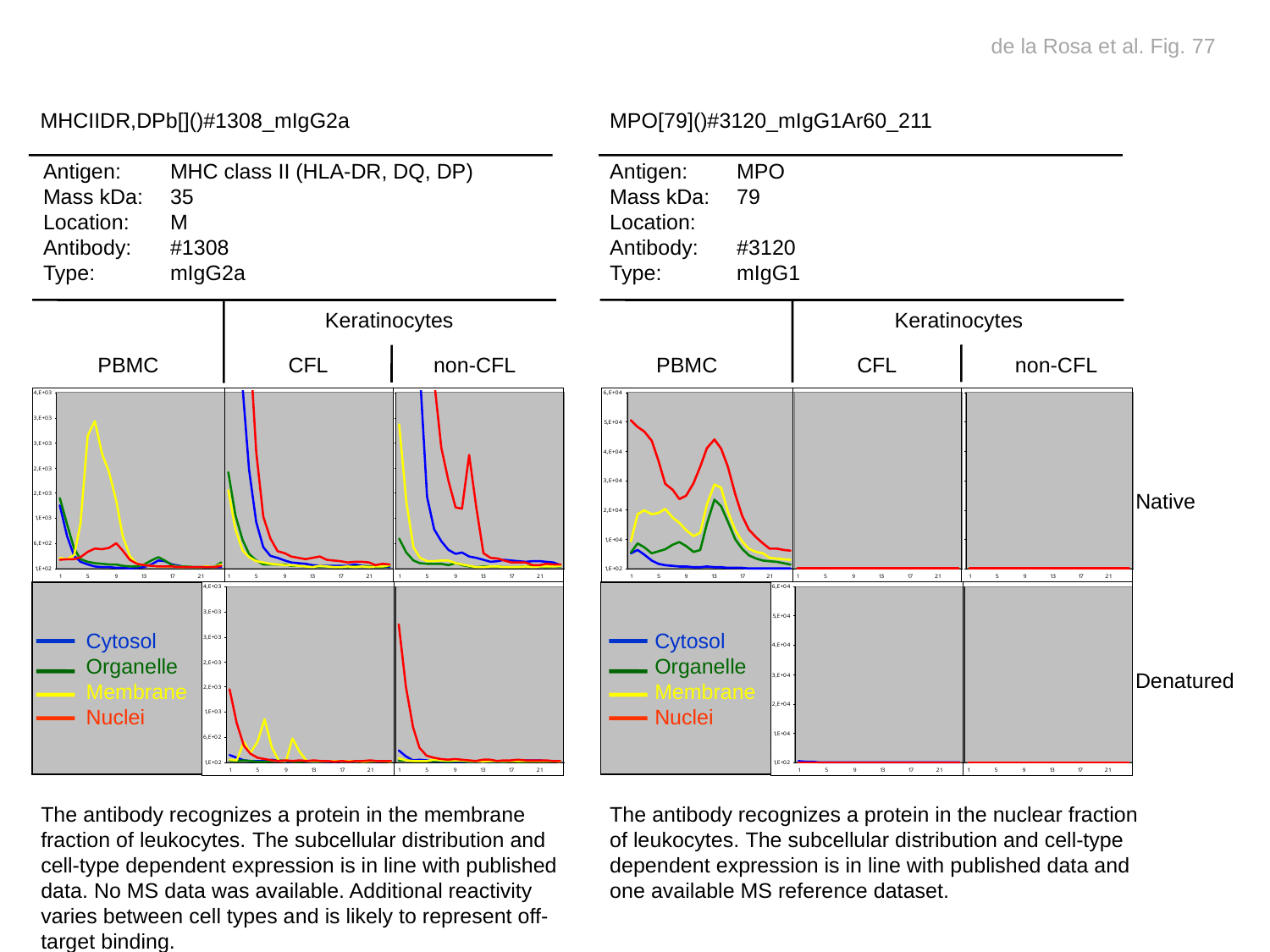

de la Rosa et al. Fig. <number>
# MPO[79]()#3120_mIgG1Ar60_211
MHCIIDR,DPb[]()#1308_mIgG2a
Antigen:	MHC class II (HLA-DR, DQ, DP)
Mass kDa:	35
Location: 	M
Antibody: 	#1308
Type:	mIgG2a
Antigen: 	MPO
Mass kDa:	79
Location:
Antibody: 	#3120
Type:	mIgG1
The antibody recognizes a protein in the membrane fraction of leukocytes. The subcellular distribution and cell-type dependent expression is in line with published data. No MS data was available. Additional reactivity varies between cell types and is likely to represent off-target binding.
The antibody recognizes a protein in the nuclear fraction of leukocytes. The subcellular distribution and cell-type dependent expression is in line with published data and one available MS reference dataset.

## Slide 78
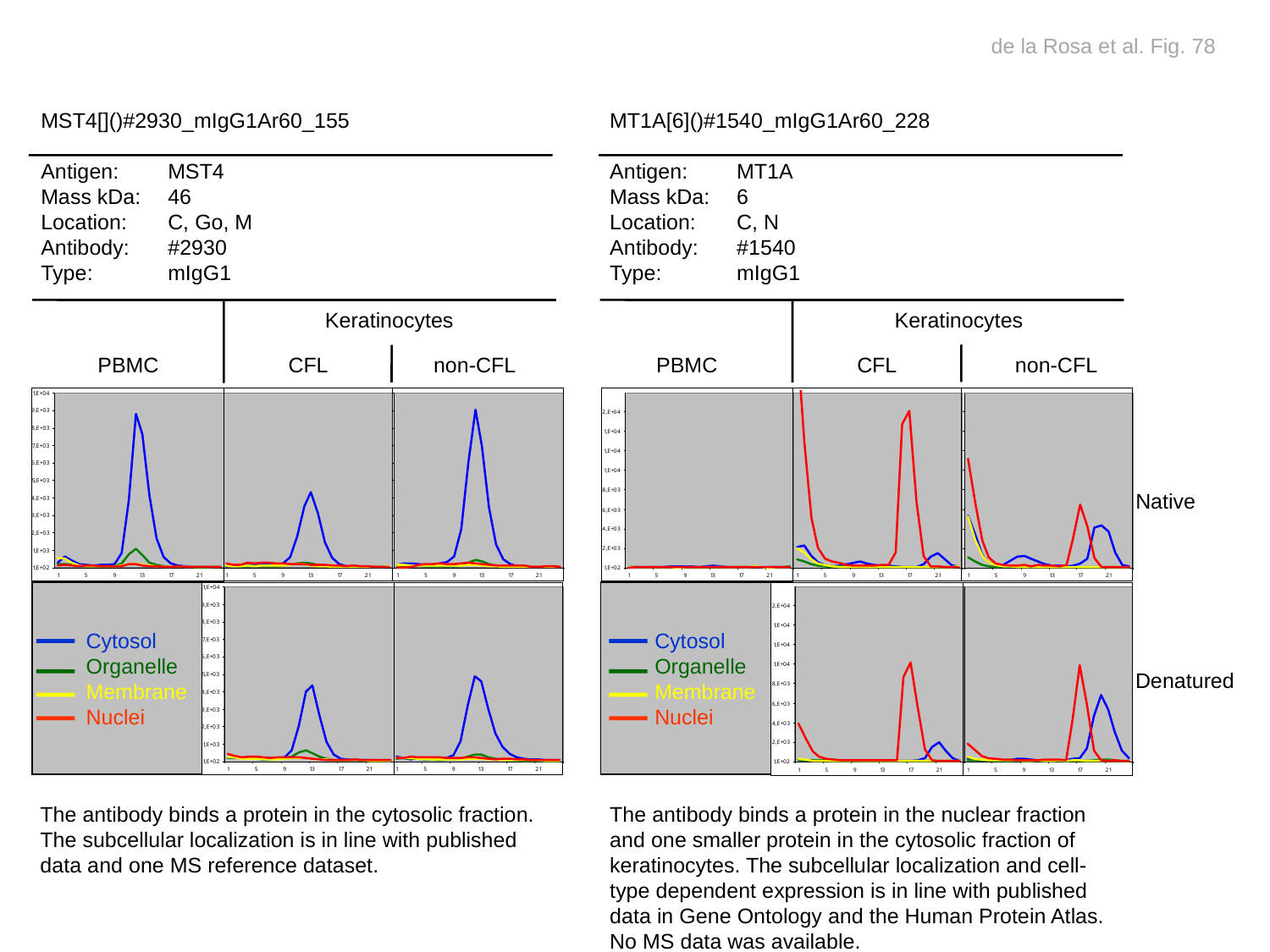

de la Rosa et al. Fig. <number>
# MST4[]()#2930_mIgG1Ar60_155
MT1A[6]()#1540_mIgG1Ar60_228
Antigen: 	MST4
Mass kDa:	46
Location: 	C, Go, M
Antibody: 	#2930
Type:	mIgG1
Antigen: 	MT1A
Mass kDa:	6
Location: 	C, N
Antibody: 	#1540
Type:	mIgG1
The antibody binds a protein in the cytosolic fraction. The subcellular localization is in line with published data and one MS reference dataset.
The antibody binds a protein in the nuclear fraction and one smaller protein in the cytosolic fraction of keratinocytes. The subcellular localization and cell-type dependent expression is in line with published data in Gene Ontology and the Human Protein Atlas. No MS data was available.

## Slide 79
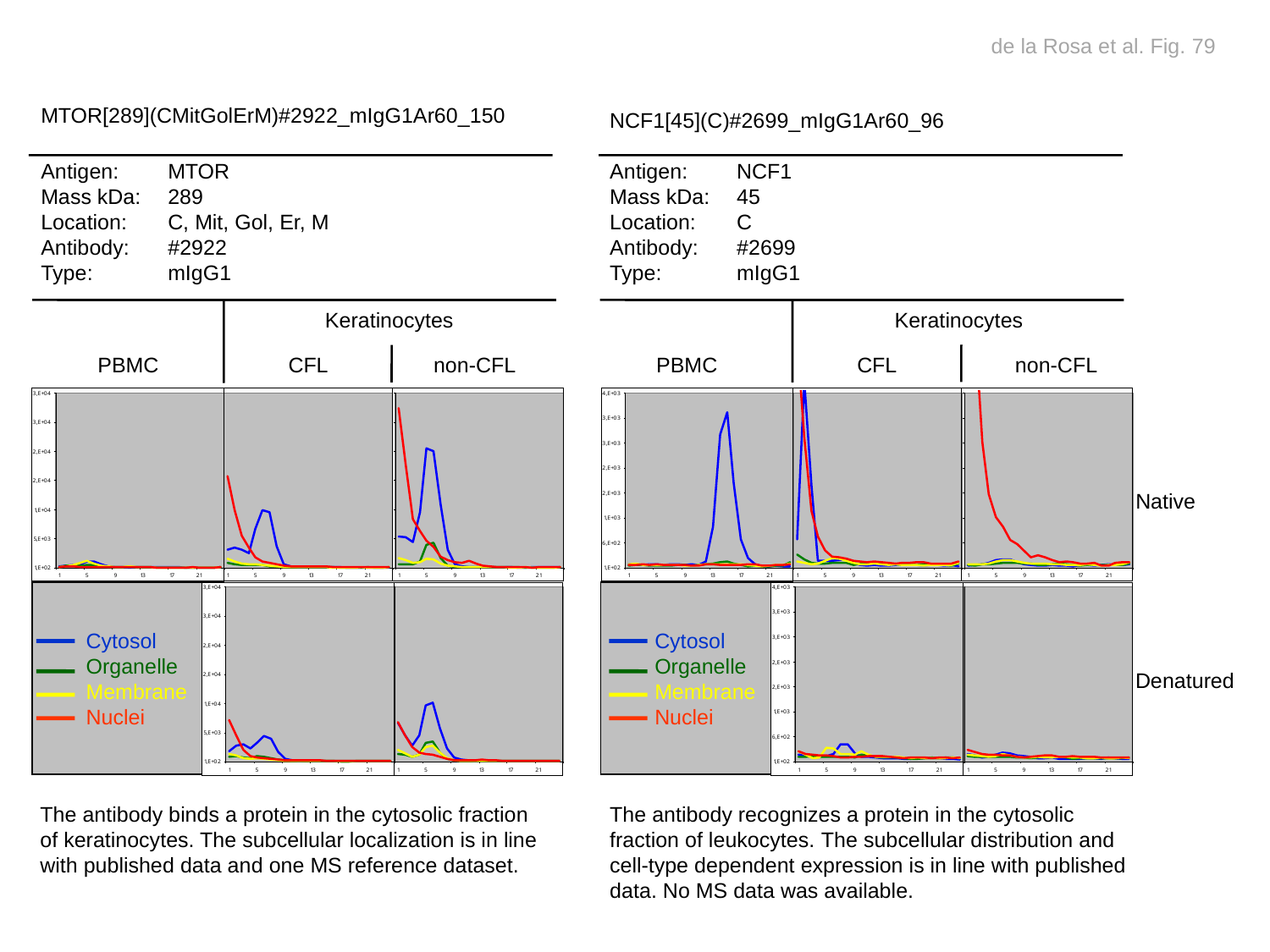

de la Rosa et al. Fig. <number>
# MTOR[289](CMitGolErM)#2922_mIgG1Ar60_150
NCF1[45](C)#2699_mIgG1Ar60_96
Antigen: 	MTOR
Mass kDa:	289
Location: 	C, Mit, Gol, Er, M
Antibody: 	#2922
Type:	mIgG1
Antigen: 	NCF1
Mass kDa:	45
Location: 	C
Antibody: 	#2699
Type:	mIgG1
The antibody binds a protein in the cytosolic fraction of keratinocytes. The subcellular localization is in line with published data and one MS reference dataset.
The antibody recognizes a protein in the cytosolic fraction of leukocytes. The subcellular distribution and cell-type dependent expression is in line with published data. No MS data was available.

## Slide 80
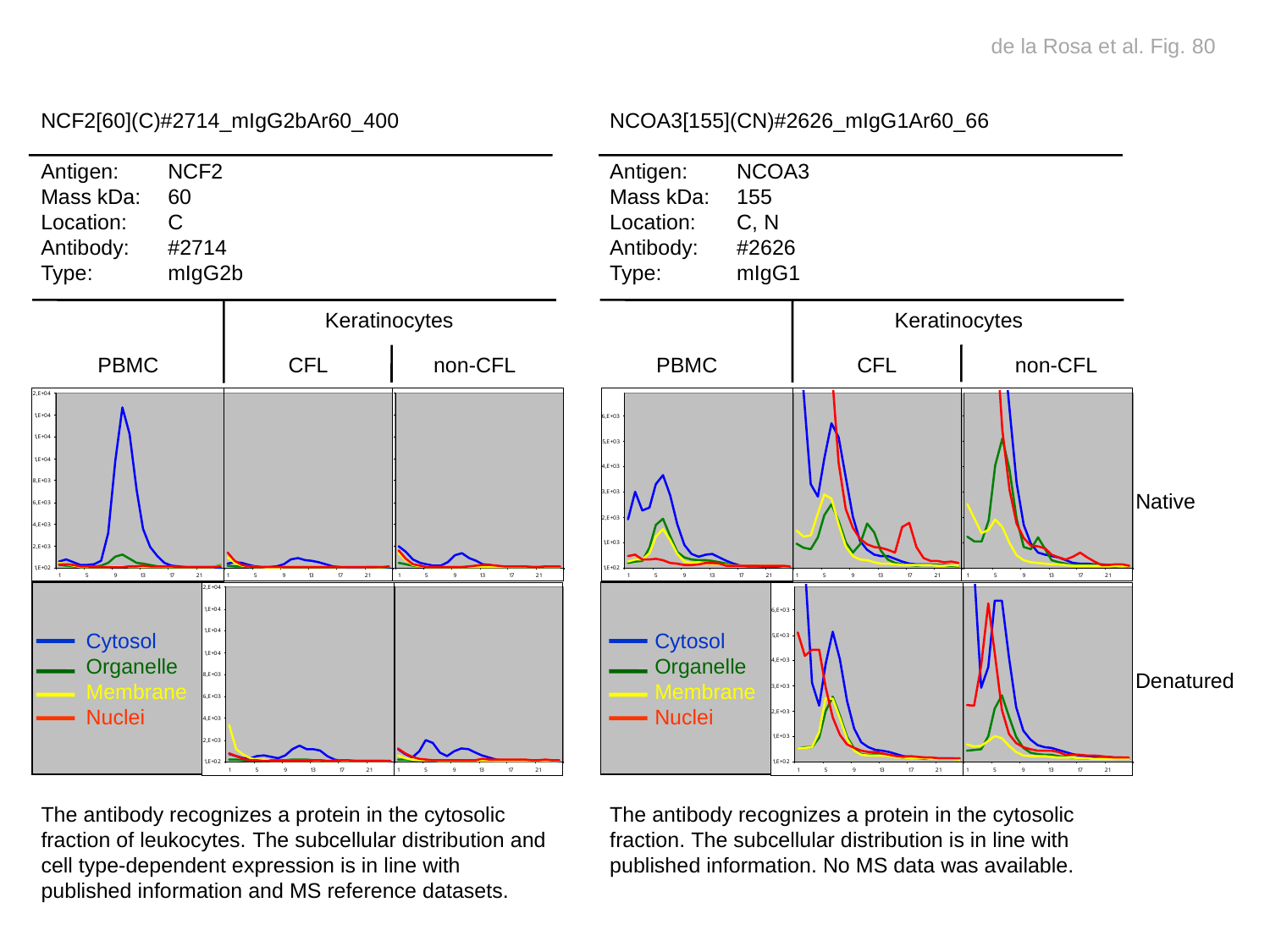

de la Rosa et al. Fig. <number>
# NCF2[60](C)#2714_mIgG2bAr60_400
NCOA3[155](CN)#2626_mIgG1Ar60_66
Antigen: 	NCF2
Mass kDa:	60
Location: 	C
Antibody: 	#2714
Type:	mIgG2b
Antigen: 	NCOA3
Mass kDa:	155
Location: 	C, N
Antibody: 	#2626
Type:	mIgG1
The antibody recognizes a protein in the cytosolic fraction of leukocytes. The subcellular distribution and cell type-dependent expression is in line with published information and MS reference datasets.
The antibody recognizes a protein in the cytosolic fraction. The subcellular distribution is in line with published information. No MS data was available.

## Slide 81
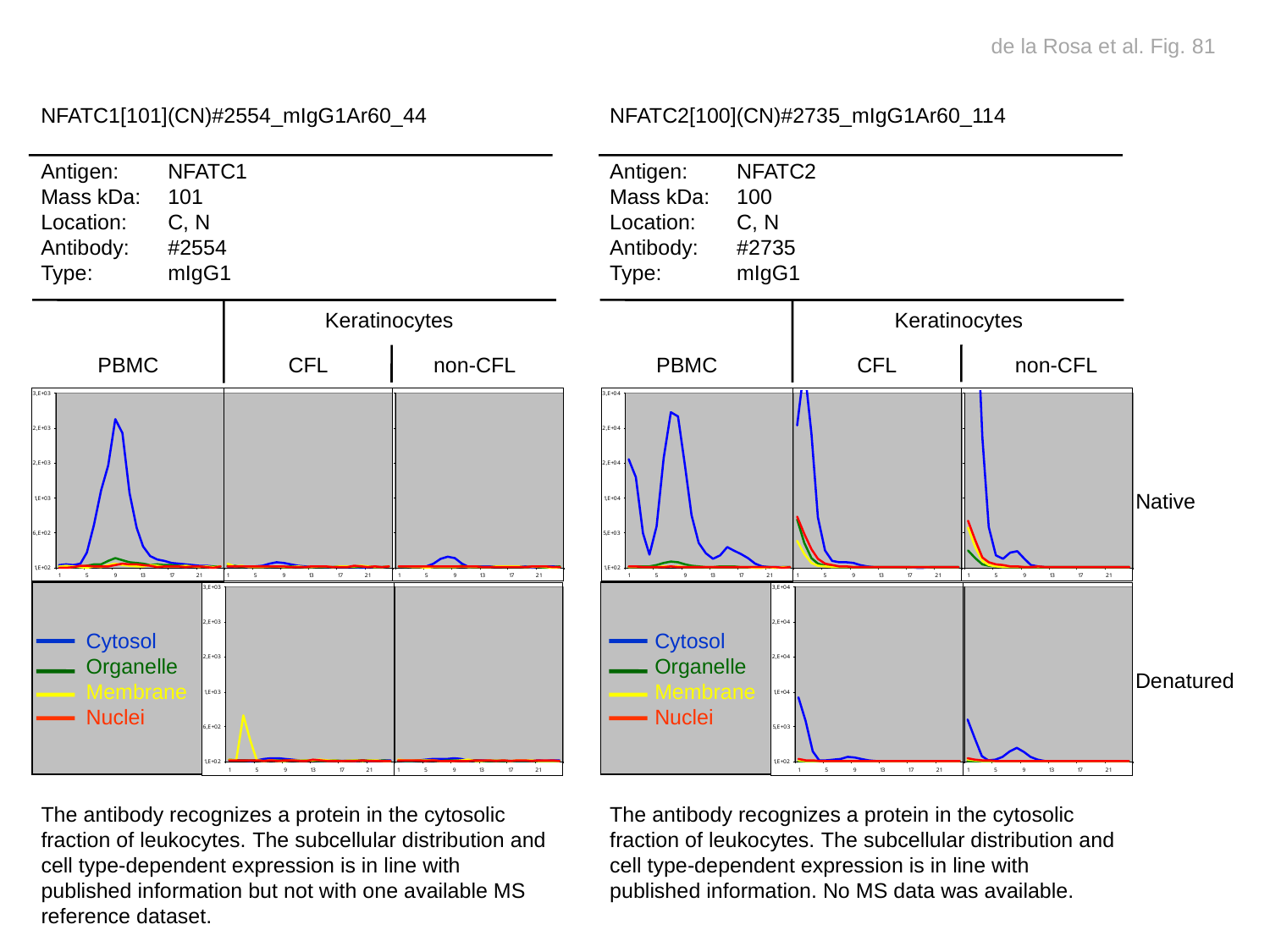

de la Rosa et al. Fig. <number>
# NFATC1[101](CN)#2554_mIgG1Ar60_44
NFATC2[100](CN)#2735_mIgG1Ar60_114
Antigen: 	NFATC1
Mass kDa:	101
Location: 	C, N
Antibody: 	#2554
Type:	mIgG1
Antigen: 	NFATC2
Mass kDa:	100
Location: 	C, N
Antibody: 	#2735
Type:	mIgG1
The antibody recognizes a protein in the cytosolic fraction of leukocytes. The subcellular distribution and cell type-dependent expression is in line with published information but not with one available MS reference dataset.
The antibody recognizes a protein in the cytosolic fraction of leukocytes. The subcellular distribution and cell type-dependent expression is in line with published information. No MS data was available.

## Slide 82
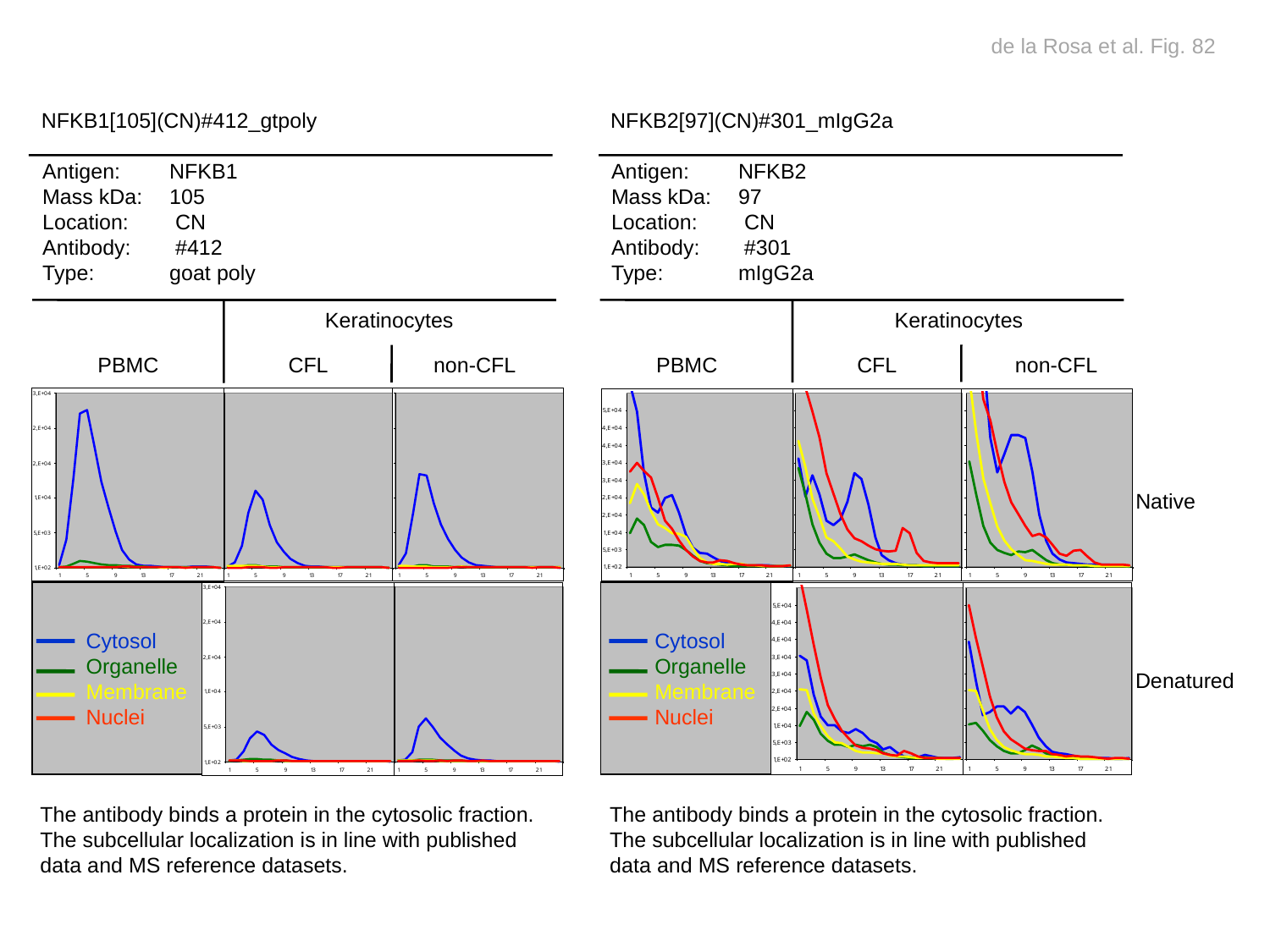

de la Rosa et al. Fig. <number>
NFKB1[105](CN)#412_gtpoly
NFKB2[97](CN)#301_mIgG2a
Antigen: 	NFKB1
Mass kDa:	105
Location: 	 CN
Antibody: 	 #412
Type:	goat poly
Antigen: 	NFKB2
Mass kDa:	97
Location: 	 CN
Antibody: 	 #301
Type:	mIgG2a
The antibody binds a protein in the cytosolic fraction. The subcellular localization is in line with published data and MS reference datasets.
The antibody binds a protein in the cytosolic fraction. The subcellular localization is in line with published data and MS reference datasets.

## Slide 83
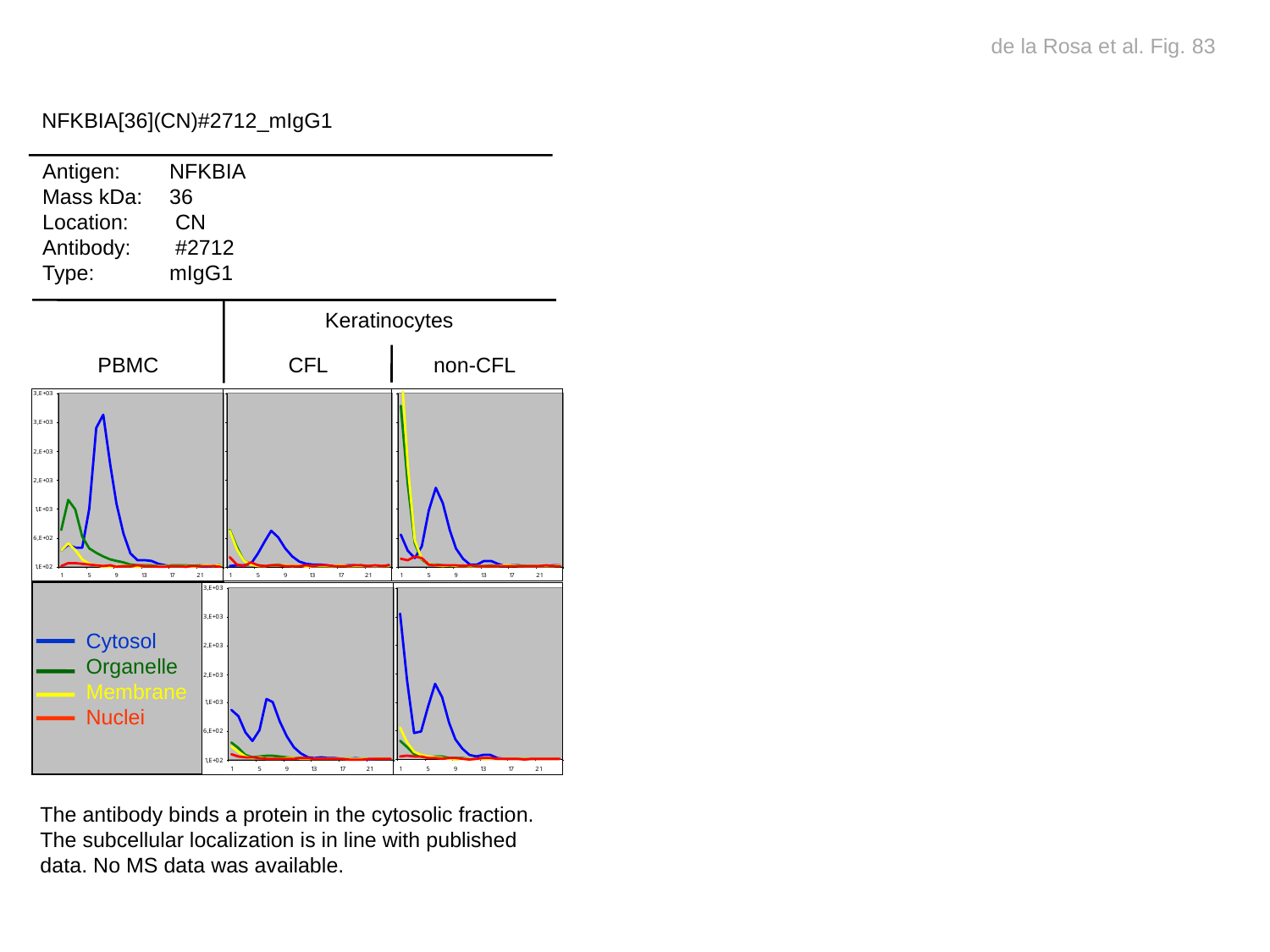

de la Rosa et al. Fig. <number>
NFKBIA[36](CN)#2712_mIgG1
Antigen: 	NFKBIA
Mass kDa:	36
Location: 	 CN
Antibody: 	 #2712
Type:	mIgG1
The antibody binds a protein in the cytosolic fraction. The subcellular localization is in line with published data. No MS data was available.

## Slide 84
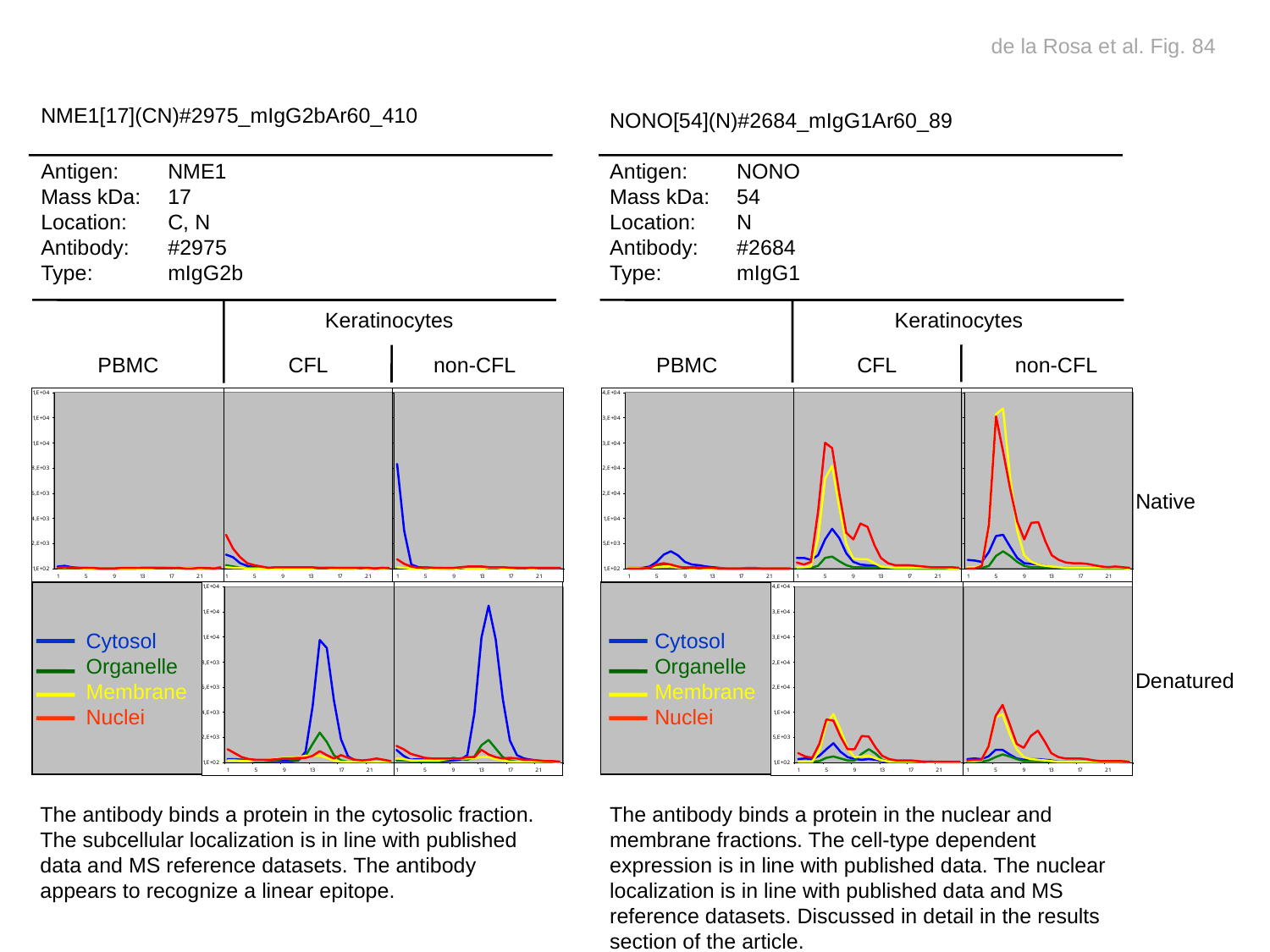

de la Rosa et al. Fig. <number>
# NME1[17](CN)#2975_mIgG2bAr60_410
NONO[54](N)#2684_mIgG1Ar60_89
Antigen: 	NME1
Mass kDa:	17
Location: 	C, N
Antibody: 	#2975
Type:	mIgG2b
Antigen: 	NONO
Mass kDa:	54
Location: 	N
Antibody: 	#2684
Type:	mIgG1
The antibody binds a protein in the cytosolic fraction. The subcellular localization is in line with published data and MS reference datasets. The antibody appears to recognize a linear epitope.
The antibody binds a protein in the nuclear and membrane fractions. The cell-type dependent expression is in line with published data. The nuclear localization is in line with published data and MS reference datasets. Discussed in detail in the results section of the article.

## Slide 85
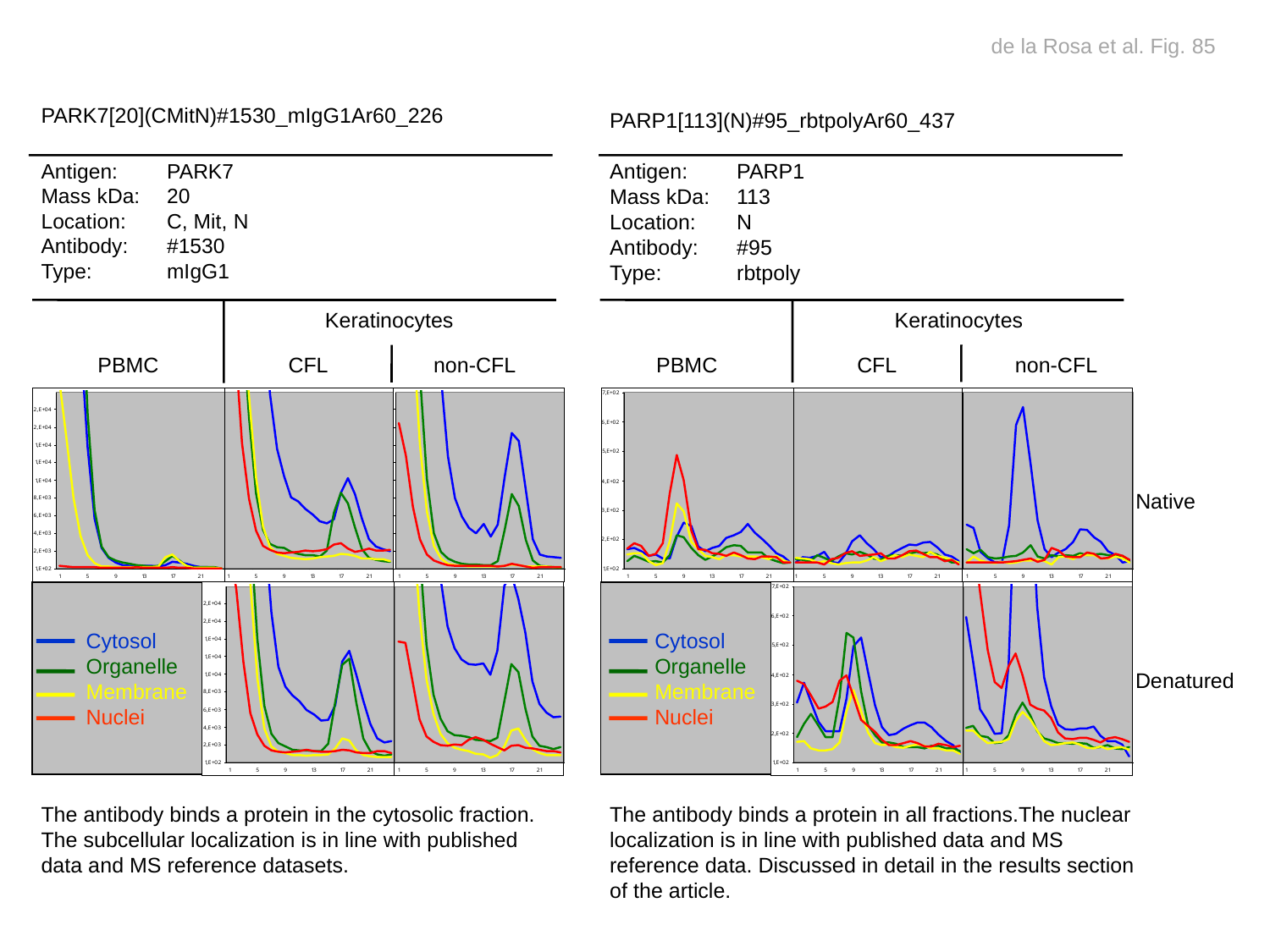

de la Rosa et al. Fig. <number>
# PARK7[20](CMitN)#1530_mIgG1Ar60_226
PARP1[113](N)#95_rbtpolyAr60_437
Antigen: 	PARK7
Mass kDa:	20
Location: 	C, Mit, N
Antibody: 	#1530
Type:	mIgG1
Antigen: 	PARP1
Mass kDa:	113
Location: 	N
Antibody: 	#95
Type:	rbtpoly
The antibody binds a protein in the cytosolic fraction. The subcellular localization is in line with published data and MS reference datasets.
The antibody binds a protein in all fractions.The nuclear localization is in line with published data and MS reference data. Discussed in detail in the results section of the article.

## Slide 86
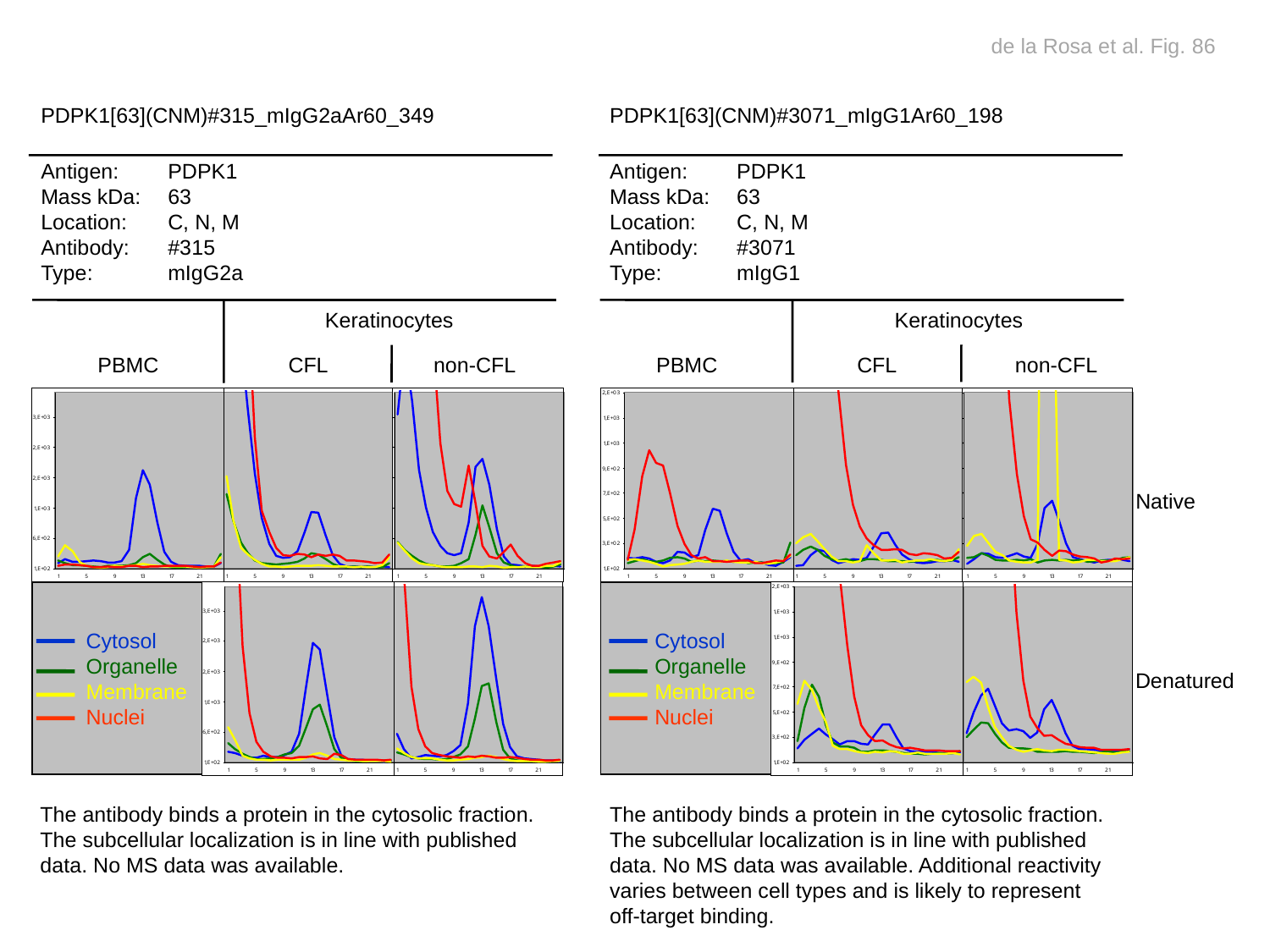

de la Rosa et al. Fig. <number>
PDPK1[63](CNM)#315_mIgG2aAr60_349
# PDPK1[63](CNM)#3071_mIgG1Ar60_198
Antigen: 	PDPK1
Mass kDa:	63
Location: 	C, N, M
Antibody: 	#315
Type:	mIgG2a
Antigen: 	PDPK1
Mass kDa:	63
Location: 	C, N, M
Antibody: 	#3071
Type:	mIgG1
The antibody binds a protein in the cytosolic fraction. The subcellular localization is in line with published data. No MS data was available.
The antibody binds a protein in the cytosolic fraction. The subcellular localization is in line with published data. No MS data was available. Additional reactivity varies between cell types and is likely to represent off-target binding.

## Slide 87
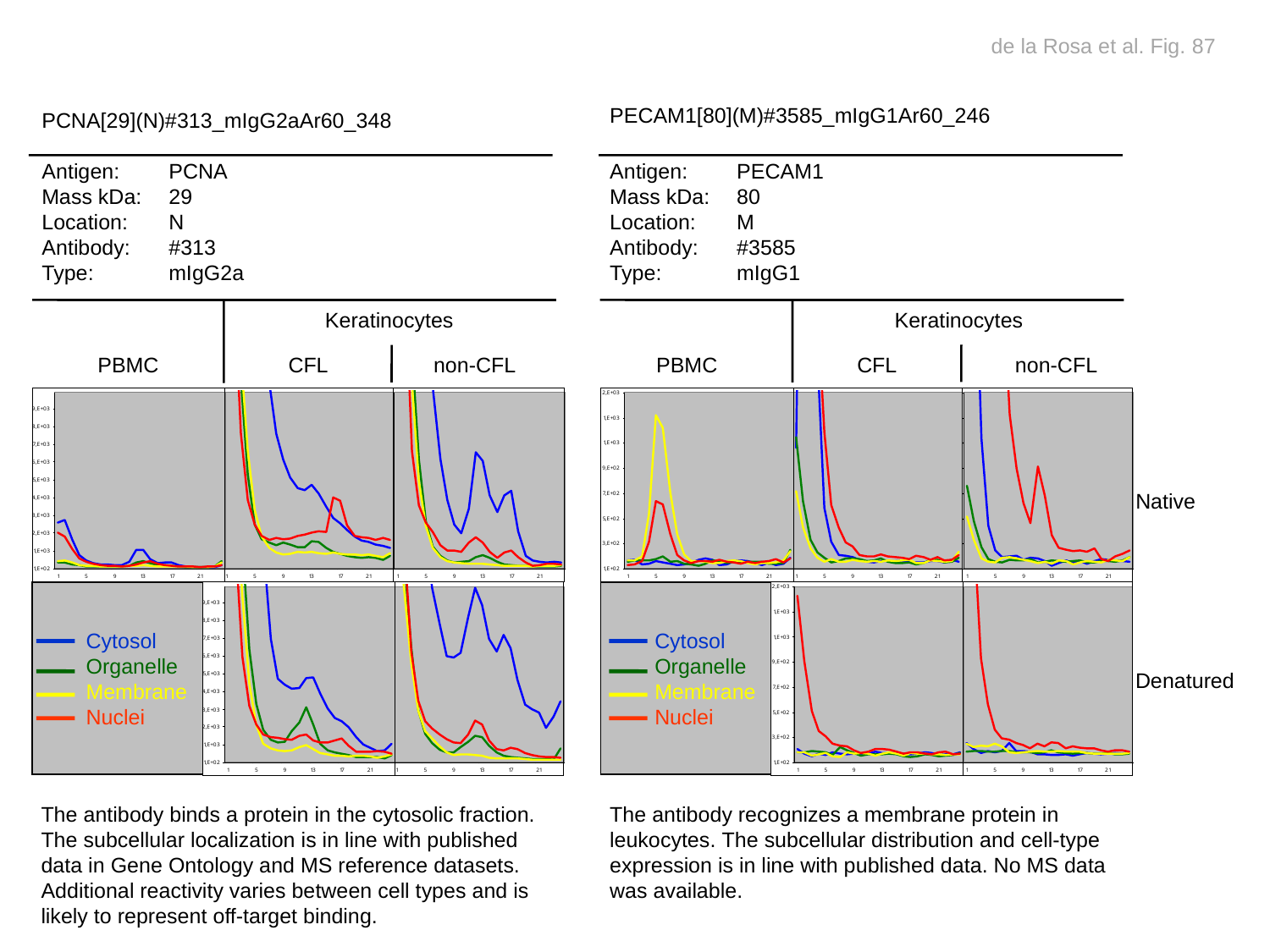

de la Rosa et al. Fig. <number>
PCNA[29](N)#313_mIgG2aAr60_348
# PECAM1[80](M)#3585_mIgG1Ar60_246
Antigen: 	PCNA
Mass kDa:	29
Location: 	N
Antibody: 	#313
Type:	mIgG2a
Antigen: 	PECAM1
Mass kDa:	80
Location: 	M
Antibody: 	#3585
Type:	mIgG1
The antibody binds a protein in the cytosolic fraction. The subcellular localization is in line with published data in Gene Ontology and MS reference datasets. Additional reactivity varies between cell types and is likely to represent off-target binding.
The antibody recognizes a membrane protein in leukocytes. The subcellular distribution and cell-type expression is in line with published data. No MS data was available.

## Slide 88
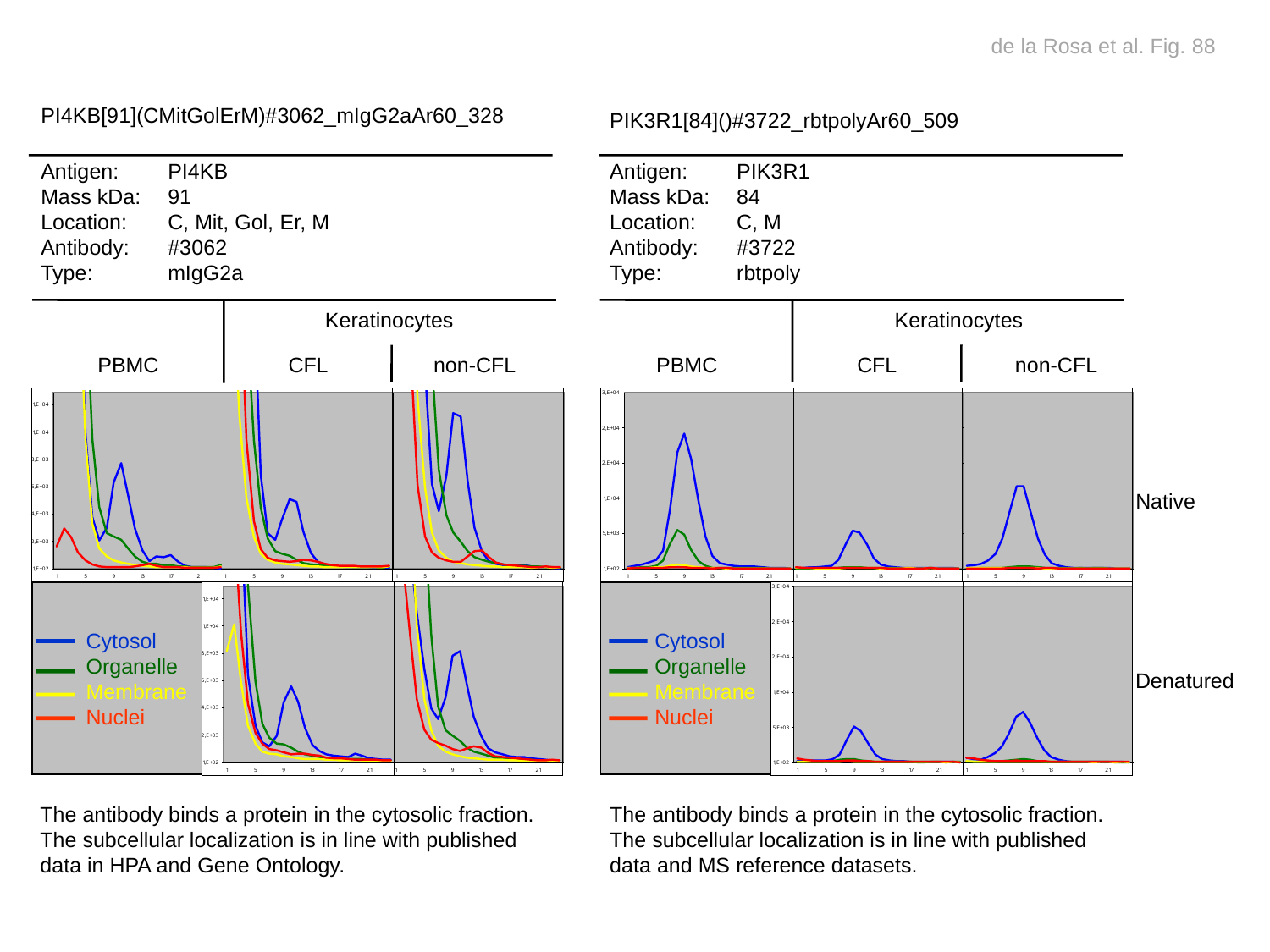

de la Rosa et al. Fig. <number>
# PI4KB[91](CMitGolErM)#3062_mIgG2aAr60_328
PIK3R1[84]()#3722_rbtpolyAr60_509
Antigen: 	PI4KB
Mass kDa:	91
Location: 	C, Mit, Gol, Er, M
Antibody: 	#3062
Type:	mIgG2a
Antigen: 	PIK3R1
Mass kDa:	84
Location: 	C, M
Antibody: 	#3722
Type:	rbtpoly
The antibody binds a protein in the cytosolic fraction. The subcellular localization is in line with published data in HPA and Gene Ontology.
The antibody binds a protein in the cytosolic fraction. The subcellular localization is in line with published data and MS reference datasets.

## Slide 89
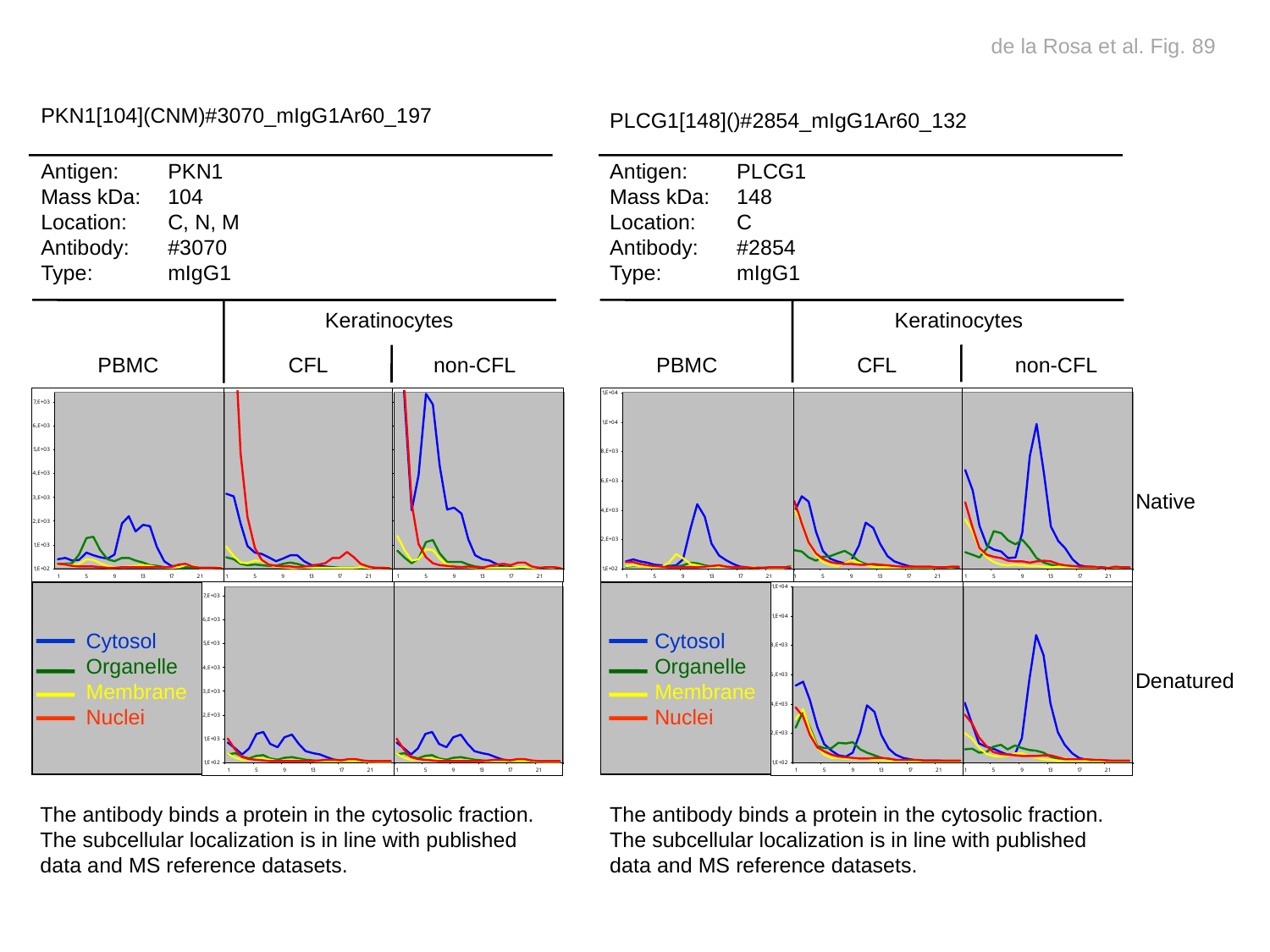

de la Rosa et al. Fig. <number>
# PKN1[104](CNM)#3070_mIgG1Ar60_197
PLCG1[148]()#2854_mIgG1Ar60_132
Antigen: 	PKN1
Mass kDa:	104
Location: 	C, N, M
Antibody: 	#3070
Type:	mIgG1
Antigen: 	PLCG1
Mass kDa:	148
Location: 	C
Antibody: 	#2854
Type:	mIgG1
The antibody binds a protein in the cytosolic fraction. The subcellular localization is in line with published data and MS reference datasets.
The antibody binds a protein in the cytosolic fraction. The subcellular localization is in line with published data and MS reference datasets.

## Slide 90
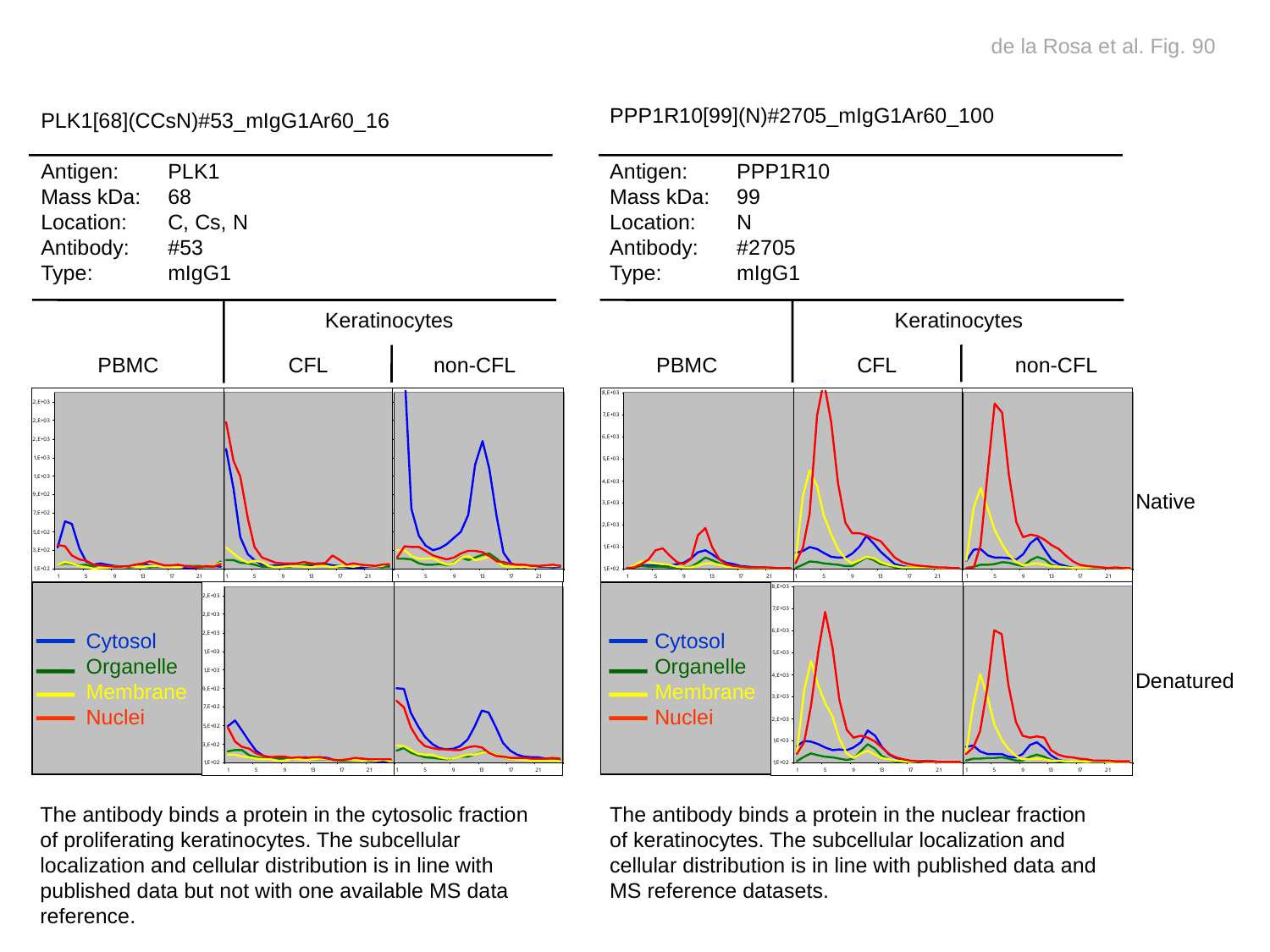

de la Rosa et al. Fig. <number>
# PLK1[68](CCsN)#53_mIgG1Ar60_16
PPP1R10[99](N)#2705_mIgG1Ar60_100
Antigen: 	PLK1
Mass kDa:	68
Location: 	C, Cs, N
Antibody: 	#53
Type:	mIgG1
Antigen: 	PPP1R10
Mass kDa:	99
Location: 	N
Antibody: 	#2705
Type:	mIgG1
The antibody binds a protein in the cytosolic fraction of proliferating keratinocytes. The subcellular localization and cellular distribution is in line with published data but not with one available MS data reference.
The antibody binds a protein in the nuclear fraction of keratinocytes. The subcellular localization and cellular distribution is in line with published data and MS reference datasets.

## Slide 91
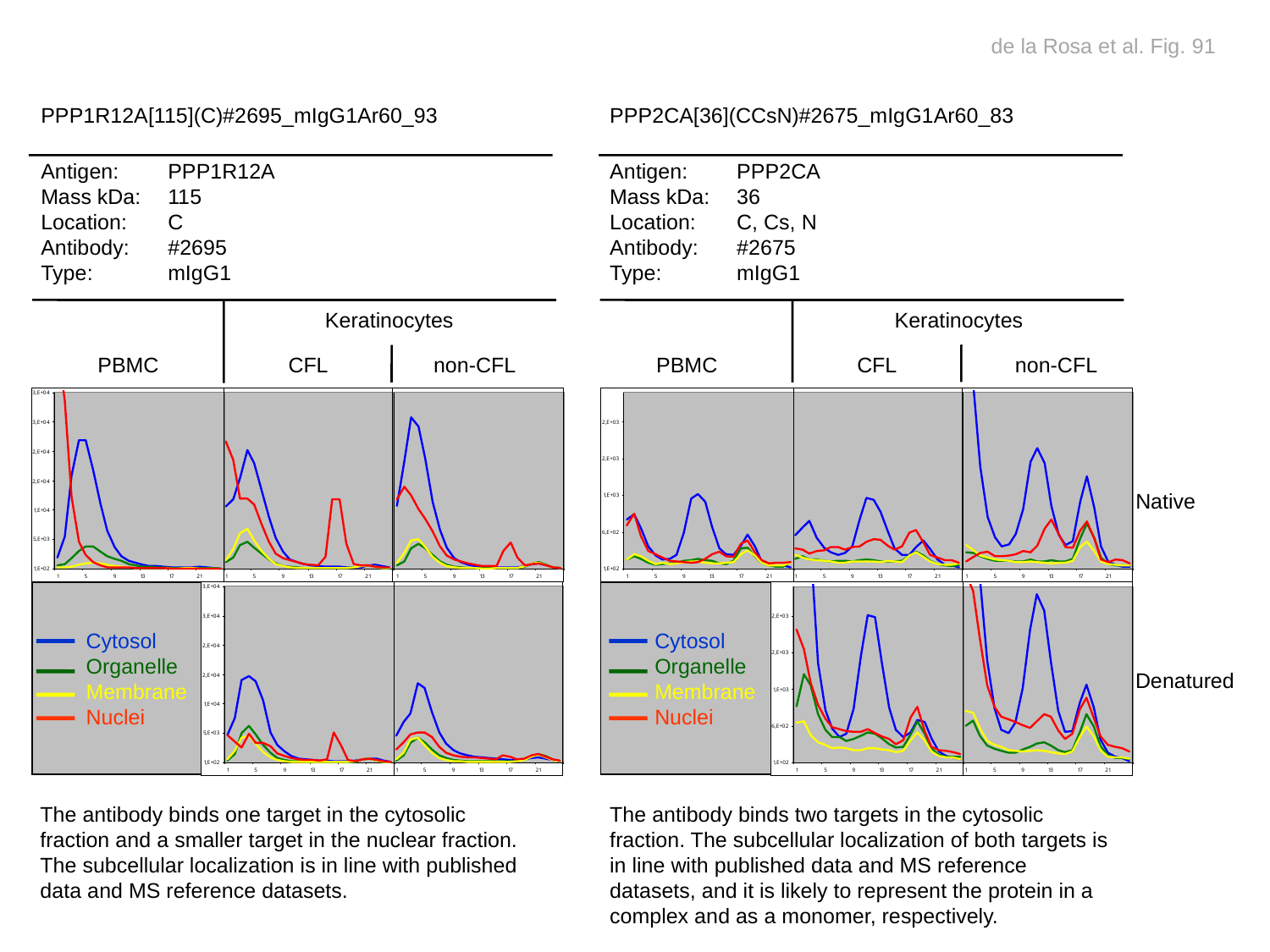

de la Rosa et al. Fig. <number>
# PPP1R12A[115](C)#2695_mIgG1Ar60_93
PPP2CA[36](CCsN)#2675_mIgG1Ar60_83
Antigen: 	PPP1R12A
Mass kDa:	115
Location: 	C
Antibody: 	#2695
Type:	mIgG1
Antigen: 	PPP2CA
Mass kDa:	36
Location: 	C, Cs, N
Antibody: 	#2675
Type:	mIgG1
The antibody binds one target in the cytosolic fraction and a smaller target in the nuclear fraction. The subcellular localization is in line with published data and MS reference datasets.
The antibody binds two targets in the cytosolic fraction. The subcellular localization of both targets is in line with published data and MS reference datasets, and it is likely to represent the protein in a complex and as a monomer, respectively.

## Slide 92
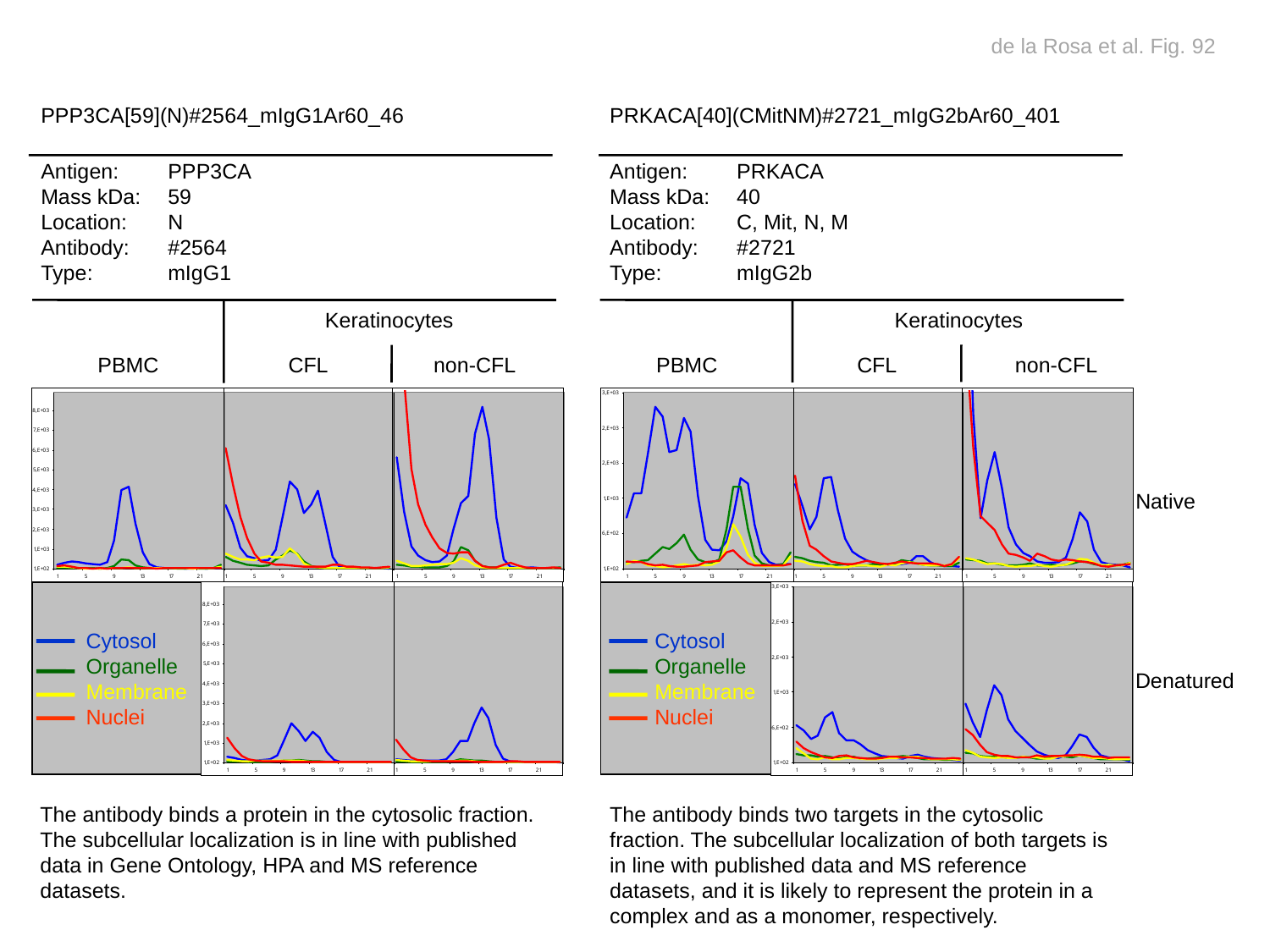

de la Rosa et al. Fig. <number>
# PPP3CA[59](N)#2564_mIgG1Ar60_46
PRKACA[40](CMitNM)#2721_mIgG2bAr60_401
Antigen: 	PPP3CA
Mass kDa:	59
Location: 	N
Antibody: 	#2564
Type:	mIgG1
Antigen: 	PRKACA
Mass kDa:	40
Location: 	C, Mit, N, M
Antibody: 	#2721
Type:	mIgG2b
The antibody binds a protein in the cytosolic fraction. The subcellular localization is in line with published data in Gene Ontology, HPA and MS reference datasets.
The antibody binds two targets in the cytosolic fraction. The subcellular localization of both targets is in line with published data and MS reference datasets, and it is likely to represent the protein in a complex and as a monomer, respectively.

## Slide 93
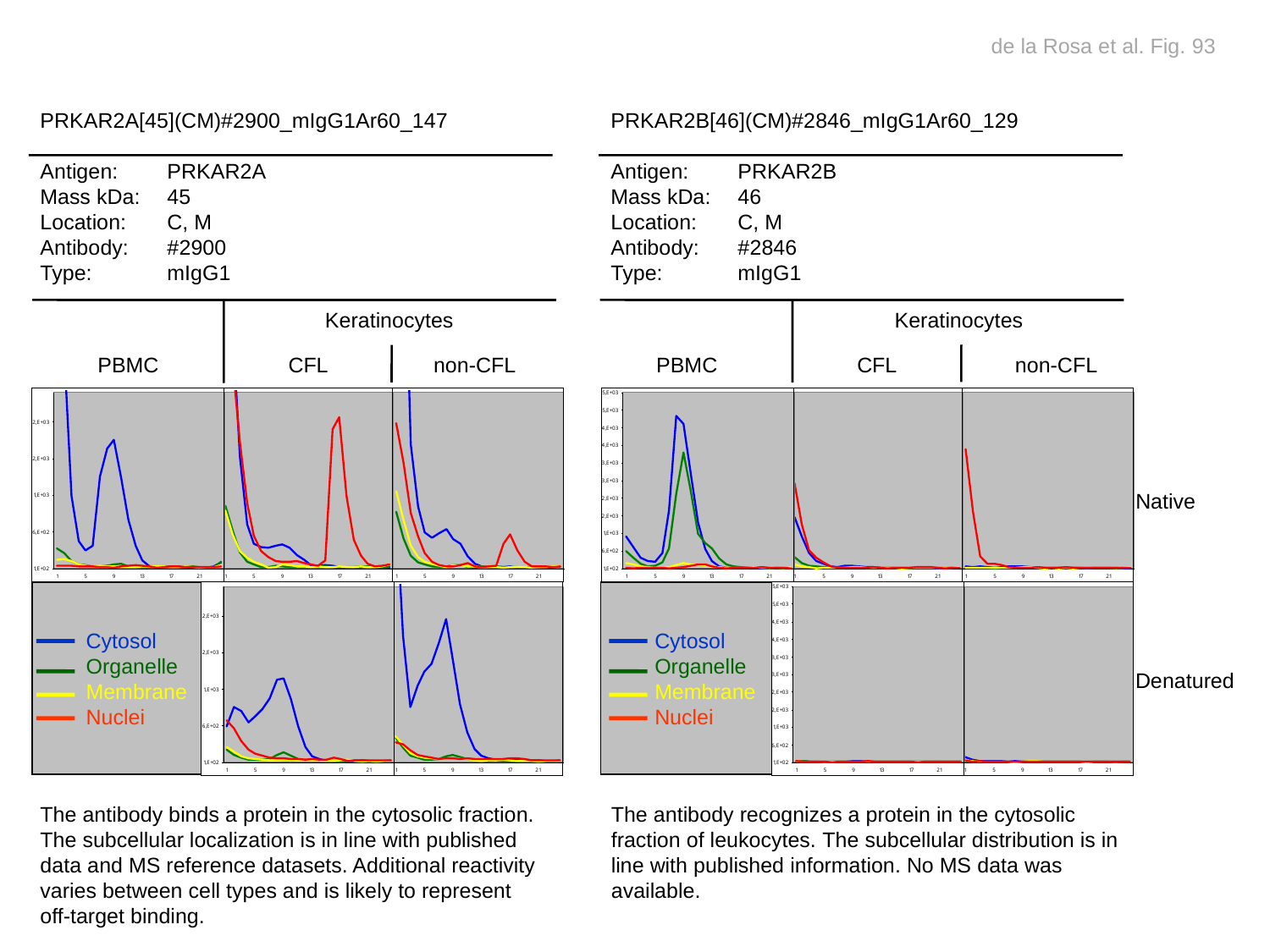

de la Rosa et al. Fig. <number>
# PRKAR2A[45](CM)#2900_mIgG1Ar60_147
PRKAR2B[46](CM)#2846_mIgG1Ar60_129
Antigen: 	PRKAR2A
Mass kDa:	45
Location: 	C, M
Antibody: 	#2900
Type:	mIgG1
Antigen: 	PRKAR2B
Mass kDa:	46
Location: 	C, M
Antibody: 	#2846
Type:	mIgG1
The antibody binds a protein in the cytosolic fraction. The subcellular localization is in line with published data and MS reference datasets. Additional reactivity varies between cell types and is likely to represent off-target binding.
The antibody recognizes a protein in the cytosolic fraction of leukocytes. The subcellular distribution is in line with published information. No MS data was available.

## Slide 94
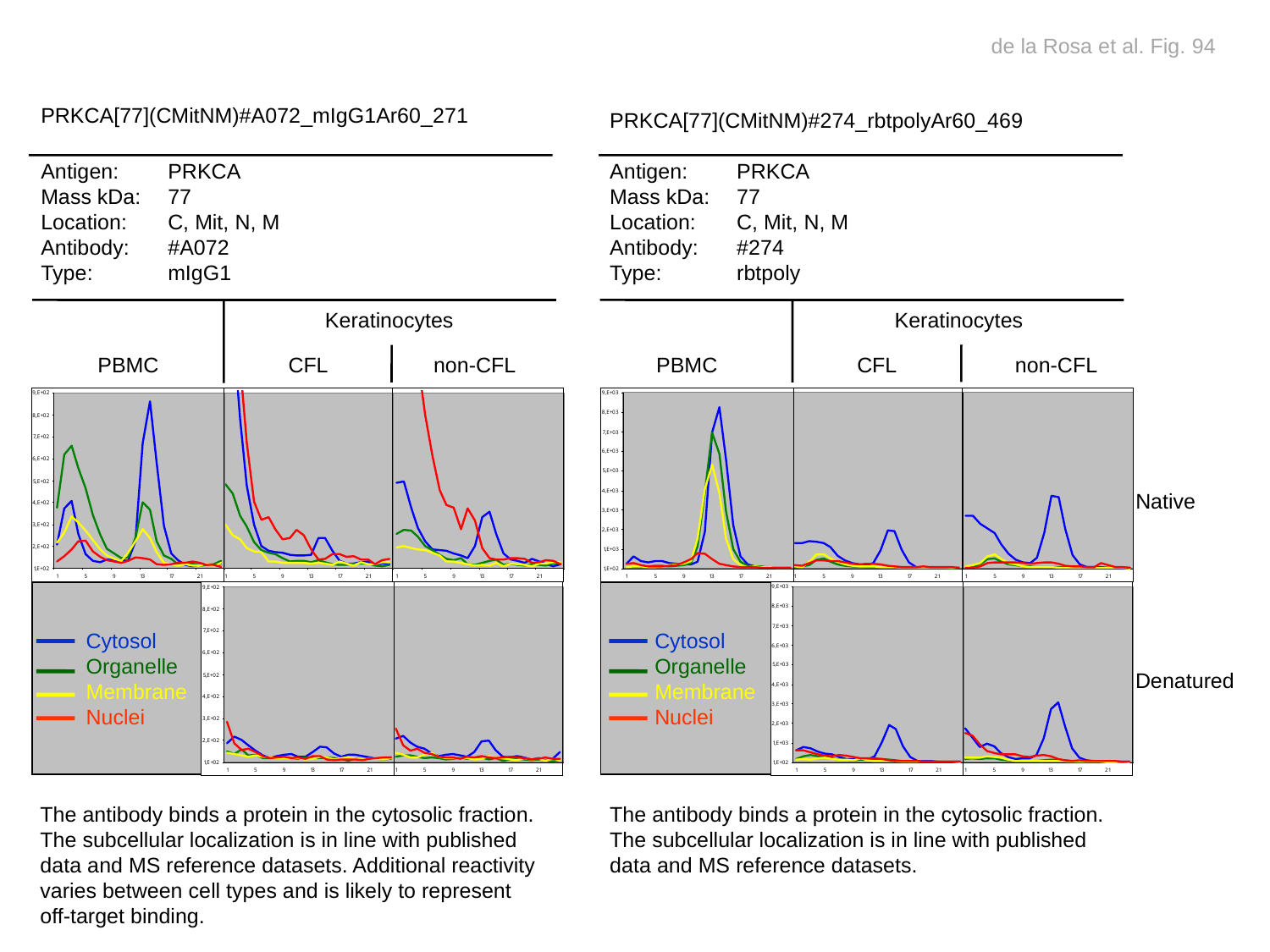

de la Rosa et al. Fig. <number>
PRKCA[77](CMitNM)#A072_mIgG1Ar60_271
# PRKCA[77](CMitNM)#274_rbtpolyAr60_469
Antigen: 	PRKCA
Mass kDa:	77
Location: 	C, Mit, N, M
Antibody: 	#A072
Type:	mIgG1
Antigen: 	PRKCA
Mass kDa:	77
Location: 	C, Mit, N, M
Antibody: 	#274
Type:	rbtpoly
The antibody binds a protein in the cytosolic fraction. The subcellular localization is in line with published data and MS reference datasets. Additional reactivity varies between cell types and is likely to represent off-target binding.
The antibody binds a protein in the cytosolic fraction. The subcellular localization is in line with published data and MS reference datasets.

## Slide 95
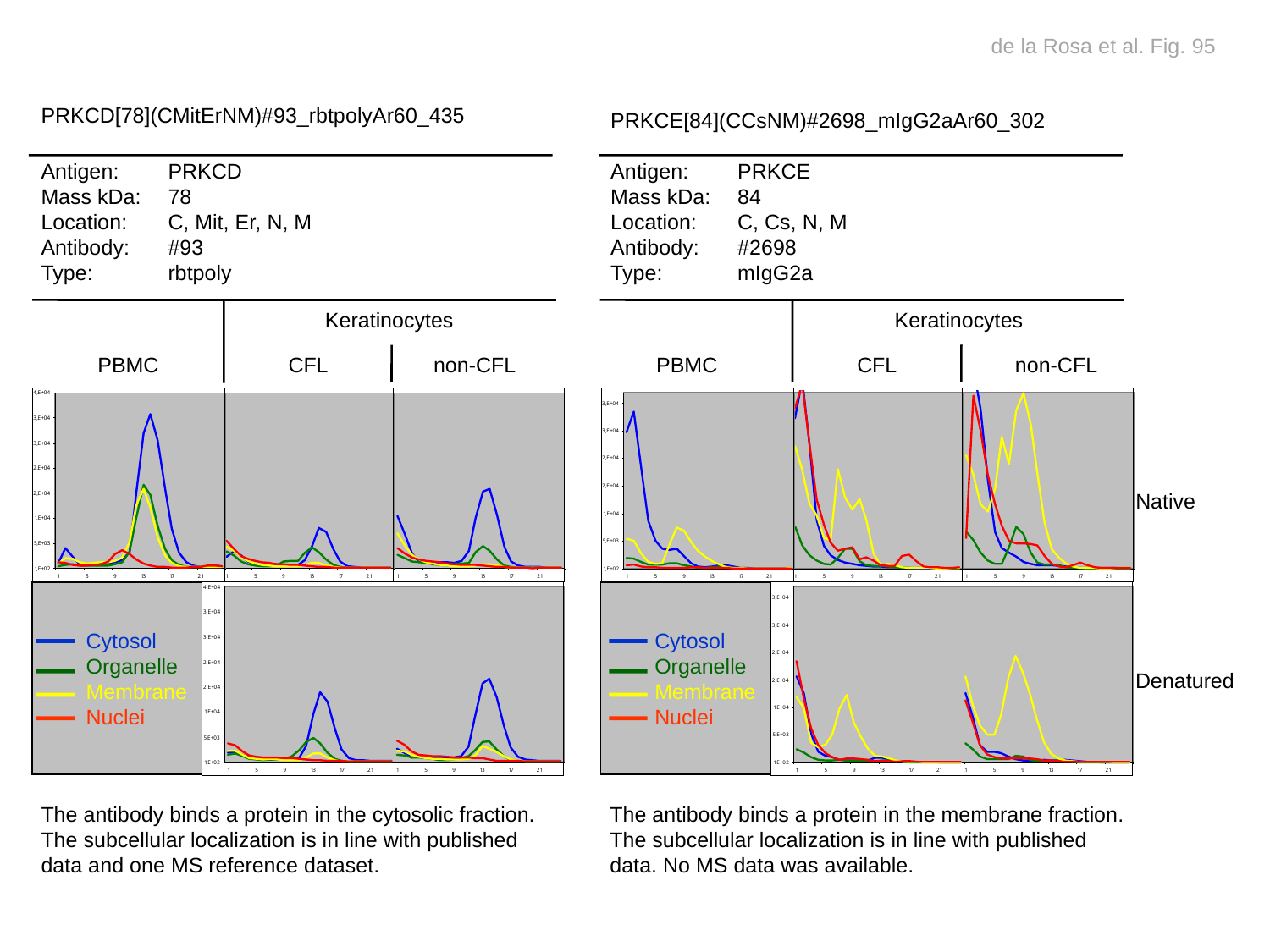

de la Rosa et al. Fig. <number>
# PRKCD[78](CMitErNM)#93_rbtpolyAr60_435
PRKCE[84](CCsNM)#2698_mIgG2aAr60_302
Antigen: 	PRKCD
Mass kDa:	78
Location: 	C, Mit, Er, N, M
Antibody: 	#93
Type:	rbtpoly
Antigen: 	PRKCE
Mass kDa:	84
Location: 	C, Cs, N, M
Antibody: 	#2698
Type:	mIgG2a
The antibody binds a protein in the cytosolic fraction. The subcellular localization is in line with published data and one MS reference dataset.
The antibody binds a protein in the membrane fraction. The subcellular localization is in line with published data. No MS data was available.

## Slide 96
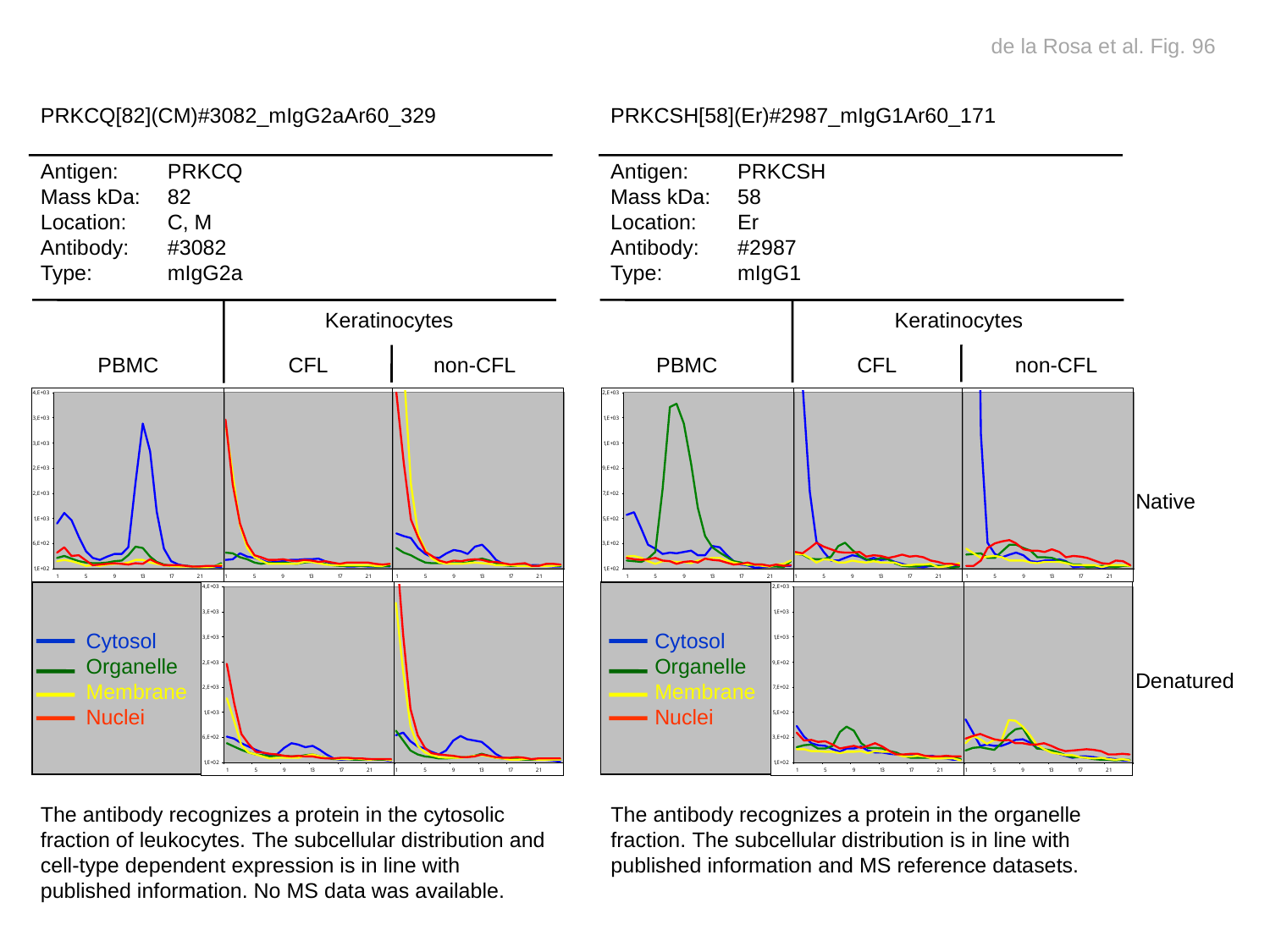

de la Rosa et al. Fig. <number>
# PRKCQ[82](CM)#3082_mIgG2aAr60_329
PRKCSH[58](Er)#2987_mIgG1Ar60_171
Antigen: 	PRKCSH
Mass kDa:	58
Location: 	Er
Antibody: 	#2987
Type:	mIgG1
Antigen: 	PRKCQ
Mass kDa:	82
Location: 	C, M
Antibody: 	#3082
Type:	mIgG2a
The antibody recognizes a protein in the cytosolic fraction of leukocytes. The subcellular distribution and cell-type dependent expression is in line with published information. No MS data was available.
The antibody recognizes a protein in the organelle fraction. The subcellular distribution is in line with published information and MS reference datasets.

## Slide 97
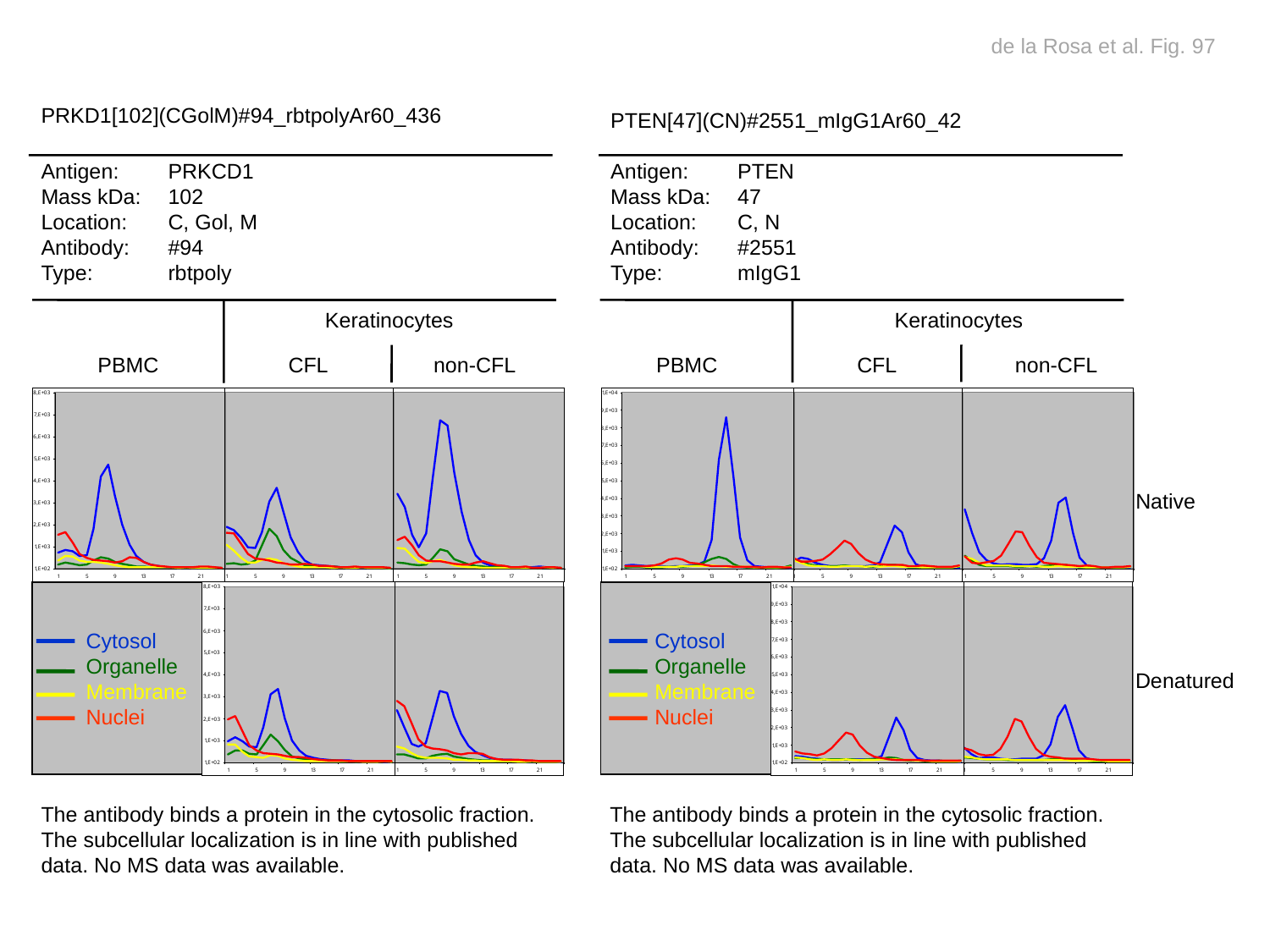

de la Rosa et al. Fig. <number>
# PRKD1[102](CGolM)#94_rbtpolyAr60_436
PTEN[47](CN)#2551_mIgG1Ar60_42
Antigen: 	PRKCD1
Mass kDa:	102
Location: 	C, Gol, M
Antibody: 	#94
Type:	rbtpoly
Antigen: 	PTEN
Mass kDa:	47
Location: 	C, N
Antibody: 	#2551
Type:	mIgG1
The antibody binds a protein in the cytosolic fraction. The subcellular localization is in line with published data. No MS data was available.
The antibody binds a protein in the cytosolic fraction. The subcellular localization is in line with published data. No MS data was available.

## Slide 98
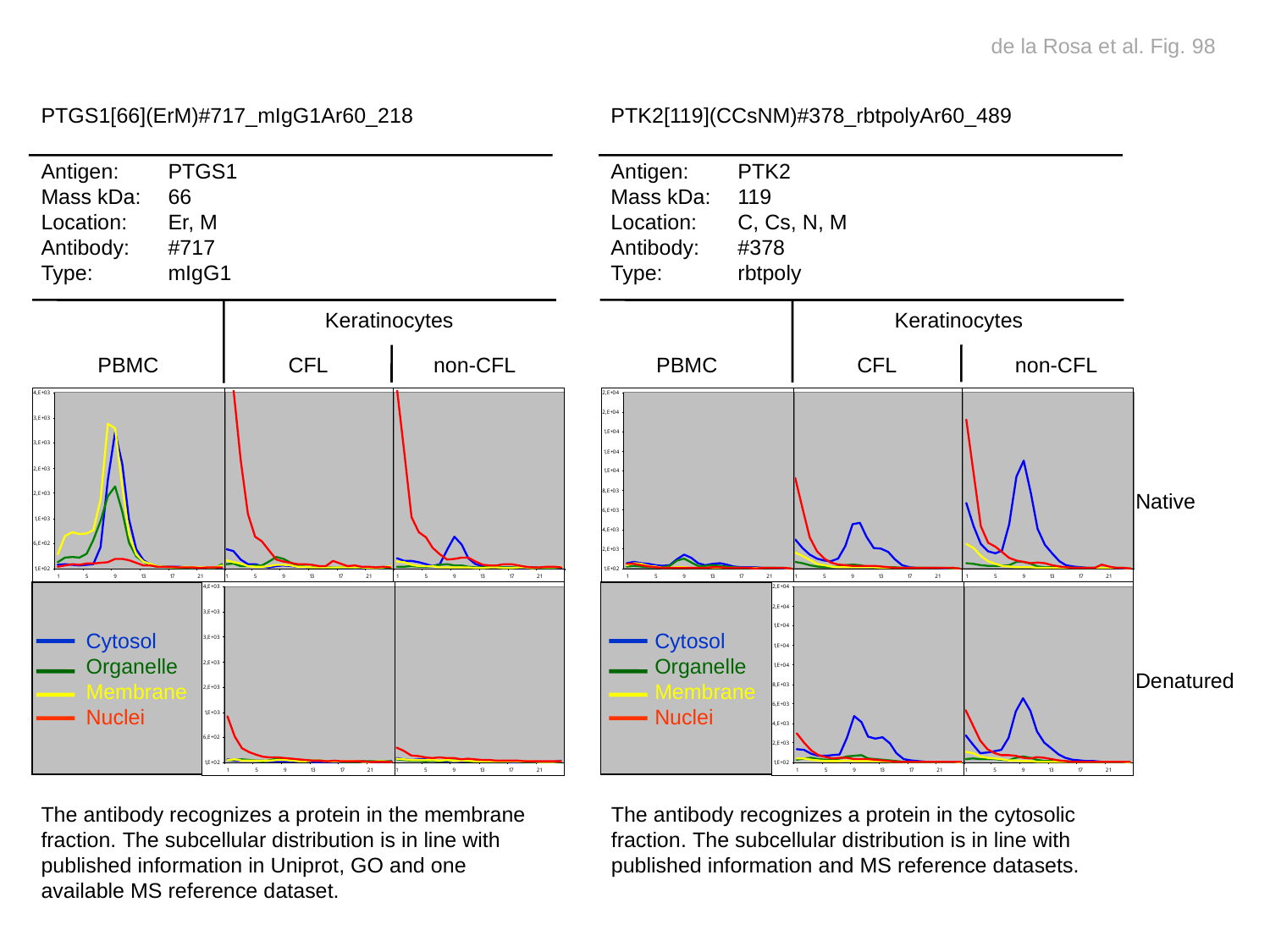

de la Rosa et al. Fig. <number>
# PTGS1[66](ErM)#717_mIgG1Ar60_218
PTK2[119](CCsNM)#378_rbtpolyAr60_489
Antigen: 	PTGS1
Mass kDa:	66
Location: 	Er, M
Antibody: 	#717
Type:	mIgG1
Antigen: 	PTK2
Mass kDa:	119
Location: 	C, Cs, N, M
Antibody: 	#378
Type:	rbtpoly
The antibody recognizes a protein in the membrane fraction. The subcellular distribution is in line with published information in Uniprot, GO and one available MS reference dataset.
The antibody recognizes a protein in the cytosolic fraction. The subcellular distribution is in line with published information and MS reference datasets.

## Slide 99
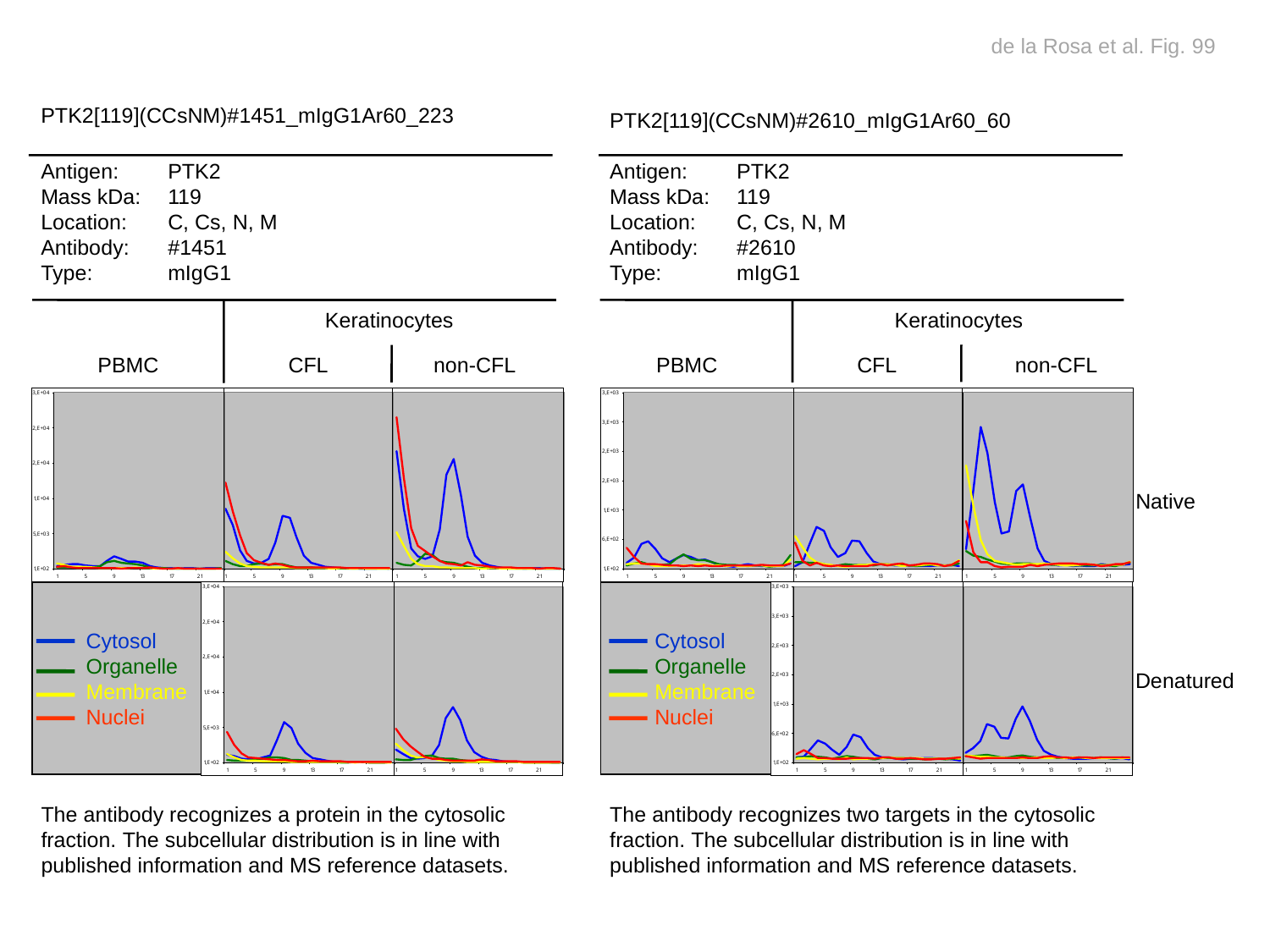

de la Rosa et al. Fig. <number>
# PTK2[119](CCsNM)#1451_mIgG1Ar60_223
PTK2[119](CCsNM)#2610_mIgG1Ar60_60
Antigen: 	PTK2
Mass kDa:	119
Location: 	C, Cs, N, M
Antibody: 	#1451
Type:	mIgG1
Antigen: 	PTK2
Mass kDa:	119
Location: 	C, Cs, N, M
Antibody: 	#2610
Type:	mIgG1
The antibody recognizes a protein in the cytosolic fraction. The subcellular distribution is in line with published information and MS reference datasets.
The antibody recognizes two targets in the cytosolic fraction. The subcellular distribution is in line with published information and MS reference datasets.

## Slide 100
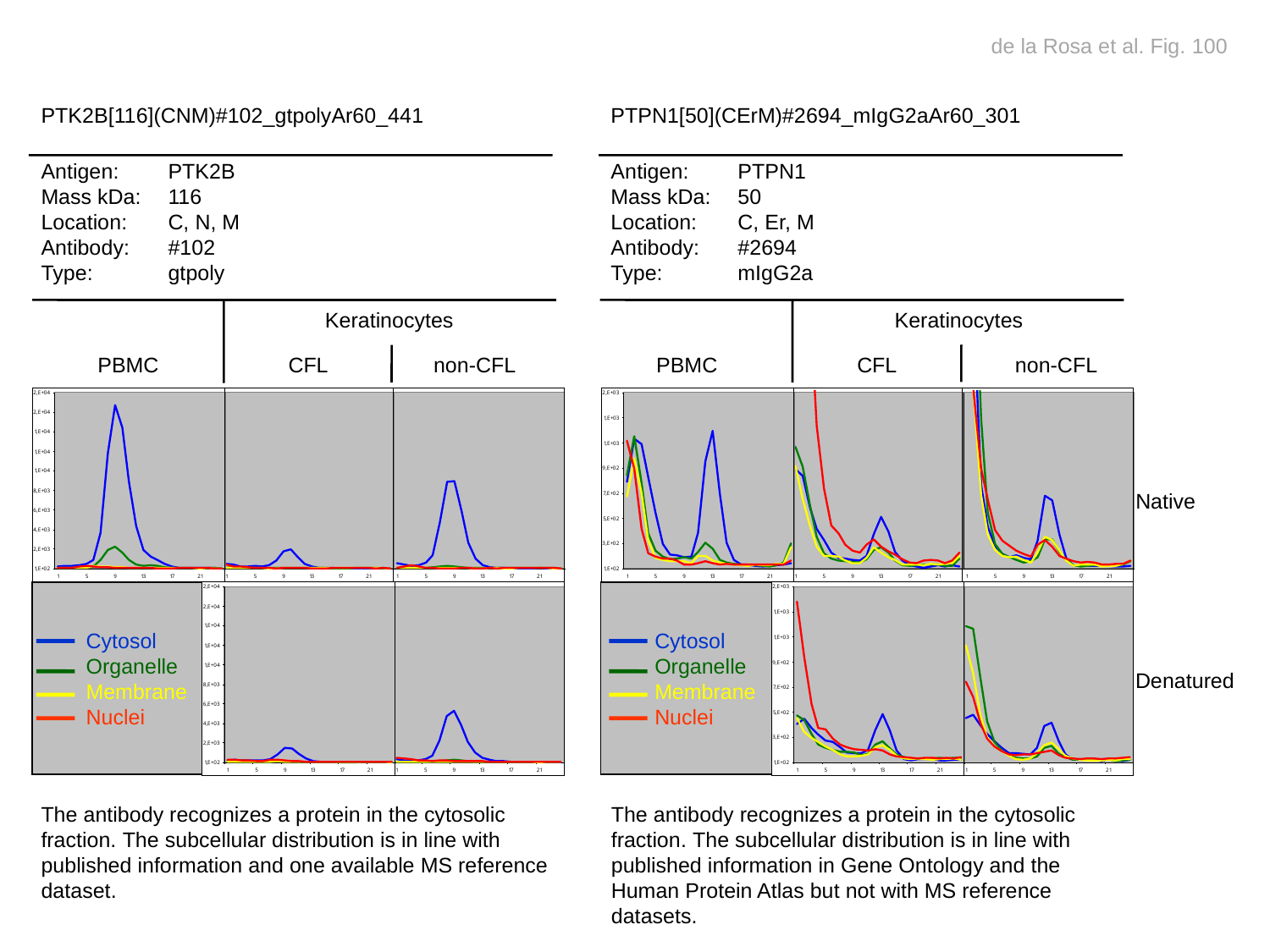

de la Rosa et al. Fig. <number>
# PTK2B[116](CNM)#102_gtpolyAr60_441
PTPN1[50](CErM)#2694_mIgG2aAr60_301
Antigen: 	PTK2B
Mass kDa:	116
Location: 	C, N, M
Antibody: 	#102
Type:	gtpoly
Antigen: 	PTPN1
Mass kDa:	50
Location: 	C, Er, M
Antibody: 	#2694
Type:	mIgG2a
The antibody recognizes a protein in the cytosolic fraction. The subcellular distribution is in line with published information and one available MS reference dataset.
The antibody recognizes a protein in the cytosolic fraction. The subcellular distribution is in line with published information in Gene Ontology and the Human Protein Atlas but not with MS reference datasets.

## Slide 101
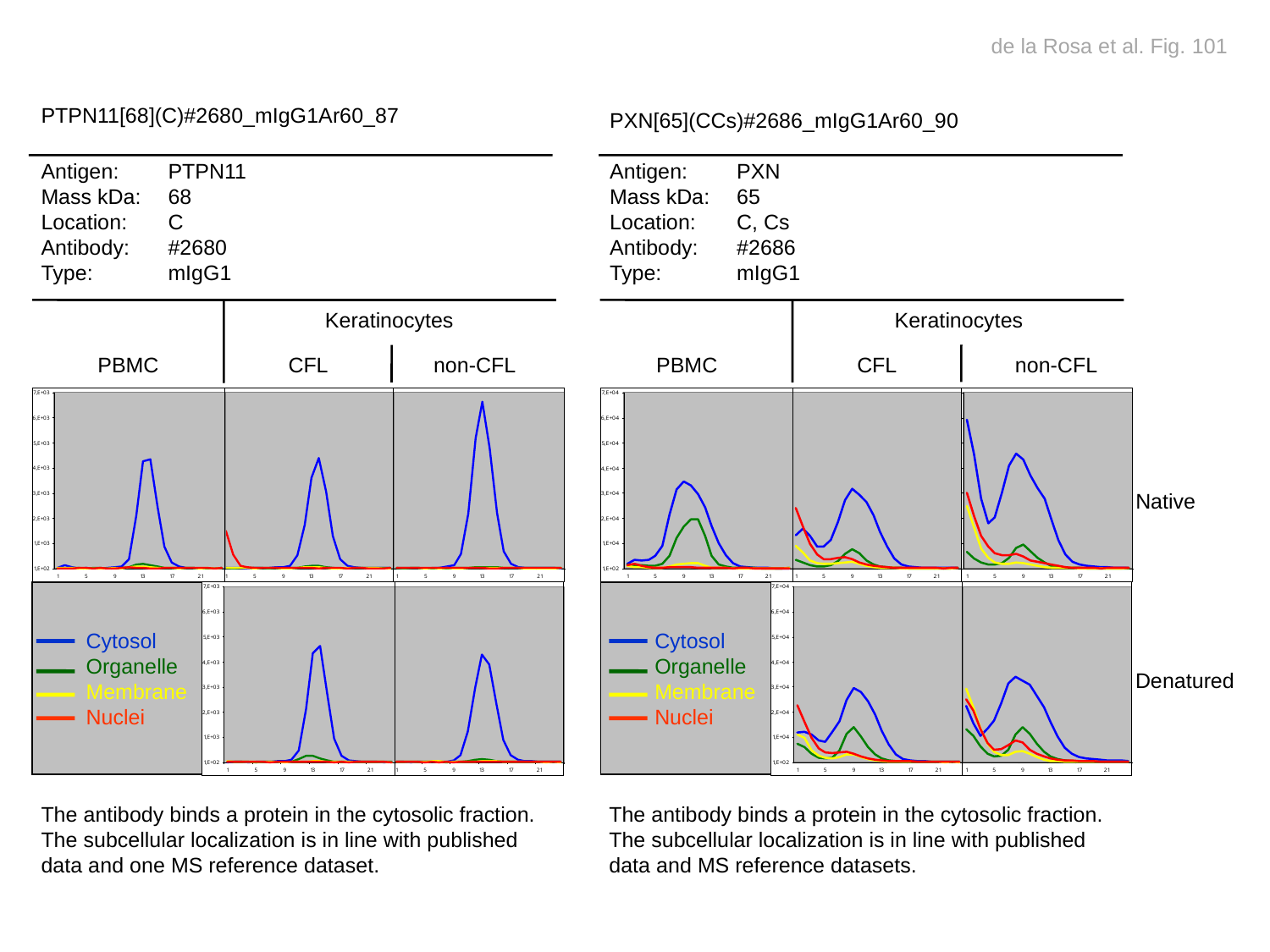

de la Rosa et al. Fig. <number>
# PTPN11[68](C)#2680_mIgG1Ar60_87
PXN[65](CCs)#2686_mIgG1Ar60_90
Antigen: 	PTPN11
Mass kDa:	68
Location: 	C
Antibody: 	#2680
Type:	mIgG1
Antigen: 	PXN
Mass kDa:	65
Location: 	C, Cs
Antibody: 	#2686
Type:	mIgG1
The antibody binds a protein in the cytosolic fraction. The subcellular localization is in line with published data and one MS reference dataset.
The antibody binds a protein in the cytosolic fraction. The subcellular localization is in line with published data and MS reference datasets.

## Slide 102
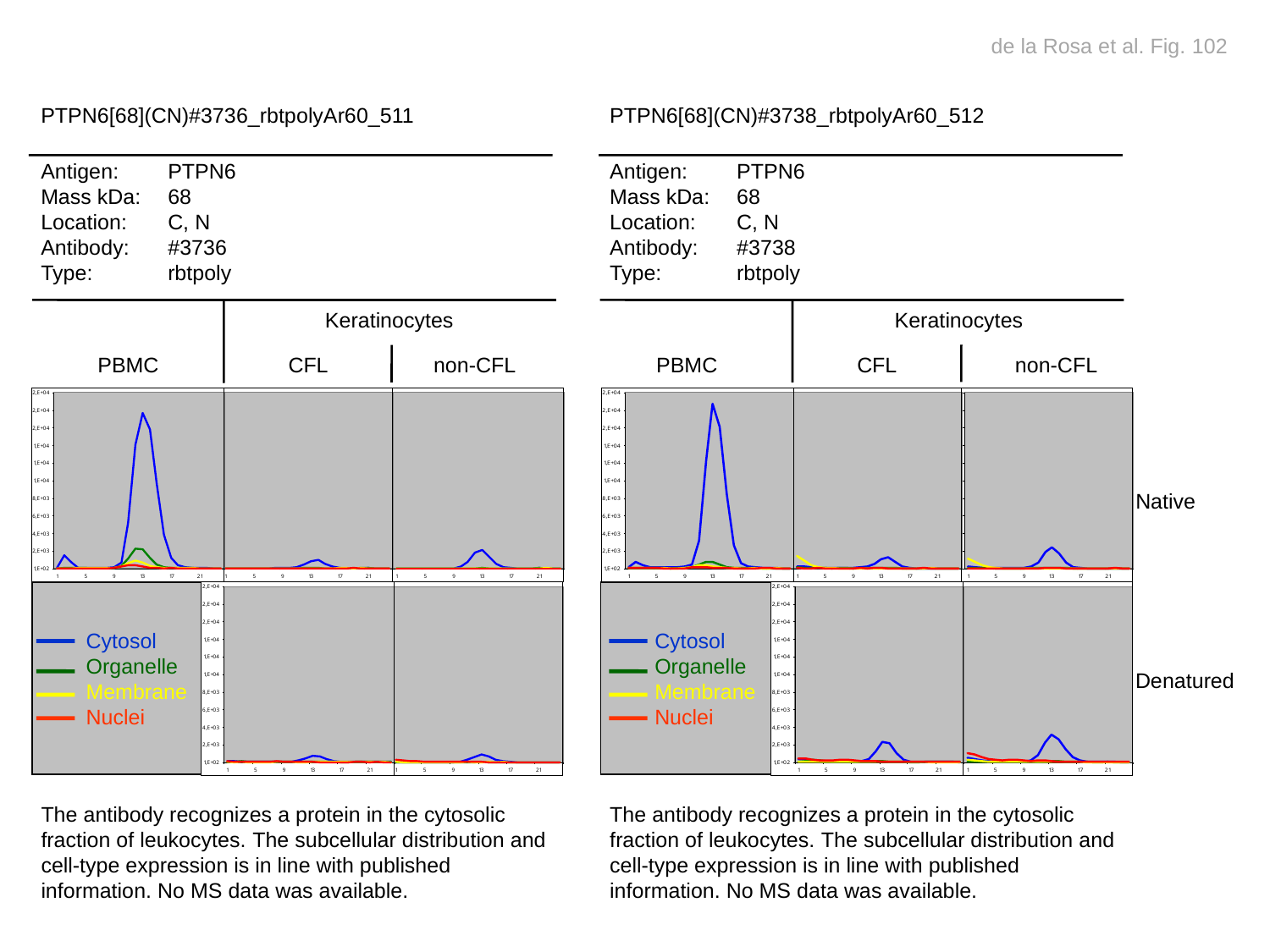

de la Rosa et al. Fig. <number>
# PTPN6[68](CN)#3736_rbtpolyAr60_511
PTPN6[68](CN)#3738_rbtpolyAr60_512
Antigen: 	PTPN6
Mass kDa:	68
Location: 	C, N
Antibody: 	#3736
Type:	rbtpoly
Antigen: 	PTPN6
Mass kDa:	68
Location: 	C, N
Antibody: 	#3738
Type:	rbtpoly
The antibody recognizes a protein in the cytosolic fraction of leukocytes. The subcellular distribution and cell-type expression is in line with published information. No MS data was available.
The antibody recognizes a protein in the cytosolic fraction of leukocytes. The subcellular distribution and cell-type expression is in line with published information. No MS data was available.

## Slide 103
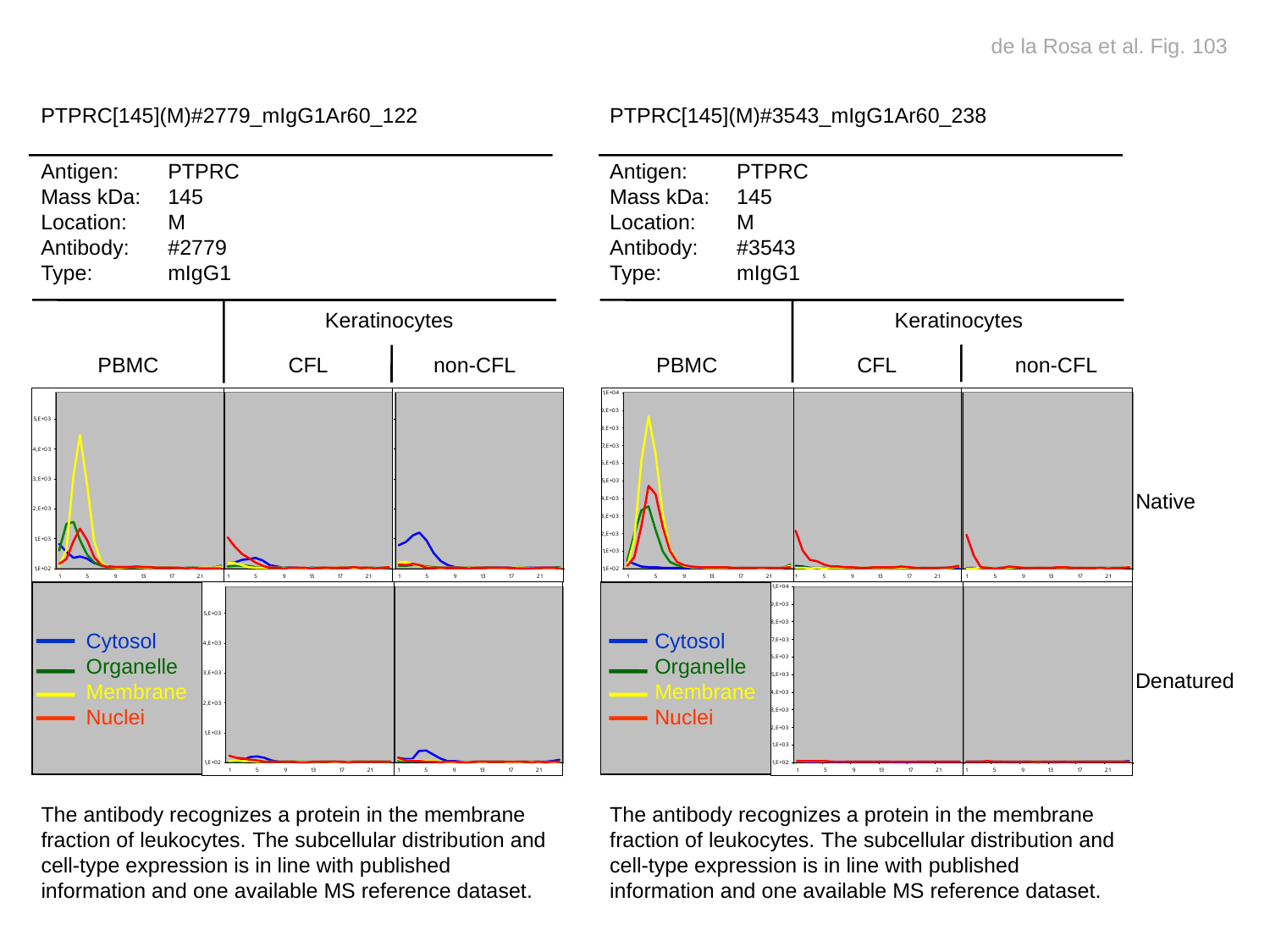

de la Rosa et al. Fig. <number>
# PTPRC[145](M)#2779_mIgG1Ar60_122
PTPRC[145](M)#3543_mIgG1Ar60_238
Antigen: 	PTPRC
Mass kDa:	145
Location: 	M
Antibody: 	#2779
Type:	mIgG1
Antigen: 	PTPRC
Mass kDa:	145
Location: 	M
Antibody: 	#3543
Type:	mIgG1
The antibody recognizes a protein in the membrane fraction of leukocytes. The subcellular distribution and cell-type expression is in line with published information and one available MS reference dataset.
The antibody recognizes a protein in the membrane fraction of leukocytes. The subcellular distribution and cell-type expression is in line with published information and one available MS reference dataset.

## Slide 104
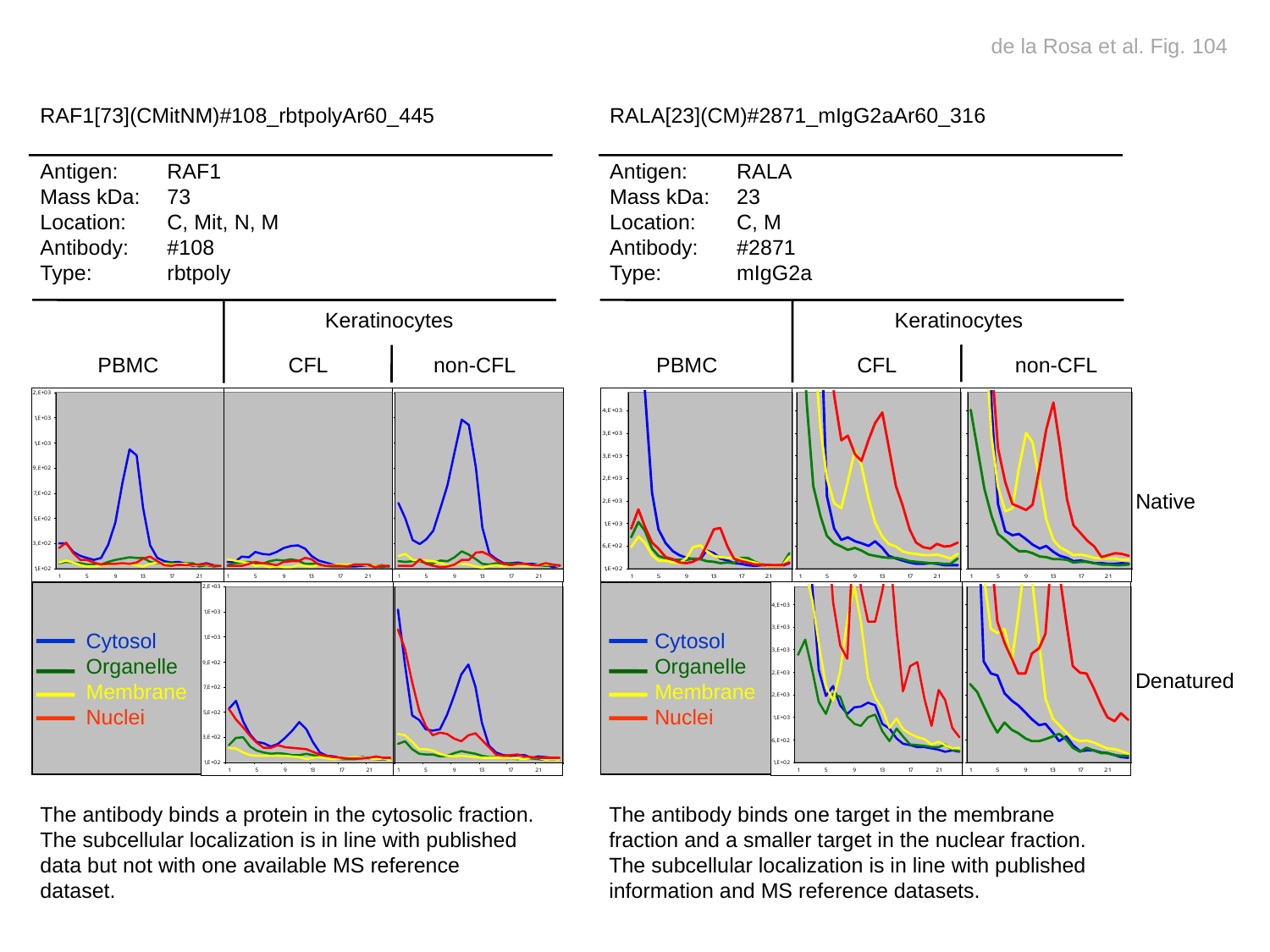

de la Rosa et al. Fig. <number>
# RAF1[73](CMitNM)#108_rbtpolyAr60_445
RALA[23](CM)#2871_mIgG2aAr60_316
Antigen: 	RAF1
Mass kDa:	73
Location: 	C, Mit, N, M
Antibody: 	#108
Type:	rbtpoly
Antigen: 	RALA
Mass kDa:	23
Location: 	C, M
Antibody: 	#2871
Type:	mIgG2a
The antibody binds a protein in the cytosolic fraction. The subcellular localization is in line with published data but not with one available MS reference dataset.
The antibody binds one target in the membrane fraction and a smaller target in the nuclear fraction. The subcellular localization is in line with published information and MS reference datasets.

## Slide 105
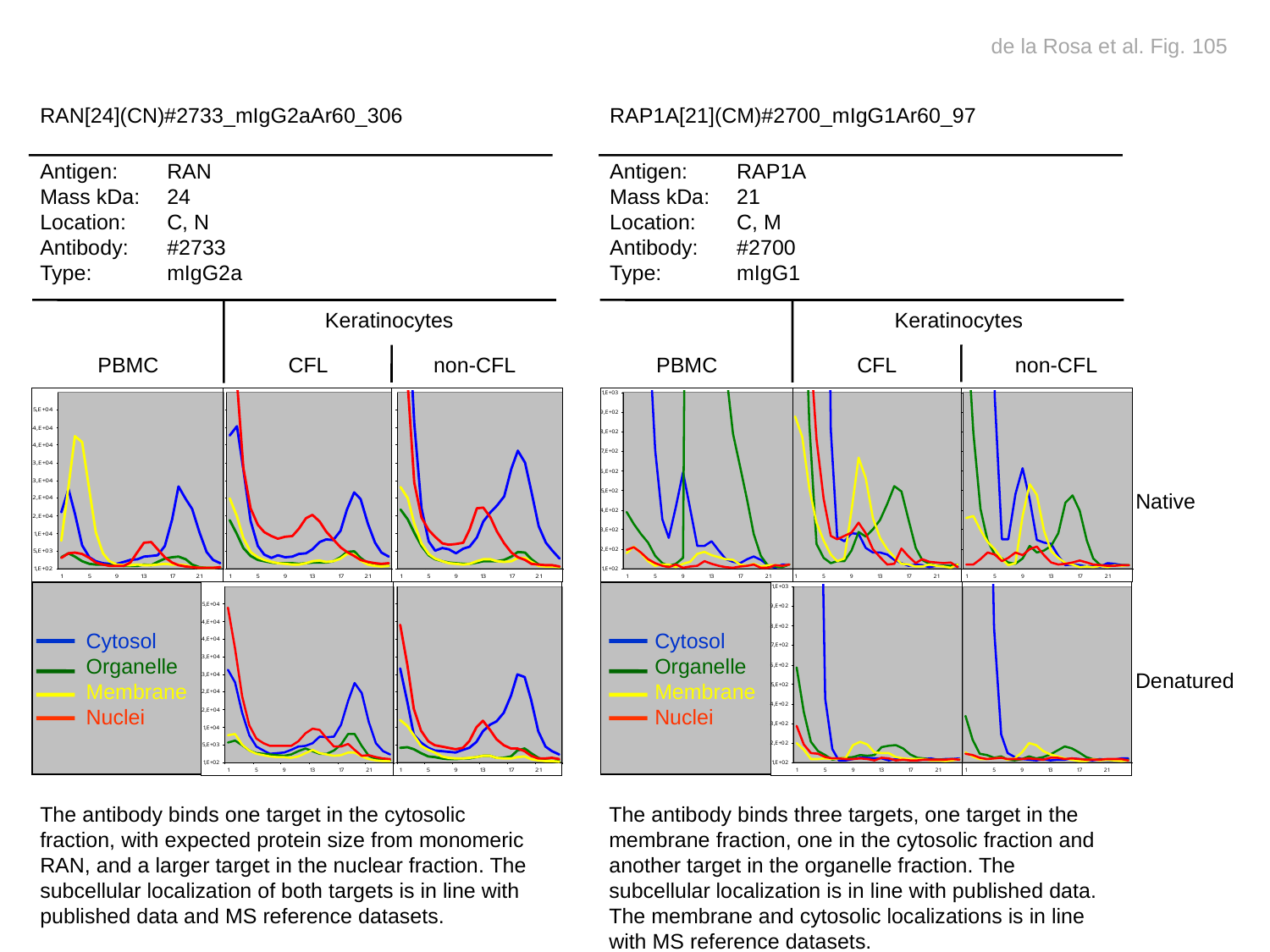

de la Rosa et al. Fig. <number>
# RAN[24](CN)#2733_mIgG2aAr60_306
RAP1A[21](CM)#2700_mIgG1Ar60_97
Antigen: 	RAN
Mass kDa:	24
Location: 	C, N
Antibody: 	#2733
Type:	mIgG2a
Antigen: 	RAP1A
Mass kDa:	21
Location: 	C, M
Antibody: 	#2700
Type:	mIgG1
The antibody binds one target in the cytosolic fraction, with expected protein size from monomeric RAN, and a larger target in the nuclear fraction. The subcellular localization of both targets is in line with published data and MS reference datasets.
The antibody binds three targets, one target in the membrane fraction, one in the cytosolic fraction and another target in the organelle fraction. The subcellular localization is in line with published data. The membrane and cytosolic localizations is in line with MS reference datasets.

## Slide 106
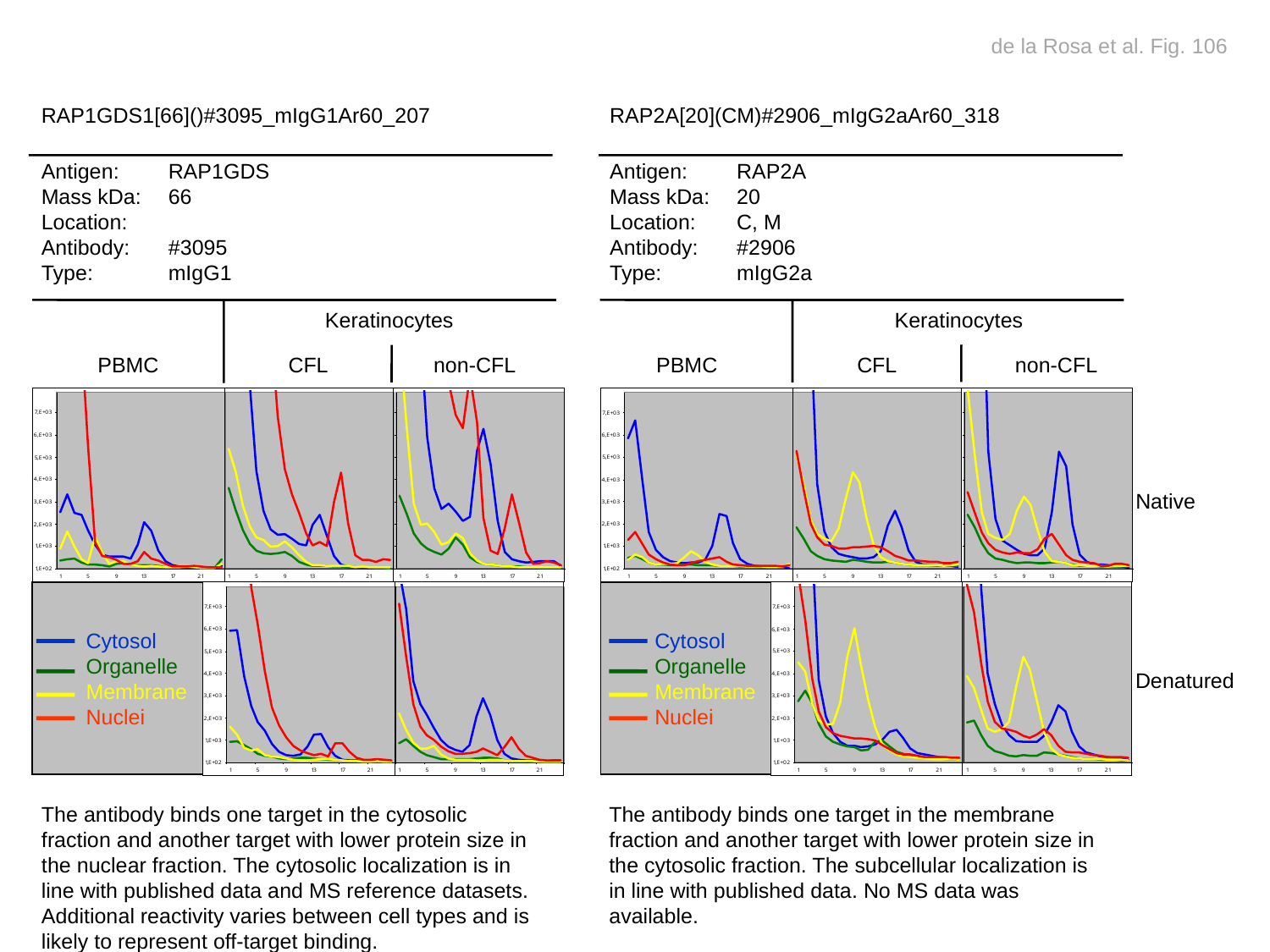

de la Rosa et al. Fig. <number>
# RAP1GDS1[66]()#3095_mIgG1Ar60_207
RAP2A[20](CM)#2906_mIgG2aAr60_318
Antigen: 	RAP1GDS
Mass kDa:	66
Location:
Antibody: 	#3095
Type:	mIgG1
Antigen: 	RAP2A
Mass kDa:	20
Location: 	C, M
Antibody: 	#2906
Type:	mIgG2a
The antibody binds one target in the cytosolic fraction and another target with lower protein size in the nuclear fraction. The cytosolic localization is in line with published data and MS reference datasets. Additional reactivity varies between cell types and is likely to represent off-target binding.
The antibody binds one target in the membrane fraction and another target with lower protein size in the cytosolic fraction. The subcellular localization is in line with published data. No MS data was available.

## Slide 107
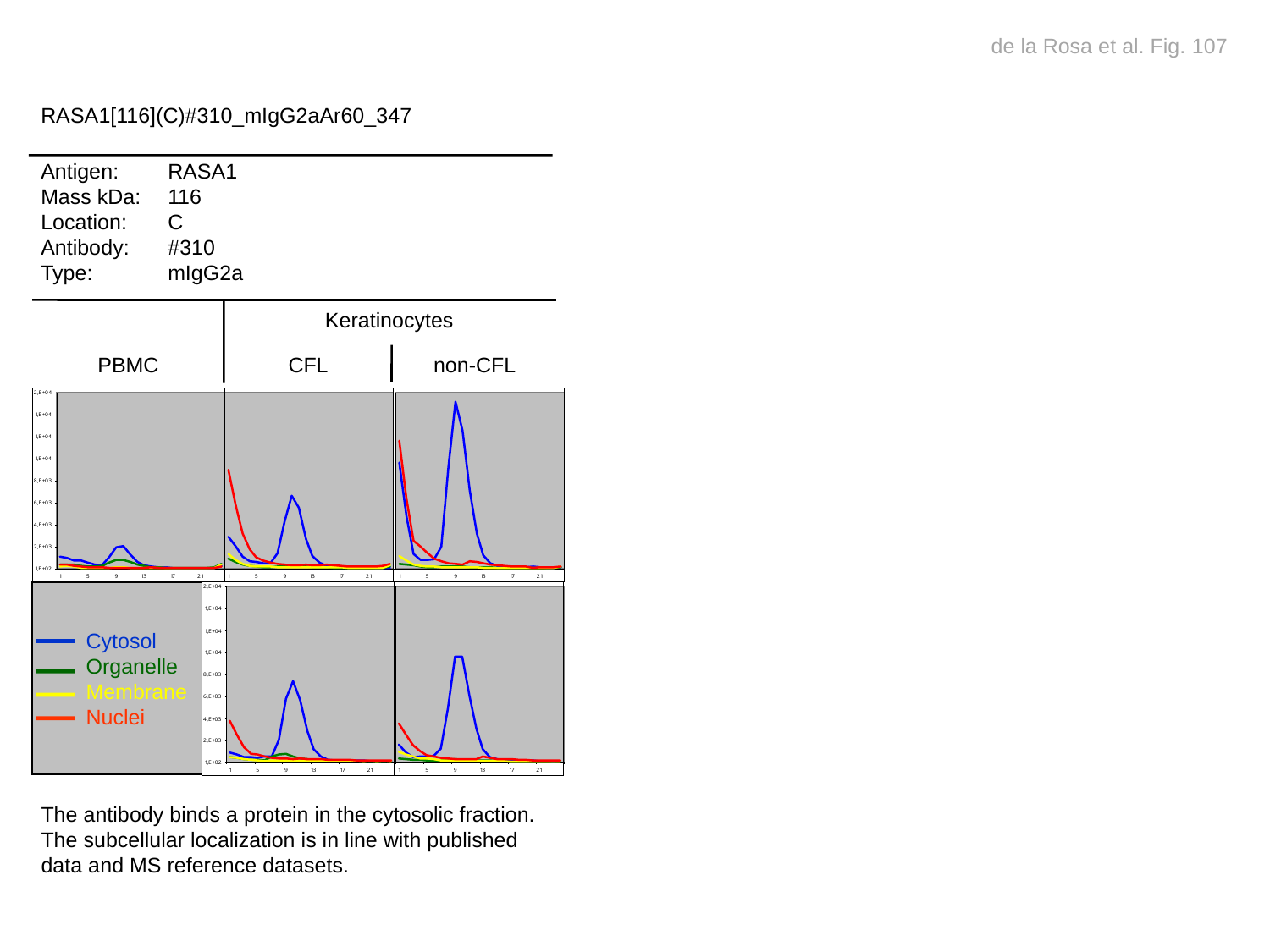

de la Rosa et al. Fig. <number>
# RASA1[116](C)#310_mIgG2aAr60_347
Antigen: 	RASA1
Mass kDa:	116
Location: 	C
Antibody: 	#310
Type:	mIgG2a
The antibody binds a protein in the cytosolic fraction. The subcellular localization is in line with published data and MS reference datasets.

## Slide 108
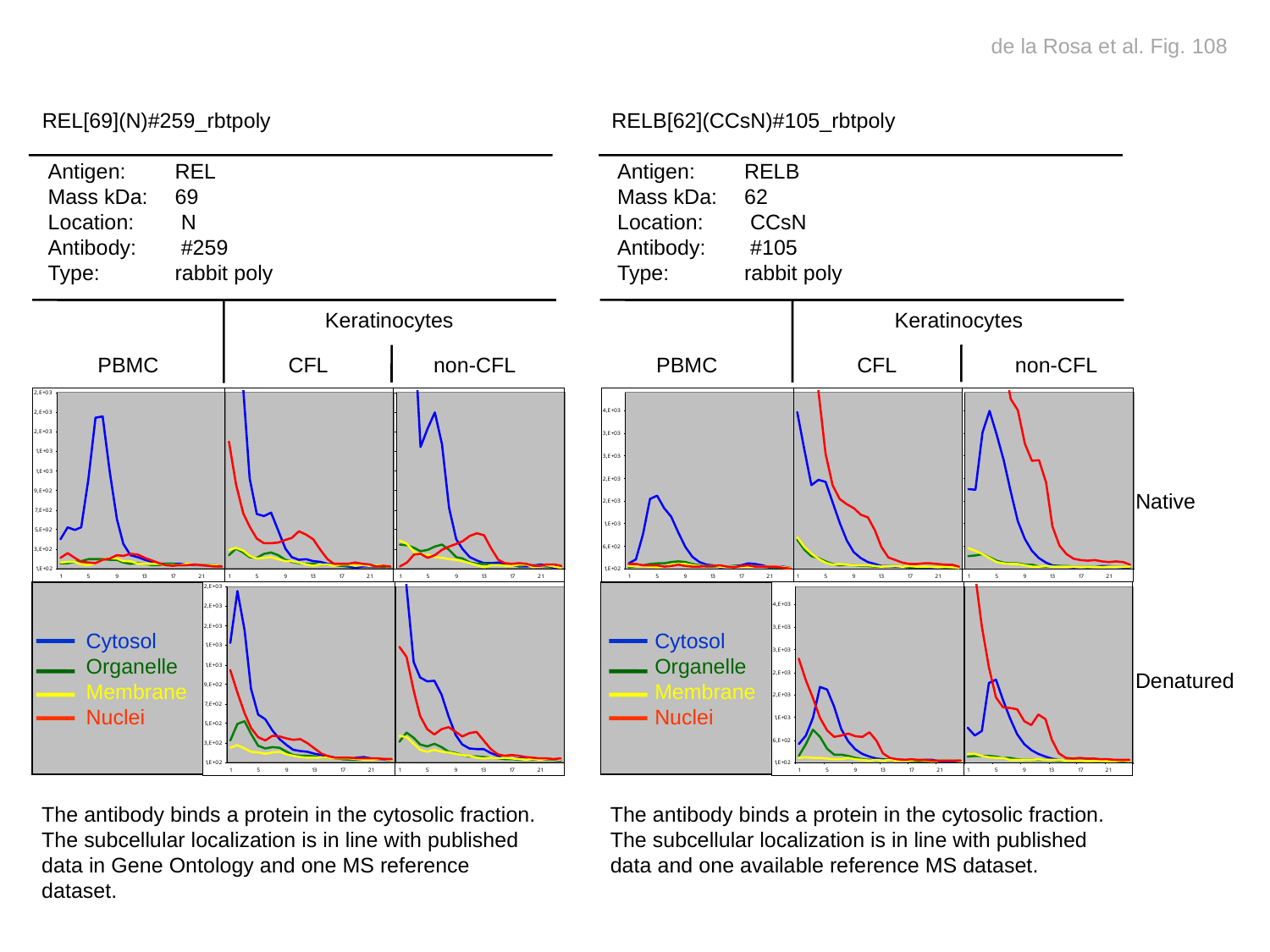

de la Rosa et al. Fig. <number>
REL[69](N)#259_rbtpoly
RELB[62](CCsN)#105_rbtpoly
Antigen: 	REL
Mass kDa:	69
Location: 	 N
Antibody: 	 #259
Type:	rabbit poly
Antigen: 	RELB
Mass kDa:	62
Location: 	 CCsN
Antibody: 	 #105
Type:	rabbit poly
The antibody binds a protein in the cytosolic fraction. The subcellular localization is in line with published data in Gene Ontology and one MS reference dataset.
The antibody binds a protein in the cytosolic fraction. The subcellular localization is in line with published data and one available reference MS dataset.

## Slide 109
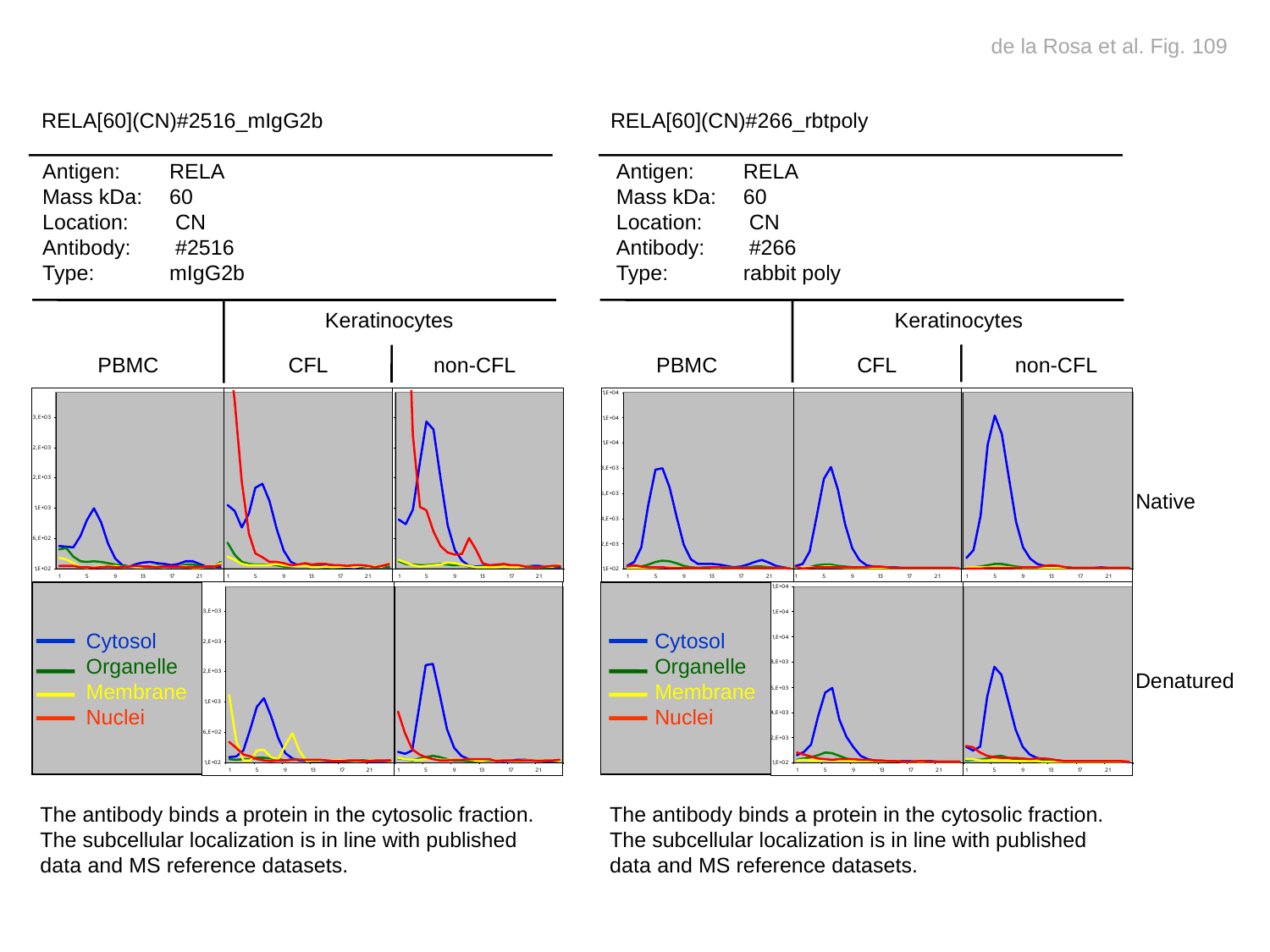

de la Rosa et al. Fig. <number>
RELA[60](CN)#2516_mIgG2b
RELA[60](CN)#266_rbtpoly
Antigen: 	RELA
Mass kDa:	60
Location: 	 CN
Antibody: 	 #2516
Type:	mIgG2b
Antigen: 	RELA
Mass kDa:	60
Location: 	 CN
Antibody: 	 #266
Type:	rabbit poly
The antibody binds a protein in the cytosolic fraction. The subcellular localization is in line with published data and MS reference datasets.
The antibody binds a protein in the cytosolic fraction. The subcellular localization is in line with published data and MS reference datasets.

## Slide 110
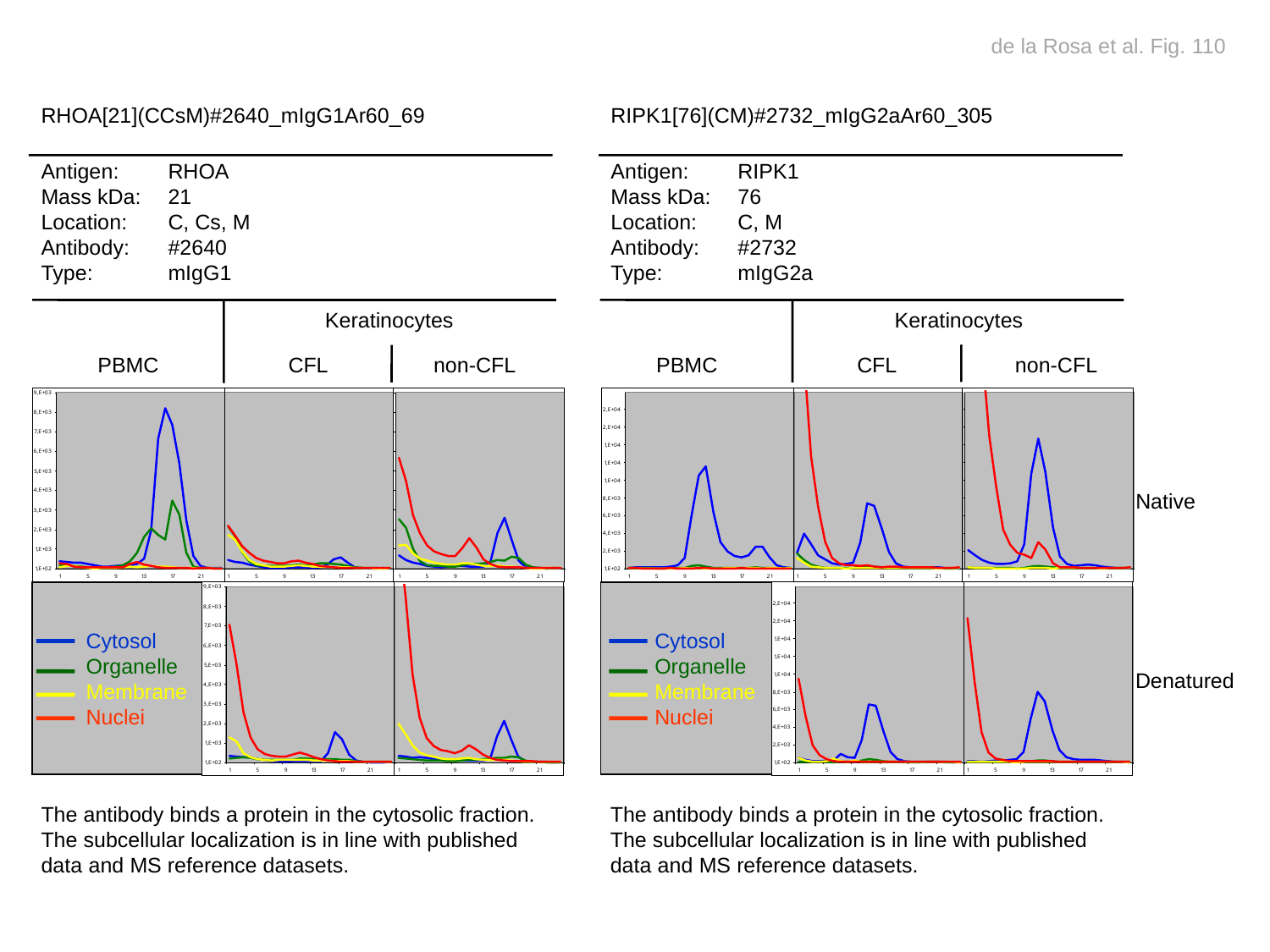

de la Rosa et al. Fig. <number>
# RHOA[21](CCsM)#2640_mIgG1Ar60_69
RIPK1[76](CM)#2732_mIgG2aAr60_305
Antigen: 	RHOA
Mass kDa:	21
Location: 	C, Cs, M
Antibody: 	#2640
Type:	mIgG1
Antigen: 	RIPK1
Mass kDa:	76
Location: 	C, M
Antibody: 	#2732
Type:	mIgG2a
The antibody binds a protein in the cytosolic fraction. The subcellular localization is in line with published data and MS reference datasets.
The antibody binds a protein in the cytosolic fraction. The subcellular localization is in line with published data and MS reference datasets.

## Slide 111
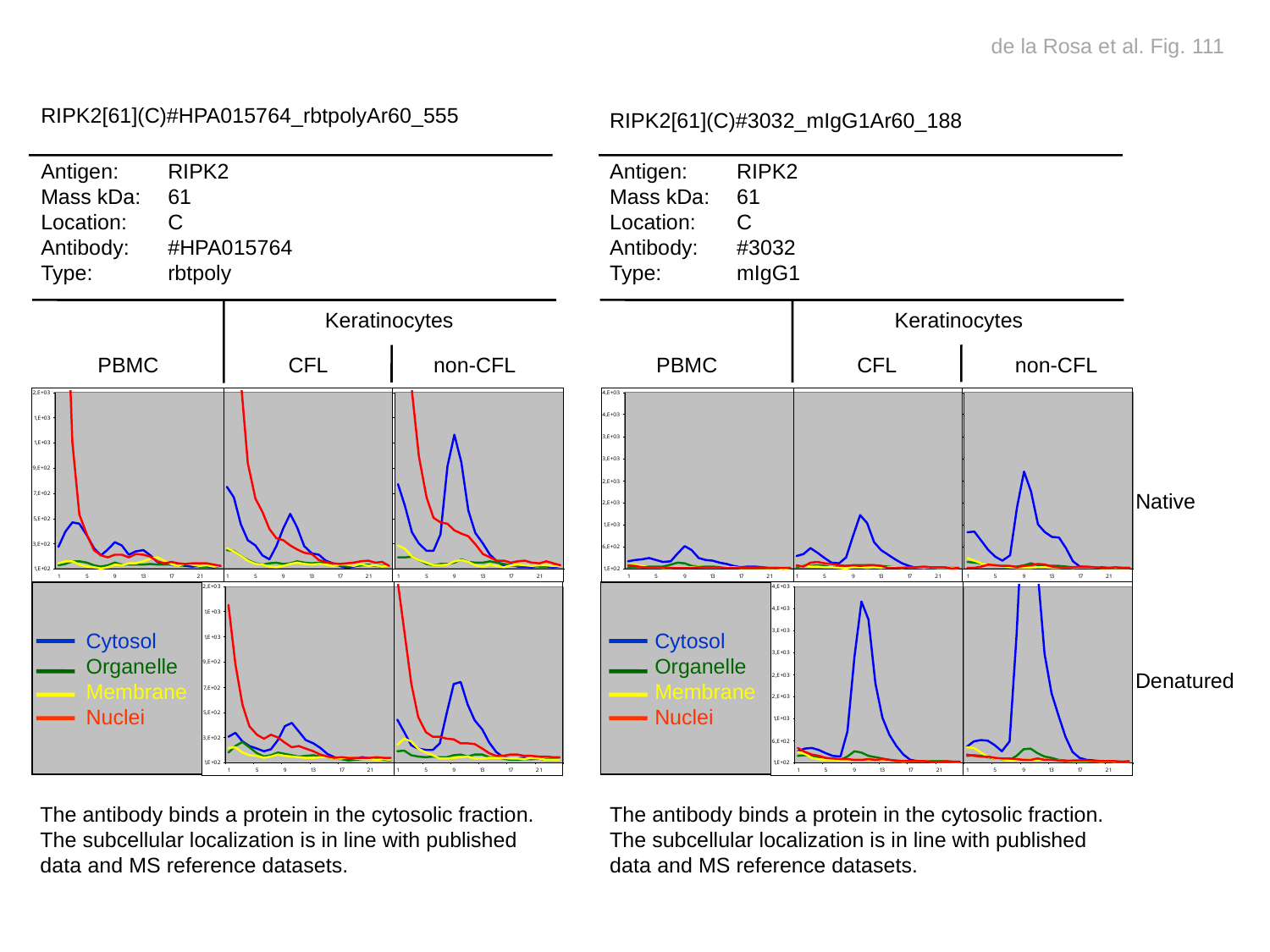

de la Rosa et al. Fig. <number>
RIPK2[61](C)#HPA015764_rbtpolyAr60_555
RIPK2[61](C)#3032_mIgG1Ar60_188
Antigen: 	RIPK2
Mass kDa:	61
Location: 	C
Antibody: 	#HPA015764
Type:	rbtpoly
# Antigen: 	RIPK2
Mass kDa:	61
Location: 	C
Antibody: 	#3032
Type:	mIgG1
The antibody binds a protein in the cytosolic fraction. The subcellular localization is in line with published data and MS reference datasets.
The antibody binds a protein in the cytosolic fraction. The subcellular localization is in line with published data and MS reference datasets.

## Slide 112
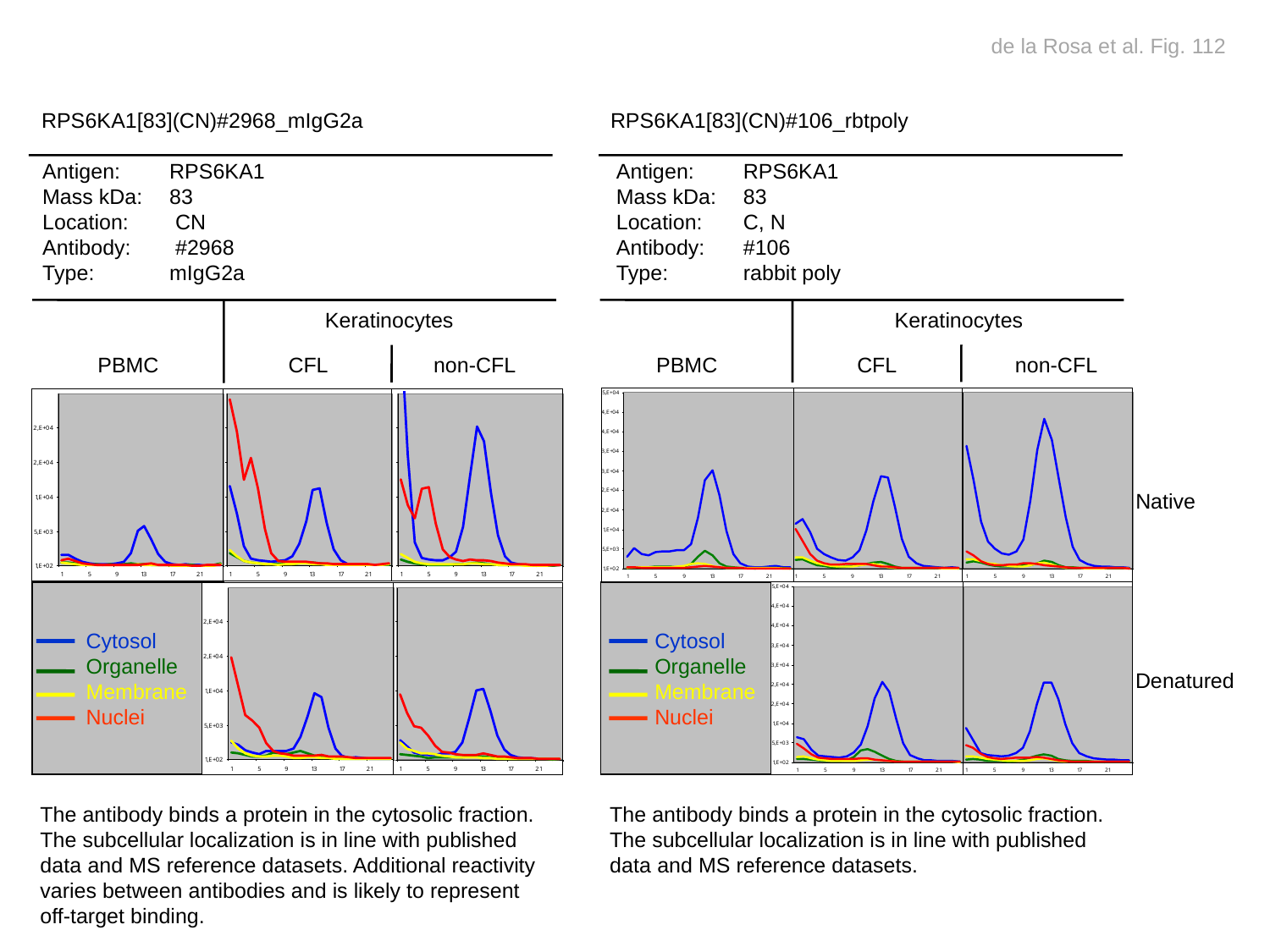

de la Rosa et al. Fig. <number>
RPS6KA1[83](CN)#2968_mIgG2a
RPS6KA1[83](CN)#106_rbtpoly
Antigen: 	RPS6KA1
Mass kDa:	83
Location: 	 CN
Antibody: 	 #2968
Type:	mIgG2a
Antigen: 	RPS6KA1
Mass kDa:	83
Location: 	C, N
Antibody: 	#106
Type:	rabbit poly
The antibody binds a protein in the cytosolic fraction. The subcellular localization is in line with published data and MS reference datasets. Additional reactivity varies between antibodies and is likely to represent off-target binding.
The antibody binds a protein in the cytosolic fraction. The subcellular localization is in line with published data and MS reference datasets.

## Slide 113
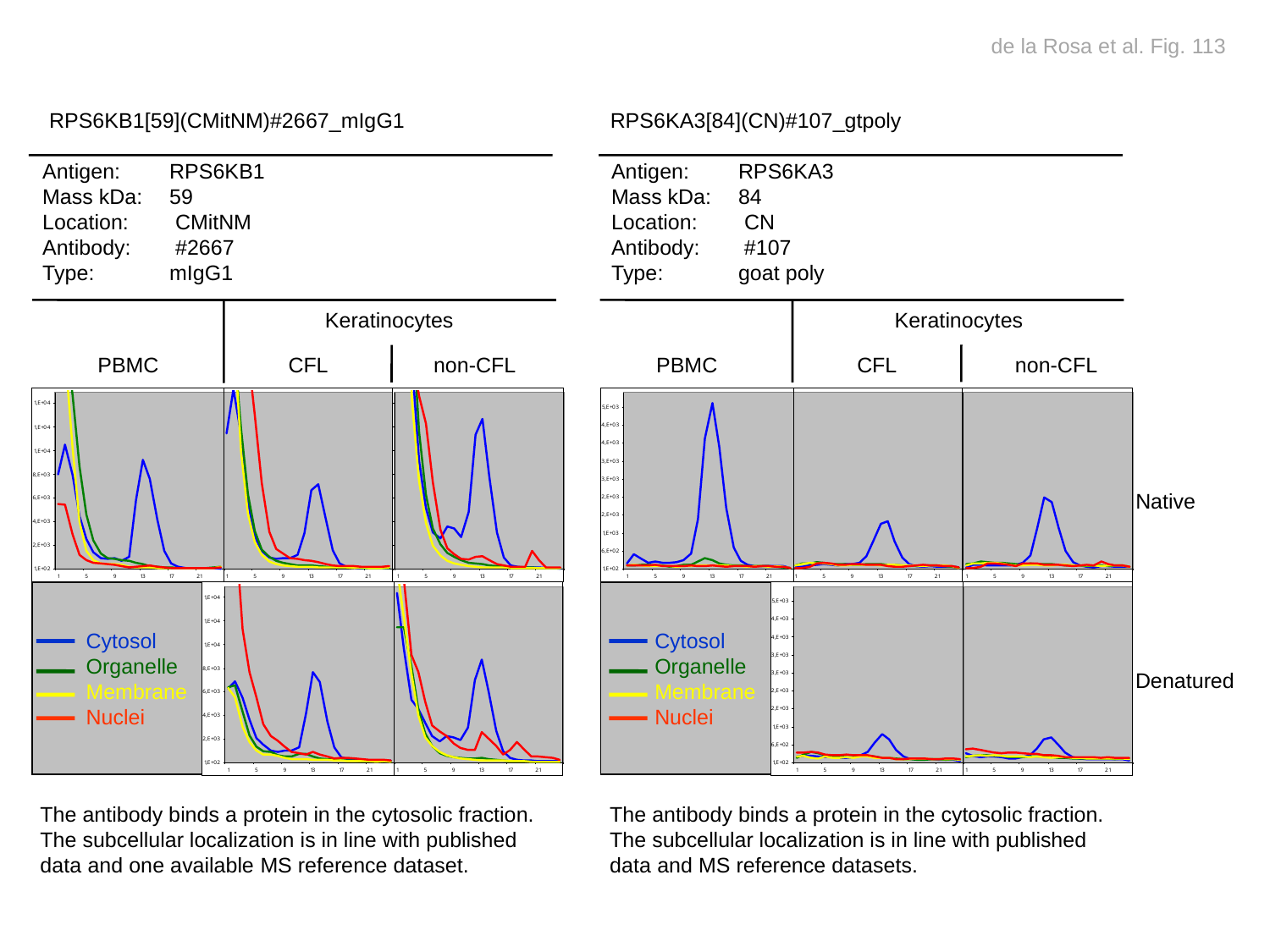

de la Rosa et al. Fig. <number>
RPS6KB1[59](CMitNM)#2667_mIgG1
RPS6KA3[84](CN)#107_gtpoly
Antigen: 	RPS6KB1
Mass kDa:	59
Location: 	 CMitNM
Antibody: 	 #2667
Type:	mIgG1
Antigen: 	RPS6KA3
Mass kDa:	84
Location: 	 CN
Antibody: 	 #107
Type:	goat poly
The antibody binds a protein in the cytosolic fraction. The subcellular localization is in line with published data and one available MS reference dataset.
The antibody binds a protein in the cytosolic fraction. The subcellular localization is in line with published data and MS reference datasets.

## Slide 114
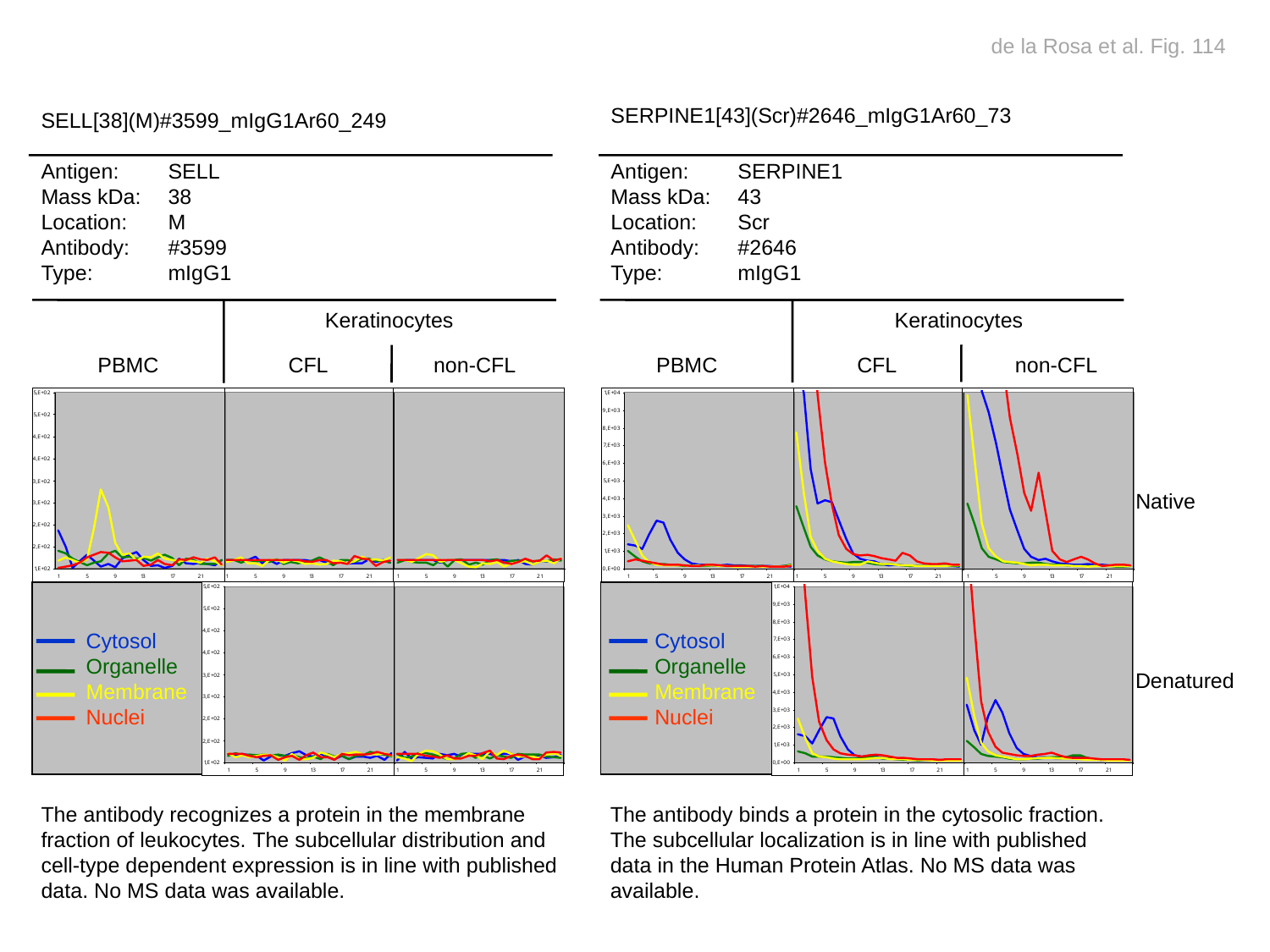

de la Rosa et al. Fig. <number>
# SELL[38](M)#3599_mIgG1Ar60_249
SERPINE1[43](Scr)#2646_mIgG1Ar60_73
Antigen: 	SELL
Mass kDa:	38
Location: 	M
Antibody: 	#3599
Type:	mIgG1
Antigen: 	SERPINE1
Mass kDa:	43
Location: 	Scr
Antibody: 	#2646
Type:	mIgG1
The antibody recognizes a protein in the membrane fraction of leukocytes. The subcellular distribution and cell-type dependent expression is in line with published data. No MS data was available.
The antibody binds a protein in the cytosolic fraction. The subcellular localization is in line with published data in the Human Protein Atlas. No MS data was available.

## Slide 115
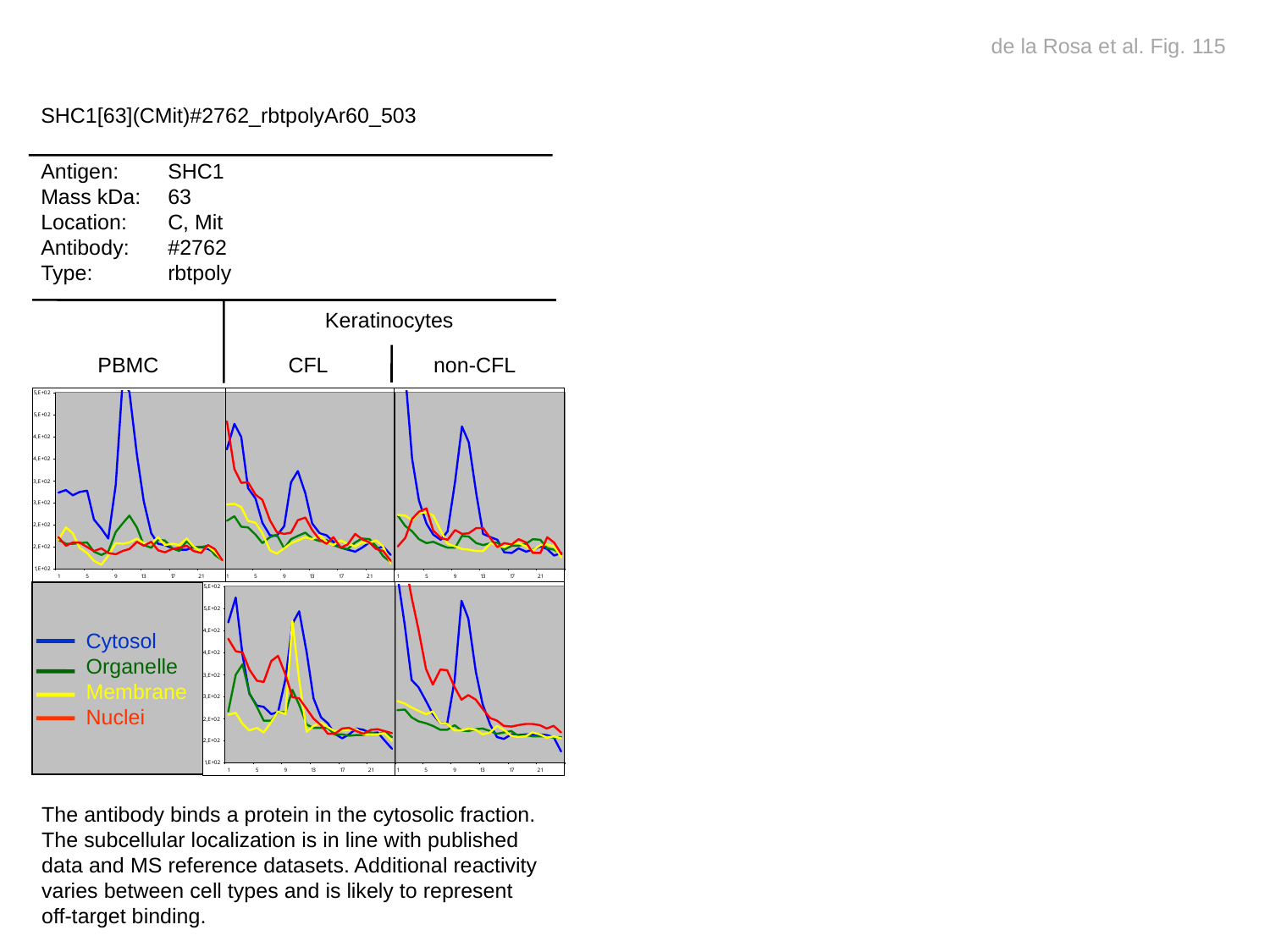

de la Rosa et al. Fig. <number>
# SHC1[63](CMit)#2762_rbtpolyAr60_503
Antigen: 	SHC1
Mass kDa:	63
Location: 	C, Mit
Antibody: 	#2762
Type:	rbtpoly
The antibody binds a protein in the cytosolic fraction. The subcellular localization is in line with published data and MS reference datasets. Additional reactivity varies between cell types and is likely to represent off-target binding.

## Slide 116
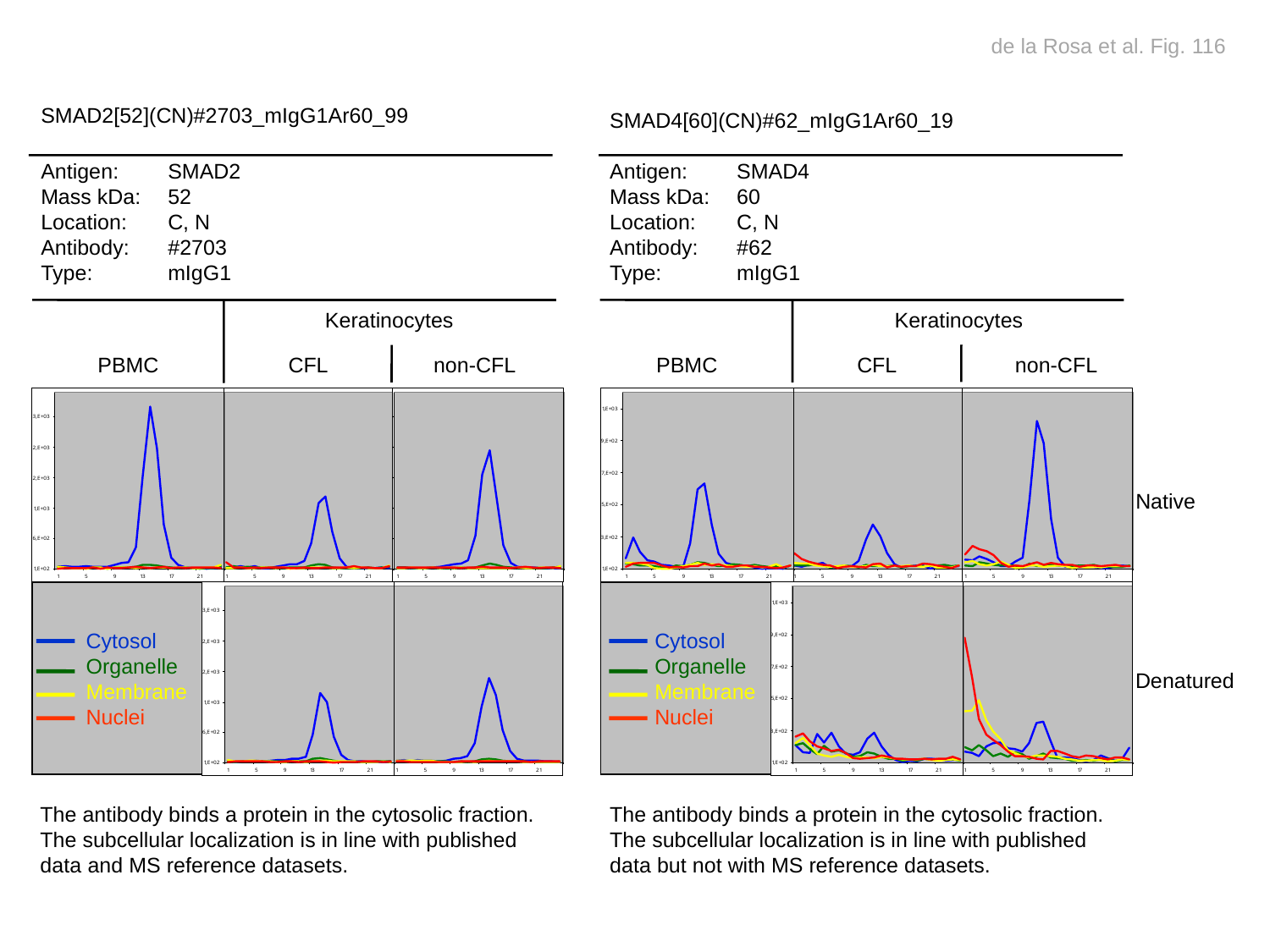

de la Rosa et al. Fig. <number>
# SMAD2[52](CN)#2703_mIgG1Ar60_99
SMAD4[60](CN)#62_mIgG1Ar60_19
Antigen: 	SMAD2
Mass kDa:	52
Location: 	C, N
Antibody: 	#2703
Type:	mIgG1
Antigen: 	SMAD4
Mass kDa:	60
Location: 	C, N
Antibody: 	#62
Type:	mIgG1
The antibody binds a protein in the cytosolic fraction. The subcellular localization is in line with published data and MS reference datasets.
The antibody binds a protein in the cytosolic fraction. The subcellular localization is in line with published data but not with MS reference datasets.

## Slide 117
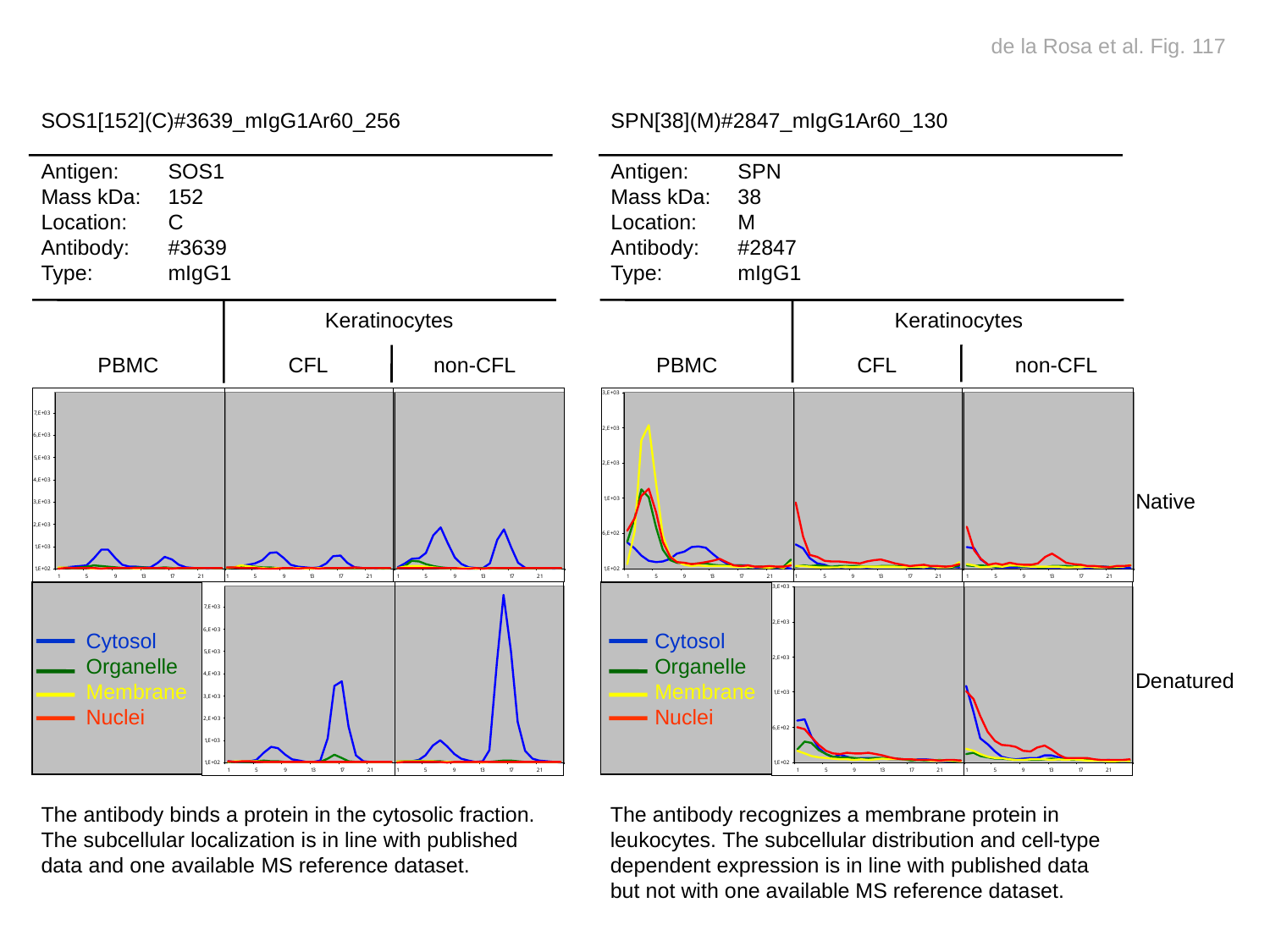

de la Rosa et al. Fig. <number>
# SOS1[152](C)#3639_mIgG1Ar60_256
SPN[38](M)#2847_mIgG1Ar60_130
Antigen: 	SOS1
Mass kDa:	152
Location: 	C
Antibody: 	#3639
Type:	mIgG1
Antigen: 	SPN
Mass kDa:	38
Location: 	M
Antibody: 	#2847
Type:	mIgG1
The antibody binds a protein in the cytosolic fraction. The subcellular localization is in line with published data and one available MS reference dataset.
The antibody recognizes a membrane protein in leukocytes. The subcellular distribution and cell-type dependent expression is in line with published data but not with one available MS reference dataset.

## Slide 118
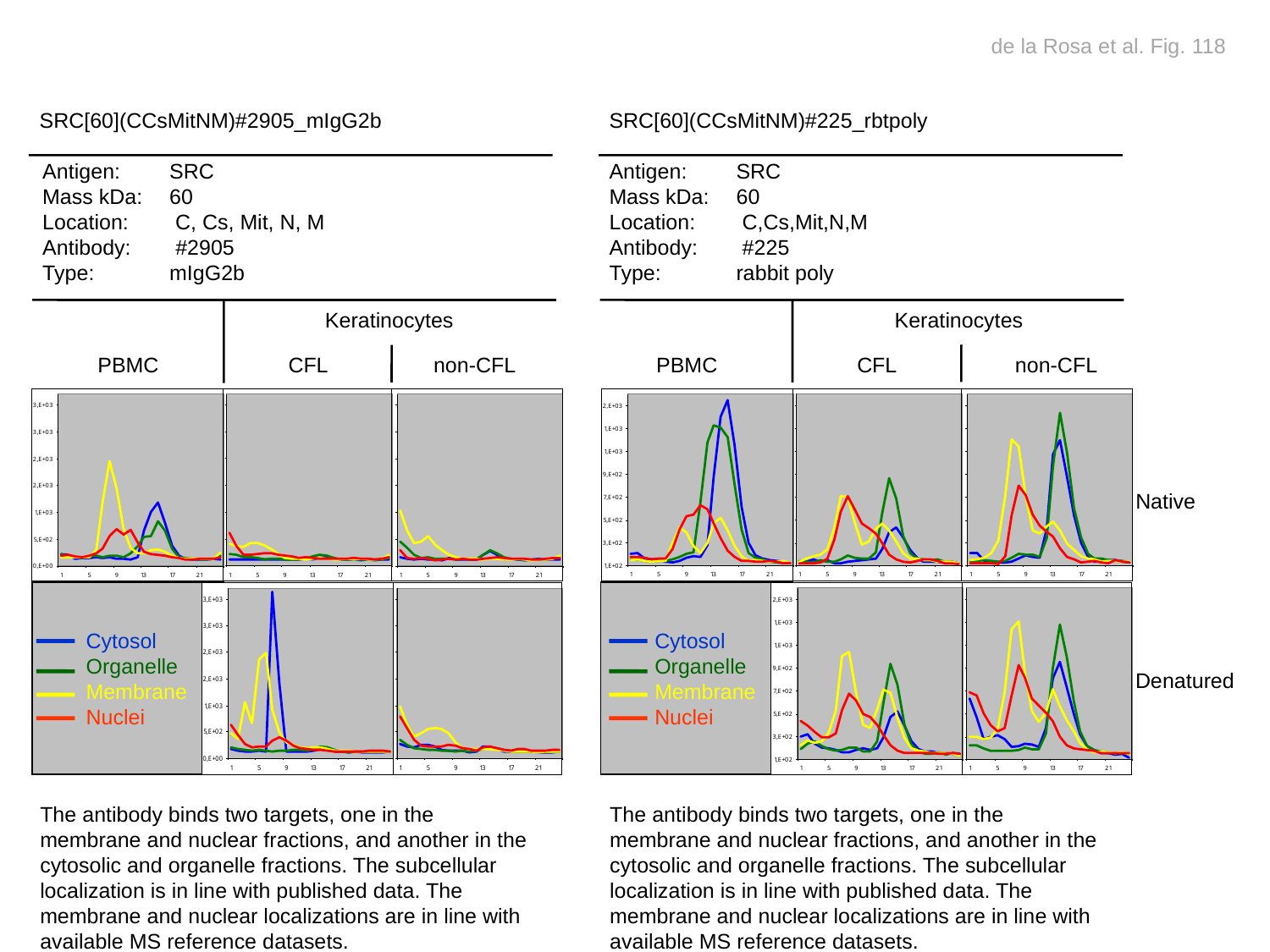

de la Rosa et al. Fig. <number>
SRC[60](CCsMitNM)#2905_mIgG2b
SRC[60](CCsMitNM)#225_rbtpoly
Antigen: 	SRC
Mass kDa:	60
Location: 	 C, Cs, Mit, N, M
Antibody: 	 #2905
Type:	mIgG2b
Antigen: 	SRC
Mass kDa:	60
Location: 	 C,Cs,Mit,N,M
Antibody: 	 #225
Type:	rabbit poly
The antibody binds two targets, one in the membrane and nuclear fractions, and another in the cytosolic and organelle fractions. The subcellular localization is in line with published data. The membrane and nuclear localizations are in line with available MS reference datasets.
.
The antibody binds two targets, one in the membrane and nuclear fractions, and another in the cytosolic and organelle fractions. The subcellular localization is in line with published data. The membrane and nuclear localizations are in line with available MS reference datasets.

## Slide 119
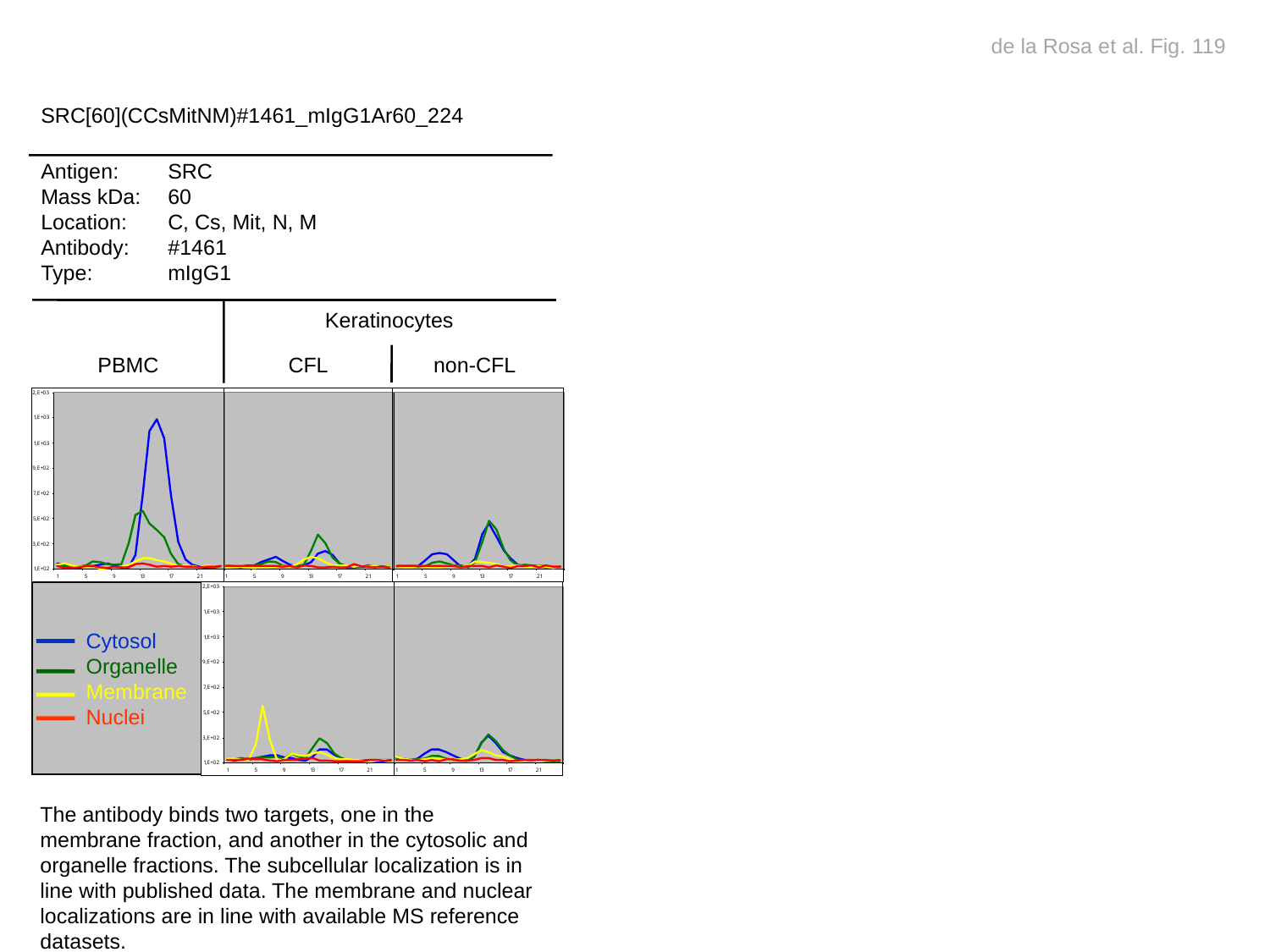

de la Rosa et al. Fig. <number>
# SRC[60](CCsMitNM)#1461_mIgG1Ar60_224
Antigen: 	SRC
Mass kDa:	60
Location: 	C, Cs, Mit, N, M
Antibody: 	#1461
Type:	mIgG1
The antibody binds two targets, one in the membrane fraction, and another in the cytosolic and organelle fractions. The subcellular localization is in line with published data. The membrane and nuclear localizations are in line with available MS reference datasets.

## Slide 120
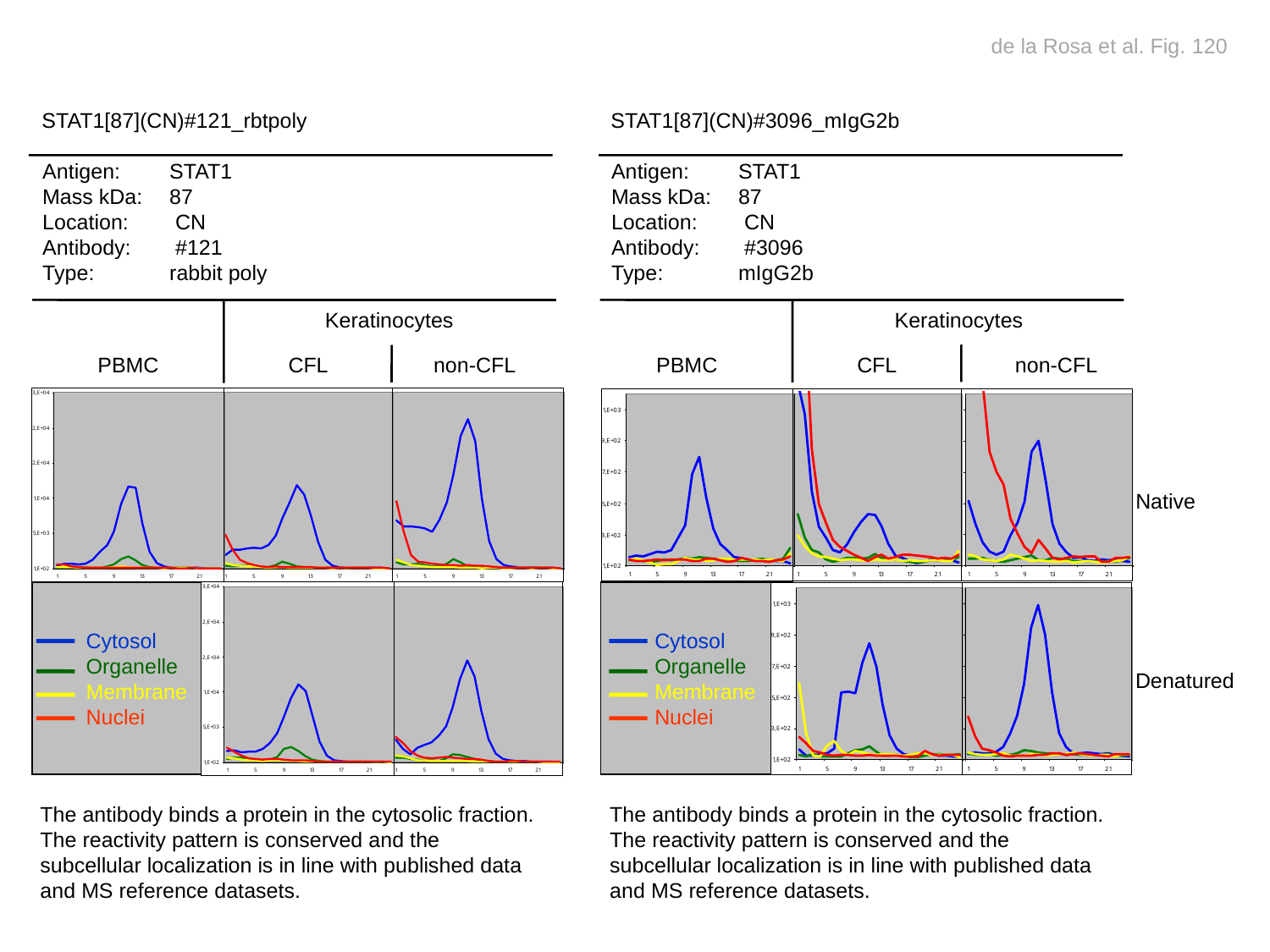

de la Rosa et al. Fig. <number>
STAT1[87](CN)#121_rbtpoly
STAT1[87](CN)#3096_mIgG2b
Antigen: 	STAT1
Mass kDa:	87
Location: 	 CN
Antibody: 	 #121
Type:	rabbit poly
Antigen: 	STAT1
Mass kDa:	87
Location: 	 CN
Antibody: 	 #3096
Type:	mIgG2b
The antibody binds a protein in the cytosolic fraction. The reactivity pattern is conserved and the subcellular localization is in line with published data and MS reference datasets.
The antibody binds a protein in the cytosolic fraction. The reactivity pattern is conserved and the subcellular localization is in line with published data and MS reference datasets.

## Slide 121
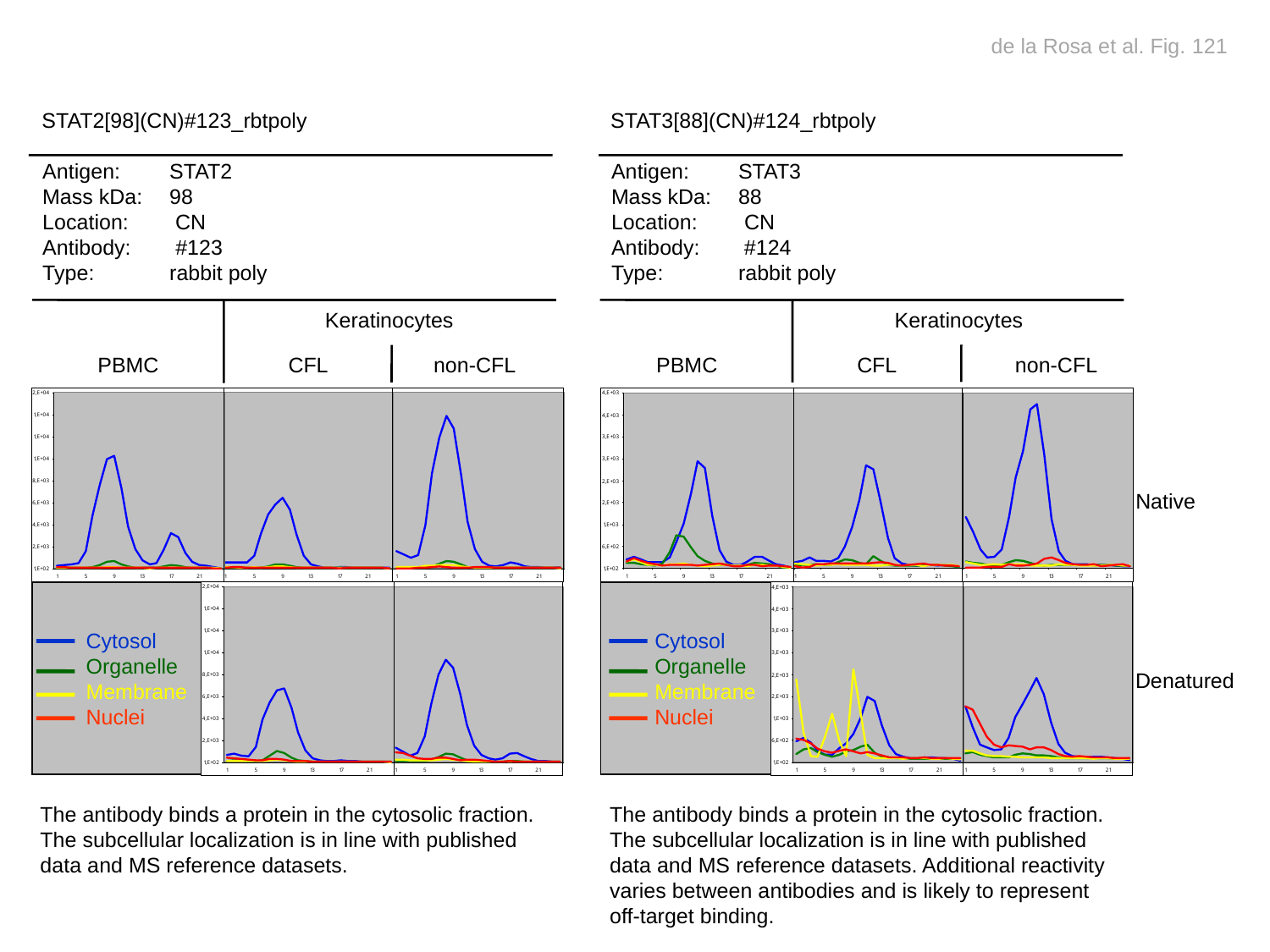

de la Rosa et al. Fig. <number>
STAT2[98](CN)#123_rbtpoly
STAT3[88](CN)#124_rbtpoly
Antigen: 	STAT2
Mass kDa:	98
Location: 	 CN
Antibody: 	 #123
Type:	rabbit poly
Antigen: 	STAT3
Mass kDa:	88
Location: 	 CN
Antibody: 	 #124
Type:	rabbit poly
The antibody binds a protein in the cytosolic fraction. The subcellular localization is in line with published data and MS reference datasets.
The antibody binds a protein in the cytosolic fraction. The subcellular localization is in line with published data and MS reference datasets. Additional reactivity varies between antibodies and is likely to represent off-target binding.

## Slide 122
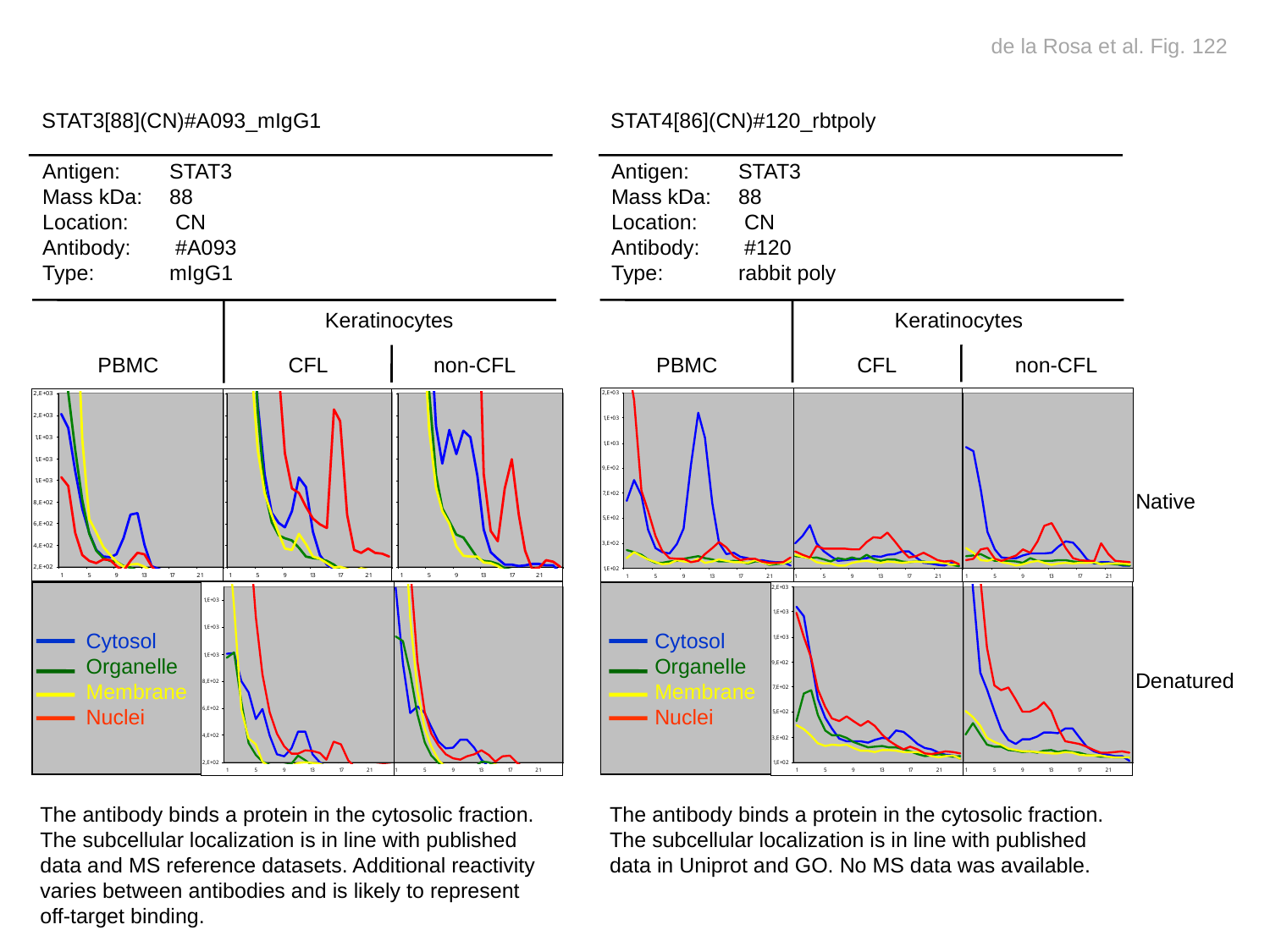

de la Rosa et al. Fig. <number>
STAT3[88](CN)#A093_mIgG1
STAT4[86](CN)#120_rbtpoly
Antigen: 	STAT3
Mass kDa:	88
Location: 	 CN
Antibody: 	 #A093
Type:	mIgG1
Antigen: 	STAT3
Mass kDa:	88
Location: 	 CN
Antibody: 	 #120
Type:	rabbit poly
The antibody binds a protein in the cytosolic fraction. The subcellular localization is in line with published data and MS reference datasets. Additional reactivity varies between antibodies and is likely to represent off-target binding.
The antibody binds a protein in the cytosolic fraction. The subcellular localization is in line with published data in Uniprot and GO. No MS data was available.

## Slide 123
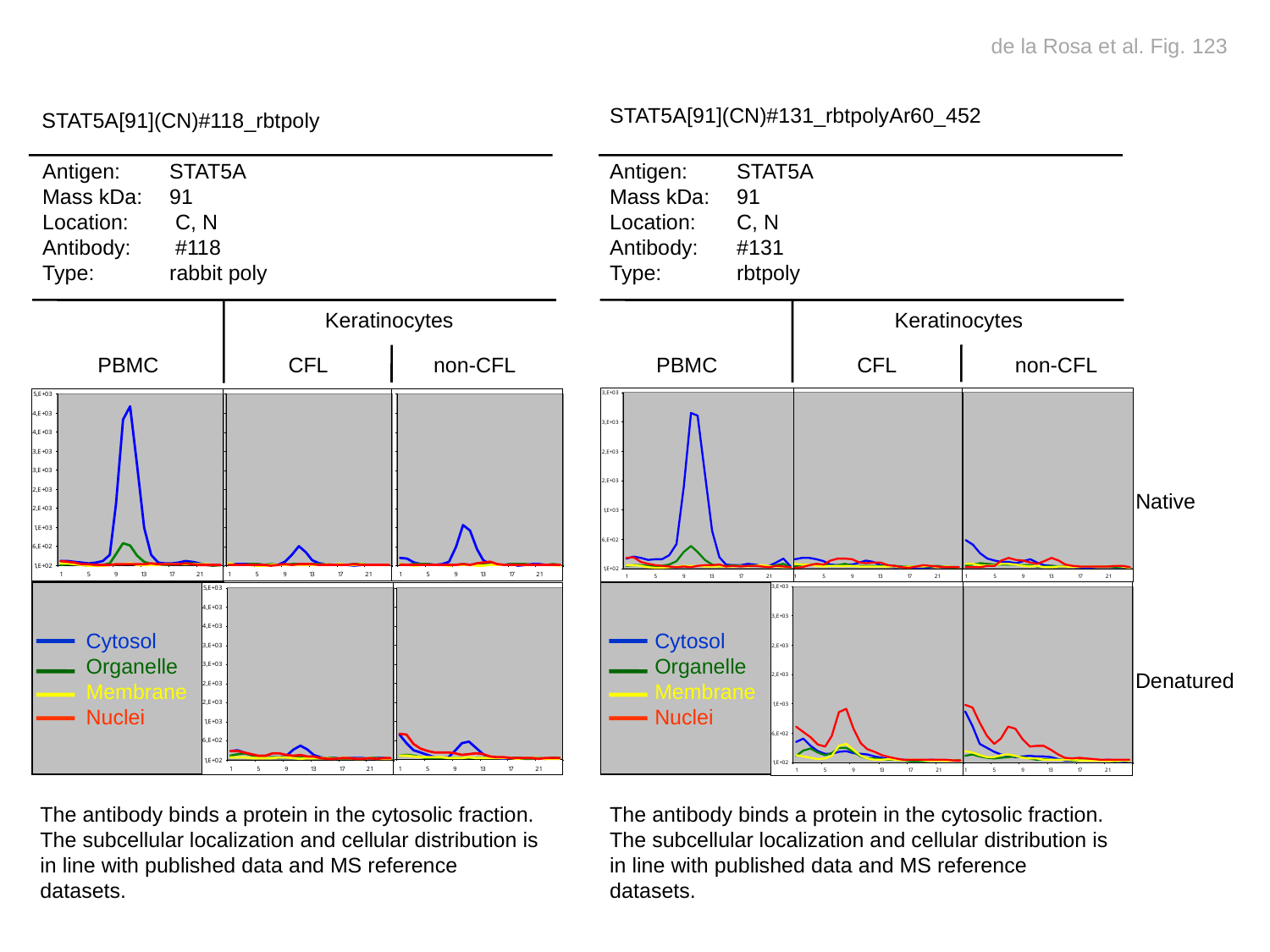

de la Rosa et al. Fig. <number>
# STAT5A[91](CN)#131_rbtpolyAr60_452
STAT5A[91](CN)#118_rbtpoly
Antigen: 	STAT5A
Mass kDa:	91
Location: 	C, N
Antibody: 	#131
Type:	rbtpoly
Antigen: 	STAT5A
Mass kDa:	91
Location: 	 C, N
Antibody: 	 #118
Type:	rabbit poly
The antibody binds a protein in the cytosolic fraction. The subcellular localization and cellular distribution is in line with published data and MS reference datasets.
The antibody binds a protein in the cytosolic fraction. The subcellular localization and cellular distribution is in line with published data and MS reference datasets.

## Slide 124
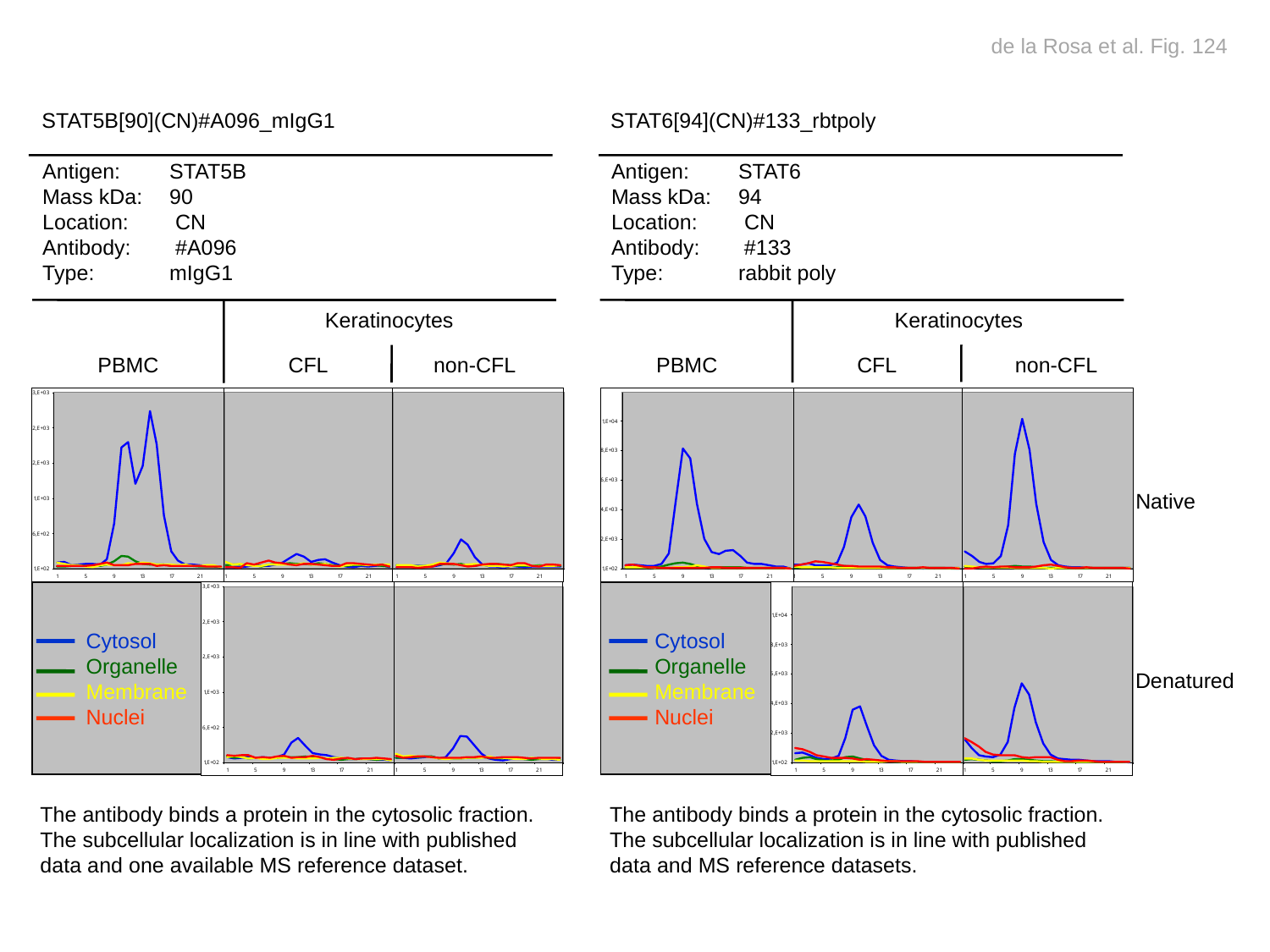

de la Rosa et al. Fig. <number>
STAT5B[90](CN)#A096_mIgG1
STAT6[94](CN)#133_rbtpoly
Antigen: 	STAT5B
Mass kDa:	90
Location: 	 CN
Antibody: 	 #A096
Type:	mIgG1
Antigen: 	STAT6
Mass kDa:	94
Location: 	 CN
Antibody: 	 #133
Type:	rabbit poly
The antibody binds a protein in the cytosolic fraction. The subcellular localization is in line with published data and one available MS reference dataset.
The antibody binds a protein in the cytosolic fraction. The subcellular localization is in line with published data and MS reference datasets.

## Slide 125
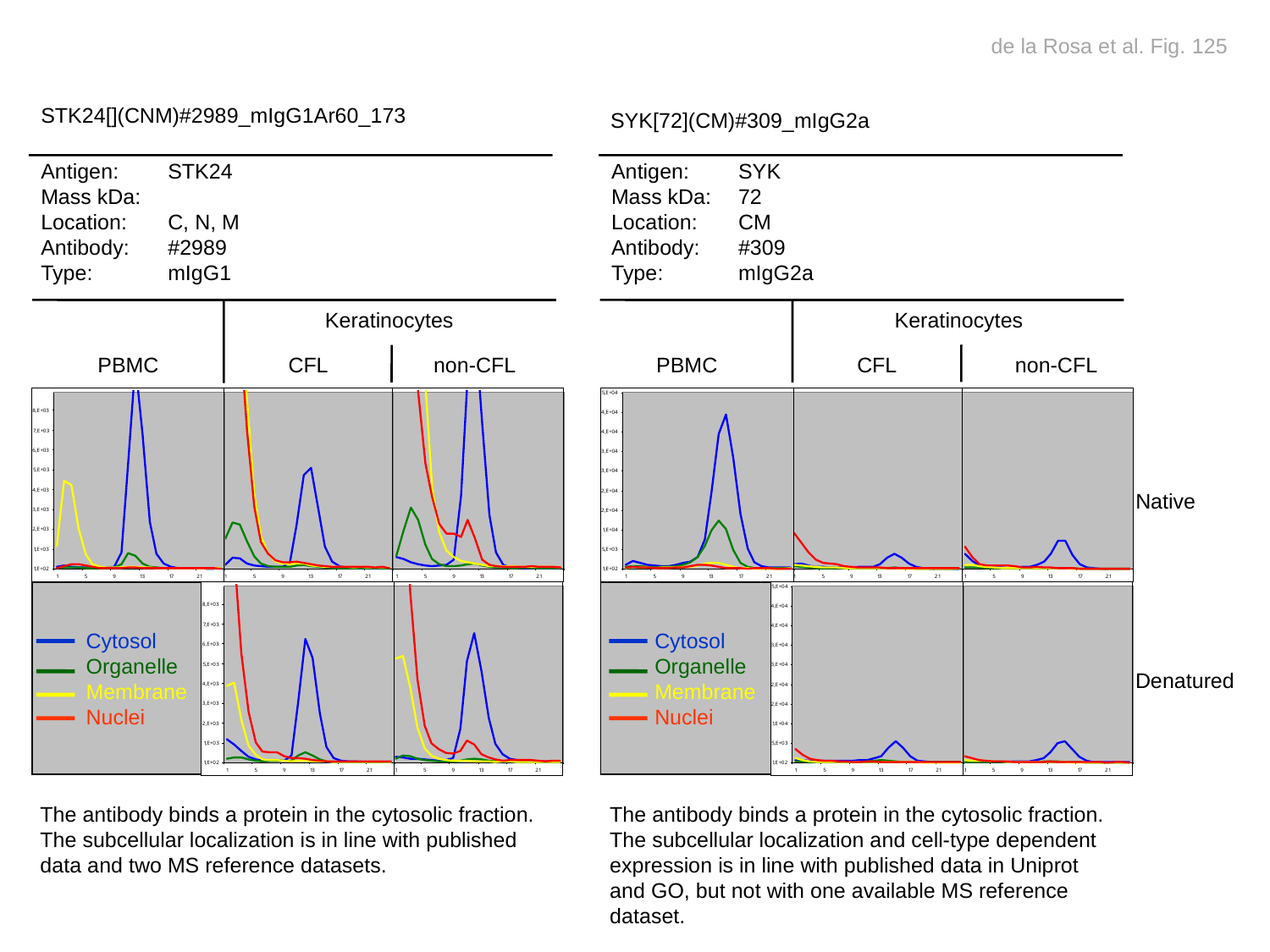

de la Rosa et al. Fig. <number>
# STK24[](CNM)#2989_mIgG1Ar60_173
SYK[72](CM)#309_mIgG2a
Antigen: 	STK24
Mass kDa:
Location: 	C, N, M
Antibody: 	#2989
Type:	mIgG1
Antigen: 	SYK
Mass kDa:	72
Location: 	CM
Antibody: 	#309
Type:	mIgG2a
The antibody binds a protein in the cytosolic fraction. The subcellular localization is in line with published data and two MS reference datasets.
The antibody binds a protein in the cytosolic fraction. The subcellular localization and cell-type dependent expression is in line with published data in Uniprot and GO, but not with one available MS reference dataset.

## Slide 126
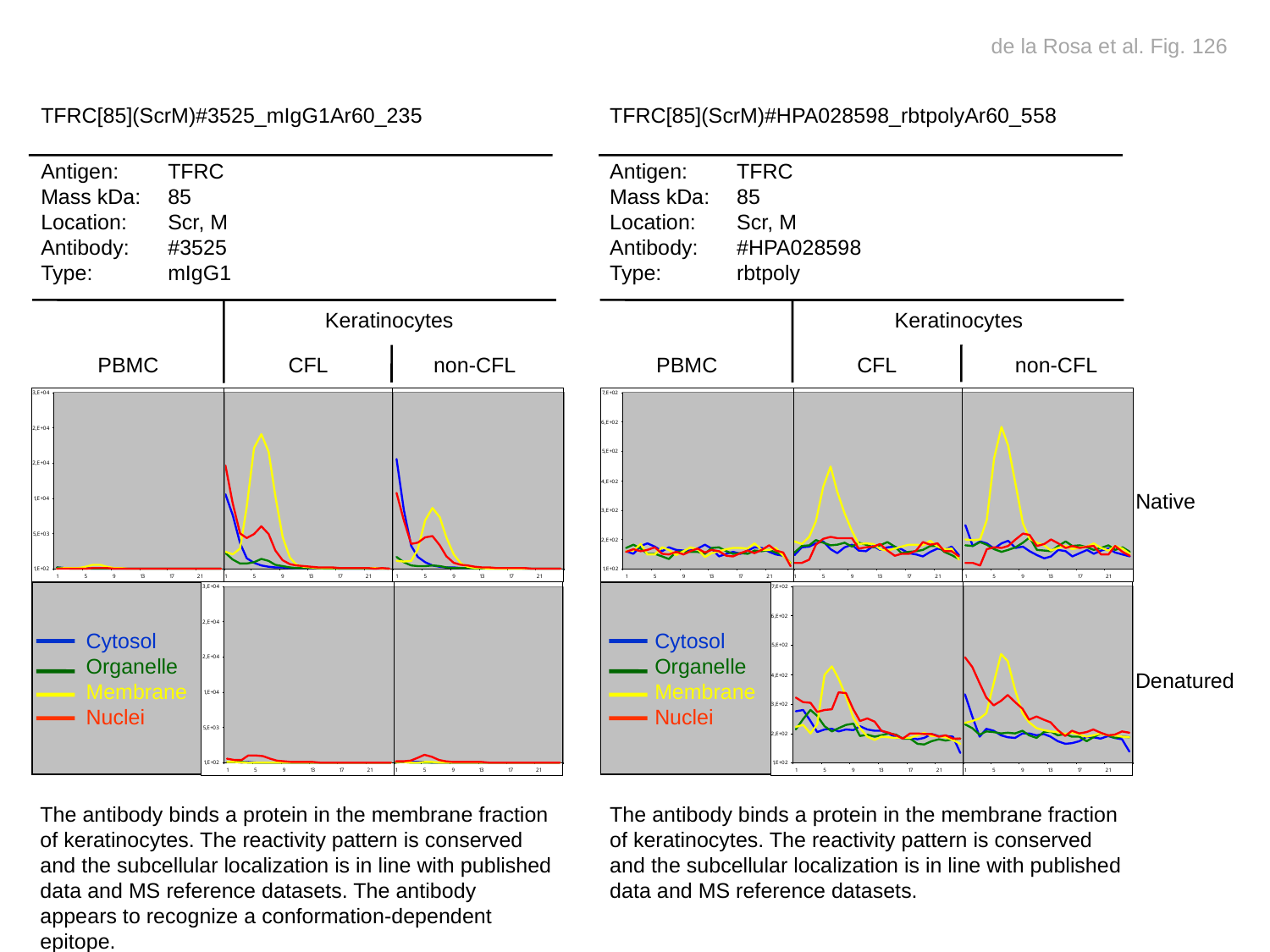

de la Rosa et al. Fig. <number>
# TFRC[85](ScrM)#3525_mIgG1Ar60_235
TFRC[85](ScrM)#HPA028598_rbtpolyAr60_558
Antigen: 	TFRC
Mass kDa:	85
Location: 	Scr, M
Antibody: 	#3525
Type:	mIgG1
Antigen: 	TFRC
Mass kDa:	85
Location: 	Scr, M
Antibody: 	#HPA028598
Type:	rbtpoly
The antibody binds a protein in the membrane fraction of keratinocytes. The reactivity pattern is conserved and the subcellular localization is in line with published data and MS reference datasets. The antibody appears to recognize a conformation-dependent epitope.
The antibody binds a protein in the membrane fraction of keratinocytes. The reactivity pattern is conserved and the subcellular localization is in line with published data and MS reference datasets.

## Slide 127
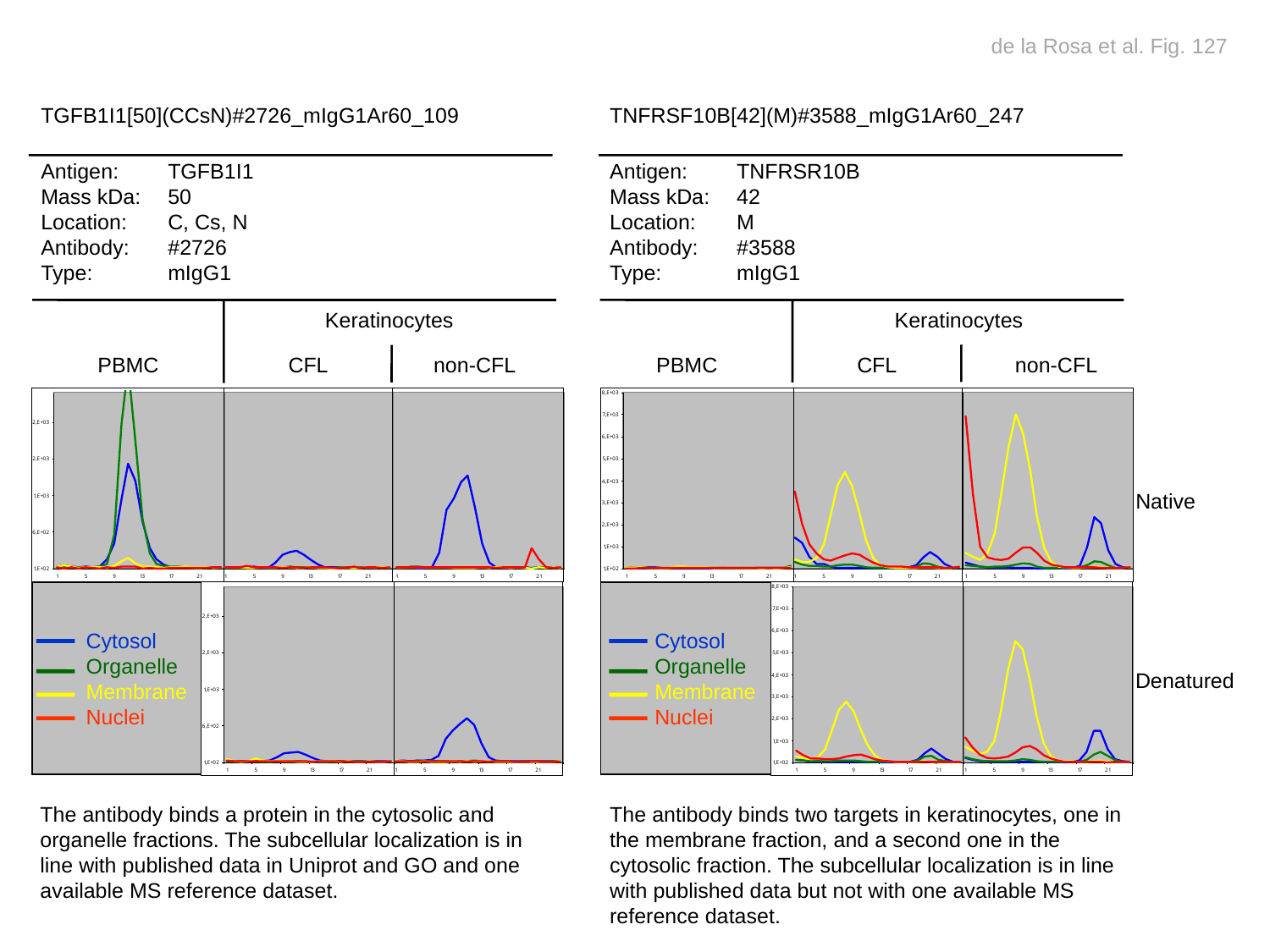

de la Rosa et al. Fig. <number>
# TGFB1I1[50](CCsN)#2726_mIgG1Ar60_109
TNFRSF10B[42](M)#3588_mIgG1Ar60_247
Antigen: 	TGFB1I1
Mass kDa:	50
Location: 	C, Cs, N
Antibody: 	#2726
Type:	mIgG1
Antigen: 	TNFRSR10B
Mass kDa:	42
Location: 	M
Antibody: 	#3588
Type:	mIgG1
The antibody binds a protein in the cytosolic and organelle fractions. The subcellular localization is in line with published data in Uniprot and GO and one available MS reference dataset.
The antibody binds two targets in keratinocytes, one in the membrane fraction, and a second one in the cytosolic fraction. The subcellular localization is in line with published data but not with one available MS reference dataset.

## Slide 128
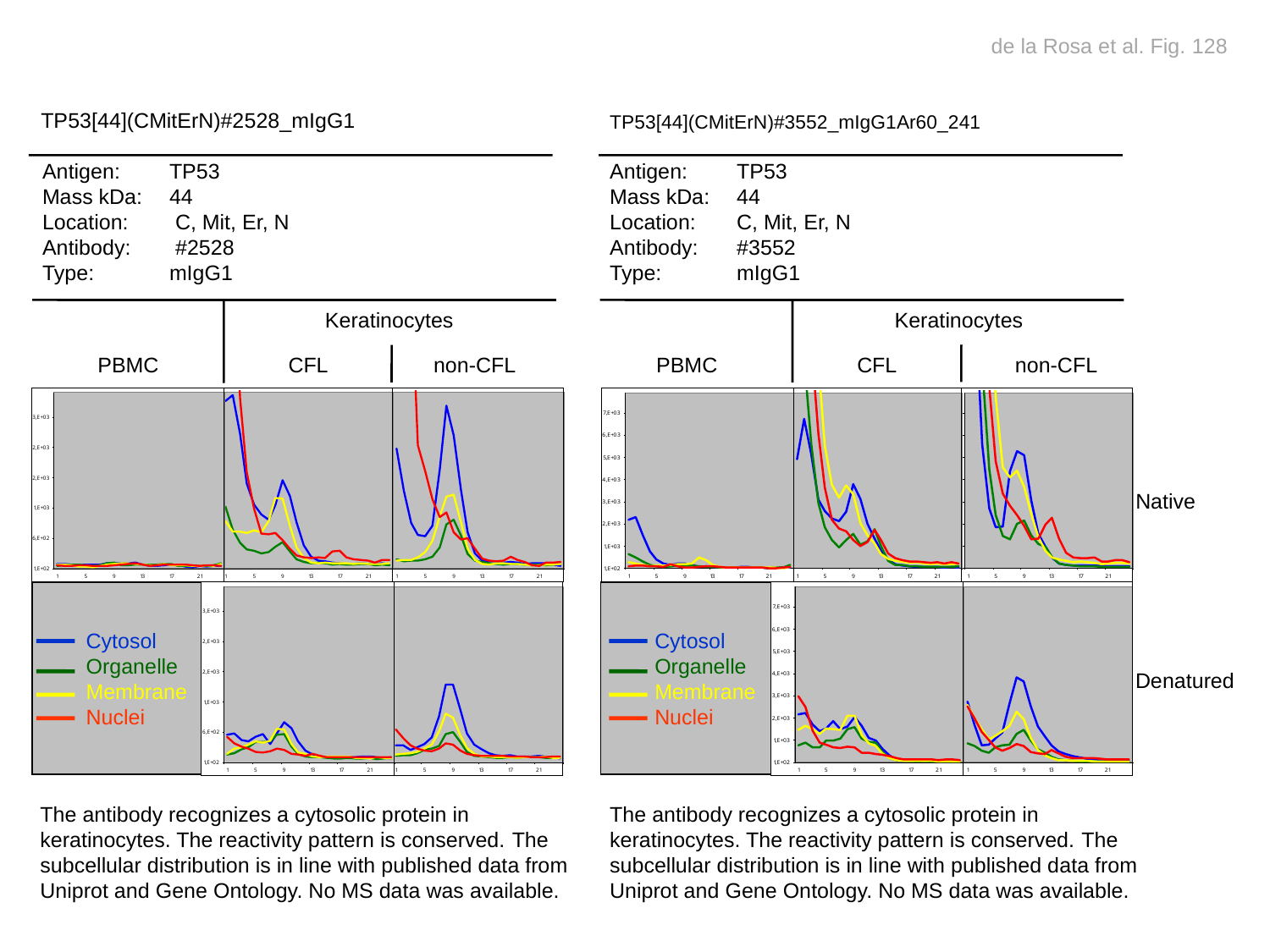

de la Rosa et al. Fig. <number>
TP53[44](CMitErN)#2528_mIgG1
# TP53[44](CMitErN)#3552_mIgG1Ar60_241
Antigen: 	TP53
Mass kDa:	44
Location: 	C, Mit, Er, N
Antibody: 	#3552
Type:	mIgG1
Antigen: 	TP53
Mass kDa:	44
Location: 	 C, Mit, Er, N
Antibody: 	 #2528
Type:	mIgG1
The antibody recognizes a cytosolic protein in keratinocytes. The reactivity pattern is conserved. The subcellular distribution is in line with published data from Uniprot and Gene Ontology. No MS data was available.
The antibody recognizes a cytosolic protein in keratinocytes. The reactivity pattern is conserved. The subcellular distribution is in line with published data from Uniprot and Gene Ontology. No MS data was available.

## Slide 129
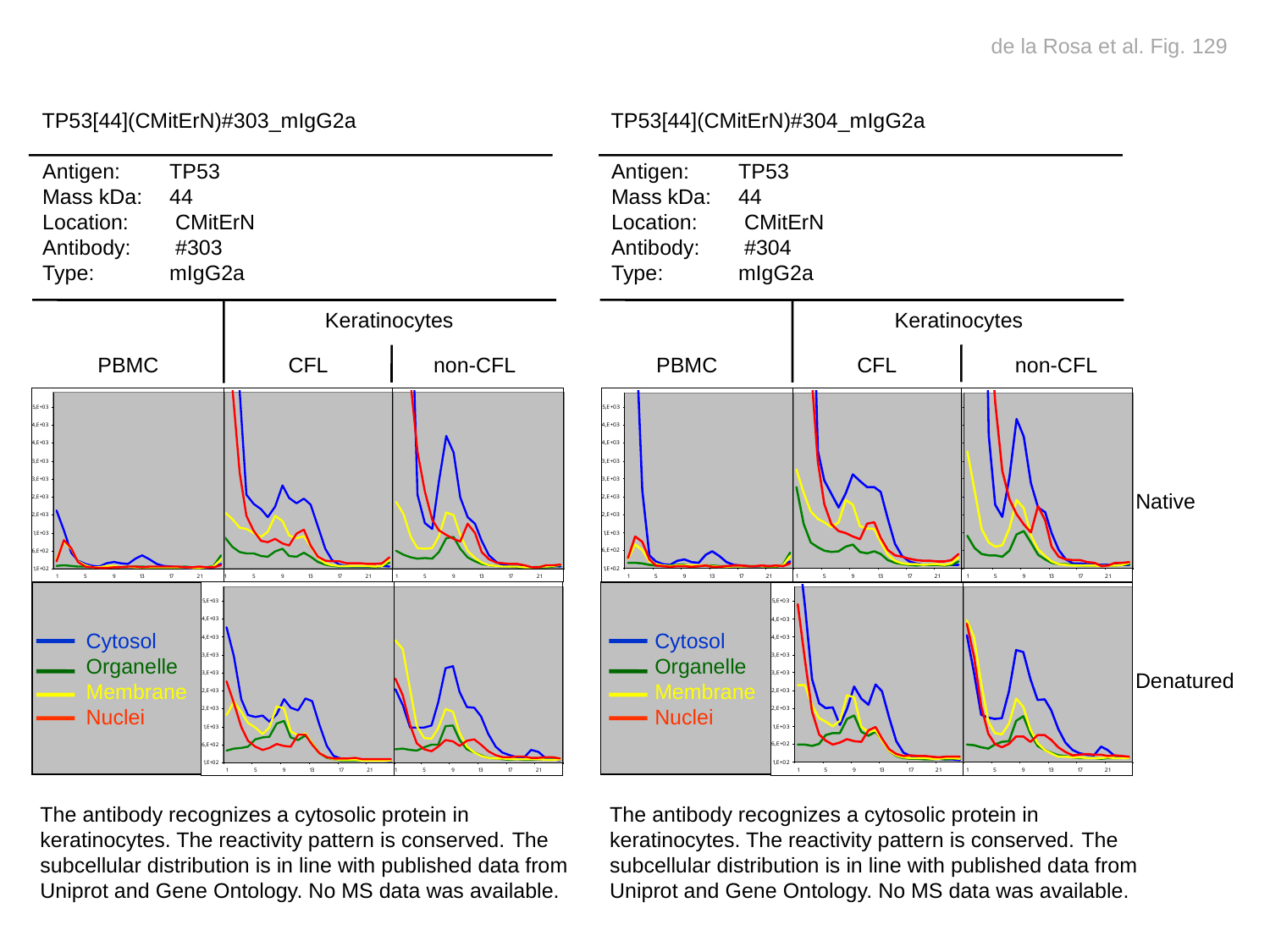

de la Rosa et al. Fig. <number>
TP53[44](CMitErN)#303_mIgG2a
TP53[44](CMitErN)#304_mIgG2a
Antigen: 	TP53
Mass kDa:	44
Location: 	 CMitErN
Antibody: 	 #303
Type:	mIgG2a
Antigen: 	TP53
Mass kDa:	44
Location: 	 CMitErN
Antibody: 	 #304
Type:	mIgG2a
The antibody recognizes a cytosolic protein in keratinocytes. The reactivity pattern is conserved. The subcellular distribution is in line with published data from Uniprot and Gene Ontology. No MS data was available.
The antibody recognizes a cytosolic protein in keratinocytes. The reactivity pattern is conserved. The subcellular distribution is in line with published data from Uniprot and Gene Ontology. No MS data was available.

## Slide 130
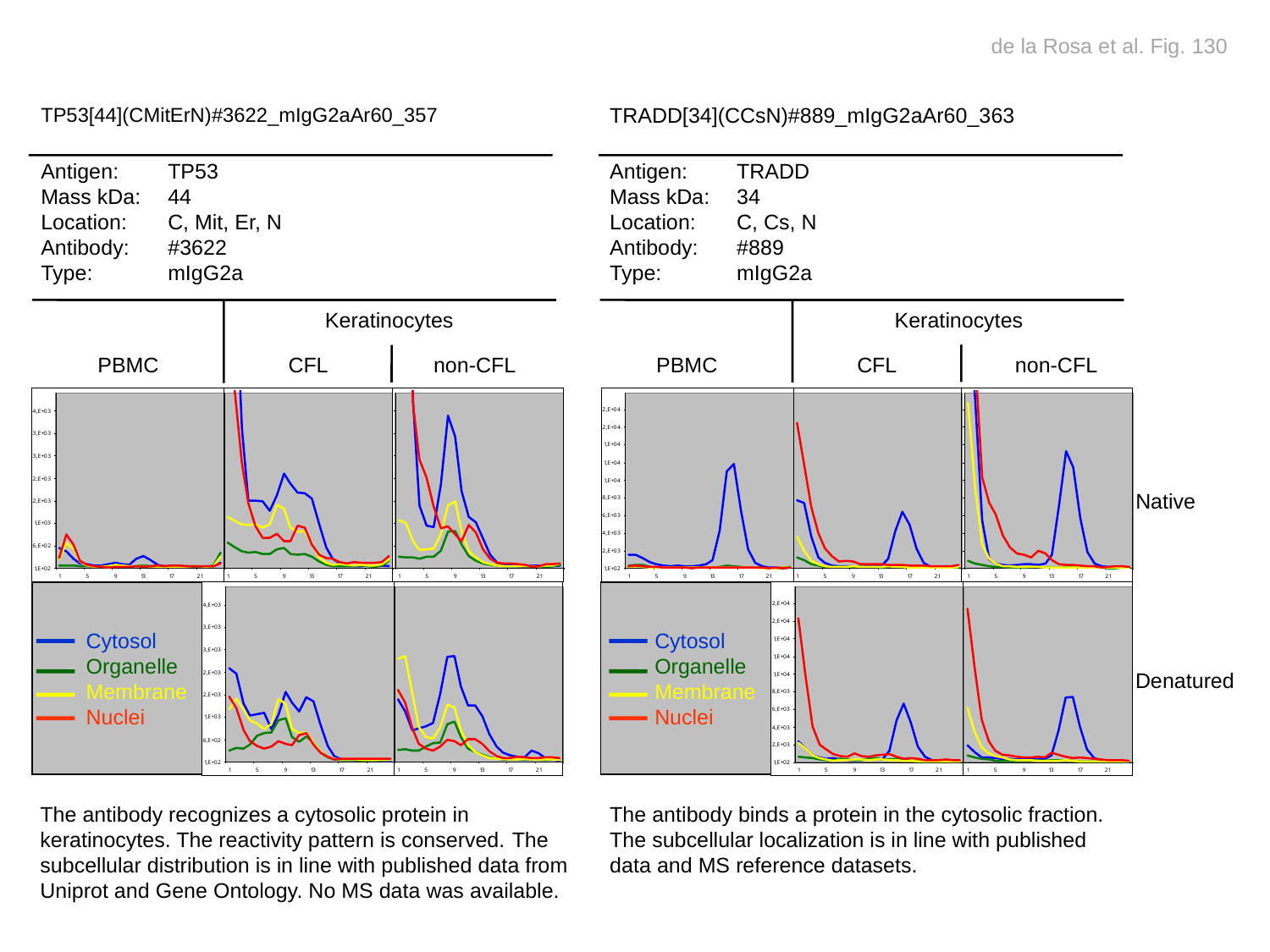

de la Rosa et al. Fig. <number>
# TP53[44](CMitErN)#3622_mIgG2aAr60_357
TRADD[34](CCsN)#889_mIgG2aAr60_363
Antigen: 	TP53
Mass kDa:	44
Location: 	C, Mit, Er, N
Antibody: 	#3622
Type:	mIgG2a
Antigen: 	TRADD
Mass kDa:	34
Location: 	C, Cs, N
Antibody: 	#889
Type:	mIgG2a
The antibody recognizes a cytosolic protein in keratinocytes. The reactivity pattern is conserved. The subcellular distribution is in line with published data from Uniprot and Gene Ontology. No MS data was available.
The antibody binds a protein in the cytosolic fraction. The subcellular localization is in line with published data and MS reference datasets.

## Slide 131
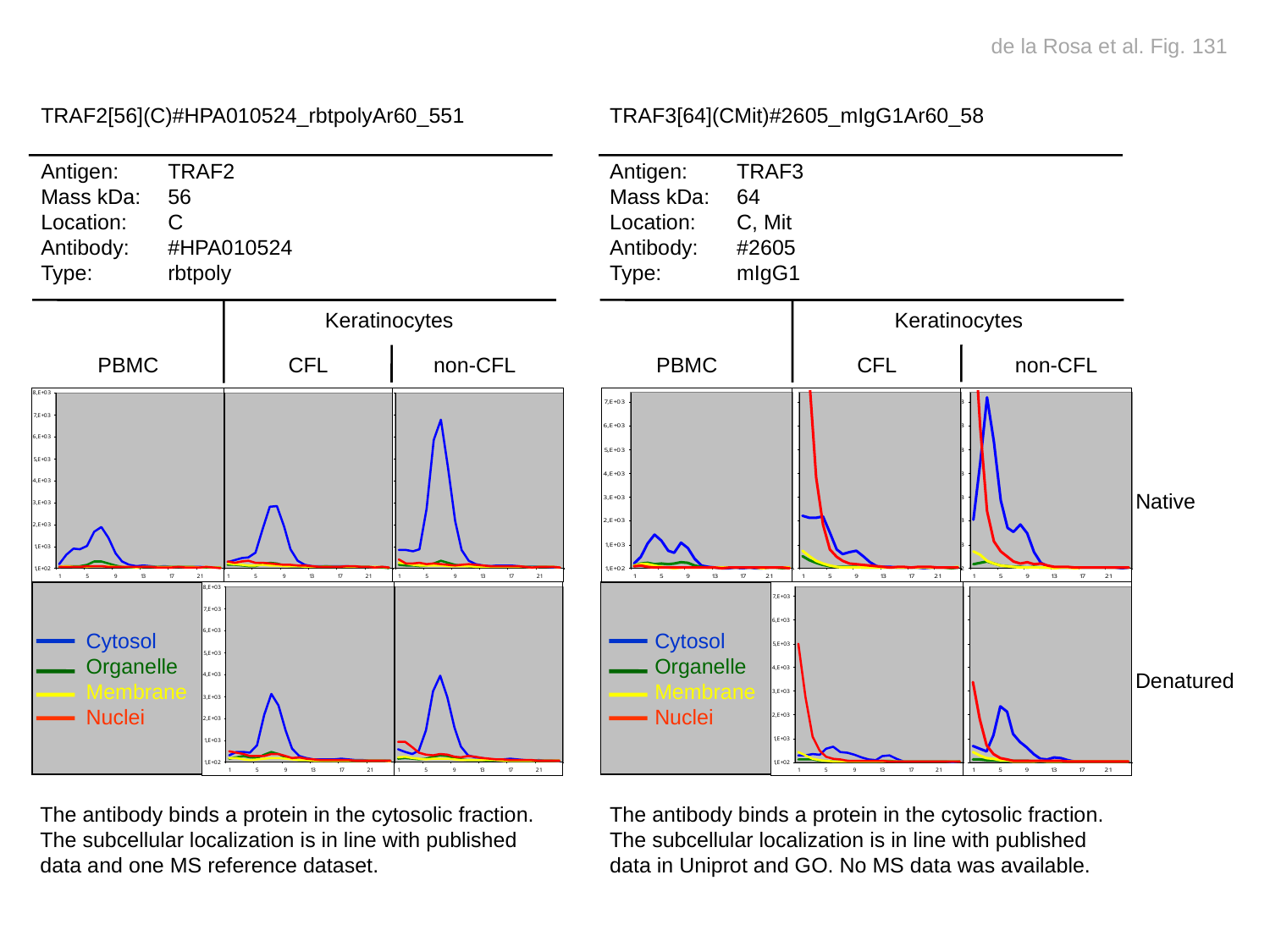

de la Rosa et al. Fig. <number>
# TRAF2[56](C)#HPA010524_rbtpolyAr60_551
TRAF3[64](CMit)#2605_mIgG1Ar60_58
Antigen: 	TRAF2
Mass kDa:	56
Location: 	C
Antibody: 	#HPA010524
Type:	rbtpoly
Antigen: 	TRAF3
Mass kDa:	64
Location: 	C, Mit
Antibody: 	#2605
Type:	mIgG1
The antibody binds a protein in the cytosolic fraction. The subcellular localization is in line with published data and one MS reference dataset.
The antibody binds a protein in the cytosolic fraction. The subcellular localization is in line with published data in Uniprot and GO. No MS data was available.

## Slide 132
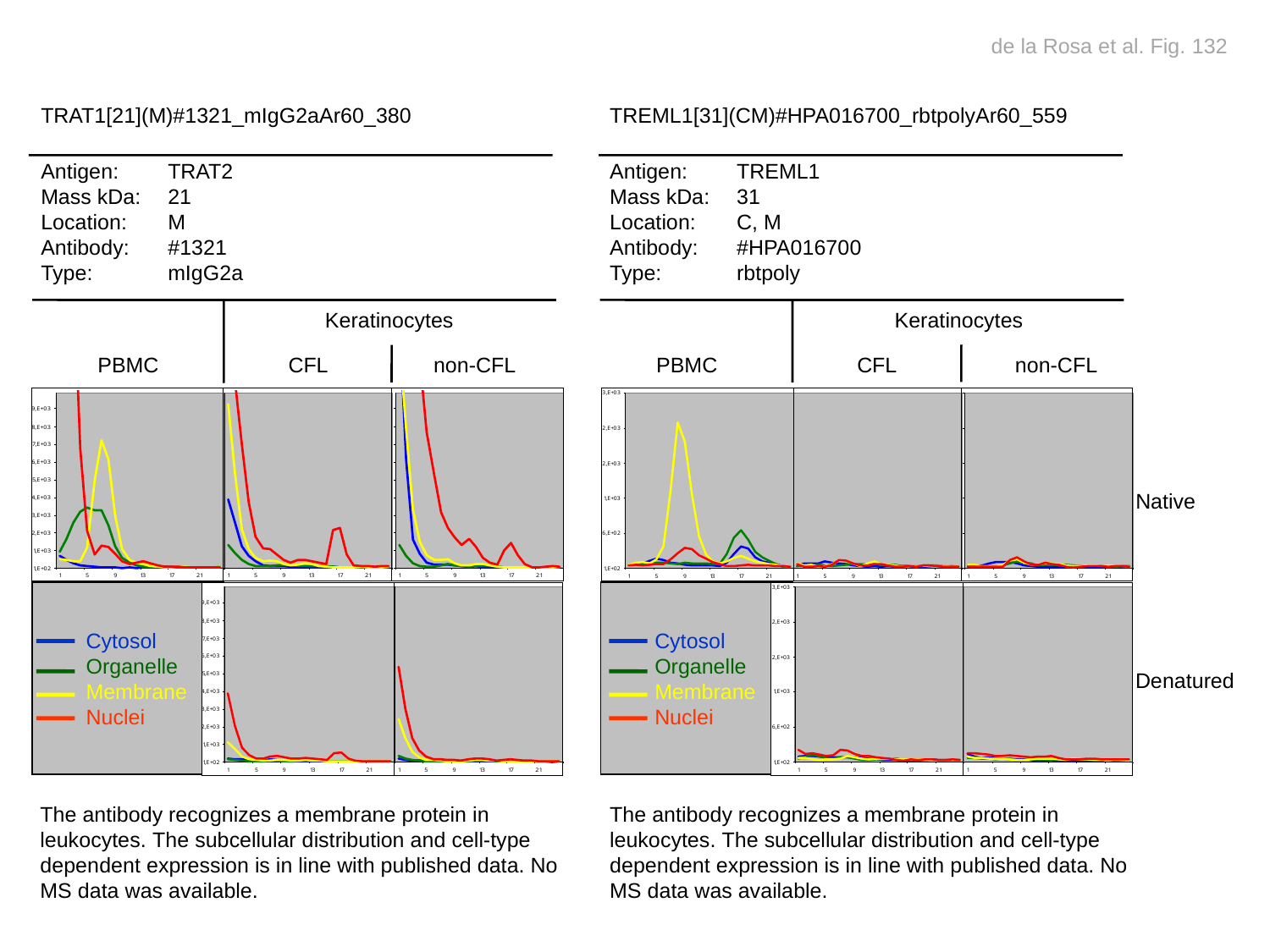

de la Rosa et al. Fig. <number>
# TRAT1[21](M)#1321_mIgG2aAr60_380
TREML1[31](CM)#HPA016700_rbtpolyAr60_559
Antigen: 	TRAT2
Mass kDa:	21
Location: 	M
Antibody: 	#1321
Type:	mIgG2a
Antigen: 	TREML1
Mass kDa:	31
Location: 	C, M
Antibody: 	#HPA016700
Type:	rbtpoly
The antibody recognizes a membrane protein in leukocytes. The subcellular distribution and cell-type dependent expression is in line with published data. No MS data was available.
The antibody recognizes a membrane protein in leukocytes. The subcellular distribution and cell-type dependent expression is in line with published data. No MS data was available.

## Slide 133
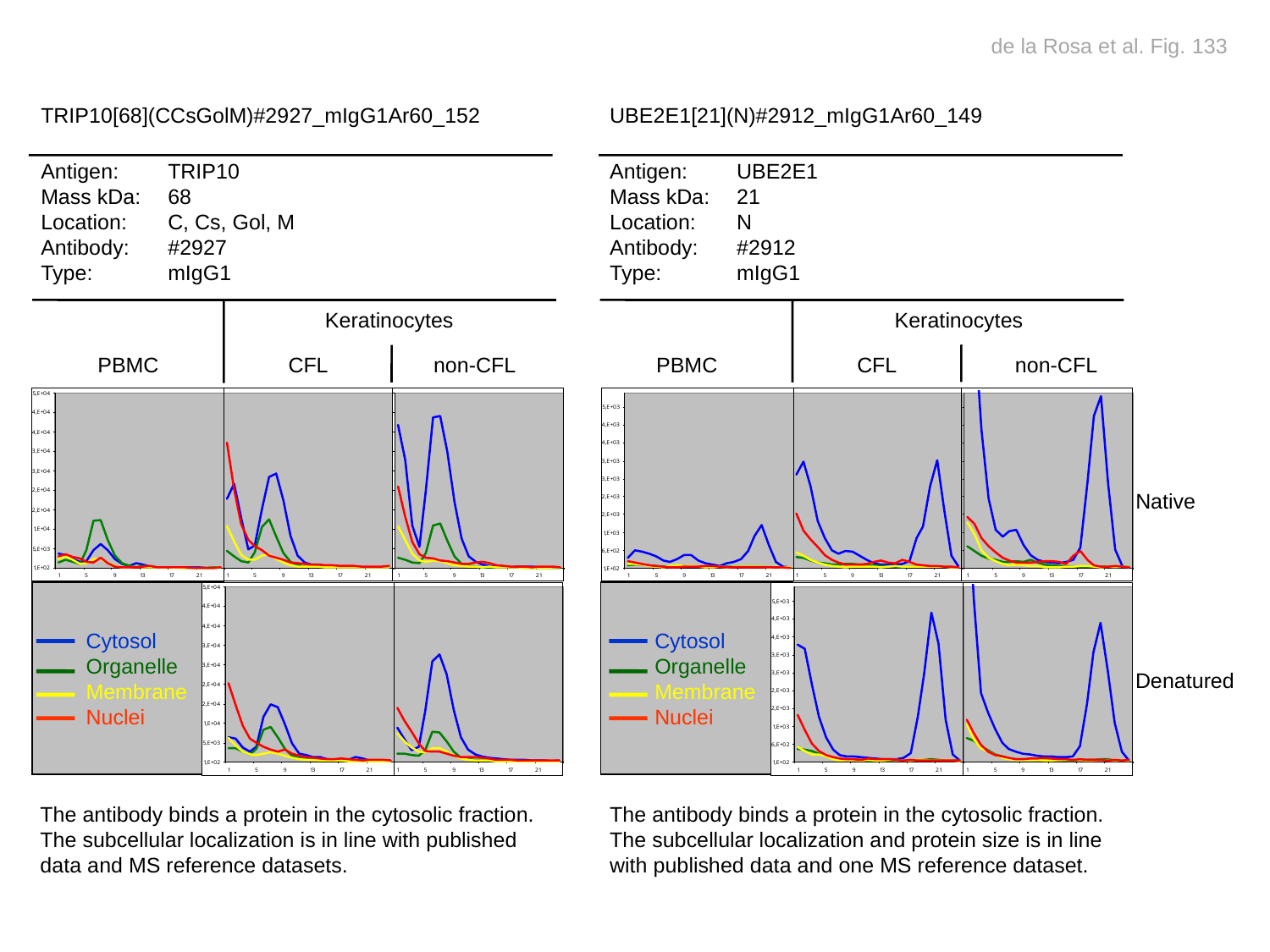

de la Rosa et al. Fig. <number>
# TRIP10[68](CCsGolM)#2927_mIgG1Ar60_152
UBE2E1[21](N)#2912_mIgG1Ar60_149
Antigen: 	TRIP10
Mass kDa:	68
Location: 	C, Cs, Gol, M
Antibody: 	#2927
Type:	mIgG1
Antigen: 	UBE2E1
Mass kDa:	21
Location: 	N
Antibody: 	#2912
Type:	mIgG1
The antibody binds a protein in the cytosolic fraction. The subcellular localization is in line with published data and MS reference datasets.
The antibody binds a protein in the cytosolic fraction. The subcellular localization and protein size is in line with published data and one MS reference dataset.

## Slide 134
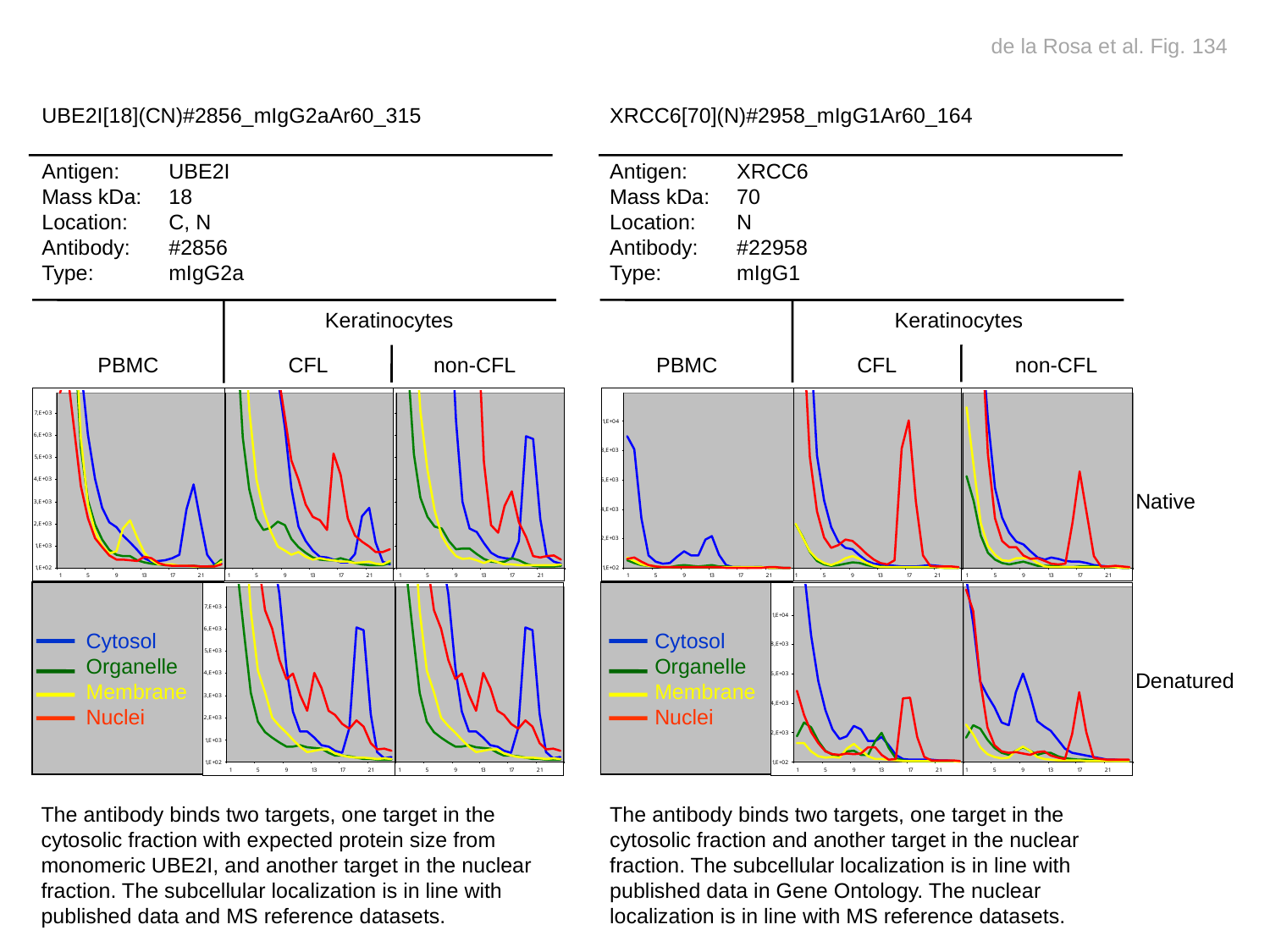

de la Rosa et al. Fig. <number>
UBE2I[18](CN)#2856_mIgG2aAr60_315
# XRCC6[70](N)#2958_mIgG1Ar60_164
Antigen: 	UBE2I
Mass kDa:	18
Location: 	C, N
Antibody: 	#2856
Type:	mIgG2a
Antigen: 	XRCC6
Mass kDa:	70
Location: 	N
Antibody: 	#22958
Type:	mIgG1
The antibody binds two targets, one target in the cytosolic fraction with expected protein size from monomeric UBE2I, and another target in the nuclear fraction. The subcellular localization is in line with published data and MS reference datasets.
The antibody binds two targets, one target in the cytosolic fraction and another target in the nuclear fraction. The subcellular localization is in line with published data in Gene Ontology. The nuclear localization is in line with MS reference datasets.

## Slide 135
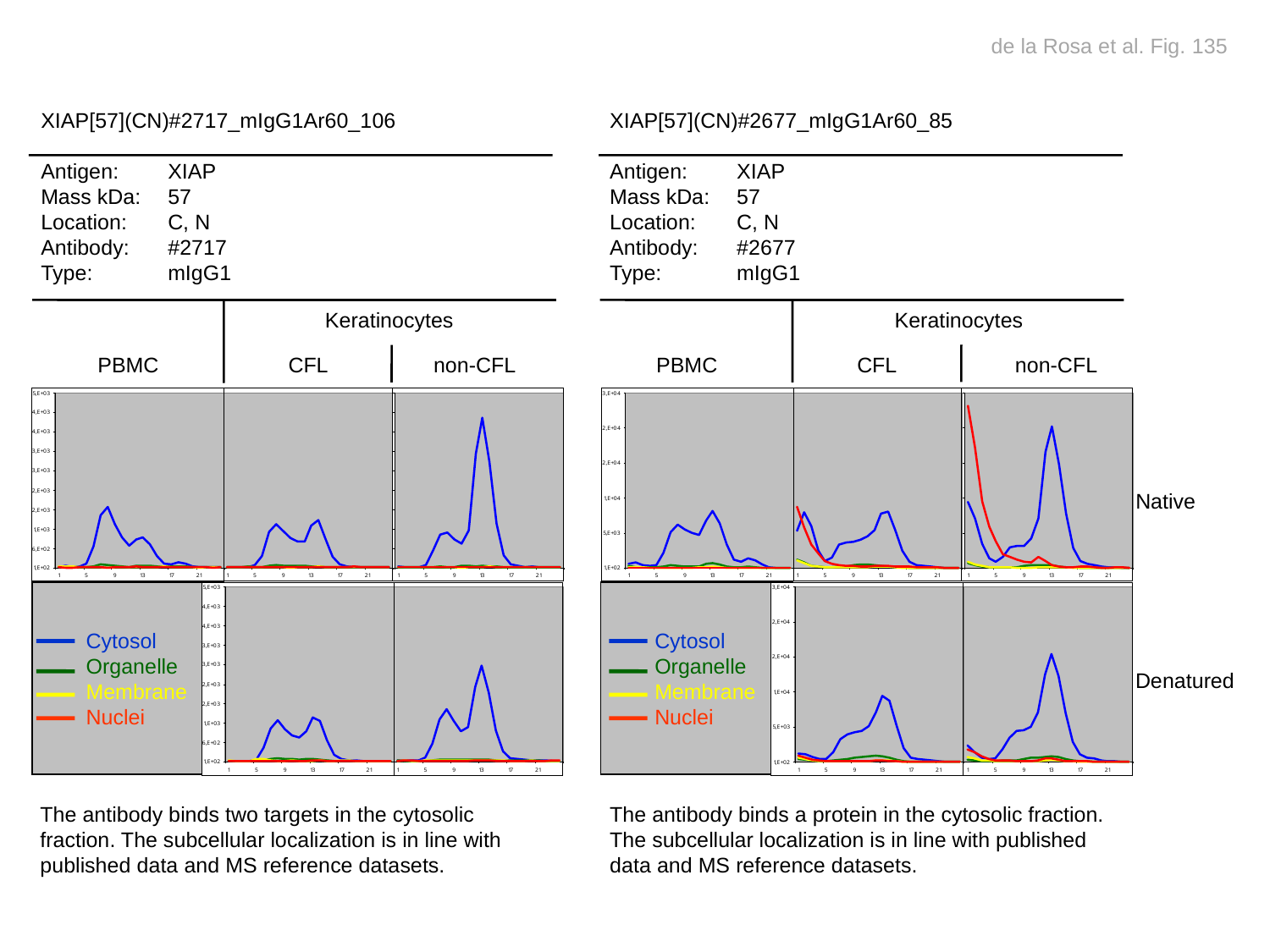

de la Rosa et al. Fig. <number>
# XIAP[57](CN)#2717_mIgG1Ar60_106
XIAP[57](CN)#2677_mIgG1Ar60_85
Antigen: 	XIAP
Mass kDa:	57
Location: 	C, N
Antibody: 	#2717
Type:	mIgG1
Antigen: 	XIAP
Mass kDa:	57
Location: 	C, N
Antibody: 	#2677
Type:	mIgG1
The antibody binds two targets in the cytosolic fraction. The subcellular localization is in line with published data and MS reference datasets.
The antibody binds a protein in the cytosolic fraction. The subcellular localization is in line with published data and MS reference datasets.

## Slide 136
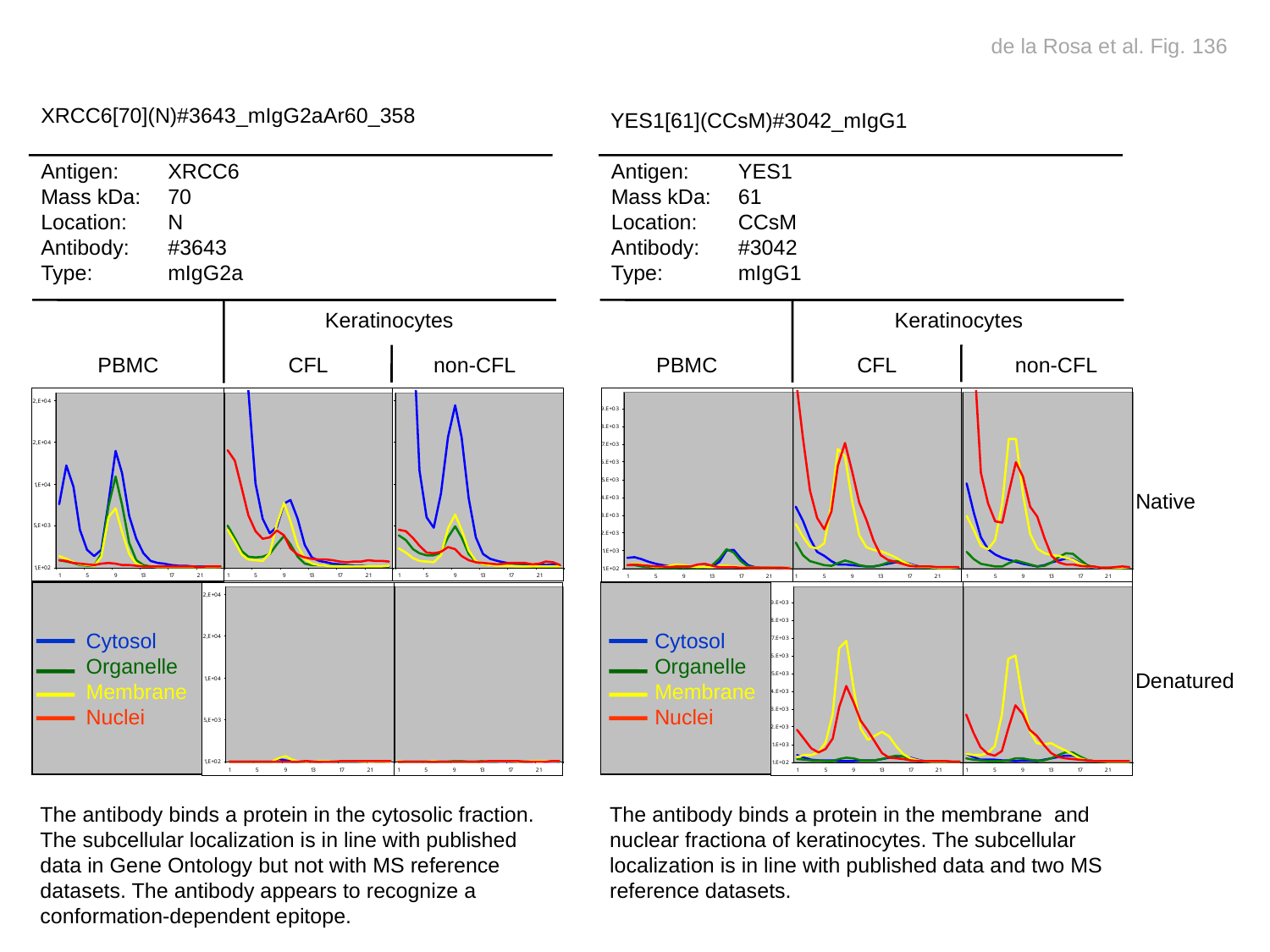

de la Rosa et al. Fig. <number>
# XRCC6[70](N)#3643_mIgG2aAr60_358
YES1[61](CCsM)#3042_mIgG1
Antigen: 	XRCC6
Mass kDa:	70
Location: 	N
Antibody: 	#3643
Type:	mIgG2a
Antigen: 	YES1
Mass kDa:	61
Location: 	CCsM
Antibody: 	#3042
Type:	mIgG1
The antibody binds a protein in the cytosolic fraction. The subcellular localization is in line with published data in Gene Ontology but not with MS reference datasets. The antibody appears to recognize a conformation-dependent epitope.
The antibody binds a protein in the membrane and nuclear fractiona of keratinocytes. The subcellular localization is in line with published data and two MS reference datasets.

## Slide 137
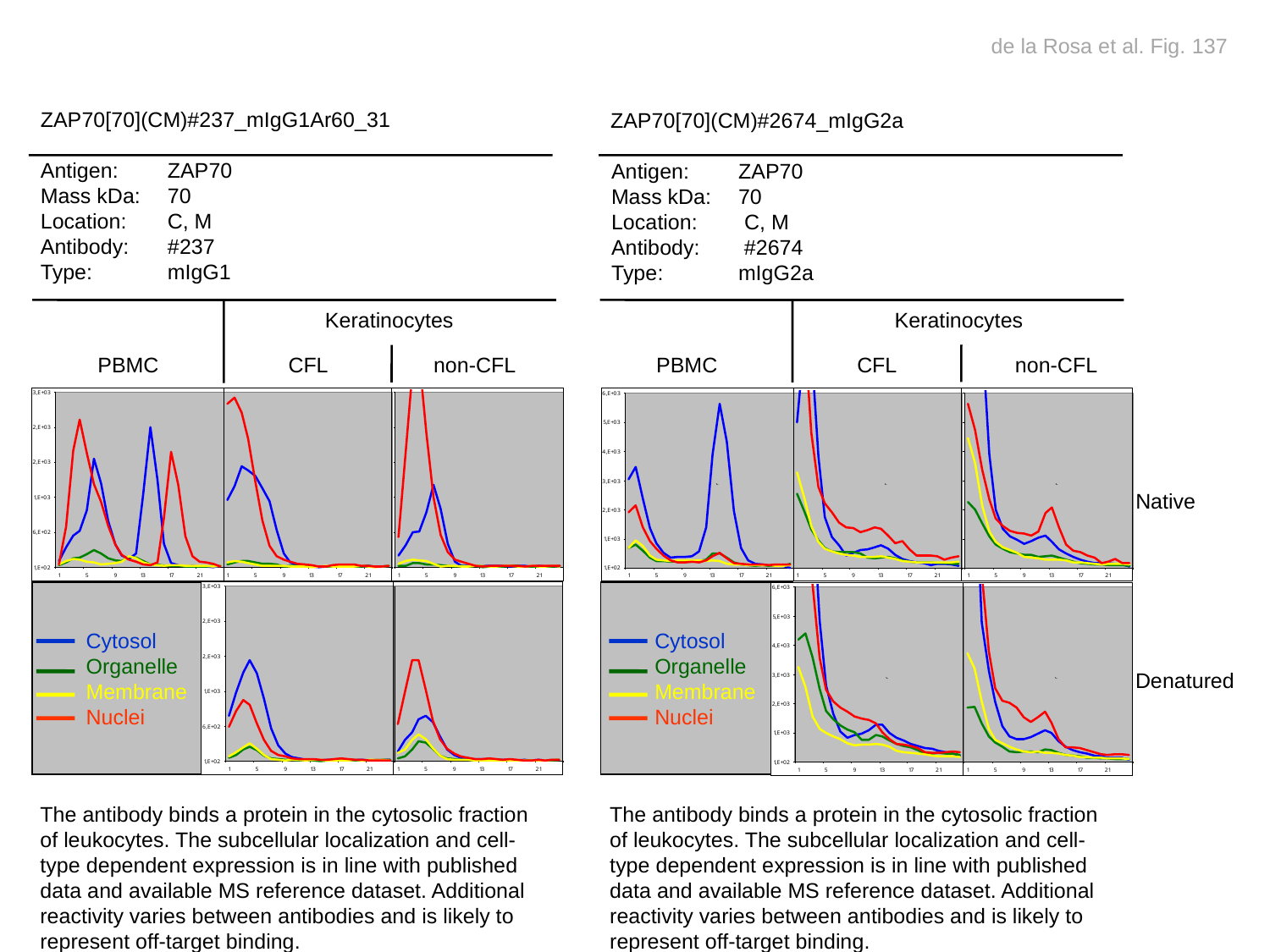

de la Rosa et al. Fig. <number>
# ZAP70[70](CM)#237_mIgG1Ar60_31
ZAP70[70](CM)#2674_mIgG2a
Antigen: 	ZAP70
Mass kDa:	70
Location: 	C, M
Antibody: 	#237
Type:	mIgG1
Antigen: 	ZAP70
Mass kDa:	70
Location: 	 C, M
Antibody: 	 #2674
Type:	mIgG2a
The antibody binds a protein in the cytosolic fraction of leukocytes. The subcellular localization and cell-type dependent expression is in line with published data and available MS reference dataset. Additional reactivity varies between antibodies and is likely to represent off-target binding.
The antibody binds a protein in the cytosolic fraction of leukocytes. The subcellular localization and cell-type dependent expression is in line with published data and available MS reference dataset. Additional reactivity varies between antibodies and is likely to represent off-target binding.

## Slide 138
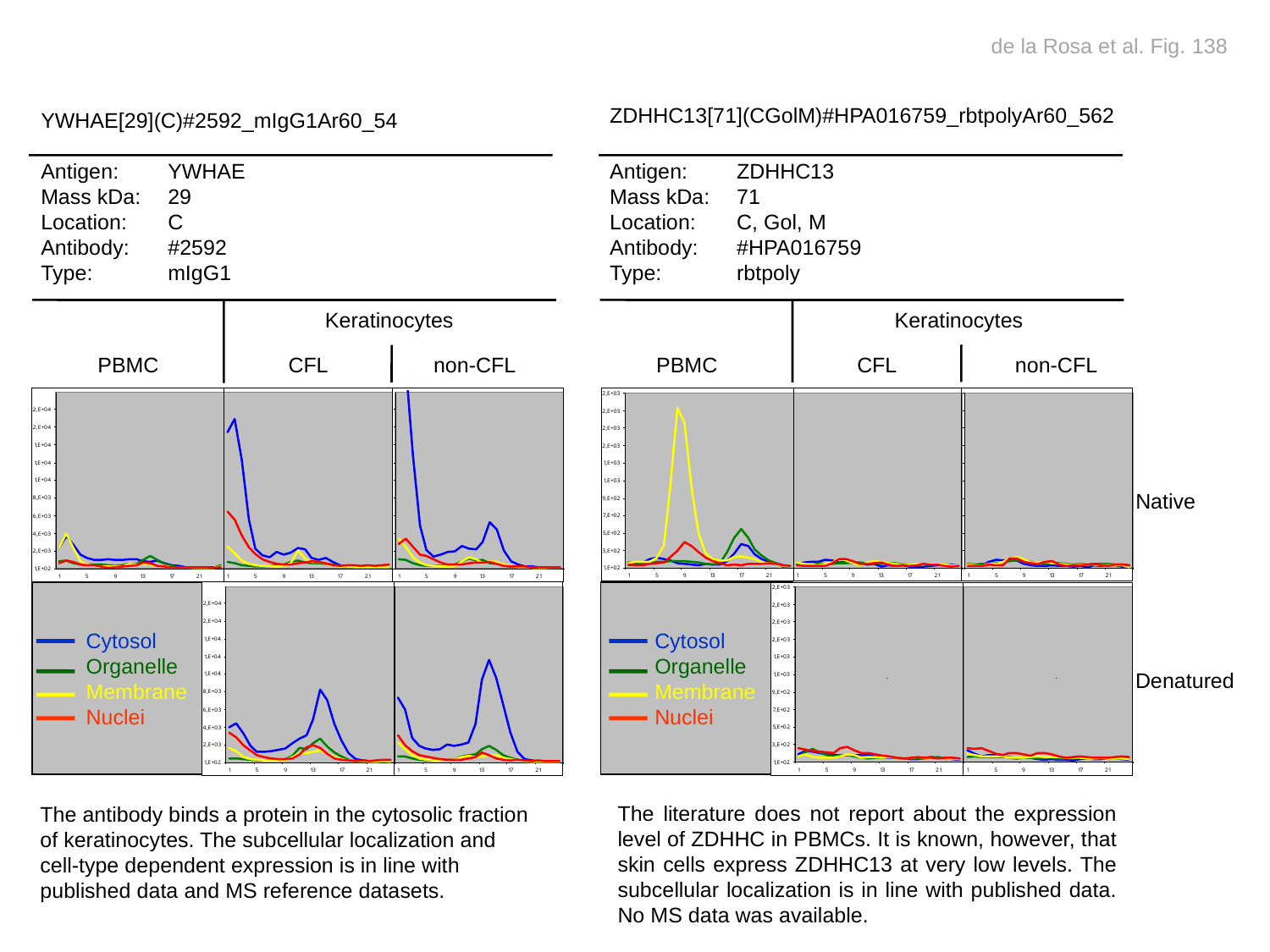

de la Rosa et al. Fig. <number>
YWHAE[29](C)#2592_mIgG1Ar60_54
# ZDHHC13[71](CGolM)#HPA016759_rbtpolyAr60_562
Antigen: 	YWHAE
Mass kDa:	29
Location: 	C
Antibody: 	#2592
Type:	mIgG1
Antigen: 	ZDHHC13
Mass kDa:	71
Location: 	C, Gol, M
Antibody: 	#HPA016759
Type:	rbtpoly
The literature does not report about the expression level of ZDHHC in PBMCs. It is known, however, that skin cells express ZDHHC13 at very low levels. The subcellular localization is in line with published data. No MS data was available.
The antibody binds a protein in the cytosolic fraction of keratinocytes. The subcellular localization and cell-type dependent expression is in line with published data and MS reference datasets.
